# Proteomic and metabolic traits of grape exocarp to explain different anthocyanin concentrations of the cultivars

**DOI:** 10.3389/fpls.2015.00603

**Published:** 2015-08-04

**Authors:** Alfredo S. Negri, Bhakti Prinsi, Osvaldo Failla, Attilio Scienza, Luca Espen

**Affiliations:** Dipartimento di Scienze Agrarie e Ambientali, Produzione, Territorio, Agroenergia, Università degli Studi di MilanoMilano, Italy

**Keywords:** anthocyanins, exocarp grape berry, metabolomics, proteomics, stress response, *Vitis vinifera*

## Abstract

The role of grape berry skin as a protective barrier against damage by physical injuries and pathogen attacks requires a metabolism able to sustain biosynthetic activities such as those relating to secondary compounds (i.e., flavonoids). In order to draw the attention on these biochemical processes, a proteomic and metabolomic comparative analysis was performed among Riesling Italico, Pinot Gris, Pinot Noir, and Croatina cultivars, which are known to accumulate anthocyanins to a different extent. The application of multivariate statistics on the dataset pointed out that the cultivars were distinguishable from each other and the order in which they were grouped mainly reflected their relative anthocyanin contents. Sorting the spots according to their significance 100 proteins were characterized by LC-ESI-MS/MS. Through GC-MS, performed in Selected Ion Monitoring (SIM) mode, 57 primary metabolites were analyzed and the differences in abundance of 16 of them resulted statistically significant to ANOVA test. Considering the functional distribution, the identified proteins were involved in many physiological processes such as stress, defense, carbon metabolism, energy conversion and secondary metabolism. The trends of some metabolites were related to those of the protein data. Taken together, the results permitted to highlight the relationships between the secondary compound pathways and the main metabolism (e.g., glycolysis and TCA cycle). Moreover, the trend of accumulation of many proteins involved in stress responses, reinforced the idea that they could play a role in the cultivar specific developmental plan.

## Introduction

Grape berry of the perennial and deciduous woody vines of the genus *Vitis* is one of the economically most important fruit crop in the world. As recounted in the 2012 report of Food and Agriculture Organization (FAO, http://faostat.fao.org/site/567/DesktopDefault.aspx?PageID=567#ancor), 69.7694 hectares are dedicated to the cultivation of grapevine (*Vitis vinifera* L), with an estimation of a production of about 67 million tons per year.

During recent years, there was a burst of genomic information about the development and ripening in grape berries. After the first pioneer gene-specific molecular approaches, the appearance of large collections of grapevine ESTs (Ablett et al., 2000) and the construction of grapevine nucleotide microarrays (Terrier et al., 2005; Waters et al., 2005) opened new horizons in the study of grape berry ripening (Grimplet et al., 2007; Zamboni et al., 2010; Fortes et al., 2011). Moreover, the work of grape genome sequencing by Jaillon et al. (2007) has contributed to provide the necessary genomic information (on 2th July 2015, 94,556 protein sequences were available in the NCBI database, http://www.ncbi.nlm.nih.gov/sites/entrezz). These studies paved the way for investigating berry proteome (Deytieux et al., 2007; Giribaldi et al., 2007; Negri et al., 2008a,b, 2011; Grimplet et al., 2009; Giribaldi and Giuffrida, 2010; Zamboni et al., 2010; Martínez-Esteso et al., 2011; Niu et al., 2013; Fraige et al., 2015).

Grape is a non-climacteric fruit formed by three major tissues: exocarp (i.e., skin), mesocarp (pulp) and endocarp which surrounds seeds. Exocarp represents a physical barrier between the external environment and the inner tissues, protecting them by physical damage and pathogen attack (Grimplet et al., 2007). It is metabolically active during all developing phases being the site of the synthesis of exclusive compounds, such as aroma and some phenolic classes. Aromas arise from volatile molecules, such as terpenes, norisoprenoids and thiols that are usually stored as amino acid and sugar conjugates in the vacuole (Lund and Bohlman, 2006). Among phenolic compounds synthetized in the exocarp cells there are anthocyanins. Their color promotes seed dispersal thanks to the high contrast between background foliage and fruits (Burns and Dalen, 2002). Moreover, these compounds are involved in the protection from UV light exposure (Solovchenko and Schmitz-Eiberger, 2003). The biosynthesis of these compounds begins at *véraison* and continues throughout the ripening phase. The levels of anthocyanins are influenced by many factors, such as genetic background, pedo-climatic conditions and vineyard management while their profiles are relatively stable for each variety (Mattivi et al., 2006; Castellarin et al., 2012; Zheng et al., 2013). The studies on the regulation of anthyocyanin pathway revealed that the synthesis of these compounds requires the UDP-glucose flavonoid glycosyl transferase (UFGT) expression. Northern blot analysis conducted on the exocarp of a range of white and red cultivars showed, in fact, that the transcript of this enzyme was detectable only in colored grapes (Boss et al., 1996a,b). Moreover, by the isolation of several *myb*-related genes from berries, it was shown that the lack of expression of *VvmybA1* in white cultivars results from the insertion of a retrotransposon in its promoter region absent in red cultivars, thus suggesting that the expression of this transcription factor may be the trigger of color set in grape (Kobayashi et al., 2004). Nevertheless, Ageorges et al. (2006) found that in addition to UFGT, at least three other isogenes related to the anthocyanin pathway, such as chalcone synthase 3 (CHS3) and the downstream elements glutathione S-transferase (GST) and caffeoyl methyl transferase (CaoMT) can be clearly associated to color in grape berries.

In the last years, studies performed on separate tissues pointed out several peculiar traits in gene expression, according to their functional role. As showed by the comparison among the main mature berry tissues of cultivar Cabernet Sauvignon, through the use of Affymetrix GeneChip® technology, more than 28% of the genes with significant differential expression showed differences in a particular tissue (Grimplet et al., 2007). The most expressed transcripts in the exocarp were those relating to secondary metabolism, amino acid and lipid metabolism. Moreover, some transcripts that related to some enzymes involved in carbon metabolism were over-represented in this tissue as well as some pathogenesis-related proteins were more abundant in the exocarp at the maturity (Deytieux et al., 2007; Grimplet et al., 2007). A further proteomic investigation performed on exocarp of cultivar Barbera showed that during the final ripening stage an increase in abundance of enzymes involved in primary metabolism, such as the glycolytic pathway, occurred (Negri et al., 2008b).

As expected, some -omic studies revealed that the environmental conditions, such as water availability or sunlight exclusion, deeply affected the metabolism of skin tissue (Grimplet et al., 2007, 2009; Niu et al., 2013; Zheng et al., 2013). Furthermore, a study, in which metabolite and transcript profiling of berry skin of Cabernet Sauvignon and Shiraz were compared, found that there were peculiar metabolic differences between the two cultivars (Degu et al., 2014).

The aim of the present work was to investigate possible relationships among the main biochemical pathways characterizing grape berry ripening and anthocyanin accumulation. For this purpose, four grape cultivars with increasing anthocyanin contents [Riesling Italico (synonymous: Welschriesling) (R), Pinot Gris (PG), Pinot Noir (PN), and Croatina (C)] were compared at the mature berry stage. To mitigate the seasonal effects, the study was conducted analyzing samples harvested in two different years and was performed combining a proteomic analysis (i.e., 2-DE gels/ LC-ESI-MS/MS) with a metabolomic one (i.e., GC-MS). Using multivariate statistical analysis (i.e., FS-LDA) on spot volume dataset, it was possible to discriminate the differences among the four cultivars and to sort the matches according to their discriminating power.

Through the integration of proteomic and metabolomic analyses, this work provided new insights on the ripening process in the skin and on the grape heterogeneity. The results clearly showed that the process of grape ripening in the skin differs among cultivars in some central metabolic traits. These variations appeared to be linked to the different trends of accumulation of secondary metabolites, but they appeared also related to the ripening plan.

## Materials and methods

### Plant material

Exocarps were harvested during the 2005 and 2006 seasons from *Vitis vinifera* L. cv. R, PG, PN, and C RS38 berries (full-ripe berries according to modified E–L system, Coombe, 1995). The plants were grown at the Experimental Station of the “Ente Regionale per i Servizi all'Agricoltura e alle Foreste” (ERSAF) of Regione Lombardia (Montebello della Battaglia, PV, Italy).

About 50 g of isolated skins of at least five different randomly chosen plants of one cultivar were detached immediately by squishing the berries in order to remove the seeds and the bulk of the mesocarp. By pressing and smearing the inner part of the skin on two layers of cheesecloth the residual pulp was completely taken away. This operation was repeated three times and each pool, representing a biological replicate (4 cultivars × 2 years × 3 replicas = 24 pools), was frozen in liquid nitrogen, ground and stored at −80°C until use. From each powder pool both proteomic (5 g/extraction) and metabolomic (150 g/extraction) technical replicates were obtained.

### Evaluation of anthocyanin contents

Anthocyanins were extracted and measured as previously described by Fumagalli et al. (2006) and Negri et al. (2008b), respectively.

### Protein extraction and two-dimensional polyacrylamide gel electrophoresis analysis

Protein fraction was extracted using the method previously described by Negri et al. (2008b) with two modifications. In detail: (i) the cold acetone powder was firstly resuspended in phenol and then incubated for 30 min at 4°C, afterwards an equal volume of extraction buffer was added to proceed with repartition step (Hurkman and Tanaka, 1986); (ii) proteins were resuspended in the IEF pH 4–7 buffer (GE Healthcare).

Protein concentration was determined by 2-D Quant Kit (GE Healthcare). Five-hundred μg of protein sample was used for each 2-DE analysis that was performed using the pH 4–7, 24 cm IPG strips (GE Healthcare) as previously described in Negri et al. (2008b).

The gels were stained according to colloidal Coomassie Brilliant Blue G-250 (cCBB) procedure, (Neuhoff et al., 1988). The gels were then scanned by an Epson Expression 1680 Pro Scanner. For each of the 6 biological replicates of a sample (3 per year), two 2-DE gels were obtained (*n* = 12).

### Gel and proteomic data analysis

Gels were analyzed with ImageMaster 2-D Platinum Software v. 6.0 (GE Healthcare) matching the gels using the landmark-assisted procedure with one of the 48 gels as reference. Since the relevant number of gels and the possibility of spreading the matching errors, an incremental checking method was set up. Briefly, a sub-reference gel was chosen for the group of maps relating to the skins of the same cultivar and year. The spots of the sub-reference gel were ordered according to their decreasing *%Vol* and only the matching of the 1000 largest spots were checked. The procedure was repeated for all the 8 distinct samples taking into account only their relative 6 gels. In the second step, the analysis comprised the 12 maps of a single cultivar obtained from the skins of both years. Also in this case, only the matches of the 1000 largest spots of the sub-reference gels were evaluated. Similarly, the gels of the different pairs of cultivars were compared. Only in the final step the check was performed considering all the 48 maps. In addition to the cutting off spots of really low abundance, matches grouping less than 3 spots in a sample were discarded. Through this procedure it was possible to eliminate a relevant part of noise in the dataset and to rescue significant matches that are often lost during automatic matching.

The molecular weight and pI of the spots were estimated as previously described by Negri et al. (2008b).

The differences among the four cultivars were assessed analyzing the *%Vol* dataset through the application of Principal Component Analysis (PCA) and Forward Stepwise Linear Discriminant Analysis (FS-LDA) on the first 10 PCA scores as previously described by Negri et al. (2011).

Significant differences relative to identified proteins were analyzed through the one-way hierarchical clustering methodology using the software PermutMatrix (Caraux and Pinloche, 2005; Meunier et al., 2007). The 2-DE data were converted into a binary matrix replacing the missing values by zero. The row by row normalization of data was performed using the classical zero-mean and unit-standard deviation technique. Pearson's distance and Ward's algorithm were used for the analysis.

### Protein in-gel digestion and LC-ESI-MS/MS analysis

Spots excised from gels stained with cCBB were digested as described by Prinsi et al. (2011).

The LC-ESI-MS/MS experiments were conducted using a Surveyor (MS pump Plus) HPLC system directly connected to the ESI source of a Finnigan LCQ DECA XP MAX ion trap mass spectrometer (ThermoFisher Scientific Inc). Chromatography separations were performed by using an Inerstil WP300 C18 column (200 μm I.D × 150 mm length, 5 μm particle size) and a gradient from 5% to 80% solvent B [solvent A: 0.1% (v/v) formic acid; solvent B: ACN containing 0.1% (v/v) formic acid] as described by Niessen and co-workers (Niessen et al., 2006), with a flow of 2 μl min^−1^. ESI was performed in positive ionization mode with the following parameters: (i) spray voltage: 2.5 kV (ii) capillary temperature: 220°C. Data were collected in the full-scan and data dependent MS/MS mode with collision energy of 35% and a dynamic exclusion window of 3 min.

Protein identification was performed by Spectrum Mill MS Proteomics Workbench (Rev B.04.00.127; Agilent Technologies). Cysteine carbamidomethylation and methionine oxidation were set as fixed and variable modifications, respectively, accepting two missed cleavages per peptide. The search was conducted against the subset of *Vitis* protein sequences (ID 3603; June 2015, 94558 *entries*) downloaded from the National Center for Biotechnology Information (http://www.ncbi.nlm.nih.gov/) and concatenated with the reverse one. The threshold used for peptide identification was Spectrum Mill score >10, Score Peak Intensity ≥70%, mass tolerance of ±2 Da for parent ion and ±1 Da fragment ions, and Database Fwd-Rev Score ≥2. Physical properties of the proteins were predicted by *in silico* tools at ExPASy (http://web.expasy.org/compute_pi/).

The identified proteins were sorted in metabolic functional classes according to the MapMan *BIN* ontology. The mass spectrometry proteomics data have been deposited to the ProteomeXchange Consortium (Vizcaíno et al., 2014) via the PRIDE partner repository with the dataset identifier PXD002539.

### Metabolite extraction and derivatization

In order to extract the metabolites of the polar fraction, the protocol by Lisec et al. (2006) was used with some modifications. One hundred-fifty milligrams (150 mg) of the frozen powder relative to the 6 biological replicates (3 per year) were resuspended in 1.4 ml of −20°C cooled methanol and 60 μl of 2 mg ml^−1^ ribitol were added as internal standard. The samples were incubated at 70°C for 15 min in continuous agitation (1200 rpm) with a thermo mixer and subsequently centrifuged for 10 min at 11,000 g at room temperature. After recovering the surnatant, 750 μl of chloroform and 1.5 ml of distilled water were added. The samples were vortexed and then centrifuged for 15 min at 2200 g at 4°C in order to separate the phases with different polarity. Aliquots of 150 μl of the water/methanol supernatant were then transferred to a clean eppendorf tube and dried on a Speedvac (RVC 2–18 CDplus, CHRIST) without heating for 16 h. The dried residues were redissolved in 40 μl of methoxyamination reagent (20 mg ml^−1^ of metoxyamine hydrochloride in pyridine) at 30°C and 700 rpm for 2 h and derivatized in 60 μl of N-methyl-N-(trimethylsilyl)-trifluoroacetamide (MSTFA) in the same conditions for 6 h. A retention time standard (10 μl), obtained diluting a saturated C7–C40 Alkane Mixture (Supelco, 1000 μg/mL for each component) 1:20 in MSTFA, was added to each sample. Before further analysis, the samples were transferred in glass vials. For each biological replicate, the analysis was repeated twice.

The employed standard substances were dissolved in distilled water or methanol at the concentration of 10 mg ml^−1^ and 1 μl was dried in vacuum and derivatized as described above to get spectral information.

### GC-MS analysis and data processing

Metabolite quantification was conducted using the instrument GC-MSD comprising the gas chromatograph 7890 and the single-quadrupole spectrometer 5975 (Agilent Technologies). The employed method was the one defined by Golm Metabolome Database (http://csbdb.mpimp-golm.mpg.de/cgi-bin/madb2ml.cgi?org=msri&c=ml&o=ht&typ=met&inp=m%5B2%5D) and here briefly summarized. One microliter of sample was injected at 230°C in splitless mode through the autosampler CTC PAL (CTC Analytics AG). The analysis was conducted using a 30 m × 0.25 mm ID × 0.25 μm film thickness DB-5 column (Agilent Technologies). Helium BIP™ (Sapio) was used as carrier gas with a constant flux of 1 ml min^−1^. The oven ramp was so set up: 1 min at 70°C, 6 min ramp to 76°C, 45 min ramp to 350°C, 1 min at 350°C, 10 min at 330°C. The spectra and the retention times of the standard substances were acquired in a m/z range between 40 and 600. Metabolite analysis was performed in SIM mode following a maximum of 7 ions per time-subgroup and setting a dwell time of 20 ms. The MS source and quad were maintained at 230°C and 150°C, respectively, using the ionization for electronic impact at −70 eV.

Spectral integration was carried out through the software MetaQuant 1.3 (Bunk et al., 2006).

Integrated peaks were normalized by the peak area of the ion with m/z = 219 of ribitol. Data were Box-Cox transformed and the differences were evaluated though ANOVA with *p*≤0.05 using the software STATISTICA v. 7.1 (StatSoft Inc., Tulsa, OK, USA).

## Results

### Anthocyanin contents

**Figure 1:**
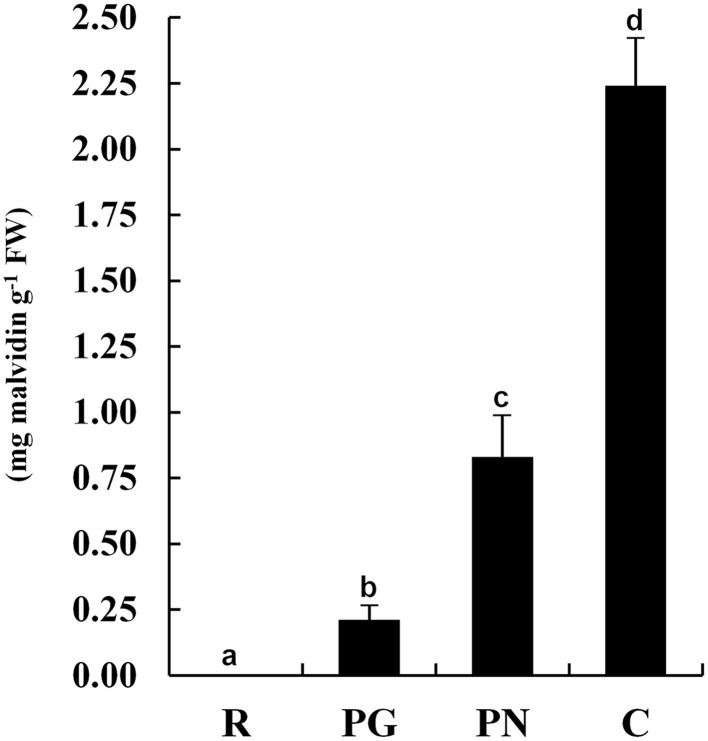
**Anthocyanin contents in grape berry exocarp of Riesling Italico, Pinot Gris, Pinot Noir, and Croatina**. R, Riesling Italico; PG, Pinot Gris; PN, Pinot Noir; C, Croatina. Data are the means ± ES, *n* = 6. Samples indicated with the different letters significantly differ according to Tukey's test (*p* < 0.01).

The anthocyanin content of the exocarp berry of four cultivars (Figure 1) moved from the undetectable levels of R to the high levels of C (2.25 mg g^−1^) passing through the pale PG (0.21 mg g^−1^) and PN (0.83 mg g^−1^).

### 2-DE analysis

**Figure 2:**
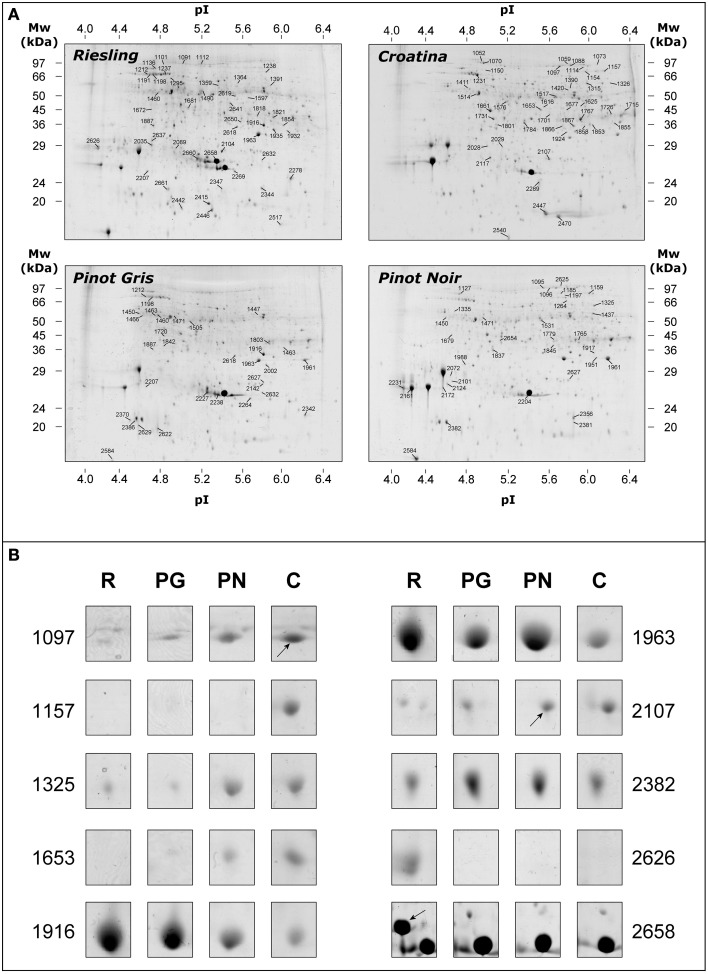
**Representative 2-DE maps of total protein fraction from grape berry exocarp of Riesling Italico, Pinot Gris, Pinot Noir, and Croatina**. **(A)** whole maps of the four experimental cultivars. **(B)** the image of 10 spots that resulted to be significantly different in abundance among the cultivars. Proteins (500 μg) were separated at pH 4–7, followed by 12.5% SDS-PAGE and visualized by cCBB-staining.

The proteomic analysis was achieved comparing 2-DE gels relating to exocarp of the four cultivars harvested in 2005 and 2006 vintages (*n* = 12). The number of the detected spots were comparable in the four samples and resulted to be of about 1300 spots per gels. Figure 2A shows the representative 2-DE maps of total protein fraction from exocarp berries of R, PG, PN, and C. Although the gel analysis pointed out a similar pattern, some differences in spot abundance were detected among the four cultivars. After automatic matching and filtering, the correspondence among the spots was assessed by manual checking. We thus focused our attention on the resulting 732 matches. In Figure 2B, some spots that resulted to be present with different abundance in the analyzed genotypes were reported.

**Figure 3:**
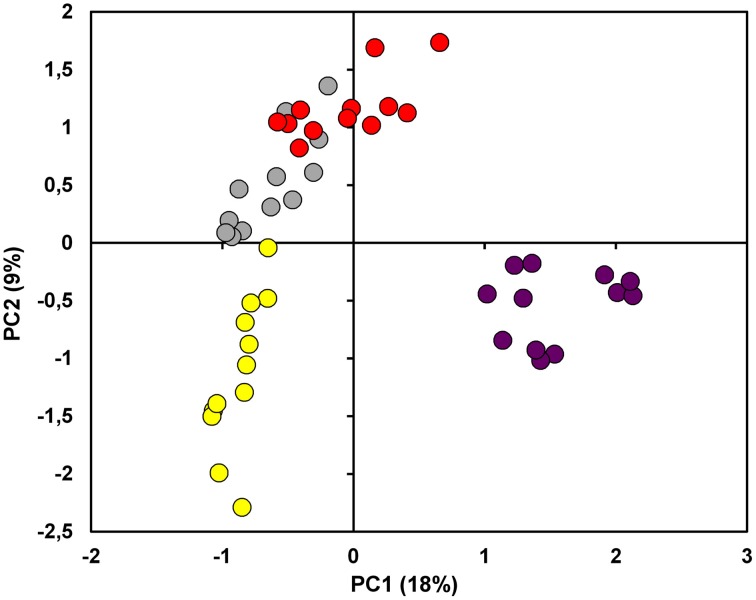
**Principal Component Analysis (PCA)**. The score plot showed in the figure was performed on the overall dataset considering the first two PCs. The samples under investigation are coded by a twelve-circle symbol colored in yellow, gray, red and violet for Riesling Italico, Pinot Gris, Pinot noir, and Croatina, respectively.

Through PCA it was possible to provide a first description of the relationship among the proteomes. PC1 accounted for the 18% of explained variance and showed a tendency to move apart the different cultivars. PC2 (9% of explained variance), on the other hand, put PG and PN at positive values while R and C were placed in the 3rd and 4th quadrant, respectively (Figure 3 and Supplementary Table S1). Overall, the score plot of the PCA clearly isolated C from the other 3 cultivars, while it showed the great affinity between PG and PN.

In order to distinguish the cultivars and to isolate the spots that were responsible of the observed differences, a linear discriminant analysis (LDA) was applied on the first 10 PCs calculated. The analysis was conducted by means of the forward stepwise (FS) algorithm (Fto_Enter = 13) that selected only 3 PCs (PC1, PC2, PC3) for the classification.

The evaluation of matrix classification showed that the 12 gels of each cultivar were correctly attributed to the class of their belonging (Table 1). Principal components and spot volumes are linked by a linear combination: this evidence permits to calculate each discrimination model according to the spots present on the gels. After the calculation of the 4 discrimination models, it was possible to order the spots in terms of their coefficients that is according of the weight by which they contribute in classifying the samples (Table 2). For each of the four cultivars the 30 most variables showing largest positive and negative coefficients were considered for identification. Since some of the selected spots overlapped among the four discrimination models the potential number of analyzed spots (*n* = 240) decrease to 144.

**Table 1:** **Classification matrix of Forward Stepwise—Linear Discriminant Analysis (FS-LDA)**.

	**Percent**	**Riesling**	**Pinot gris**	**Pinot noir**	**Croatina**
Riesling	100.0	12	0	0	0
Pinot gris	100.0	0	12	0	0
Pinot noir	100.0	0	0	12	0
Croatina	100.0	0	0	0	12
Total	100.0	12	12	12	12

The analyses was performed on the principal components (PCs) calculated, allowing the discrimination of the classes of samples and sorting the variables according to their relevance.

**Table 2:** **Matrix of the classification functions of Forward Stepwise—Linear Discriminant Analysis (FS-LDA)**.

	**Riesling**	**Pinot gris**	**Pinot noir**	**Croatina**
“PC1”	−38,5467	−11,8858	4,32349	46,1091
“PC2”	−20,5383	5,1528	13,11723	2,2683
“PC3”	−19,0547	−1,0812	5,84695	14,2889
Constant	−37,2914	−6,1952	−9,12430	−38,4861

Squared Mahalanobis distances from group centroids were obtained from PC1, PC2, and PC3.

### Protein identification and functional distribution

One hundred spots were identified by LC-ESI-MS/MS with a high degree of confidence among the group of 144 selected from FS-LDA (Table 3 and Supplementary Table S3). The missed identifications referred both to spots present in lower abundance that were not pickable from the gels (14% of the spots) and to the excised spots for which the analysis gave results under the set level of significance (19% of analyzed spots). Some spots, such as ACO (spots 1095, 1096, 1097, and 1114), NADP-ME (spots 1315 and 1325), ENO (spots 1505, 1531, and 2619), ENO-1 (spots 1471 and 1517), IFL-5 (spots 1924, 1935, and 2618), MET, (spots 1154, 1157, and 1159), VHA-B2-X1 (spots 1411 and 1450), PPO (spots 1821 and 2386) and many spots classified in stress group (HSP70-2, Thaumatin-like protein, TLP, PRP-10, and PRP-4) were identified as the same protein, indicating the presence of different forms with peculiar pI and/or M_r_. Nevertheless, different isoforms of some proteins were found (i.e., ENO and IFR).

**Table 3:** **List of spots identified by LC-ESI-MS/MS**.

**Spot ID**	**Accession number**	**Species**	**Protein description**	**Abbreviation**	**M_r_^a^ / pI ^a^**	**M_r_^b^ / pI ^b^**	**Cov (%)^c^**
**PHOTOSYNTHESIS (FIGURE 5A)**							
1238	XP_002266494.2	*Vitis vinifera*	Transketolase, chloroplastic	TK	68.8 / 5.9	78.8/ 6.6	10
1988	XP_002274796.1	*Vitis vinifera*	Oxygen-evolving enhancer protein 1, chloroplastic	OEE-1	30.4 / 4.8	35.3 / 6.1	19
**C-COMPOUND/CARBOHYDRATE/ENERGY METABOLISM (FIGURE 5B)**							
1095	XP_002278138.1	*Vitis vinifera*	Aconitate hydratase, cytoplasmic	ACO	95.1 / 5.6	110.1 / 6.7	12
1096	XP_002278138.1	*Vitis vinifera*	Aconitate hydratase, cytoplasmic	ACO	95.1 / 5.7	110.1 / 6.7	17
1097	XP_002278138.1	*Vitis vinifera*	Aconitate hydratase, cytoplasmic	ACO	94.3 / 5.7	110.1 / 6.7	26
1114	XP_002263337.1	*Vitis vinifera*	Aconitate hydratase 1	ACO-1	91.5 / 5.9	98.2 / 6.0	8
1197	XP_002270157.1	*Vitis vinifera*	NADH dehydrogenase [ubiquinone] iron-sulfur protein1, mitochondrial	NDUFS-1	74.2 / 5.8	80.9 / 6.5	14
1315	P51615.1	*Vitis vinifera*	NADP-dependent malic enzyme (NADP-ME)	NADP-ME	62.2 / 6.0	65.2 / 6.1	18
1325	P51615.1	*Vitis vinifera*	NADP-dependent malic enzyme (NADP-ME)	NADP-ME	61.8 / 6.1	65.2 / 6.1	27
1326	XP_002284729.1	*Vitis vinifera*	Phosphoglucomutase, cytoplasmic	PGMc	61.8 / 6.2	63.6 / 5.8	15
1437	XP_002263180.1	*Vitis vinifera*	Dihydrolipoyl dehydrogenase, mitochondrial	DLD	55.0 / 6.1	52.9 / 6.7	29
1460	XP_002283951.1	*Vitis vinifera*	ATP synthase subunit beta, mitochondrial	ATPase-β	53.5 / 4.8	59.6 / 5.8	32
1471	XP_002283632.1	*Vitis vinifera*	Enolase 1	ENO-1	53.5 / 5.0	47.9 / 5.7	18
1505	XP_002267091.2	*Vitis vinifera*	Enolase	ENO	52.1 / 5.1	55.8 / 8.4	36
1517	XP_002283632.1	*Vitis vinifera*	Enolase 1	ENO-1	51.5 / 5.7	47.9 / 7.0	15
1531	XP_002267091.2	*Vitis vinifera*	Enolase	ENO	50.9 / 5.6	55.8 / 8.4	37
1576	XP_002280514.1	*Vitis vinifera*	ATP-citrate synthase alpha chain protein 2	ACLA-2	48.6 / 5.2	46.4 / 5.3	9
1715	XP_002278444.1	*Vitis vinifera*	Formate dehydrogenase, mitochondrial	FDH	40.1 / 6.3	42.0 / 6.9	6
1726	XP_002278444.1	*Vitis vinifera*	Formate dehydrogenase, mitochondrial	FDH	39.4 / 6.2	42.0 / 6.9	10
1767	XP_002263950.1	*Vitis vinifera*	Phosphoglycerate kinase, cytosolic	PGK	36.6 / 5.9	42.4 / 6.3	52
1779	XP_002283381.1	*Vitis vinifera*	Fructose-bisphosphate aldolase cytoplasmic isozyme	FbPA	37.2 / 5.9	38.6 / 8.0	7
1951	XP_002277446.1	*Vitis vinifera*	Glucan endo-1,3-beta-glucosidase	Glu-β-EnGluco	31.6 / 6.1	36.7 / 8.4	25
1961	XP_002277446.1	*Vitis vinifera*	Glucan endo-1,3-beta-glucosidase	Glu-β-EnGluco	31.2 / 6.2	36.7 / 8.4	45
1963	XP_002277446.1	*Vitis vinifera*	Glucan endo-1,3-beta-glucosidase	Glu-β-EnGluco	31.2 / 5.8	36.7 / 8.4	25
2619	XP_002267091.2	*Vitis vinifera*	Enolase	ENO	50.5 / 5.6	55.8 / 5.4	28
**CELL WALL (FIGURE 5C)**							
1765	XP_002263490.1	*Vitis vinifera*	UDP-arabinopyranose mutase 1	UDP-Arab-M1	36.5 / 5.7	40.8/ 6.2	12
2002	BAB78506.1	*Vitis labrusca x Vitis vinifera*	Xyloglucan endo-transglycosylase	XTHs	30.1 / 5.9	32.7 / 5.5	7
**NITROGEN METABOLISM/AMINO ACID METABOLISM (FIGURE 5D)**							
1154	XP_002276438.1	*Vitis vinifera*	5-methyltetrahydropteroyltriglutamate–homocysteine methyltransferase	MET	80.8 / 6.0	85.0/ 6.1	9
1157	XP_002276438.1	*Vitis vinifera*	5-methyltetrahydropteroyltriglutamate–homocysteine methyltransferase	MET	79.8 / 6.1	85.0/ 6.1	21
1159	XP_002276438.1	*Vitis vinifera*	5-methyltetrahydropteroyltriglutamate–homocysteine methyltransferase	MET	80.1 / 6.0	85.0 / 6.1	12
1625	XP_003635049.1	*Vitis vinifera*	Cysteine desulfurase 1, mitochondrial-like	CyD-1	46.2 / 5.9	49.9 / 6.7	17
1720	CAC39216.1	*Vitis vinifera*	Glutamine synthetase	GS	39.8 / 4.9	39.0 / 5.4	13
1731	CAC39216.1	*Vitis vinifera*	Glutamine synthetase	GS	38.6 /5.1	39.0 / 5.4	34
**SECONDARY METABOLISM (FIGURE 5E)**							
1616	BAB41020.1	*Vitis vinifera*	UDP-glucose:flavonoid 3-O-glucosyltransferase	UFGT	46.7 / 5.6	50.1 / 6.2	6
1653	ABM67590.1	*Vitis vinifera*	Anthocyanidin synthase	ANS	45.0 / 5.5	40.2 / 5.6	28
1818	2P3X	*Vitis vinifera*	Polyphenol oxidase	PPO	35.2 / 5.8	38.4 / 5.5^e^	5
1821	AAB41022.1	*Vitis vinifera*	Polyphenol oxidase	PPO	35.2 / 5.9	67.4 / 6.4	7
1916	CAI56335.1	*Vitis vinifera*	Isoflavone reductase-like protein 6	IFL-6	32.7 / 5.9	33.9 / 6.0	43
1924	CAI56334.1	*Vitis vinifera*	Isoflavone reductase-like protein 5	IFL-5	32.4 / 5.8	33.9 / 5.8	35
1935	CAI56334.1	*Vitis vinifera*	Isoflavone reductase-like protein 5	IFL-5	32.0 / 6.0	33.9 / 5.8	24
2117	P51117.1	*Vitis vinifera*	Chalcone-flavonone isomerase 1	CHI-1	27.0 / 5.0	25.1 / 5.3	22
2382	S52629	*Vitis vinifera*	Catechol oxidase (EC 1.10.3.1) precursor - grape	Cat-OX	21.8 / 4.7	67.3 / 6.3	9
2386	AAB41022.1	*Vitis vinifera*	Polyphenol oxidase	PPO	21.7 / 4.6	67.4 / 6.4	7
2618	CAI56334.1	*Vitis vinifera*	Isoflavone reductase-like protein 5	IFL-5	33.1 / 5.6	33.9 / 5.8	29
**HORMONE METABOLISM (FIGURE 5F)**							
1391	AAX48772	*Vitis vinifera*	9,10[9',10']carotenoid cleavage dioxygenase	CCD	58.1 / 5.9	61.1 / 6.0	9
1853	CAN68994.1	*Vitis vinifera*	Aldo-keto reductase 4^d^ *(auxin regulated)*	Ald-Keto-Red-4	34.5 / 6.0	37.5 / 6.5	7
1854	CAN68994.1	*Vitis vinifera*	Aldo-keto reductase 4^d^ *(auxin regulated)*	Ald-Keto-Red-4	34.5 / 6.1	37.5 / 6.5	7
1855	CAN68994.1	*Vitis vinifera*	Aldo-keto reductase 4^d^ *(auxin regulated)*	Ald-Keto-Red-4	34.5 / 6.2	37.5 / 6.1	6
2227	XP_002280658.1	*Vitis vinifera*	Stem-specific protein TSJT1 *(auxin regulated)*	TSJT-1	25.6 / 5.3	25.2 / 5.7	22
2029	XP_002283483.1	*Vitis vinifera*	Stem-specific protein TSJT1 *(auxin regulated)*	TSJT-1	28.9 / 5.1	27.2 / 5.5	22
**REDOX (FIGURE 5G)**							
1784	XP_002282603.1	*Vitis vinifera*	Probable protein disulfide-isomerase A6	PDI-A6-1	36.1 / 5.4	39.3 / 5.6	23
2107	XP_002284767.1	*Vitis vinifera*	L-ascorbate peroxidase 2, cytosolicc	AscPOX-2	27.2 / 5.6	27.6 / 5.7	54
2269	AKG51696.1	*Vitis vinifera*	Superoxide dismutase	SOD	25.0 / 5.6	25.3 / 6.8	17
**PROTEIN (FIGURE 5H)**							
1070	XP_002282146.1	*Vitis vinifera*	Cell division control protein 48 homolog	CDC48	100.2 / 5.0	89.6 / 5.1	24
1088	XP_002266780.1	*Vitis vinifera*	elongation factor 2	EF-2	95.6 / 5.8	94.0 / 5.8	9
1150	XP_002273246.1	*Vitis vinifera*	Probable Xaa-Pro aminopeptidase P	AMPP	82.0 / 5.0	71.7 / 5.2	19
1390	XP_002283510.1	*Vitis vinifera*	T-complex protein 1 subunit zeta	TCP-1	58.0 / 5.8	59.1 / 6.0	20
1447	XP_002284370.1	*Vitis vinifera*	Probable mitochondrial-processing peptidase subunit beta	MPP-β	54.9 / 5.8	58.5 / 6.4	13
1463	XP_002283310.1	*Vitis vinifera*	Mitochondrial-processing peptidase subunit alpha	MPP-α	53.7 / 4.8	54.5 / 5.7	14%
1490	XP_002276114.1	*Vitis vinifera*	Leucine aminopeptidase 1	LAP-1	52.8 / 5.2	60.7 / 6.7	11
1597	XP_002283140.2	*Vitis vinifera*	Aminoacylase-1 isoform X1	AmCyl-X1	47.6 / 5.7	54.1 / 5.6	5
1679	XP_002276099.1	*Vitis vinifera*	DNA damage-inducible protein 1	DDI-1	43.1 / 4.7	45.1 / 5.0	11
1803	XP_002278975.1	*Vitis vinifera*	26S proteasome non-ATPase regulatory subunit 7 homolog A	PSMD-7	35.6 / 5.9	34.8 / 6.0	25
1917	XP_002284566.1	*Vitis vinifera*	26S proteasome non-ATPase regulatory subunit 14	PSMD-14	32.7 / 6.1	34.5/ 6.3	11
**CELL ORGANIZATION/SIGNAL (FIGURE 5I)**							
1514	XP_002285721.1	*Vitis vinifera*	Tubulin alpha-3 chain	TUB-3	51.5 / 4.9	49.7 / 4.9	15
1661	XP_002282516.1	*Vitis vinifera*	Actin-7	ACT-7	44.0 / 5.0	41.7 / 5.3	37
**TRANSPORT (FIGURE 5L)**							
1411	XP_010664138.1	*Vitis vinifera*	V-type proton ATPase subunit B 2 isoform X1	VHA-B2-X1	56.0 / 4.8	56.2 / 5.0	6
1450	XP_010664138.1	*Vitis vinifera*	V-type proton ATPase subunit B 2 isoform X1	VHA-B2-X1	54.5 / 4.6	56.2 / 5.0	8
2627	XP_002270168.1	*Vitis vinifera*	V-type proton ATPase subunit E1	VHA-E1	27.0 / 5.9	26.0 / 6.2	6
**OTHER FUNCTIONS (FIGURE 5M)**							
1677	XP_010649306.1	*Vitis vinifera*	Protein YLS2-like	YLS2	43.3 / 5.8	42.0 / 5.9	9
1801	XP_002284459.1	*Vitis vinifera*	11S globulin subunit beta	11S-Globulin	35.6 / 5.1	38.3 / 5.4	16
1866	XP_002270155.1	*Vitis vinifera*	Glutelin type-A 3	Glutelin-A3	34.3 / 5.7	38.5 / 5.6	9
1867	XP_002270155.1	*Vitis vinifera*	Glutelin type-A 3	Glutelin-A3	34.2 / 5.8	38.5 / 5.6	21
1887	ABC86739.1	*Vitis pseudoreticulata*	Cyclase	Cyclase	33.5 / 4.8	29.8 / 5.5	16
2028	XP_002267609.1	*Vitis vinifera*	Remorin	Remorin	28.9 / 5.0	21.8 / 5.3	14
2104	XP_010654260.1	*Vitis vinifera*	Uncharacterized protein At5g02240	Unc-1	27.2 / 5.4	36.1 / 9.4	15
2264	XP_002283286.1	*Vitis vinifera*	NAD(P)H dehydrogenase (quinone) FQR1	FQR-1	25.0 / 5.7	21.7 / 5.8	46
2289	NP_001268052.1	*Vitis vinifera*	Ripening-related protein grip22 precursor	Grip22	24.1 / 5.5	22.9 / 4.8	6
2517	XP_002271352.1	*Vitis vinifera*	Nucleoside diphosphate kinase B	NdPK-B	16.6 / 6.0	16.3 / 6.8	32
2637	NP_001268052.1	*Vitis vinifera*	Ripening-related protein grip22 precursor	Grip22	29.7 / 4.7	22.9 / 4.8	17
**STRESS (FIGURE 5N)**							
1136	XP_002273244.1	*Vitis vinifera*	Heat shock cognate protein 80-like	HSP80	84.5 / 4.8	80.8 / 5.0	24
1198	XP_002283532.2	*Vitis vinifera*	Heat shock cognate 70 kDa protein 2	HSP70-2	72.9 / 4.8	71.2 / 5.2	28
1231	XP_002283532.2	*Vitis vinifera*	Heat shock cognate 70 kDa protein 2	HSP70-2	66.9 / 4.9	71.2 / 5.2	45
1237	XP_002283532.2	*Vitis vinifera*	Heat shock cognate 70 kDa protein 2	HSP70-2	67.5 / 4.9	71.2 / 5.2	28
2124	XP_010657212.1	*Vitis vinifera*	Carboxymethylenebutenolidase homolog	Carboxy-Ase	27.0 / 4.7	26.3 / 5.0	20
2161	4L5H_A	*Vitis vinifera*	Thaumatin-like protein	TLP	26.5 / 4.5	21.3 / 4.8	14
2172	4L5H_A	*Vitis vinifera*	Thaumatin-like protein	TLP	26.4 / 4.6	21.3 / 4.8	28
2204	AAZ93634.1	*Vitis vinifera*	MSA	MSA	25.7 / 5.4	16.7 / 5.7	14
2231	CAB85637.1	*Vitis vinifera*	Putative thaumatin-like protein	TLP	25.7 / 4.2	24.0 / 4.9	9
2278	ABB02395.1	*Vitis vinifera*	Temperature-induced lipocalin	TInLi	24.6 / 6.1	21.5 / 6.6	13
2342	XP_002281506.1	*Vitis vinifera*	Class I heat shock protein	HSP-I	22.8 / 6.2	16.3/ 6.9	18
2344	XP_010657906.1	*Vitis vinifera*	22.0 kDa class IV heat shock protein-like	HSP-IV	22.8 / 5.8	21.3/ 5.9	30
2356	CAC16165.1	*Vitis vinifera*	Pathogenesis-related protein 10	PRP-10	22.5 / 5.9	17.1 / 6.0	23
2381	CAC16165.1	*Vitis vinifera*	Pathogenesis-related protein 10	PRP-10	22.0 / 5.9	17.1 / 6.0	23
2415	XP_002281285.1	*Vitis vinifera*	18.2 kDa class I heat shock protein	HSP-I	19.9 / 5.3	17.1 / 5.8	9
2446	XP_003631809.1	*Vitis vinifera*	17.3 kDa class II heat shock protein-like	HSP-s	18.7 / 5.3	17.5 / 5.7	27
2540	ADG35965.1	*Vitis hybrid cultivar*	Pathogenesis-related protein 4	PRP-4	15.9 / 5.2	15.2 / 5.5	14
2584	ADG35965.1	*Vitis hybrid cultivar*	Pathogenesis-related protein 4	PRP-4	14.7 / 4.4	15.2 / 5.5	24
2626	AAB65776.1	*Vitis vinifera*	Class IV endochitinase	EnChi-4	29.0 / 4.2	27.2 / 5.4	32
2658	AAZ93634.1	*Vitis vinifera*	MSA	MSA	26.7 / 5.4	16.7 / 5.7	31

Proteins were divided according to their functions.

a Experimental molecular weight (kDa) or isoelectric point.

b Theoretical molecular weight (kDa) or isoelectric point.

c Amino acid coverage (%).

d Information obtained by blastp (protein-protein BLAST) algorithm.

e Partial sequence.

**Figure 4:**
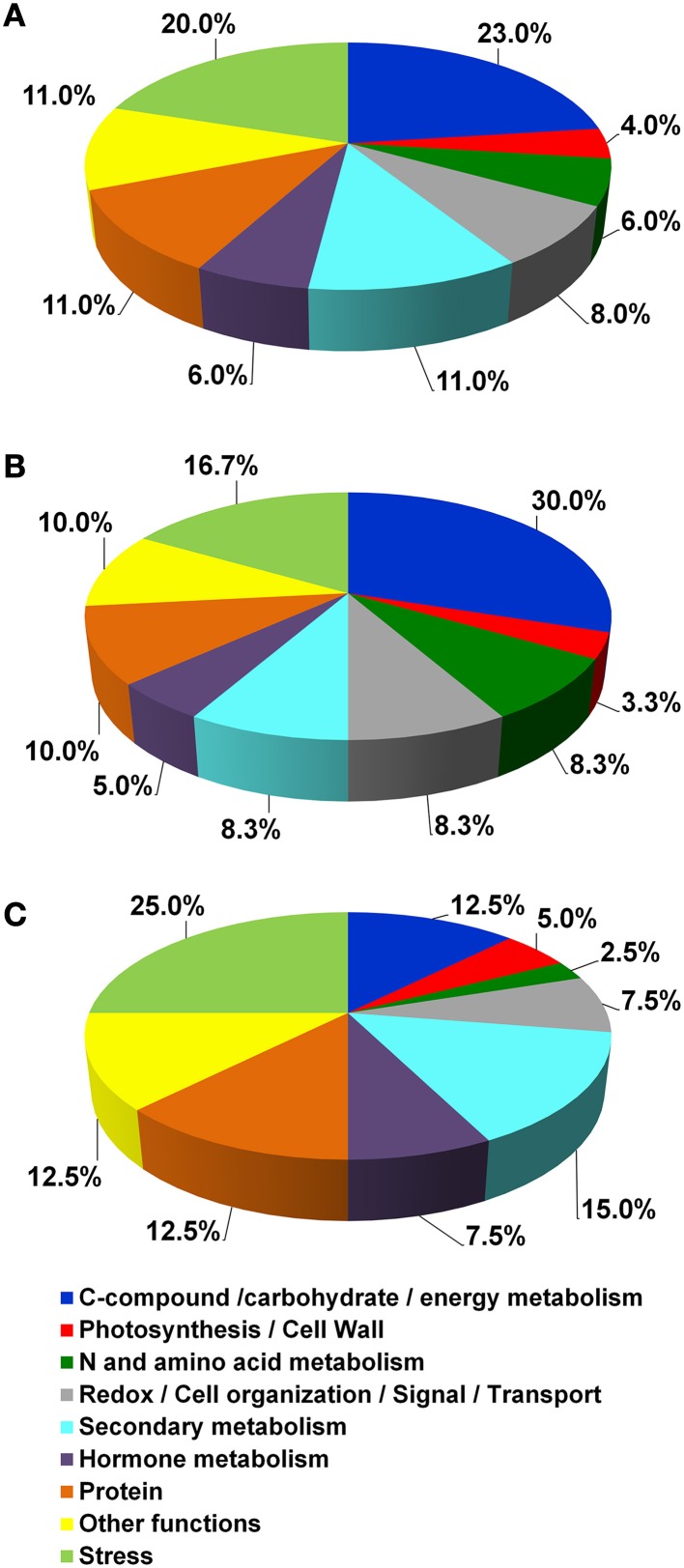
**Functional distribution of the identified proteins**. **(A)** functional distribution of all the identified proteins reported in Table 3. **(B,C)** functional distribution of proteins that showed greater abundance in Croatina and in Riesling Italico, respectively.

According to their function, the identified proteins were grouped in 12 main classes (Table 3 and Figure 4A). In detail, they were involved in Photosynthesis/Cell Wall (4%), C-compound/carbohydrate/energy metabolism (23%), N and amino acid metabolism (6%), Redox/Cell organization/Signal/Transport (8%), Secondary metabolism (11%), Hormone metabolism (6%), Protein (11%), Other functions (11%) and Stress (20%).

The quantitative differences among the four genotypes are showed in the Supplementary Figure S1, in which spot volume percentages were reported for all identified proteins. As a whole, the results showed that the greater differences occurred between R and C genotypes. In this view, we created two functional distribution pie charts in which R and C genotypes were compared. Figures 4B,C showed the proteins having higher abundance in C and in R, respectively. It is interesting to observe that in C the proteins with higher abundance belonged to the C-compound/carbohydrate/energy metabolism, N and amino acid metabolism functional classes, while in R Secondary metabolism and Stress ones prevailed.

**Figure 5:**
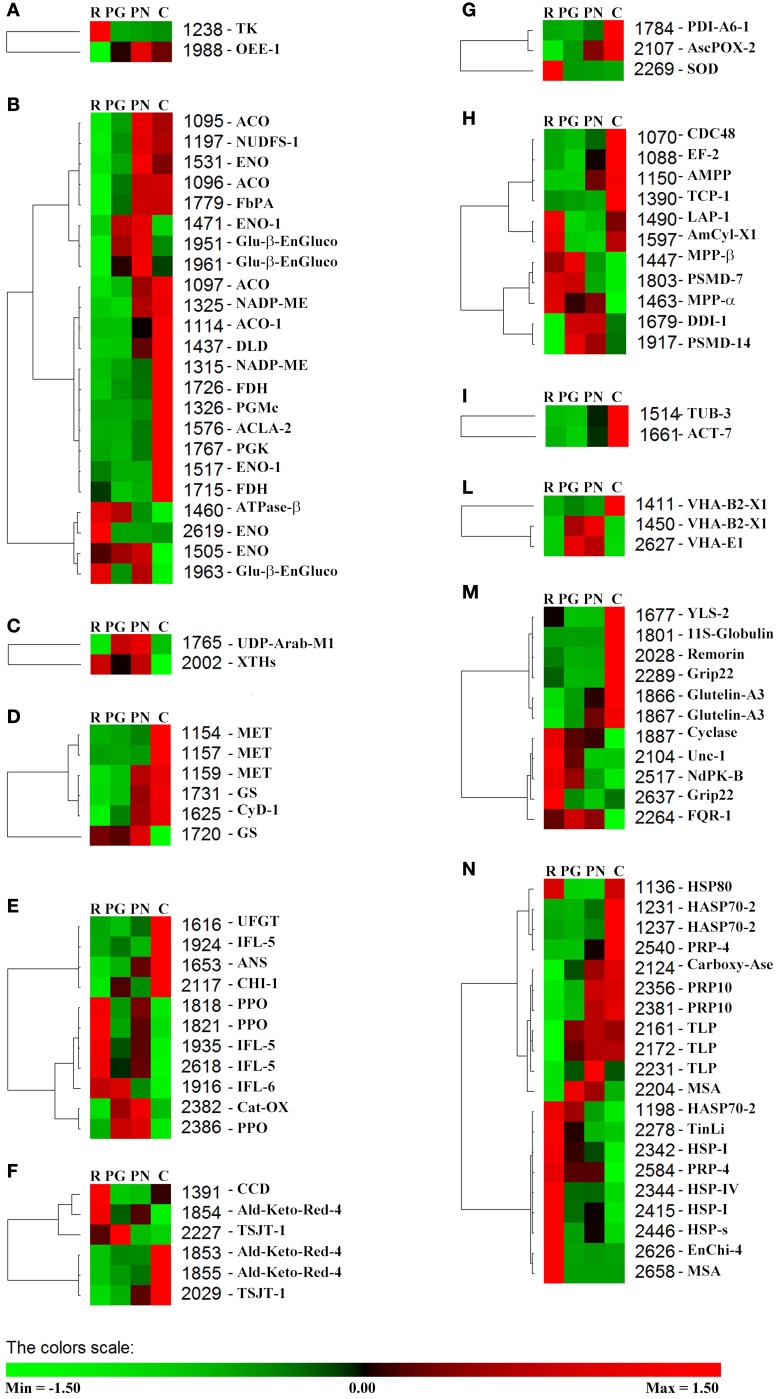
**Clustering analysis of the spots grouped according to their functional class**. Two-way hierarchical clustering analysis of proteins that resulted to be significantly different in their relative spot volumes and identified by LC-ESI-MS/MS (Table 3) was performed with PermutMatrix. Pearson's distance and Ward's algorithm were used for the analysis. Each colored cell represents the average of the relative spot value, according to the color scale at the bottom of the figure. **(A)** Phothosynthesis. **(B)** C-compound/carbohydrate/energy metabolism. **(C)** Cell wall. **(D)** Nitrogen metabolism and amino acid metabolism. **(E)** Secondary metabolism. **(F)** Hormone metabolism. **(G)** Redox. **(H)** Protein. **(I)** Cell organization/Signal. **(L)** Transport. **(M)** Other functions. **(N)** Stress.

Using PermutMatrix software, a hierarchical clustering of the different functional groups was created to depict in detail the differences in protein abundances as well as to appreciate the gap among the proteomes of the four cultivars (Figure 5). The most striking evidence was that many proteins showed a direct or inverse relation with anthocyanin content. R and C showed the most divergent proteomes, while PG and PN looked more similar.

### Metabolite analysis

GC-MS analysis permitted the identification and quantification of 56 metabolites (Supplementary Table S2). The choice of performing these analyses through a SIM approach was linked to the fact that, especially in a ripe fruit, the metabolome is dominated by large amount of accumulated sugars. Because of this, the peaks of ions relating to primary metabolites could be reduced or not easily integrated. In order to solve these analytical problems, we thus decided to limit the acquisition to the ions of interest, improving the signal and the quality of ion shape (data not shown). The ions used for the quantification were chosen on the basis of the spectra and the retention times obtained by the isolated injection of standard substances under the same chromatographic and MS conditions. Among the quantified metabolites, there were sugars (16), amino acids (15), organic acids (13), and phenolic acids (5). Moreover, among the identified compounds, 5 were glycolytic intermediates and 7 were intermediates of TCA cycle. Sixteen metabolites resulted significantly different to ANOVA test (*p*≤0.05) and some of these showed a trend that was related with the anthocyanin one (Supplementary Figure S2).

## Discussion

### The multivariate statistical analysis permitted to isolate spots useful to discriminate the four cultivar

Through multivariate analyses it was possible to overview the relationship among the proteomes of the 4 cultivars, clearly distinguishing the samples according to the cultivar and sorting the most relevant observed differences in spot abundance.

As expected, the biological dataset resulted to be quite complex since the first 5 PCs accounted only the 43.37% of the explained variance (Supplementary Table S1). In every case, it is interesting to note that in PC1 the cultivars were roughly ordered following their anthocyanin content, moving from R at negative values to C proteomes at positive values. Nevertheless, the placement on PC2, in which there was substantial distinction between Pinot and non-Pinot cultivars, suggested a contribution of genetic factors (Figure 3). At the same time, it was surprising that on the first 2 PCs differences linked to seasonal variation did not emerge. In this view, it must be considered that the technique used in this study permitted to consider only a part of whole proteome and this could be inadequate to find the plasticity of the grapevine berry previously revealed by transcriptomic study (Dal Santo et al., 2013).

The relation between the four proteomes was further investigated through FS-LDA performed on the first 10 PCs calculated. The trick of using PCs instead original variables led to dimensionality reduction and to noise elimination by excluding the less significant ones as performed in Negri et al. (2011). The linear discriminant analysis reflected and reinforced PCA results since, as witnessed by the classification matrix (Table 1), it correctly distinguished the gels according to the cultivar of belonging. Moreover, the squared Mahalanobis distances from group centroids (Table 2) demonstrated that the objects (i.e., the gels) could be ordered following the anthocyanin content of the 4 cultivars: R gels, for instance, are thus really distant from the centroid of C and rather close to the PG one. At the same time, it was interesting to note that although they are quite identical at the genetic level and overlapped in PCA score plot (Figure 3), the gels of the two Pinot cultivars were clearly separated by FS-LDA, showing a clear proximity as inferable by the small values of the relative squared Mahalanobis distances (Table 2).

As described, an interesting number of the variables with the highest or the lowest coefficients of discrimination showed to have a high significance in the models of 3 or even all the 4 cultivars, suggesting that some of the main factors that differentiate them, as for anthocyanin content, could pass through the modulation of some specific proteins.

### Proteomic and metabolomic data highlight new peculiar traits of the metabolism operating in grape berry exocarp

Previous proteomic studies revealed that, differently to the mesocarp, in the exocarp tissue of grape berry the glycolysis and the hexose-monophosphate shunt pathways remained active also at full ripe stage (Sarry et al., 2004; Negri et al., 2008b, 2011). This metabolic state was attributed to the demand of C-skeletons and energy to sustain the synthesis of secondary compounds (mainly anthocyanins) and/or other activities linked to defensive mechanisms.

Focusing the attention on primary carbon metabolism (i.e., glycolysis and TCA cycle) and respiration chain, the accumulation trend of the spots referring to PGMc (spot 1326), PGK (spot 1767), ENO-1 and ENO (spot 1517 and 1531), ACLA-2 (spot 1576), DLD (spot 1437), and NDUFS-1 (spot 1197) showed a good association with anthocyanin content also in this study (Figures 1, 2, 5; Table 3; Supplementary Figure S1). According to a greater activation of glycolysis and TCA cycle, some intermediates of these pathways, such as fructose-1,6-phosphate, pyruvate and succinate, showed the same tendency (Supplementary Figure S2).

The greater activation of the carbon metabolism was also suggested by a coherent increase in the red cultivar of aconitate hydratase, an enzyme having a central role in citric acid metabolism (Terol et al., 2010). Three spots identified as ACO showed high similarity with the same accession (XP_002278138.1), so suggesting that the spots could refer to different phosphorylated forms, according to the PTMs previously identified for this enzyme (Bycova et al., 2003; Millar et al., 2005).

The results regarding enolase highlighted the multifunctional role of these glycolytic enzymes. Of the five spots referring to enolase, three revealed to be the same protein (accession XP_002267091.2), while the others shared the greatest similarity with the accessions XP_002283632.1 suggesting the presence of different isoforms (Table 3). Although, the current knowledge on the pattern of *Vitis vinifera* enolase did not permit further consideration from the classification point of view, by using iPSORT software (http://ipsort.hgc.jp/index.html) we verified the absence of N-terminal signal peptide, suggesting that all identified forms are cytosolic enolase. These data supported the suggestion that also in grape berry exocarp, as previously observed in others plant tissues (Voll et al., 2009), the plastidic isoform could be lacking. As recently emerged, in the absence of a complete glycolysis pathway in the plastids, cytosolic enolase plays a central role to modulate the synthesis of aromatic amino acids and secondary phenyilpropanoid compounds (Voll et al., 2009; Eremina et al., 2015). Moreover, the trend of spots 1517 and 1531 could suggest that they are the forms that modulate shikimate pathway.

On the base of the above conclusion that cytosolic phosphoenolpyruvate is requested for both phenylpropanoid biosynthesis and respiratory pathway, the higher level of pyruvate in red cultivars could appear unexpected (Supplementary Figure S2). In this context, it could be observed that among the spots showing an increase in abundance in red cultivars, two were identified as a NADP-dependent malic enzyme (NADP-ME, spots 1315 and 1325), supporting the idea that this enzyme in the skin tissue could play an important role to sustain pyruvate request. Nevertheless, the level of malic acid was higher in the red cultivars (Supplementary Figure S2), suggesting that a concomitant supply of this organic acid occurred. Previously, Iland and Coombe (1988) showed that during ripening the levels of this organic acid decreased in the mesocarp, whilst it did not change in the exocarp. Moreover, these authors found that the leaching of malate from the mesocarp tissue increased during ripening. Although a direct evidence is not available, an interesting hypothesis is that during ripening malic acid could move from mesocarp to skin, sustaining the carbon demand occurring in this tissue.

Two spots identified as a mitochondrial formate dehydrogenase (spots 1715 and 1726) showed greater abundance in C. Their reciprocal position on the gel appeared ascribable to PTMs, such as a phosphorylation (Bycova et al., 2003; Millar et al., 2005). Although a complete understanding of the functional role of this enzyme is awaited, its accumulation could be interpreted as a further request of reducing power (Plaxton and Podestà, 2015) that occurs in the cultivar with the highest level of anthocyanins.

Previously, a role of glycolytic enzymes, such as aldolase and enolase in the activation of vacuolar H^+^-ATPase through an association with a subunit VHA-B has been described (Barkla et al., 2009 and references therein). Moreover, an activation of vacuolar proton pumps during grape berry was reported (Terrier et al., 2001; Grimplet et al., 2009; He et al., 2010). In red cultivars an upsurge of both the FbPA (spots 1779) and the subunit VHA-B2-X1 (spots 1411) took place (Figures 5B,L). Although the anthocyanin transport into vacuole in exocarp tissue involves different mechanisms (Coon et al., 2008; Gomez et al., 2009; Sweetman et al., 2009; Francisco et al., 2013), these results suggest that on the whole the transport of these compounds could require an increase of the tonoplastic H^+^-pump activity.

Some identified spots, such as CHI-1, ANS and UFGT (spots 2117, 1653, and 1616, respectively), were enzymes operating in the anthocyanin synthesis pathway. Their trends were well related to the anthocyanin levels (Figures 1, 5E). These results appear in accordance to previous transcriptional analysis in which the expression of the genes codifying for these proteins were mainly (CHI-1, ANS) or only (UFGT) detected in the red cultivars (Boss et al., 1996b). In this context, the greater abundance in PN or C of two spots that were identified as glutamine synthetase (Spots 1731, 1720) was in agreement with interlinks between anthocyanin metabolism and nitrogen recycling (Singh et al., 1998; Cantón et al., 2005). Moreover, we found that the abundances of three spots corresponding to methionine synthase (spots 1154, 1157, and 1159) related with the anthocyanin contents (Figure 5D). Nevertheless the levels of methionine were not different among the four cultivars, suggesting that a simultaneous transformation of this amino acid occurred in red cultivars (Supplementary Figure S2). Although a more direct evidence is needed, an intriguing hypothesis is that this amino acid could be required for the biosynthesis of ethylene, according to an involvement of this hormone in the anthocyanin production (El-Kereamy et al., 2003; Böttcher and Davies, 2012).

From the proteomic analysis differences in protein metabolism emerged (Figure 5H). In C the Elongation factor 2-like isoform 1 (EF-2, spot 1088), a probable Xaa-Pro aminopeptidase P (AMPP, spot 1150) and the subunit zeta T-Complex protein 1 (TCP-1, spot 1390) resulted more abundant, while some proteins involved in mitochondrial-proteolytic system (MPP-α and MPP-β, spots 1463 and 1447) or in protein degradation (PSMD-7 and PMSD-14, spots 1803 and 1917, respectively) were inversely related to the anthocyanin content. Taken together, these results pointed out that in C prevailed activities involved in protein synthesis, whilst in the other cultivars a more evident protein catabolism seemed to take place. In this view, it is interesting to observe that in C CDC48, TUB-3 and ACT-7 (spots 1070, 1514, and 1661, respectively) resulted most abundant, so sustaining that in this cultivar was operating a more active cellular metabolism (Figures 5H,I).

### Many of the identified proteins are involved in the stress-related processes

This work brought to the identification of many proteins that are known to be involved in defense and stress responses. It is interesting to observed that many studies performed on grape berry reported the presence of proteins, such as thaumatin-like and chitinases, even in the absence of pathogen infections (Deytieux et al., 2007; da Silva et al., 2005; Negri et al., 2008b, 2011; Fraige et al., 2015), suggesting that their accumulation could be linked to a preventive defensive plan closely related to the genetic background of the cultivar. Nevertheless, it was suggested that these proteins could also play a multifaceted role in the ripening process (van Hengel et al., 2001, 2002; Kasprzewska, 2003). In our work we find the presence of proteins belonging to the stress class also in healthy fruit as well as we confirm that they resulted to be linked to the genetic background (Figure 5N and Table 3).

Previously, Pilati et al. (2007) described in the red cultivar PN that the start of ripening phase was characterized by an oxidative burst and that this depended on specific changes in gene expression. Recently, these authors found that this process occurs mainly in the skin tissue and reported evidences on possible role of ROS as cellular signal (Pilati et al., 2014). In our work the majority of the proteins classified in the stress group showed an abundance that was both directly or inversely related to the anthocyanin contents. Considering their functions, we found a similar oxidative stress condition, but the data suggested that the analysed cultivars used different strategies in responding to it. This was particular evident comparing C with R. In this last cultivar some proteins that are known to be involved in the oxidative stress responses (i.e., PPO and SOD, spots 1818, 1821, and 2269 respectively) were present in higher abundance (Figures 5E,G). Considering the Mr it was possible to conclude that these PPO spots corresponded to the active form of the enzyme (Dry and Robinson, 1994). According to the possibility that a suffering condition was occurring in the R, five HSPs were more accumulated (spots 1198, 2342, 2344, 2415, 2446). Moreover, the level of ascorbic acid resulted significantly lower in R respect to other cultivars (Supplementary Figure S2). This result could be linked to genetic characteristic as well as to be a symptom of a suffering status.

It is interesting to observe that in this cultivar a 9,10[9′,10′]carotenoid cleavage dioxygenase (CCD, spot 1391) was found to be present in the highest amount, an enzyme involved in the carotenoid cleavage to produce apocarotenoid from which important signaling molecules, such as abscisic acid (ABA) and stringolactones derive (Harrison and Bugg, 2014). Recently, Ye et al. (2011) reported interesting evidences on possible role of ABA in the control of catalase gene expression, an enzyme that was described to play a central role in the ROS detoxification also in grape berry skin (Pilati et al., 2014). Although the pH range used in IEF excluded the possibility to detect the catalase, taken together, also these results further sustained the idea that in the white cultivar R biochemical mechanisms to counteract an oxidative stress condition were activated.

This study led to the identification of some forms of isoflavone reductase-like (IFL) protein (spots, 1916, 1924, 1935, and 2618). Three of these (1924, 1935, and 2618) showed high similarity with the same accession (CAI56334.1). On the basis of experimental pI, it could be suggested that the three spots refer to different phosphorylation state, a PTM that is not yet described for this enzyme. Since the stereospecific reduction of isoflavones by isoflavone reductase (IFR) is restricted primarily to legumes, a distinct reductase reaction was postulated for IFL. In fact, this enzyme was related to oxidative stress occurring during the somatic embryogenesis of *Vitis vinifera* callus (Zhang et al., 2009). Considering that three IFL spots showed to be more abundant in R, our data suggested a similar protective role in grape berry skin.

Differently from the white cultivar R, in the red cultivars many of these responses resulted less active. In this context, it could be underlined that flavonoid biosynthesis produces some compounds, such as quercetin, myrecetin, kaempferol, having high antioxidant activity (Pietta, 2000). The metabolic profiling on berry skin performed on a large number of cultivars, revealed that these compounds are higher in PN and C than in R (Mattivi et al., 2006). Hence, our work revealed that the oxidative burst occurring in grape berry skin ripening stimulates typical antioxidant responses in which the phenolic compounds have a central role. In the red cultivars, in fact, the high levels of phenolic compounds probably compensate for the induction of the other antioxidant mechanisms observed in the white cultivar.

## Concluding remarks

This work highlighted new information about the biochemical and physiological events occurring in the skin tissue during grape berry ripening. Considering the functional distribution of the identified proteins and the trends of some metabolites, the results showed how many physiological processes, such as carbon metabolism (e.g., glycolysis and TCA cycle), energy conversion, secondary metabolism and oxidative stress, are involved in the protective role against damage by physical injuries and pathogen attacks. Nevertheless, some biochemical responses appeared requested to counteract oxidative burst, an event that characterizes the ripening step of grape berry skin. In this view, the strategy used strictly depended on flavonoid biosynthesis.

## Author contributions

AN contributed to the conception of the experimental design, carried out protein extraction, 2-DE, gel analysis, metabolomic analysis and statistical analysis. BP contributed to the conception of the experimental design, carried out protein characterization by LC-ESI-MS/MS, analyzed the MS data. OF and AS participated to the manuscript revision. LE conceived the study, coordinated the experiments, wrote and edited the manuscript. All authors read and approved the final manuscript.

### Conflict of interest statement

The authors declare that the research was conducted in the absence of any commercial or financial relationships that could be construed as a potential conflict of interest.

## Abbreviations

2-DE: two-dimensional electrophoresis
ACN: acetonitrile
cCBB: colloidal Coomassie Brilliant Blue G-250
C: Croatina
FS-LDA: Forward Stepwise Linear Discriminant Analysis
PCA: Principal Component Analysis
PG: Pinot Gris
PN: Pinot Noir
R: Riesling Italico
SIM: Selected Ion Monitoring.
