# Supplementary material for: Proteomic and metabolic traits of grape exocarp to explain different anthocyanin concentrations of the cultivars
Source: Front Plant Sci. 2015 Aug 4;6:603. doi: 10.3389/fpls.2015.00603 (PMC4523781; doi:10.3389/fpls.2015.00603)
Supplement: Supplementary file 1 [file Table1.PDF]

Table S1. Principal Component Analysis (PCA). In the analyses the first ten principal components were considered. PCA was conducted on 48 x 732 matrix: 48 being the number of samples [six replicate gels x 2 years (n = 12) for each cultivars] and 732 being the spots matched among gels.

	Eigenvalue	% Total (variance)	Cumulative Eigenvalue	Cumulative (%)
**PC1**	129.5012	17.71562	129.5012	17.71562
**PC2**	68.7619	9.40656	198.2631	27.12218
**PC3**	52.1529	7.13445	250.416	34.25663
**PC4**	37.993	5.1974	288.409	39.45404
**PC5**	28.6032	3.91289	317.0122	43.36692
**PC6**	24.731	3.38317	341.7432	46.7501
**PC7**	23.3083	3.18854	365.0515	49.93864
**PC8**	21.9833	3.00729	387.0348	52.94593
**PC9**	20.4554	2.79828	407.4902	55.74421
**PC10**	18.3221	2.50645	425.8123	58.25066
