# Supplementary material for: Proteomic and metabolic traits of grape exocarp to explain different anthocyanin concentrations of the cultivars
Source: Front Plant Sci. 2015 Aug 4;6:603. doi: 10.3389/fpls.2015.00603 (PMC4523781; doi:10.3389/fpls.2015.00603)
Supplement: Supplementary file 2 [file Table2.PDF]

Table S2. Metabolites identified in the exocarp berries by GC-MS.

Abbreviation: [a]. quantification ion: [b]. retention time.

Compound	Q. Ion[a]	R. time[b]	Riesling	Pinot gris	Pinot noir	Croatina	ANOVA ($p \leq 0.05$)
**3-P-Glyceric acid**	357	28.81	**79.07** ± 11.02	**68.39** ±11.29	**60.56** ± 6.37	**69.57** ± 5.80	No
**4-Hydroxyproline**	158	22.29	**124.63** ± 21.76	**170.75** ± 22.80	**121.07** ± 19.89	**106.12** ± 12.39	No
**Alanine**	188	20.31	**0.78** ± 0.29	**0.31** ± 0.22	**0.63** ± 0.28	**0.16** ± 0.16	No
**Ascorbic acid**	419	31.21	**40.29** ± 6.37	**60.89** ± 5.70	**71.19** ± 7.89	**72.47** ± 5.02	Yes
**Aspartic acid**	233	23.35	**280.24** ± 32.01	**226.79** ± 21.24	**224.15** ± 30.93	**180.96** ± 21.31	Yes
**Caffeic acid**	219	33.84	**10.63** ± 0.70	**11.47** ± 0.61	**12.29** ± 0.41	**11.05** ± 0.54	No
**Cinnamic acid**	220	21.54	**177.57** ± 26.98	**147.48** ± 19.51	**165.81** ±19.75	**175.73** ± 17.02	No
**Citric acid**	465	29.02	**4.10** ± 1.55	**5.94** ± 1.40	**6.80** ± 1.56	**5.53** ± 1.49	No
**Coumaric acid**	235	30.78	**71.60** ± 8.29	**69.53** ± 3.69	**74.28** ± 5.23	**75.06** ± 4.86	No
**Cysteine**	218	24.11	**63.58** ± 7.18	**87.26** ± 6.27	**100.30** ± 11.53	**90.03** ± 5.91	Yes
**Erythrose**	205	22.12	**37772.8** ± 6569.2	**49968.8** ± 6724.1	**57869.1** ± 5959.5	**47267.7** ± 6144.5	No
**Fructose**	307	30.23	**184.17** ± 8.08	**168.99** ± 10.87	**174.07** ± 10.11	**191.83** ± 12.76	No
**Fructose-1.6-P**	299	41.98	**34.63** ± 7.88	**56.11** ± 6.39	**54.84** ± 7.53	**65.12** ± 7.36	Yes
**Fumaric acid**	147	19.57	**425.92** ± 36.45	**440.06** ± 42.66	**516.25** ± 41.96	**391.78** ± 44.96	No
**GABA**	174	23.51	**66.32** ± 10.79	**66.24** ± 10.34	**56.49** ± 11.72	**54.09** ± 7.23	No
**Gallic acid**	458	31.16	**52.11** ± 12.08	**69.37** ± 8.40	**85.22** ± 15.96	**101.45** ± 10.52	Yes
**Gentiobiose**	451	42.21	**847.07** ± 128.68	**1250.35** ± 134.24	**819.05** ± 134.14	**1021.06** ± 105.73	No
**Glucose**	480	30.51	**17.10** ± 0.50	**16.60** ± 0.47	**17.36** ± 0.47	**17.12** ± 0.54	No
**Glutamic acid**	246	25.40	**4.96** ± 1.34	**3.29** ± 1.23	**4.80** ± 1.28	**3.18** ± 1.19	No
**Glutamine xTMS**	191	31.91	**12782.4** ± 1739.0	**17636.3** ± 2013.7	**18330.7** ± 4085.9	**17597.8** ± 1982.3	No
**Glyceraldehyde**	147	16.61	**376.27** ± 12.36	**372.33** ± 22.53	**411.97** ± 14.37	**404.80** ± 14.91	No
**Glyceraldheyde-3-P**	328	27.25	**1.98** ± 0.01	**1.96** ± 0.01	**1.97** ± 0.00	**1.96** ± 0.01	No
**Glyceric acid**	147	19.32	**134.06** ± 15.41	**156.32** ± 8.21	**198.05** ± 21.84	**167.49** ± 13.19	Yes
**Glycerol**	205	18.02	**9.85** ± 0.08	**9.77** ± 0.12	**10.37** ± 0.17	**9.93** ± 0.08	No
**Glycine**	174	18.71	**36.95** ± 8.56	**53.76** ± 8.75	**56.31** ± 10.66	**48.09** ± 5.15	No
**Glycolic acid**	177	12.55	**11.39** ± 4.18	**14.08** ± 1.49	**16.51** ± 2.14	**12.99** ± 2.52	No
**Homoserine**	219	22.11	**441.76** ± 64.40	**587.39** ± 48.26	**587.25** ± 73.12	**532.73** ± 62.05	No
**Isoleucine**	158	18.41	**60.02** ± 14.58	**91.03** ± 11.78	**89.38** ± 11.46	**72.70** ± 8.04	No
**Leucine**	158	17.88	**16.21** ± 4.07	**17.14** ± 1.95	**12.91** ± 2.55	**9.26** ± 2.53	No

**Malic acid**	233	22.72	**84.04** ± 12.26	**133.52** ± 15.50	**134.58** ± 19.91	**133.57** ± 15.94	Yes
**Malonic acid - 3TMS**	305	24.95	**6.58** ± 2.20	**10.00** ± 2.27	**9.38** ± 2.54	**11.31** ± 2.11	No
**Mannitol**	524	31.07	**6.02** ± 0.57	**5.58** ± 0.45	**6.84** ± 0.42	**6.62** ± 0.35	No
**Mannose**	381	30.29	**27.00** ± 2.02	**29.34** ± 2.02	**30.67** ± 2.14	**29.45** ± 1.50	No
**Methionine**	176	23.28	**76.01** ± 18.51	**91.76** ± 12.41	**92.11** ± 10.57	**90.66** ± 10.54	No
**Ornithine**	174	27.66	**68.49** ± 15.71	**91.42** ± 13.39	**72.08** ± 16.33	**101.20** ± 10.49	No
**Oxaloacetic acid**	290	22.19	**32.95** ± 8.55	**59.66** ± 7.69	**56.61** ± 8.42	**42.58** ± 6.36	Yes
**Phenylalanine**	218	25.38	**445.04** ± 57.58	**607.10** ± 54.28	**639.53** ± 39.43	**679.62** ± 57.63	Yes
**Proline**	142	18.44	**10.81** ± 0.40	**10.12** ± 0.36	**10.44** ± 0.36	**9.87** ± 0.29	No
**Pyruvic acid**	218	11.86	**10.47** ± 0.09	**10.81** ± 0.18	**11.10** ± 0.13	**11.28** ± 0.16	No
**Ribose**	307	26.63	**89.32** ± 5.68	**109.41** ± 6.40	**121.58** ± 4.48	**124.14** ± 6.88	Yes
**Sarcosine**	218	13.38	**232.50** ± 13.76	**232.84** ± 17.86	**236.52** ± 16.44	**233.74** ± 13.66	No
**Sedoheptulose**	204	30.95	**104.72** ± 2.17	**107.58** ± 2.90	**121.04** ± 2.75	**129.87** ± 4.69	Yes
**Serine - 3TMS**	218	19.99	**16.37** ± 3.41	**16.46** ± 2.44	**13.48** ± 2.95	**9.58** ± 2.70	No
**Shikimic acid**	204	28.83	**11.72** ± 2.64	**7.86** ± 2.62	**9.53** ± 3.01	**12.21** ± 2.79	No
**Sorbitol**	319	31.19	**66.27** ± 2.05	**74.77** ± 3.07	**82.85** ± 2.29	**82.57** ± 2.28	Yes
**Succinic acid**	247	18.87	**6.45** ± 1.21	**8.63** ± 0.32	**8.99** ± 0.37	**8.58** ± 0.30	Yes
**Sucrose**	361	41.06	**40.47** ± 0.50	**41.55** ± 0.76	**38.15** ± 2.89	**41.89** ± 0.49	No
**Threonine**	218	20.60	**1094.71** ± 140.85	**1409.90** ± 136.93	**1604.62** ± 241.43	**1358.31** ± 165.64	No
**Xylitol**	217	27.09	**20772.8** ± 4383.5	**37750.6** ± 4575.2	**27943.2** ± 3151.2	**31404.4** ± 4168.4	Yes
**Xylose**	364	26.22	**25.38** ± 7.43	**35.24** ± 4.19	**41.11** ± 5.07	**31.54** ± 4.91	No
**Xylulose**	205	26.56	**558.25** ± 82.59	**854.23** ± 89.24	**1012.47** ± 86.73	**902.85** ± 80.43	Yes
**α-Ketoglutaric acid**	198	24.39	**18.67** ± 4.34	**21.58** ± 3.28	**16.23** ± 3.22	**13.80** ± 3.97	No
**beta-Alanine**	174	21.38	**20.44** ± 4.59	**22.96** ± 4.34	**22.98** ± 4.57	**15.68** ± 3.64	No
**cis-Aconitic acid**	229	27.62	**46.29** ± 12.48	**78.67** ± 8.38	**44.71** ± 10.41	**70.00** ± 7.39	No
**myo-Inositol**	305	33.53	**180.50** ± 4.27	**205.81** ± 6.16	**225.41** ± 8.91	**201.24** ± 5.39	Yes
**Maltose**	361	42.34	**316.51** ± 53.18	**487.45** ± 44.70	**324.99** ± 62.01	**474.17** ± 32.14	No
