# Supplementary material for: Proteomic and metabolic traits of grape exocarp to explain different anthocyanin concentrations of the cultivars
Source: Front Plant Sci. 2015 Aug 4;6:603. doi: 10.3389/fpls.2015.00603 (PMC4523781; doi:10.3389/fpls.2015.00603)

Supplementary Material - LC-ESI-MS/MS analysis

Proteomic and metabolic traits of grape exocarp to explain different anthocyanin concentrations of the cultivars

Alfredo Simone Negri[1], Bhakti Prinsi[1], Osvaldo Failla[1], Attilio Scienza[1], Luca Espen[1]*

[1]Dipartimento di Scienze Agrarie e Ambientali, Produzione, Territorio, Agroenergia (DISAA), Università degli Studi di Milano, Milano, Italia

*** Correspondence:** Luca Espen, Dipartimento di Scienze Agrarie e Ambientali, Produzione, Territorio, Agroenergia (DISAA), Università degli Studi di Milano, via Celoria n.2, Milano, 20133, Italia.
luca.espen@unimi.it

Supplementary Table S1| Detailed information and statistical data about protein characterization by LC-ESI-MS/MS.
Spot ID: spot number reported in Table 3, Figure 5 and Figure 1S. n. pep. U (T): n. of identified peptides per protein. U: unique. T: total. aa cov. (%): percentage of amino acid coverage of the protein. Protein S: Spectrum Mill Score. z: charge state of the precursor. Score: Spectrum Mill peptide score. Fwd-Rev Score: difference between scores obtained using forward database and the associated reversed one. SPI (%): Scored Peak Intensity (%). Sequence map: the symbols, \, / and |, represent b-ions, y-ions, and both bions and y-ions, respectively. m:oxidized methionine. MH+ matched (D): mass of mono-charged peptide (MH+) matched with the precursor. MH+ mass shift (D): difference between the precursor mass and MH+.

<table>
<tr><th>Spot ID</th><th>Version AN</th><th>Description (species)</th><th>n. pep u(t)</th><th>AA cov (%)</th><th>Protein S</th><th>z</th><th>Score</th><th>Fwd-Rev Score</th><th>SPI (%)</th><th>Sequence Map</th><th>m/z measured (Da)</th><th>MH+ matched (Da)</th><th>MH+ mass shift (Da)</th></tr>
<tr><td>1070</td><td>XP_002282146.1</td><td>**Cell division control protein 48 homolog**</td><td>13(22)</td><td>24%</td><td>231.21</td><td>2</td><td>22.37</td><td>13.05</td><td>98.1</td><td>(R)E N/P/E/A/M|E/E/D\V|D|E|E V|A\E\I\K(A)</td><td>1039.436</td><td>2075.896</td><td>1.9686</td></tr>
<tr><td></td><td></td><td>*(Vitis vinifera)*</td><td></td><td></td><td></td><td>2</td><td>21.97</td><td>10.85</td><td>98.9</td><td>(R)E T V/V/E|V|P N|V|S/W/E/D/I|G|G L E\N V K(R)</td><td>1157.3584</td><td>2313.161</td><td>0.5486</td></tr>
<tr><td></td><td></td><td></td><td></td><td></td><td></td><td>2</td><td>21.25</td><td>13</td><td>100</td><td>(K)Y Q|A|F|A|Q|T|L|Q|Q|S R(G)</td><td>721.2521</td><td>1440.723</td><td>0.7739</td></tr>
<tr><td></td><td></td><td></td><td></td><td></td><td></td><td>2</td><td>20.91</td><td>12.16</td><td>98.8</td><td>(R)I V/S/Q|L|L|T|L|M|D|G L\K(T)</td><td>715.8014</td><td>1430.829</td><td>-0.2332</td></tr>
<tr><td></td><td></td><td></td><td></td><td></td><td></td><td>2</td><td>20.75</td><td>15.43</td><td>100</td><td>(R)L V V D E A|V/N/D/D|N/S V/V/A/L|H|P D/T M E K(L)</td><td>1255.5288</td><td>2510.208</td><td>-0.1576</td></tr>
<tr><td></td><td></td><td></td><td></td><td></td><td></td><td>2</td><td>19.22</td><td>9.56</td><td>94.5</td><td>(R)V L/N Q/L|L|T/E|M|D|G|M\S A K(K)</td><td>825.8811</td><td>1649.824</td><td>0.9312</td></tr>
<tr><td></td><td></td><td></td><td></td><td></td><td></td><td>3</td><td>18.5</td><td>9.48</td><td>92.8</td><td>(R)A/H|V|I|V|I|G/A|T|N R P N S I D|P A L R(R)</td><td>705.5258</td><td>2114.183</td><td>0.38</td></tr>
<tr><td></td><td></td><td></td><td></td><td></td><td></td><td>3</td><td>17.76</td><td>7.95</td><td>95.4</td><td>(R)L V/V/D/E/A|V|N D/D/N S V/V/A/L/H|P D T M E K(L)</td><td>837.2419</td><td>2510.208</td><td>-0.4968</td></tr>
<tr><td></td><td></td><td></td><td></td><td></td><td></td><td>2</td><td>17.16</td><td>7.14</td><td>92.8</td><td>(R)I V/S Q|L|L|T|L|m\D G L K(T)</td><td>724.845</td><td>1430.829</td><td>17.854</td></tr>
<tr><td></td><td></td><td></td><td></td><td></td><td></td><td>2</td><td>17.15</td><td>4.28</td><td>90.7</td><td>(K)L/S|E/D|V\D|L|E|R(I)</td><td>538.3862</td><td>1075.527</td><td>0.2385</td></tr>
<tr><td></td><td></td><td></td><td></td><td></td><td></td><td>2</td><td>16.82</td><td>5.24</td><td>94.4</td><td>(R)L D Q\L|I|Y|I|P|L|P\D E D\S R(H)</td><td>894.1538</td><td>1786.922</td><td>0.3782</td></tr>
<tr><td></td><td></td><td></td><td></td><td></td><td></td><td>2</td><td>15.78</td><td>2.05</td><td>92.7</td><td>(R)L D Q L\I|Y|I|P/L|P\D E D\S R(H)</td><td>893.8536</td><td>1786.922</td><td>-0.2222</td></tr>
<tr><td></td><td></td><td></td><td></td><td></td><td></td><td>2</td><td>15.5</td><td>7.56</td><td>85.7</td><td>(R)I/V/S Q\L|L|T|L\m\D G L K(T)</td><td>724.8195</td><td>1430.829</td><td>17.803</td></tr>
<tr><td></td><td></td><td></td><td></td><td></td><td></td><td>3</td><td>15.23</td><td>5.96</td><td>94.7</td><td>(R)L V/V/D/E/A|V|N D/D/N S V/V A/L H|P D T M E K(L)</td><td>837.7992</td><td>2510.208</td><td>1.1751</td></tr>
<tr><td></td><td></td><td></td><td></td><td></td><td></td><td>2</td><td>14.94</td><td>5.64</td><td>92</td><td>(K)T/A/L/G|T|S/N|P S\A L R(E)</td><td>594.3448</td><td>1187.638</td><td>0.0444</td></tr>
<tr><td></td><td></td><td></td><td></td><td></td><td></td><td>3</td><td>14.82</td><td>6.35</td><td>88.3</td><td>(R)L V/V/D/E/A|V|N D|D N S V V\A|L H/P D\T m E K(L)</td><td>843.3574</td><td>2510.208</td><td>17.8497</td></tr>
<tr><td></td><td></td><td></td><td></td><td></td><td></td><td>2</td><td>14.02</td><td>4.95</td><td>85.4</td><td>(K)D F|S|T|A/I|L|E\R(K)</td><td>526.8575</td><td>1051.542</td><td>1.1659</td></tr>
<tr><td></td><td></td><td></td><td></td><td></td><td></td><td>2</td><td>12.43</td><td>3.21</td><td>95</td><td>(K)T/A/L/G/T/S|N|P S A/L R(E)</td><td>594.6219</td><td>1187.638</td><td>0.5986</td></tr>
<tr><td></td><td></td><td></td><td></td><td></td><td></td><td>3</td><td>11.75</td><td>2.24</td><td>86.4</td><td>(R)E L/Q/E|T V/Q/Y/P V E H\P\E K(F)</td><td>609.4927</td><td>1825.897</td><td>0.5669</td></tr>
<tr><td></td><td></td><td></td><td></td><td></td><td></td><td>2</td><td>11.56</td><td>2.97</td><td>92.3</td><td>(R)E I/D|I|G|V/P D E V\G R(L)</td><td>649.9077</td><td>1298.659</td><td>0.1495</td></tr>
<tr><td></td><td></td><td></td><td></td><td></td><td></td><td>2</td><td>11.5</td><td>4.58</td><td>100</td><td>(R)V L N Q/L|L|T|E|m D G M\S A K(K)</td><td>833.9009</td><td>1649.824</td><td>16.9708</td></tr>
<tr><td></td><td></td><td></td><td></td><td></td><td></td><td>2</td><td>11.26</td><td>4.11</td><td>94.5</td><td>(R)V L/N Q/L/L/T/E/M|D G m\S A K(K)</td><td>833.7894</td><td>1649.824</td><td>16.7478</td></tr>
<tr><td>1088</td><td>XP_002266780.1</td><td>**elongation factor 2**</td><td>6(7)</td><td>9%</td><td>104.86</td><td>2</td><td>21.84</td><td>10.1</td><td>97.2</td><td>(K)D L/Q/D/D|F|M|G|G|A|E|I\V\K(S)</td><td>769.5242</td><td>1537.72</td><td>0.3208</td></tr>
<tr><td></td><td></td><td>*(Vitis vinifera)*</td><td></td><td></td><td></td><td>2</td><td>21.39</td><td>8.99</td><td>97.8</td><td>(K)A Y/L|P V|V|E|S/F|G|F|S|G\T L R(A)</td><td>871.9878</td><td>1742.911</td><td>0.0571</td></tr>
<tr><td></td><td></td><td></td><td></td><td></td><td></td><td>2</td><td>19.38</td><td>11.68</td><td>97.3</td><td>(K)A Y/L/P/V|V|E|S/F|G|F|S\G T L R(A)</td><td>872.563</td><td>1742.911</td><td>1.2075</td></tr>
<tr><td></td><td></td><td></td><td></td><td></td><td></td><td>2</td><td>19</td><td>9.83</td><td>100</td><td>(R)V E N L|Y|E|G|P L D|D\I|Y\A T\A I\R(N)</td><td>1026.8086</td><td>2052.028</td><td>0.5815</td></tr>
<tr><td></td><td></td><td></td><td></td><td></td><td></td><td>2</td><td>15</td><td>2.65</td><td>94</td><td>(K)G/V/Q|Y|L|N|E\I\K(D)</td><td>532.55</td><td>1063.578</td><td>0.5145</td></tr>
<tr><td></td><td></td><td></td><td></td><td></td><td></td><td>2</td><td>13.89</td><td>3.63</td><td>85.4</td><td>(R)I R|P V|L|T|V|N\K(M)</td><td>520.4478</td><td>1039.662</td><td>0.2261</td></tr>
<tr><td></td><td></td><td></td><td></td><td></td><td></td><td>2</td><td>13.74</td><td>3.89</td><td>89.3</td><td>(R)L/W|G E|N|F|F|D|P S T R(K)</td><td>735.5527</td><td>1468.686</td><td>1.4126</td></tr>
<tr><td>1095</td><td>XP_002278138.1</td><td>**Aconitate hydratase, cytoplasmic**</td><td>10(13)</td><td>12%</td><td>147.78</td><td>3</td><td>17.54</td><td>13.31</td><td>88.4</td><td>(K)A D G H D T/I V\L\A|G A/E|Y|G|S|G|S\S R(D)</td><td>655.4337</td><td>1962.915</td><td>1.3714</td></tr>
<tr><td></td><td></td><td>*(Vitis vinifera)*</td><td></td><td></td><td></td><td>2</td><td>17.36</td><td>7.49</td><td>89.5</td><td>(R)m F|V|D|Y|N|E|P Q V\E\R(F)</td><td>771.6919</td><td>1526.694</td><td>15.6821</td></tr>
<tr><td></td><td></td><td></td><td></td><td></td><td></td><td>3</td><td>15.72</td><td>4.33</td><td>91.9</td><td>(K)A/G/E|D|A/D/T/L/G/L/T G/H E R(Y)</td><td>514.7532</td><td>1541.719</td><td>0.526</td></tr>
<tr><td></td><td></td><td></td><td></td><td></td><td></td><td>3</td><td>15.41</td><td>4.75</td><td>82.1</td><td>(K)I/S/E I/R|P G/Q D\V|T|V/T|T/D N G K(S)</td><td>644.3142</td><td>1929.988</td><td>0.9404</td></tr>
<tr><td></td><td></td><td></td><td></td><td></td><td></td><td>2</td><td>15.25</td><td>5.59</td><td>100</td><td>(K)L/Y|V|F|D|A|A|m\R(Y)</td><td>551.0531</td><td>1085.545</td><td>15.5541</td></tr>
<tr><td></td><td></td><td></td><td></td><td></td><td></td><td>2</td><td>14.8</td><td>2.75</td><td>91</td><td>(K)I/I|D|W|E|N T|S P\K(Q)</td><td>602.0405</td><td>1202.605</td><td>0.4685</td></tr>
<tr><td></td><td></td><td></td><td></td><td></td><td></td><td>2</td><td>14.29</td><td>4.71</td><td>93.2</td><td>(K)F Y|S|L|P/A L\N D|P R(I)</td><td>646.9303</td><td>1292.663</td><td>0.19</td></tr>
<tr><td></td><td></td><td></td><td></td><td></td><td></td><td>2</td><td>13.98</td><td>2.36</td><td>99.2</td><td>(K)Y/L|L|Q/S|G/L\Q\K(Y)</td><td>525.5978</td><td>1049.599</td><td>0.5894</td></tr>
<tr><td></td><td></td><td></td><td></td><td></td><td></td><td>2</td><td>13.48</td><td>4.75</td><td>90</td><td>(R)M/F|V/D|Y|N E|P Q V E\R(F)</td><td>764.033</td><td>1526.694</td><td>0.3643</td></tr>
<tr><td></td><td></td><td></td><td></td><td></td><td></td><td>2</td><td>13.18</td><td>3.18</td><td>96.8</td><td>(R)I/L|L|E|S|A\I\R(N)</td><td>457.8588</td><td>914.567</td><td>0.1434</td></tr>
<tr><td></td><td></td><td></td><td></td><td></td><td></td><td>2</td><td>12.5</td><td>4.59</td><td>100</td><td>(K)L/Y|V|F/D|A\A M\R(Y)</td><td>543.4091</td><td>1085.545</td><td>0.2661</td></tr>
<tr><td></td><td></td><td></td><td></td><td></td><td></td><td>2</td><td>10.25</td><td>2.66</td><td>100</td><td>(K)F/S/F/H|G/Q|P A E\L\K(H)</td><td>630.8062</td><td>1260.637</td><td>-0.032</td></tr>
<tr><td></td><td></td><td></td><td></td><td></td><td></td><td>3</td><td>10.07</td><td>5.03</td><td>77</td><td>(K)A D G H D T I V\L A G\A|E|Y|G\S/G\S\S R(D)</td><td>654.8086</td><td>1962.915</td><td>-0.5039</td></tr>
<tr><td>1096</td><td>XP_002278138.1</td><td>**Aconitate hydratase, cytoplasmic**</td><td>13(13)</td><td>17%</td><td>194.62</td><td>3</td><td>18.15</td><td>8.83</td><td>95.5</td><td>(K)I/S/E/I/R|P/G/Q D\V|T|V/T|T D N\G K(S)</td><td>644.5181</td><td>1929.988</td><td>1.5521</td></tr>
<tr><td></td><td></td><td>*(Vitis vinifera)*</td><td></td><td></td><td></td><td>2</td><td>16.96</td><td>8.09</td><td>93.9</td><td>(R)M F|V|D|Y|N E|P Q\V\E\R(F)</td><td>763.9614</td><td>1526.694</td><td>0.2211</td></tr>
<tr><td></td><td></td><td></td><td></td><td></td><td></td><td>2</td><td>16.47</td><td>8.68</td><td>92.8</td><td>(K)F Y|S|L|P|A/L/N D|P R(I)</td><td>647.4448</td><td>1292.663</td><td>1.219</td></tr>
<tr><td></td><td></td><td></td><td></td><td></td><td></td><td>3</td><td>16.4</td><td>5.13</td><td>91.6</td><td>(K)A/G/E/D/A/D|T/L/G L/T/G|H E\R(Y)</td><td>514.5259</td><td>1541.719</td><td>-0.1559</td></tr>
<tr><td></td><td></td><td></td><td></td><td></td><td></td><td>2</td><td>16.31</td><td>7.25</td><td>90.7</td><td>(K)A/S/E/L/G/L/E|V|K|P W I\K(T)</td><td>736.1212</td><td>1469.836</td><td>1.3989</td></tr>
<tr><td></td><td></td><td></td><td></td><td></td><td></td><td>2</td><td>16.14</td><td>4.79</td><td>94.4</td><td>(K)I/I|D|W|E|N\T|S P\K(Q)</td><td>602.0109</td><td>1202.605</td><td>0.4093</td></tr>
<tr><td></td><td></td><td></td><td></td><td></td><td></td><td>3</td><td>15.11</td><td>5.16</td><td>84.7</td><td>(K)G I|L|T|S|V|P K/P\G G/G E F G K(F)</td><td>515.4192</td><td>1543.848</td><td>0.3952</td></tr>
<tr><td></td><td></td><td></td><td></td><td></td><td></td><td>2</td><td>15.08</td><td>7.13</td><td>85</td><td>(R)S D E T/V|A/M|I|E|A\Y\L\R(A)</td><td>749.6847</td><td>1497.725</td><td>0.6368</td></tr>
<tr><td></td><td></td><td></td><td></td><td></td><td></td><td>2</td><td>14.29</td><td>4.53</td><td>91.8</td><td>(K)F/S/F/H|G/Q|P A\E|L\K(H)</td><td>631.4265</td><td>1260.637</td><td>1.2086</td></tr>
<tr><td></td><td></td><td></td><td></td><td></td><td></td><td>2</td><td>13.73</td><td>3.4</td><td>99</td><td>(K)T S|L|A|P|G\S\G V V T K(Y)</td><td>558.9435</td><td>1116.626</td><td>0.2538</td></tr>
<tr><td></td><td></td><td></td><td></td><td></td><td></td><td>2</td><td>12.47</td><td>2.08</td><td>87.1</td><td>(R)I/L|L|E|S|A\I R(N)</td><td>458.0204</td><td>914.567</td><td>0.4666</td></tr>
<tr><td></td><td></td><td></td><td></td><td></td><td></td><td>2</td><td>12.43</td><td>2.49</td><td>97.7</td><td>(K)L/Y|V|F|D|A\A\M R(Y)</td><td>543.1685</td><td>1085.545</td><td>-0.2151</td></tr>
</table>

<table>
<tr><td></td><td></td><td></td><td></td><td></td><td></td><td>3</td><td>11.08</td><td>5.65</td><td>87</td><td>(K)A D G H D T I V L\A/G\A E|Y|G\S\G\S\S R(D)</td><td>655.134</td><td>1962.915</td><td>0.4723</td></tr>
<tr><td>1097</td><td>XP_002278138.1</td><td>Aconitate hydratase, cytoplasmic</td><td>18(28)</td><td>26%</td><td>306.35</td><td>2</td><td>22.47</td><td>12.61</td><td>99</td><td>(R)N/M/L\V|V|P/P/G S/G I|V/H|Q/V/N\L E Y L G R(V)</td><td>1197.2349</td><td>2392.281</td><td>1.182</td></tr>
<tr><td></td><td></td><td>(Vitis vinifera)</td><td></td><td></td><td></td><td>2</td><td>21.81</td><td>10.69</td><td>97.6</td><td>(K)F V/E/F/Y|G/E/G|M|G/E|L|S|L A\D R(A)</td><td>960.7502</td><td>1919.884</td><td>0.6087</td></tr>
<tr><td></td><td></td><td></td><td></td><td></td><td></td><td>3</td><td>19.94</td><td>4.99</td><td>98.4</td><td>(R)A T/I/A/N/M|S|P/E/Y/G A\T\M/G F/F/P V D H V T L Q Y L K(L)</td><td>1035.0332</td><td>3101.511</td><td>1.574</td></tr>
<tr><td></td><td></td><td></td><td></td><td></td><td></td><td>3</td><td>19.86</td><td>7.27</td><td>92.8</td><td>(R)Y K A D G H\D/T I V|L|A|G|A|E|Y|G|S\G\S\S\R(D)</td><td>752.2479</td><td>2254.074</td><td>0.6557</td></tr>
<tr><td></td><td></td><td></td><td></td><td></td><td></td><td>2</td><td>19.5</td><td>9.97</td><td>100</td><td>(K)I S E/I R|P/G Q D|V|T|V|T|T D|N\G K(S)</td><td>966.1305</td><td>1929.988</td><td>1.2661</td></tr>
<tr><td></td><td></td><td></td><td></td><td></td><td></td><td>2</td><td>19.08</td><td>9.06</td><td>96.2</td><td>(R)M/F|V|D|Y|N/E|P Q|V|E\R(F)</td><td>763.8933</td><td>1526.694</td><td>0.0849</td></tr>
<tr><td></td><td></td><td></td><td></td><td></td><td></td><td>2</td><td>18.67</td><td>7.97</td><td>94.4</td><td>(K)F V/E/F/Y|G/E|G\M|G E|L|S L A\D\R(A)</td><td>960.876</td><td>1919.884</td><td>0.8603</td></tr>
<tr><td></td><td></td><td></td><td></td><td></td><td></td><td>2</td><td>18.29</td><td>8.86</td><td>96.2</td><td>(R)S D E\T/V|A|M/I|E|A|Y\L\R(A)</td><td>749.6981</td><td>1497.725</td><td>0.6636</td></tr>
<tr><td></td><td></td><td></td><td></td><td></td><td></td><td>2</td><td>17.57</td><td>4.9</td><td>94.8</td><td>(K)F Y|S|L|P A/L|N/D|P R(I)</td><td>646.8088</td><td>1292.663</td><td>-0.053</td></tr>
<tr><td></td><td></td><td></td><td></td><td></td><td></td><td>2</td><td>17.22</td><td>8.05</td><td>94.4</td><td>(K)F/S/F/H|G|Q|P\A\E|L\K(H)</td><td>631.071</td><td>1260.637</td><td>0.4976</td></tr>
<tr><td></td><td></td><td></td><td></td><td></td><td></td><td>2</td><td>16.53</td><td>9.87</td><td>86.2</td><td>(K)A/G/E/D/A/D|T|L G|L|T/G H E\R(Y)</td><td>771.406</td><td>1541.719</td><td>0.0857</td></tr>
<tr><td></td><td></td><td></td><td></td><td></td><td></td><td>3</td><td>16.2</td><td>7.12</td><td>82.6</td><td>(R)A T/I/A/N/M|S/P E Y G A|T\m G F/F/P/V D H V T L Q Y L K(L)</td><td>1040.4978</td><td>3101.511</td><td>17.9678</td></tr>
<tr><td></td><td></td><td></td><td></td><td></td><td></td><td>3</td><td>16.03</td><td>7.33</td><td>85</td><td>(K)A D G H D T I V\L\A G|A/E|Y|G|S|G|S\S R(D)</td><td>655.3256</td><td>1962.915</td><td>1.0471</td></tr>
<tr><td></td><td></td><td></td><td></td><td></td><td></td><td>2</td><td>15.85</td><td>6.66</td><td>91.3</td><td>(K)A/S/E/L/G/L/E|V|K|P W I\K(T)</td><td>735.7532</td><td>1469.836</td><td>0.6629</td></tr>
<tr><td></td><td></td><td></td><td></td><td></td><td></td><td>2</td><td>15.8</td><td>11.12</td><td>84.5</td><td>(R)S E/N A V|Q/A|N/M D|L/E|F Q R(N)</td><td>877.0598</td><td>1751.802</td><td>1.3106</td></tr>
<tr><td></td><td></td><td></td><td></td><td></td><td></td><td>2</td><td>15.69</td><td>2.38</td><td>97.7</td><td>(K)Y/L|L|Q/S|G/L\Q\K(Y)</td><td>525.4131</td><td>1049.599</td><td>0.22</td></tr>
<tr><td></td><td></td><td></td><td></td><td></td><td></td><td>2</td><td>15.14</td><td>9.5</td><td>86.9</td><td>(K)I S E/I\R|P/G Q D|V\T\V|T|T D\N\G K(S)</td><td>966.1285</td><td>1929.988</td><td>1.2621</td></tr>
<tr><td></td><td></td><td></td><td></td><td></td><td></td><td>3</td><td>14.49</td><td>4.7</td><td>88.9</td><td>(K)A/G/E/D/A/D|T\L/G\L/T/G H E R(Y)</td><td>514.8391</td><td>1541.719</td><td>0.7837</td></tr>
<tr><td></td><td></td><td></td><td></td><td></td><td></td><td>3</td><td>13.54</td><td>2.33</td><td>83.1</td><td>(K)G/I/L|T|S/V|P K/P\G G/G E F G K(F)</td><td>515.108</td><td>1543.848</td><td>-0.5384</td></tr>
<tr><td></td><td></td><td></td><td></td><td></td><td></td><td>2</td><td>13.51</td><td>8.24</td><td>70.5</td><td>(R)S E N A\V|Q|A|N/M/D/L/E|F\Q R(N)</td><td>876.5859</td><td>1751.802</td><td>0.3628</td></tr>
<tr><td></td><td></td><td></td><td></td><td></td><td></td><td>2</td><td>13.48</td><td>4.31</td><td>98.8</td><td>(K)I/I|D|W|E|N T|S\P\K(Q)</td><td>602.1208</td><td>1202.605</td><td>0.6291</td></tr>
<tr><td></td><td></td><td></td><td></td><td></td><td></td><td>3</td><td>12.71</td><td>4.15</td><td>73.9</td><td>(K)A D G H D T I V\L A G\A/E|Y|G\S|G\S\S\R(D)</td><td>655.022</td><td>1962.915</td><td>0.1363</td></tr>
<tr><td></td><td></td><td></td><td></td><td></td><td></td><td>2</td><td>12.35</td><td>2.26</td><td>95.2</td><td>(K)L/Y|V|F/D/A\A|M\R(Y)</td><td>543.4822</td><td>1085.545</td><td>0.4123</td></tr>
<tr><td></td><td></td><td></td><td></td><td></td><td></td><td>3</td><td>11.34</td><td>2.3</td><td>95</td><td>(K)I N/P/L/V|P V D/L/V I D|H S V Q V D V T R(S)</td><td>777.2617</td><td>2328.292</td><td>1.4784</td></tr>
<tr><td></td><td></td><td></td><td></td><td></td><td></td><td>3</td><td>11.32</td><td>2.31</td><td>83.9</td><td>(K)F Y\S/L|P\A|L/N/D|P R(I)</td><td>431.7906</td><td>1292.663</td><td>0.6939</td></tr>
<tr><td></td><td></td><td></td><td></td><td></td><td></td><td>3</td><td>10.97</td><td>4.4</td><td>89.6</td><td>(K)I/S/E/I R\P G/Q D V|T|V/T|T D N G K(S)</td><td>644.4562</td><td>1929.988</td><td>1.3664</td></tr>
<tr><td></td><td></td><td></td><td></td><td></td><td></td><td>2</td><td>10.21</td><td>7.1</td><td>79</td><td>(K)F V E F/Y|G/E|G|M|G E\L\S L A D R(A)</td><td>960.5214</td><td>1919.884</td><td>0.1511</td></tr>
<tr><td></td><td></td><td></td><td></td><td></td><td></td><td>2</td><td>10.17</td><td>4.49</td><td>87.4</td><td>(K)F V E/F Y/G E|G/M|G E L|S L A D R(A)</td><td>961.1703</td><td>1919.884</td><td>1.4489</td></tr>
<tr><td>1114</td><td>XP_002263337.1</td><td>Aconitate hydratase 1</td><td>5(6)</td><td>8%</td><td>75.85</td><td>2</td><td>17.82</td><td>7.86</td><td>97.9</td><td>(K)S E G Q D/T I|I|L|A|G/A/E|Y|G S G\S S\R(D)</td><td>999.7297</td><td>1997.941</td><td>0.5111</td></tr>
<tr><td></td><td></td><td>(Vitis vinifera)</td><td></td><td></td><td></td><td>2</td><td>16.38</td><td>8.94</td><td>90.2</td><td>(R)A N/N/M/F|V|D|Y|S|Q|P Q V\E K(V)</td><td>886.1821</td><td>1769.816</td><td>1.5406</td></tr>
<tr><td></td><td></td><td></td><td></td><td></td><td></td><td>2</td><td>16.36</td><td>7.79</td><td>97.7</td><td>(K)V V/E/F/S/Y|H|G|T|P\A Q L R(H)</td><td>802.551</td><td>1603.823</td><td>0.272</td></tr>
<tr><td></td><td></td><td></td><td></td><td></td><td></td><td>2</td><td>12.95</td><td>4.69</td><td>98.3</td><td>(K)L/S|V|F|D|A/A M\R(Y)</td><td>505.1704</td><td>1009.514</td><td>-0.18</td></tr>
<tr><td></td><td></td><td></td><td></td><td></td><td></td><td>2</td><td>12.34</td><td>4.47</td><td>92.1</td><td>(K)F V|E|F/Y|G|E/G M R(E)</td><td>618.2024</td><td>1234.556</td><td>0.8414</td></tr>
<tr><td></td><td></td><td></td><td></td><td></td><td></td><td>2</td><td>11.25</td><td>5.43</td><td>100</td><td>(K)F V|E|F/Y|G/E/G m\R(E)</td><td>626.095</td><td>1234.556</td><td>16.6266</td></tr>
<tr><td>1136</td><td>XP_002273244.1</td><td>Heat shock cognate protein 80-like</td><td>15(17)</td><td>24%</td><td>250.45</td><td>2</td><td>20.42</td><td>8.26</td><td>96.7</td><td>(K)T M/E|I|N|P/E|N\P I|M/E\E\L R(K)</td><td>908.8787</td><td>1815.862</td><td>0.8886</td></tr>
<tr><td></td><td></td><td>(Vitis vinifera)</td><td></td><td></td><td></td><td>2</td><td>19.77</td><td>9.81</td><td>95.2</td><td>(K)A D/L|V|N|N L|G|T/I|A\R(S)</td><td>628.8055</td><td>1256.696</td><td>-0.092</td></tr>
<tr><td></td><td></td><td></td><td></td><td></td><td></td><td>2</td><td>19.08</td><td>9.4</td><td>96.5</td><td>(K)H S/E/F|I|S|Y|P I|S L|W|I E\K(T)</td><td>925.8485</td><td>1848.953</td><td>1.7367</td></tr>
<tr><td></td><td></td><td></td><td></td><td></td><td></td><td>2</td><td>18.65</td><td>6.89</td><td>96.9</td><td>(K)G I/V/D/S/E|D\L|P|L|N|I|S\R(E)</td><td>764.3456</td><td>1527.801</td><td>-0.1174</td></tr>
<tr><td></td><td></td><td></td><td></td><td></td><td></td><td>2</td><td>18.63</td><td>10.53</td><td>94.1</td><td>(K)S L/T/N/D|W|E/E|H\L\A\V\K(H)</td><td>771.5327</td><td>1541.759</td><td>0.2987</td></tr>
<tr><td></td><td></td><td></td><td></td><td></td><td></td><td>2</td><td>18.34</td><td>7.53</td><td>95.3</td><td>(K)L D/G Q|P D/L|F|I|H/I|V|P D\K(T)</td><td>854.1248</td><td>1706.911</td><td>0.3311</td></tr>
<tr><td></td><td></td><td></td><td></td><td></td><td></td><td>2</td><td>17.3</td><td>3.83</td><td>94.7</td><td>(R)E I|I/S/N/S S/D/A\L|D K(I)</td><td>646.5387</td><td>1291.638</td><td>0.4325</td></tr>
<tr><td></td><td></td><td></td><td></td><td></td><td></td><td>2</td><td>17.18</td><td>7.95</td><td>94.9</td><td>(K)S G/D/E/L/T/S|L/K/D|Y V\T R(M)</td><td>792.5319</td><td>1583.791</td><td>0.2654</td></tr>
<tr><td></td><td></td><td></td><td></td><td></td><td></td><td>3</td><td>16.09</td><td>5.12</td><td>90.4</td><td>(K)S/L|T/N/D|W/E/E/H/L A V\K(H)</td><td>514.8369</td><td>1541.759</td><td>0.7367</td></tr>
<tr><td></td><td></td><td></td><td></td><td></td><td></td><td>2</td><td>16.07</td><td>3.39</td><td>89</td><td>(K)A/V|E|N|S|P\F L|E\K(L)</td><td>567.5042</td><td>1133.584</td><td>0.4174</td></tr>
<tr><td></td><td></td><td></td><td></td><td></td><td></td><td>2</td><td>15.86</td><td>4.8</td><td>91.2</td><td>(K)S/G|D|E|L|T|S|L\K(D)</td><td>475.3032</td><td>949.484</td><td>0.1155</td></tr>
<tr><td></td><td></td><td></td><td></td><td></td><td></td><td>2</td><td>15.64</td><td>7.12</td><td>92.1</td><td>(K)E G Q/S/D/I|Y Y|I|T\G E S K(K)</td><td>795.4695</td><td>1589.733</td><td>0.1988</td></tr>
<tr><td></td><td></td><td></td><td></td><td></td><td></td><td>3</td><td>15.2</td><td>5.94</td><td>89.6</td><td>(R)K/P/E/E/I/T\K|E/E/Y/A S\F Y\K(S)</td><td>621.4595</td><td>1861.922</td><td>0.4421</td></tr>
<tr><td></td><td></td><td></td><td></td><td></td><td></td><td>3</td><td>15</td><td>3.72</td><td>90.1</td><td>(K)L/D|G|Q|P D/L\F I H/I/V/P\D K(T)</td><td>570.0369</td><td>1706.911</td><td>1.1849</td></tr>
<tr><td></td><td></td><td></td><td></td><td></td><td></td><td>2</td><td>13.07</td><td>6.21</td><td>84.9</td><td>(K)E V/S/H/E|W/S L|V|N K(Q)</td><td>664.3251</td><td>1327.664</td><td>-0.0212</td></tr>
<tr><td></td><td></td><td></td><td></td><td></td><td></td><td>3</td><td>12.81</td><td>5.41</td><td>81.1</td><td>(R)M/K E G Q\S D\I/Y/Y/I|T|G E S K(K)</td><td>616.9216</td><td>1848.868</td><td>-0.1182</td></tr>
<tr><td></td><td></td><td></td><td></td><td></td><td></td><td>2</td><td>12.43</td><td>6.06</td><td>90.9</td><td>(R)A/P/F|D|L F|D|T R(K)</td><td>541.1604</td><td>1081.531</td><td>-0.2178</td></tr>
<tr><td>1150</td><td>XP_002273246.1</td><td>Probable Xaa-Pro aminopeptidase P</td><td>9(10)</td><td>19%</td><td>143.71</td><td>2</td><td>21.44</td><td>11.6</td><td>98.9</td><td>(K)L/T/E|V/S/A/S/D/K/L|E|S F\R(A)</td><td>791.5396</td><td>1581.812</td><td>0.26</td></tr>
<tr><td></td><td></td><td>(Vitis vinifera)</td><td></td><td></td><td></td><td>3</td><td>21.41</td><td>8.96</td><td>97.9</td><td>(K)N R P P/A E T N|P\V|I|I|Q|P V/E\F\A\G\R(S)</td><td>736.136</td><td>2205.177</td><td>1.216</td></tr>
<tr><td></td><td></td><td></td><td></td><td></td><td></td><td>3</td><td>18.09</td><td>6.76</td><td>92.3</td><td>(K)L/T|E|V|S/A/S D/K/L/E|S F R(A)</td><td>528.1403</td><td>1581.812</td><td>0.5945</td></tr>
<tr><td></td><td></td><td></td><td></td><td></td><td></td><td>2</td><td>17.94</td><td>10.53</td><td>93.8</td><td>(R)A F/V S/G F/T/G S/A/G L|A|L|I|T|M N E A R(L)</td><td>1057.6099</td><td>2113.075</td><td>1.1379</td></tr>
<tr><td></td><td></td><td></td><td></td><td></td><td></td><td>2</td><td>15.85</td><td>3.71</td><td>96.5</td><td>(K)V V/L/Q|Q|S P L/A\I\A K(A)</td><td>634.6173</td><td>1266.778</td><td>1.4493</td></tr>
<tr><td></td><td></td><td></td><td></td><td></td><td></td><td>2</td><td>15</td><td>6.66</td><td>100</td><td>(R)Y F/L/Q/A/S/Q|E|L|S|D Q W\K(L)</td><td>872.2052</td><td>1742.838</td><td>0.5647</td></tr>
<tr><td></td><td></td><td></td><td></td><td></td><td></td><td>2</td><td>14.45</td><td>8.12</td><td>94.8</td><td>(R)F/P/S/G T/A/G/H/T/L/D|I L\A R(V)</td><td>778.8088</td><td>1555.823</td><td>0.7876</td></tr>
<tr><td></td><td></td><td></td><td></td><td></td><td></td><td>2</td><td>13.23</td><td>9.57</td><td>96.6</td><td>(K)L V/Q/T/S/T/N/L|V|D\E V\W\K(N)</td><td>816.0516</td><td>1631.864</td><td>-0.768</td></tr>
<tr><td></td><td></td><td></td><td></td><td></td><td></td><td>2</td><td>12.39</td><td>2.57</td><td>94.6</td><td>(R)L/E|N|V/L|V\I\K(E)</td><td>464.5608</td><td>927.587</td><td>0.527</td></tr>
<tr><td></td><td></td><td></td><td></td><td></td><td></td><td>2</td><td>12</td><td>5.51</td><td>100</td><td>(K)L D/S D K V/V|L|Q|Q/S P L\A I\A K(A)</td><td>913.2095</td><td>1825.043</td><td>0.3688</td></tr>
<tr><td>1154</td><td>XP_002276438.1</td><td>5-methyltetrahydropteroyltriglutamate--homocysteine methyltransferase</td><td>5(5)</td><td>9%</td><td>88.32</td><td>3</td><td>19.07</td><td>7.83</td><td>94.5</td><td>(K)Y/G|A|G/I|G/P G/V/Y D|I|H/S P\R(I)</td><td>553.8352</td><td>1658.829</td><td>0.6625</td></tr>
<tr><td></td><td></td><td>(Vitis vinifera)</td><td></td><td></td><td></td><td>2</td><td>18.97</td><td>7.58</td><td>95</td><td>(K)A G I/T/V|I|Q|I|D|E\A|A\L\R(E)</td><td>735.7698</td><td>1469.832</td><td>0.7001</td></tr>
<tr><td></td><td></td><td></td><td></td><td></td><td></td><td>3</td><td>18.15</td><td>8.34</td><td>93.5</td><td>(K)A A/G A/S/S/I/Q|F|D/E|P T L/V/M D L D S H K(L)</td><td>778.2466</td><td>2332.113</td><td>0.6127</td></tr>
<tr><td></td><td></td><td></td><td></td><td></td><td></td><td>2</td><td>17.78</td><td>6.93</td><td>95.7</td><td>(K)G V/T|G/F|G|F D|L\V\R(G)</td><td>584.326</td><td>1167.616</td><td>0.029</td></tr>
</table>

						2	14.35	4.63	92.6	(K)Y/L|F|A|G V|V|D|G R(N)	549.0814	1096.579	0.577
		**5-methyltetrahydropteroyltriglutamate--homocysteine**											
1157	XP_002276438.1	**methyltransferase**	12(21)	21%	206.35	2	20.68	8.7	98.7	(K)L/P V/L|P T/T/T|I|G/S/F|P Q T M D\L\R(R)	1044.6548	2087.121	1.1818
		(Vitis vinifera)				2	19.58	11.56	96.4	(K)W F/D/T N Y/H|Y|I|V|P\E|L|G\P E V K(F)	1104.731	2207.081	1.3739
						2	19.25	11.64	97.4	(K)G M/L/T/G/P V|T/I|L|N/W|S|F V\R(N)	896.6765	1790.962	1.3835
						2	18.59	9.1	96.6	(K)G V/T|G/F|G|F|D|L V\R(G)	584.6912	1167.616	0.7594
						2	17.89	9.89	87.9	(K)Y G A/G/I|G/P/G V|Y|D|I/H|S\P R(I)	829.8245	1658.829	-0.1868
						2	17.85	7.96	91.7	(R)A G I/T/V|I|Q|I|D|E\A\A\L R(E)	735.2061	1469.832	-0.4273
						2	17.52	5.56	92.3	(R)A G I T/V|I|Q|I|D|E|A\A\L\R(E)	735.0836	1469.832	-0.6723
						2	17.07	7.97	95.9	(K)S F|S|L|L|S|L/I\D\K(I)	562.5791	1122.641	1.5104
						2	16.88	5.89	91.2	(K)F/A|L|E|S|F/W\D|K\K(S)	636.1552	1270.647	0.6565
						3	16.87	5.28	94	(K)A A/G A/S/S/I/Q|F/D/E|P T L/V/M/D L D S H K(L)	778.3887	2332.113	1.039
						3	16.51	7.49	90.8	(K)A A/G/A/S/S/I/Q|F/D E|P T L V/m D\L D S H K(L)	783.8884	2332.113	17.5381
						2	16.45	8.47	86.3	(R)A G/I/T/V|I|Q|I|D\E A A\L\R(E)	736.105	1469.832	1.3705
						3	16.22	6.01	90.7	(K)Y/G|A/G/I|G|P G V/Y/D/I H|S P R(I)	553.7549	1658.829	0.4216
						2	15.47	5.49	93.5	(R)K/Y|T/E|V|M|P A\L\K(N)	590.5204	1179.644	0.3893
						3	15.34	5.01	88.3	(K)Y/G|A/G|I|G/P/G V/Y D/I H\S P R(I)	553.813	1658.829	0.5959
						2	14.53	6.12	95.6	(K)Y/L|F|A|G V|V|D|G R(N)	549.3514	1096.579	1.117
						2	14.25	5.47	85.3	(K)Y/L|F|A|G V|V|D|G R(N)	548.7936	1096.579	0.0014
						2	14.04	4.61	80.9	(R)A G/I T/V|I|Q/I|D E|A\A L\R(E)	735.824	1469.832	0.8085
						2	13.99	4.21	80.2	(K)L P V/L|P T/T/T/I|G/S/F|P Q T m D L R(R)	1052.7563	2087.121	17.3848
						3	12.89	3.01	77.7	(K)W/F/D T N Y H Y/I/V|P E/L\G\P\E V K(F)	736.6184	2207.081	0.7599
						2	11.69	3.72	98.4	(K)S/W|L|A|F|A/A\Q\K(V)	511.4824	1021.547	0.411
		**5-methyltetrahydropteroyltriglutamate--homocysteine**											
1159	XP_002276438.1	**methyltransferase**	6(9)	12%	93.18	2	21.41	10.36	98.4	(R)A G I/T/V|I|Q|I|D|E|A|A|L\R(E)	735.7063	1469.832	0.5731
		(Vitis vinifera)				2	18.61	9.51	94.2	(R)A G/I T/V|I|Q|I|D|E|A/A\L\R(E)	735.4664	1469.832	0.0933
						3	16.38	6.91	89.8	(K)A/A/G A/S/S|I|Q|F/D/E|P T L/V/M D L D S H K(L)	778.2689	2332.113	0.6796
						3	16.11	6.69	90.1	(K)Y/G|A|G|I|G/P/G V/Y D\I H|S P R(I)	554.0239	1658.829	1.2286
						3	15.77	5	84.3	(K)A/A/G/A/S/S/I/Q|F/D/E|P T L/V M/D L D S H K(L)	778.2317	2332.113	0.568
						2	14.46	6.29	91.9	(K)Y/L|F|A|G V|V|D|G R(N)	548.7816	1096.579	-0.0226
						2	13.54	5.1	95.7	(K)G V/T/G/F|G\F|D L\V\R(G)	584.3188	1167.616	0.0146
						3	12.62	3.05	77.1	(K)A A/G/A/S|S/I/Q/F/D E P T/L\V m/D L D S H K(L)	783.5674	2332.113	16.5751
						2	11.28	4.63	89.4	(K)L P V L/P T/T/T/I|G/S/F|P Q T M D\L R(R)	1044.8481	2087.121	1.5684
		**NADH dehydrogenase [ubiquinone] iron-sulfur protein 1,**											
1197	XP_002270157.1	**mitochondrial**	7(8)	14%	107.53	2	19.07	10.64	96.5	(R)F A/T E/V|A|G/V|Q|D/L|G M\L\G R(G)	833.1045	1663.847	1.3545
		(Vitis vinifera)				2	18.35	6.5	96.1	(R)S G/Y|L|L|N/S N/I|A|G L\E\K(A)	739.8472	1478.785	-0.0978
						2	18	9.53	90.2	(R)E/A/A/T/F/S/T/S/L|K/P E|L S E\K(M)	869.6301	1737.891	0.3624
						2	15.25	5.69	100	(R)G/D/K/D/A|I|F|A|A|V\E\T I A K(L)	775.5825	1548.827	1.3309
						3	14.16	6.91	98.1	(K)V/P/D/D/A/F|V/V|Y Q G H/H\G D Q S V Y R(A)	763.9861	2289.068	0.8754
						2	11.57	4.17	91.1	(K)G T E T I/D/V|T/D|A\V|G\S N I R(I)	824.5432	1647.818	0.2607
						2	11.13	3.01	92.9	(K)A D V F L|L V|G|T|Q|P R(V)	658.6219	1315.737	0.4997
						2	10.42	5.54	92.2	(K)G T E/T I/D/V|T D|A/V|G/S N\I\R(I)	824.624	1647.818	0.4223
1198	XP_002283532.2	**Heat shock cognate 70 kDa protein 2**	14(20)	28%	229.33	2	21.29	13.37	96.6	(K)N Q/V|A/m|N|P I|N/T|V|F\D\A K(R)	839.7566	1661.832	16.6744
		(Vitis vinifera)				2	21.24	12.51	96.9	(K)V Q|Q|L|L|Q|D|F|F|N\G\K(E)	718.427	1436.753	-0.9065
						2	19.05	11.56	95	(K)V Q/Q|L|L|Q|D|F/F|N\G\K(E)	719.4788	1436.753	1.1971
						2	18.87	10.64	98.5	(K)Q F\A/A/E|E/I S|S/M|V|L I\K(M)	783.5415	1565.824	0.2514
						2	18.31	13.11	95.9	(K)S T/I/H/D|V|V|L|V|G|G S T R(I)	720.855	1440.781	-0.0778
						2	17.9	9.48	93.1	(K)A T/A/G D/T/H|L|G|G/E|D\F\D|N\R(M)	838.72	1675.731	0.7021
						2	17.48	9.04	95.5	(R)T T/P/S/Y/V|A|F|T|D\T E R(L)	744.4446	1487.701	0.1807
						2	17.15	8.22	93.8	(R)F/S|D|A|S|V\Q|S D|I\K(H)	599.0361	1196.579	0.4856
						2	16.93	6.57	94.6	(K)D A G\V|I|A|G|L|N V|M\R(I)	608.7395	1215.651	0.8203
						2	15.55	5.98	83.2	(K)N Q/V|A/M|N|P I|N/T V|F D\A K(R)	831.2678	1661.832	-0.3032
						2	15.48	4.7	87.6	(R)F E|E|L|N|M|D|L|F R(K)	657.8334	1313.619	1.0401
						2	15.31	7.99	88.8	(K)V Q/Q|L|L|Q|D/F|F|N G K(E)	719.4611	1436.753	1.1617
						2	15.07	7.38	80.1	(K)N Q/V|A|M|N|P I N/T V|F D\A\K(R)	831.2139	1661.832	-0.411
						2	14.98	4.64	83.3	(R)I I/N E|P T/A|A/A\I/A|Y G\L\D K(K)	830.4763	1659.895	0.0501
						2	13.79	3.53	92.8	(K)N A V|V|T|V|P A Y/F N/D S Q R(Q)	840.8113	1680.834	-0.2187
						2	13.01	6.32	83.7	(K)N A/L|E/N/Y|A|Y/N\M R(N)	679.4409	1358.616	-0.7412
						2	12.35	4.38	76.3	(R)E I/A/E/A|Y/L|G|S\T V K(N)	641.0032	1280.673	0.3259
						2	11.15	4.83	85.4	(K)N A/L|E/N/Y\A|Y|N\m R(N)	688.0262	1358.616	16.4294
						2	10.55	2.65	84.4	(K)E L/E/S/I|C/N|P I/I\A K(M)	693.8293	1386.73	-0.0784
						2	10.11	5.65	94.5	(K)V Q/Q/L/L|Q/D F|F\N G K(E)	719.2981	1436.753	0.8357
1231	XP_002283532.2	**Heat shock cognate 70 kDa protein 2**	22(57)	45%	385.69	2	21.88	12.61	98.5	(R)L S/K/E/E|I|E|N|M|V|Q\E\A\E\K(Y)	889.2634	1776.868	0.6511
		(Vitis vinifera)				2	21	12.95	100	(K)S I/N/P D E A V/A/Y/G/A|A/V|Q/A/A/I|L/S G E G N E K(V)	1287.5889	2574.268	-0.0977
						2	20.94	10.15	99.1	(K)V/Q/Q|L|L|Q|D|F|F|N\G K(E)	719.302	1436.753	0.8435
						2	20.85	8.45	95	(R)I I/N/E|P/T/A|A|A/I|A|Y|G\L D K(K)	831.2004	1659.895	1.4983
						3	20.47	8.73	98	(K)A/T|A/G/D/T H/L/G G E|D/F|D|N R(M)	559.8193	1675.731	1.7127

						2	20.24	12.35	97.5	(K)S/T/I/H|D|V|V|L|V|G|G S T R(I)	721.0845	1440.781	0.3812
						2	19.9	8.91	96.2	(R)I I/N/E|P/T/A|A|A/I|A|Y\G L\D K(K)	830.525	1659.895	0.1475
						2	19.8	10.06	94.9	(K)E/Q V/F S T/Y/S/D/N Q|P/G V|L|I|Q|V/Y E G E R(T)	1329.3674	2658.268	-0.5407
						2	18.55	5.83	96.7	(R)F/E|E|L|N|M|D|L|F\R(K)	657.3921	1313.619	0.1575
						2	18.5	9.82	88.3	(K)N Q/V|A|M|N|P\I|N T|V|F\D A K(R)	832.2167	1661.832	1.5946
						2	18.47	8.54	95.3	(R)V/E|I|I|A|N|D|Q|G N R(T)	615.0503	1228.628	0.4653
						3	18.33	8.76	94.7	(R)M/V|N/H|F|V|Q E|F\K(R)	427.1945	1278.63	0.939
						2	18.12	10.09	95.1	(K)V Q/Q|L|L|Q|D|F|F|N\G K(E)	719.4688	1436.753	1.1771
						2	18	10.5	100	(K)A T/A G D/T/H/L|G/G/E|D|F\D|N R(M)	839.0222	1675.731	1.3065
						2	17.91	9.5	93.2	(K)V Q|Q|L|L|Q|D/F|F\N\G\K(E)	719.5142	1436.753	1.2679
						2	17.81	10.65	85.4	(K)N Q|V|A|M|N|P I|N/T V/F\D\A K(R)	831.7258	1661.832	0.6128
						2	17.79	8.08	95.8	(K)D A|G\V|I|A|G\L|N V|M\R(I)	608.7167	1215.651	0.7747
						2	17.63	8.38	96	(R)A R/F/E|E|L|N|M/D|L\F\R(K)	771.6821	1540.758	1.5992
						2	17.33	8.63	91.2	(K)E/Q V/F S T/Y/S D N Q|P/G V|L|I|Q/V/Y\E G E R(T)	1330.4365	2658.268	1.5975
						2	17.32	9.54	92.3	(K)G E/D/K/Q|F|A|A|E/E|I\S S M V|L\I K(M)	998.7543	1995.01	1.491
						2	17.28	7.53	95.3	(R)V E|I|I|A|N|D|Q|G N R(T)	614.7107	1228.628	-0.2139
						2	17.12	9.86	94.7	(K)V/Q/Q|L/L|Q|D|F|F\N\G K(E)	719.1552	1436.753	0.5499
						2	16.92	7.76	87.9	(K)V Q|Q|L|L|Q|D\F|F\N\G K(E)	719.1827	1436.753	0.6049
						2	16.81	6.98	85.5	(K)N Q/V|A|M|N|P I/N|T\V|F D A K(R)	831.9746	1661.832	1.1104
						3	16.77	5.44	89.3	(K)A/T|A/G/D|T H/L/G G E/D F\D|N\R(M)	559.6167	1675.731	1.1049
						2	16.61	5.73	91.1	(R)V E|I|I|A|N|D|Q|G N\R(T)	614.7542	1228.628	-0.1269
						3	16.52	4.82	93.3	(R)A R F E E\L|N|M|D|L|F R(K)	514.916	1540.758	1.9758
						2	16.03	6.72	87	(K)Q/F/A/A/E|E|I S/S M|V|L\I\K(M)	783.6714	1565.824	0.5112
						2	15.78	6.31	91.9	(K)Q F|A|A/E/E|I S/S M/V\L I\K(M)	783.4736	1565.824	0.1156
						2	15.35	7.13	86.7	(K)N/A/L|E|N|Y\A|Y|N m R(N)	687.8093	1358.616	15.9956
						2	15.31	3.83	86.5	(R)I I/N E/P\T/A/A/A|I/A/Y/G L D\K K(A)	894.7069	1787.99	0.4163
						2	15.22	6.65	95.7	(K)N A/L|E|N|Y|A|Y|N M\R(N)	680.0513	1358.616	0.4796
						2	14.93	7.34	94.3	(R)F/S|D|A|S/V Q|S D\I K(H)	598.6909	1196.579	-0.2048
						3	14.74	3.04	99.3	(R)I/I|N|E|P T/A/A/A I/A Y G L D K K(A)	596.8525	1787.99	0.5528
						2	14.67	5.75	87.9	(K)Q F/A/A/E/E/I S|S/M V|L\I K(M)	783.5975	1565.824	0.3634
						2	14.63	6	98	(R)F E|E|L|N/M|D|L\F R(K)	657.7067	1313.619	0.7867
						3	14.17	8.65	78.6	(K)M Y Q G A/G/G/P D A\G\A\G A M|D D D/G|P S A/G G\S G A G\P K(I)	874.928	2622.083	0.6863
						2	13.6	4.23	87.8	(R)A R/F E|E|L/N\M\D|L F\R(K)	771.04	1540.758	0.315
						2	13.41	5.64	87	(K)Q F A/A/E/E/I S/S/M/V L\I\K(M)	783.0144	1565.824	-0.8028
						2	13.38	3.23	70.1	(K)V Q/Q|L|L|Q|D\F F\N\G K(E)	719.4861	1436.753	1.2117
						2	13.27	8.15	96	(K)N Q/V|A/m/N|P\I N T/V/F\D\A K(R)	839.4126	1661.832	15.9864
						2	13.24	4.5	95	(R)V E|I|I|A|N/D|Q/G N R(T)	614.9941	1228.628	0.3529
						2	13.04	5.49	88.6	(K)N A/L|E|N|Y|A|Y/N M R(N)	680.4565	1358.616	1.29
						2	12.96	3.76	98.1	(K)N A V|V|T|V|P A/Y/F N D S Q R(Q)	840.8701	1680.834	-0.1011
						2	12.72	2.62	88.6	(R)T T/P S/Y/V|A/F/T/D\T E R(L)	744.5745	1487.701	0.4405
						3	12.45	3.05	97.2	(K)V/V|P/G/P G D/K/P M/I/T V T Y K(G)	568.1426	1701.924	0.4888
						2	12.4	3.05	87.5	(K)N A/V|V/T|V|P A Y/F N D S Q R(Q)	841.0542	1680.834	0.2671
						3	12.32	4.09	75.4	(R)I I N/E/P T/A A\A|I|A|Y|G\L D K(K)	554.3201	1659.895	1.0505
						2	12	4.04	100	(R)I I/N/E|P T/A A/A|I|A/Y\G L D K(K)	830.8386	1659.895	0.7747
						2	11.94	4.28	82.1	(R)F/S|D/A|S|V Q|S D I\K(H)	599.0388	1196.579	0.491
						3	11.76	3.13	92.9	(K)S T I H D V\V|L|V G|G\S T R(I)	481.0197	1440.781	0.264
						2	11.46	2.74	93.6	(R)V E|I|I|A|N/D\Q G N R(T)	615.7151	1228.628	1.7949
						2	11.39	4.2	96.3	(R)I I/N E|P T A|A/A|I/A Y\G L D K(K)	830.5566	1659.895	0.2107
						2	10.86	2.41	81	(K)N A/L|E|N|Y|A/Y/N M R(N)	680.3992	1358.616	1.1754
						2	10.59	2.89	85.4	(R)V E|I|I|A|N/D Q/G N R(T)	615.2117	1228.628	0.7881
						2	10.14	3.95	79	(K)V Q/Q/L|L/Q/D F|F|N G K(E)	719.4917	1436.753	1.2229
						2	10.05	3.77	82.3	(K)Q/F/A/A/E/E/I S/S M/V\L I K(M)	783.5887	1565.824	0.3458
1237	XP_002283532.2	**Heat shock cognate 70 kDa protein 2** *(Vitis vinifera)*	15(30)	28%	244.22	2	20.37	9.36	95.8	(R)I I/N/E|P/T/A|A/A|I|A|Y|G L D K(K)	830.5588	1659.895	0.2151
						2	19.34	9.67	97.8	(K)V Q/Q|L|L|Q|D|F|F\N\G K(E)	719.7346	1436.753	1.7087
						2	18.57	4.65	96.9	(R)F/E|E|L|N|M|D|L|F\R(K)	657.8816	1313.619	1.1365
						2	18.41	8.37	85.4	(K)V Q/Q|L|L|Q|D|F/F|N\G K(E)	719.8469	1436.753	1.9333
						2	18.31	10.02	96.7	(R)F/S|D|A|S/V\Q|S|D|I\K(H)	599.0775	1196.579	0.5684
						2	18.04	6.85	89.7	(K)N Q|V|A|M|N|P I/N/T V|F\D\A K(R)	830.8335	1661.832	-1.1718
						2	17.79	9.51	91.6	(K)V Q/Q|L|L|Q|D|F|F|N\G K(E)	719.7773	1436.753	1.7941
						2	17.78	10.36	97.3	(K)V Q/Q|L|L|Q|D|F|F\N\G K(E)	719.4956	1436.753	1.2307
						2	17.61	8.95	89.5	(R)I/I/N/E/P/T/A/A/A/I|A/Y|G L D\K K(A)	895.1587	1787.99	1.3199
						2	17.61	7.72	87.2	(R)I I/N|E|P T/A|A/A|I|A|Y\G L D K(K)	831.4155	1659.895	1.9285
						2	17.08	6.08	92.5	(R)E I/A/E|A|Y|L|G|S A V\K(N)	626.1995	1250.663	0.729
						2	16.34	6.71	93.4	(K)N A/L|E|N/Y|A|Y/N/M R(N)	679.9075	1358.616	0.192
						2	15.81	5.63	91.6	(K)N A V|V|T|V|P/A/Y/F/N D S Q R(Q)	840.6978	1680.834	-0.4457
						2	15.74	9.85	90.5	(K)S T/I/H|D|V|V|L|V|G/G S T R(I)	721.2788	1440.781	0.7698
						2	15.68	5.73	91.3	(K)D A G\V|I|A|G|L|N V\M\R(I)	608.3256	1215.651	-0.0075

<table>
<tr><th></th><th></th><th></th><th></th><th></th><th></th><th></th><th></th><th></th><th></th><th></th><th></th><th></th><th></th></tr>
<tr><td></td><td></td><td></td><td></td><td></td><td></td><td>2</td><td>15.27</td><td>6.17</td><td>89.1</td><td>(R)E I/A/E/A|Y/L|G|S\T V K(N)</td><td>641.1031</td><td>1280.673</td><td>0.5257</td></tr>
<tr><td></td><td></td><td></td><td></td><td></td><td></td><td>2</td><td>14.64</td><td>6.45</td><td>97.1</td><td>(R)V E|I|I|A|N|D|Q/G N R(T)</td><td>615.1968</td><td>1228.628</td><td>0.7583</td></tr>
<tr><td></td><td></td><td></td><td></td><td></td><td></td><td>2</td><td>14.42</td><td>3.71</td><td>92.1</td><td>(R)T T/P/S/Y/V|A/F/T|D T E R(L)</td><td>744.7847</td><td>1487.701</td><td>0.8609</td></tr>
<tr><td></td><td></td><td></td><td></td><td></td><td></td><td>2</td><td>14.29</td><td>3.19</td><td>78.3</td><td>(R)I I/N/E/P T/A|A/A|I/A/Y\G/L D K(K)</td><td>831.0903</td><td>1659.895</td><td>1.2781</td></tr>
<tr><td></td><td></td><td></td><td></td><td></td><td></td><td>2</td><td>14.08</td><td>5.6</td><td>92.4</td><td>(R)V E|I|I|A|N/D|Q/G N R(T)</td><td>615.3789</td><td>1228.628</td><td>1.1225</td></tr>
<tr><td></td><td></td><td></td><td></td><td></td><td></td><td>2</td><td>14.04</td><td>6.76</td><td>92.7</td><td>(R)F/S|D/S/S|V\Q|S D|I\K(L)</td><td>606.708</td><td>1212.574</td><td>-0.1655</td></tr>
<tr><td></td><td></td><td></td><td></td><td></td><td></td><td>3</td><td>13.78</td><td>3.87</td><td>87.4</td><td>(K)A T/A/G/D/T/H/L/G G E/D/F/D/N\R(M)</td><td>559.8528</td><td>1675.731</td><td>1.8132</td></tr>
<tr><td></td><td></td><td></td><td></td><td></td><td></td><td>2</td><td>13.57</td><td>5.76</td><td>80.4</td><td>(K)V Q/Q|L|L|Q|D\F|F|N G K(E)</td><td>718.6392</td><td>1436.753</td><td>-0.4821</td></tr>
<tr><td></td><td></td><td></td><td></td><td></td><td></td><td>3</td><td>13.51</td><td>2.18</td><td>85.2</td><td>(K)S T I H/D V\V|L|V G|G|S\T R(I)</td><td>480.897</td><td>1440.781</td><td>-0.1041</td></tr>
<tr><td></td><td></td><td></td><td></td><td></td><td></td><td>2</td><td>13.46</td><td>5.08</td><td>84.8</td><td>(K)D A G\V|I|A|G|L|N\V\M R(I)</td><td>608.7751</td><td>1215.651</td><td>0.8915</td></tr>
<tr><td></td><td></td><td></td><td></td><td></td><td></td><td>2</td><td>13.17</td><td>4.83</td><td>79.2</td><td>(K)N/Q/V|A|M/N|P\I N T V F D\A\K(R)</td><td>830.615</td><td>1661.832</td><td>-1.6088</td></tr>
<tr><td></td><td></td><td></td><td></td><td></td><td></td><td>2</td><td>12.84</td><td>5.48</td><td>80.7</td><td>(K)V Q/Q/L|L|Q/D\F\F N\G K(E)</td><td>719.5769</td><td>1436.753</td><td>1.3933</td></tr>
<tr><td></td><td></td><td></td><td></td><td></td><td></td><td>2</td><td>12.52</td><td>4.96</td><td>70.4</td><td>(K)N Q/V|A|M|N|P I N\T V F/D A K(R)</td><td>831.7343</td><td>1661.832</td><td>0.6298</td></tr>
<tr><td></td><td></td><td></td><td></td><td></td><td></td><td>2</td><td>11.21</td><td>3.67</td><td>86.1</td><td>(R)I T/P S W|V|A F|T\D S E R(L)</td><td>755.1074</td><td>1508.738</td><td>0.4695</td></tr>
<tr><td></td><td></td><td></td><td></td><td></td><td></td><td>2</td><td>10.58</td><td>2.74</td><td>72</td><td>(K)N Q/V|A/M|N|P I/N T V F D\A K(R)</td><td>831.6361</td><td>1661.832</td><td>0.4334</td></tr>
<tr><td></td><td></td><td></td><td></td><td></td><td></td><td>2</td><td>10.3</td><td>3.44</td><td>85.8</td><td>(K)A T/A G D/T H|L|G/G E D|F D|N R(M)</td><td>838.8533</td><td>1675.731</td><td>0.9687</td></tr>
<tr><td></td><td></td><td></td><td></td><td></td><td></td><td>2</td><td>10.3</td><td>3.95</td><td>83.7</td><td>(K)Q F A/A/E/E/I S/S M\V/L I\K(M)</td><td>783.782</td><td>1565.824</td><td>0.7324</td></tr>
<tr><td></td><td></td><td></td><td></td><td></td><td></td><td>2</td><td>10.25</td><td>4.5</td><td>100</td><td>(K)D A G\V|I|A|G L|N V\M\R(I)</td><td>608.8401</td><td>1215.651</td><td>1.0215</td></tr>
<tr><td>1238</td><td>XP_002266494.2</td><td>Transketolase, chloroplastic</td><td>6(6)</td><td>10%</td><td>90.42</td><td>3</td><td>20.82</td><td>6.78</td><td>97.5</td><td>(R)H/I P D G/A\A|L|E|A|E|W|N\A\K(F)</td><td>541.2819</td><td>1621.797</td><td>0.0343</td></tr>
<tr><td></td><td></td><td>(Vitis vinifera)</td><td></td><td></td><td></td><td>2</td><td>19.23</td><td>10.18</td><td>95.9</td><td>(K)S I/I|T|G|E|L|P A|G W|D\K(A)</td><td>694.0715</td><td>1386.726</td><td>0.4094</td></tr>
<tr><td></td><td></td><td></td><td></td><td></td><td></td><td>2</td><td>15.53</td><td>4.93</td><td>90.7</td><td>(K)E S/V|L|P/A/A/V/T A R(V)</td><td>557.4888</td><td>1113.626</td><td>0.3441</td></tr>
<tr><td></td><td></td><td></td><td></td><td></td><td></td><td>2</td><td>12.17</td><td>4.18</td><td>91.2</td><td>(K)V T|T/T/I/G/Y/G|S/P N K(A)</td><td>620.0098</td><td>1237.642</td><td>1.37</td></tr>
<tr><td></td><td></td><td></td><td></td><td></td><td></td><td>2</td><td>11.64</td><td>2.45</td><td>89.6</td><td>(R)Y/N|P S/N|P Y/W|F/N R(D)</td><td>729.7427</td><td>1457.66</td><td>0.8184</td></tr>
<tr><td></td><td></td><td></td><td></td><td></td><td></td><td>3</td><td>11.03</td><td>2.28</td><td>92.6</td><td>(R)Q K/L P/Q/L|P/G T S I/A G V\E K(G)</td><td>555.748</td><td>1665.953</td><td>-0.7239</td></tr>
<tr><td>1315</td><td>P51615.1</td><td>NADP-dependent malic enzyme (NADP-ME)</td><td>9(15)</td><td>18%</td><td>138.95</td><td>3</td><td>18.68</td><td>10.47</td><td>91.8</td><td>(R)V H D/E m\L\L|A|A|S/E|A\L|A\R(Q)</td><td>547.5634</td><td>1625.832</td><td>14.8441</td></tr>
<tr><td></td><td></td><td>(Vitis vinifera)</td><td></td><td></td><td></td><td>2</td><td>18.44</td><td>9.74</td><td>93.9</td><td>(R)V/H/D/E/M|L|L|A|A|S|E A\L\A\R(Q)</td><td>813.5029</td><td>1625.832</td><td>0.167</td></tr>
<tr><td></td><td></td><td></td><td></td><td></td><td></td><td>3</td><td>17.7</td><td>7.1</td><td>92.7</td><td>(K)V I/K P T\V|L|I|G|S/S/G V\G K(A)</td><td>485.7777</td><td>1454.894</td><td>0.4245</td></tr>
<tr><td></td><td></td><td></td><td></td><td></td><td></td><td>3</td><td>17.06</td><td>6.73</td><td>94.8</td><td>(R)V H D E M L\L\A|A|S E|A|L|A R(Q)</td><td>542.6453</td><td>1625.832</td><td>0.0898</td></tr>
<tr><td></td><td></td><td></td><td></td><td></td><td></td><td>2</td><td>16.48</td><td>4.48</td><td>90.6</td><td>(K)L/L|A/N/E|F/Y|I|G L\K(Q)</td><td>640.8643</td><td>1280.725</td><td>-0.0036</td></tr>
<tr><td></td><td></td><td></td><td></td><td></td><td></td><td>2</td><td>15.64</td><td>9.98</td><td>98.1</td><td>(R)I/Q|V|I|V|V|T|D\G E\R(I)</td><td>614.9822</td><td>1228.69</td><td>0.2675</td></tr>
<tr><td></td><td></td><td></td><td></td><td></td><td></td><td>2</td><td>15.55</td><td>3.49</td><td>94</td><td>(R)L/L|G|G/T/L|A/D|H\K(F)</td><td>513.1555</td><td>1024.579</td><td>0.7252</td></tr>
<tr><td></td><td></td><td></td><td></td><td></td><td></td><td>2</td><td>14.81</td><td>8.03</td><td>95</td><td>(R)A I/F A S|G/S|P F/D|P V\E|Y\N G K(T)</td><td>900.2355</td><td>1798.865</td><td>0.5991</td></tr>
<tr><td></td><td></td><td></td><td></td><td></td><td></td><td>2</td><td>14.4</td><td>4.78</td><td>90.4</td><td>(K)L/L|A/N/E|F/Y/I/G L\K(Q)</td><td>640.9167</td><td>1280.725</td><td>0.1012</td></tr>
<tr><td></td><td></td><td></td><td></td><td></td><td></td><td>2</td><td>14.26</td><td>3.39</td><td>82.5</td><td>(K)L/L|A/N/E|F/Y\I|G L\K(Q)</td><td>641.0874</td><td>1280.725</td><td>0.4426</td></tr>
<tr><td></td><td></td><td></td><td></td><td></td><td></td><td>2</td><td>13.87</td><td>6.82</td><td>96.9</td><td>(K)A Y|E|L|G|L|A|T\R(L)</td><td>497.3768</td><td>993.536</td><td>0.21</td></tr>
<tr><td></td><td></td><td></td><td></td><td></td><td></td><td>2</td><td>13.67</td><td>3.66</td><td>93.6</td><td>(R)D G A|S|V|L/D/L|D|P\K(A)</td><td>565.5248</td><td>1129.574</td><td>0.4688</td></tr>
<tr><td></td><td></td><td></td><td></td><td></td><td></td><td>2</td><td>12.55</td><td>4.92</td><td>89.7</td><td>(K)Y/M|A|M|M/D\L|Q E\R(N)</td><td>644.2799</td><td>1287.553</td><td>-0.0005</td></tr>
<tr><td></td><td></td><td></td><td></td><td></td><td></td><td>2</td><td>10.43</td><td>3.63</td><td>96.5</td><td>(K)V I/K/P/T/V|L/I|G S S\G V G K(A)</td><td>728.2876</td><td>1454.894</td><td>0.6738</td></tr>
<tr><td></td><td></td><td></td><td></td><td></td><td></td><td>3</td><td>10.41</td><td>2.96</td><td>82</td><td>(R)V H D E M L|L A|A|S/E|A|L\A\R(Q)</td><td>542.5546</td><td>1625.832</td><td>-0.1823</td></tr>
<tr><td>1325</td><td>P51615.1</td><td>NADP-dependent malic enzyme (NADP-ME)</td><td>31(24)</td><td>27%</td><td>213.82</td><td>2</td><td>22.17</td><td>14.78</td><td>99.1</td><td>(R)V/H/D/E/M|L|L|A|A|S|E\A\L\A\R(Q)</td><td>813.8181</td><td>1625.832</td><td>0.7974</td></tr>
<tr><td></td><td></td><td>(Vitis vinifera)</td><td></td><td></td><td></td><td>2</td><td>21.46</td><td>7.77</td><td>98.1</td><td>(K)V/I/K/P/T|V|L|I|G|S S\G V\G\K(A)</td><td>727.876</td><td>1454.894</td><td>-0.1494</td></tr>
<tr><td></td><td></td><td></td><td></td><td></td><td></td><td>3</td><td>19.21</td><td>9.36</td><td>90.9</td><td>(R)V H D/E m L\L|A|A|S/E|A|L|A\R(Q)</td><td>547.978</td><td>1625.832</td><td>16.0879</td></tr>
<tr><td></td><td></td><td></td><td></td><td></td><td></td><td>3</td><td>18.8</td><td>6.19</td><td>94.6</td><td>(K)V I|K P T\V|L|I|G|S S/G V\G K(A)</td><td>486.2454</td><td>1454.894</td><td>1.8276</td></tr>
<tr><td></td><td></td><td></td><td></td><td></td><td></td><td>3</td><td>17.89</td><td>6.04</td><td>93.1</td><td>(R)V H D E M L\L|A|A|S/E|A|L|A R(Q)</td><td>542.8765</td><td>1625.832</td><td>0.7834</td></tr>
<tr><td></td><td></td><td></td><td></td><td></td><td></td><td>2</td><td>17.79</td><td>4.6</td><td>97</td><td>(K)A/Y|E|L|G|L|A|T\R(L)</td><td>497.3696</td><td>993.536</td><td>0.1956</td></tr>
<tr><td></td><td></td><td></td><td></td><td></td><td></td><td>2</td><td>17.16</td><td>9.2</td><td>97.6</td><td>(R)A I F/A/S/G/S|P F/D|P V|E|Y\N\G\K(T)</td><td>900.3398</td><td>1798.865</td><td>0.8077</td></tr>
<tr><td></td><td></td><td></td><td></td><td></td><td></td><td>2</td><td>17.08</td><td>6.18</td><td>96.7</td><td>(R)D G|A|S|V|L/D|L|D|P K(A)</td><td>565.5287</td><td>1129.574</td><td>0.4766</td></tr>
<tr><td></td><td></td><td></td><td></td><td></td><td></td><td>2</td><td>16.82</td><td>11.18</td><td>92.2</td><td>(R)I/Q|V|I|V|V|T|D|G E R(I)</td><td>614.8583</td><td>1228.69</td><td>0.0197</td></tr>
<tr><td></td><td></td><td></td><td></td><td></td><td></td><td>2</td><td>16.41</td><td>11.91</td><td>84.6</td><td>(K)V L/I/Q/F/E/D/F/A/N/H|N A F\D|L\L\A K(Y)</td><td>1103.3242</td><td>2205.134</td><td>0.5072</td></tr>
<tr><td></td><td></td><td></td><td></td><td></td><td></td><td>2</td><td>16.04</td><td>5.14</td><td>89.5</td><td>(K)Y/M|A|M|M/D|L|Q|E\R(N)</td><td>644.2876</td><td>1287.553</td><td>0.0149</td></tr>
<tr><td></td><td></td><td></td><td></td><td></td><td></td><td>2</td><td>15.58</td><td>3.67</td><td>94.9</td><td>(R)R/P\Q/G L|Y\I|S|L\K(E)</td><td>588.1538</td><td>1174.694</td><td>0.6061</td></tr>
<tr><td></td><td></td><td></td><td></td><td></td><td></td><td>2</td><td>15.36</td><td>3.74</td><td>86</td><td>(R)R/P Q\G L|Y\I|S\L\K(E)</td><td>588.5027</td><td>1174.694</td><td>1.3039</td></tr>
<tr><td></td><td></td><td></td><td></td><td></td><td></td><td>2</td><td>15.02</td><td>10.44</td><td>95.4</td><td>(K)I/S/A/H|I|A|A|N V\A\A\K(A)</td><td>584.2168</td><td>1165.669</td><td>1.7575</td></tr>
<tr><td></td><td></td><td></td><td></td><td></td><td></td><td>2</td><td>14.03</td><td>4.06</td><td>91.5</td><td>(K)G/L/I|Y|P|P F\S/N/I R(K)</td><td>638.8235</td><td>1276.705</td><td>-0.0651</td></tr>
<tr><td></td><td></td><td></td><td></td><td></td><td></td><td>2</td><td>13.73</td><td>5.63</td><td>89.7</td><td>(K)E Y|S E/F/L/Q|E/F M|S A V\K(Q)</td><td>854.6379</td><td>1707.794</td><td>0.4751</td></tr>
<tr><td></td><td></td><td></td><td></td><td></td><td></td><td>2</td><td>13.44</td><td>4.47</td><td>97.4</td><td>(K)G L/I|Y|P|P F\S/N/I R(K)</td><td>638.6707</td><td>1276.705</td><td>-0.3707</td></tr>
<tr><td></td><td></td><td></td><td></td><td></td><td></td><td>2</td><td>13.39</td><td>6.37</td><td>97.2</td><td>(R)A I/F A S|G/S|P F D|P\V\E|Y N G K(T)</td><td>900.58</td><td>1798.865</td><td>1.2881</td></tr>
<tr><td></td><td></td><td></td><td></td><td></td><td></td><td>2</td><td>13.13</td><td>6.28</td><td>95.2</td><td>(R)V/H/D E m|L|L|A/A\S\E A L A R(Q)</td><td>821.6584</td><td>1625.832</td><td>16.478</td></tr>
<tr><td></td><td></td><td></td><td></td><td></td><td></td><td>3</td><td>12.53</td><td>3.31</td><td>94.6</td><td>(K)V/L/I|Q|F|E D/F A N H N A F/D L L A K(Y)</td><td>736.0255</td><td>2205.134</td><td>0.9281</td></tr>
<tr><td></td><td></td><td></td><td></td><td></td><td></td><td>3</td><td>11.71</td><td>5.64</td><td>88.1</td><td>(R)V H D E M L\L\A|A|S/E|A|L\A R(Q)</td><td>542.7894</td><td>1625.832</td><td>0.5221</td></tr>
<tr><td></td><td></td><td></td><td></td><td></td><td></td><td>2</td><td>11.49</td><td>3.22</td><td>83</td><td>(R)I/Q/V|I|V|V|T|D G E R(I)</td><td>615.1519</td><td>1228.69</td><td>0.6069</td></tr>
<tr><td></td><td></td><td></td><td></td><td></td><td></td><td>2</td><td>10.53</td><td>2.03</td><td>83.2</td><td>(R)L/P/Q|P/E/N L\V\K(Y)</td><td>519.2489</td><td>1037.599</td><td>-0.1084</td></tr>
<tr><td></td><td></td><td></td><td></td><td></td><td></td><td>2</td><td>10.39</td><td>4.54</td><td>85.7</td><td>(R)V/H/D/E m/L|L|A|A\S E A L A R(Q)</td><td>821.791</td><td>1625.832</td><td>16.7432</td></tr>
<tr><td>1326</td><td>XP_002284729.1</td><td>Phosphoglucomutase, cytoplasmic</td><td>7(7)</td><td>15%</td><td>106.04</td><td>3</td><td>20.26</td><td>10.42</td><td>96.8</td><td>(K)A/D|E|F|E Y|K/D|P/V/D|G S I\S K(H)</td><td>600.9583</td><td>1799.833</td><td>1.0269</td></tr>
<tr><td></td><td></td><td>(Vitis vinifera)</td><td></td><td></td><td></td><td>2</td><td>17.12</td><td>6.32</td><td>91.9</td><td>(R)Y/D|Y|E|N|V|D|A/G A\A K(E)</td><td>658.6802</td><td>1315.58</td><td>0.773</td></tr>
<tr><td></td><td></td><td></td><td></td><td></td><td></td><td>2</td><td>17.11</td><td>5.85</td><td>94.6</td><td>(K)L V|T|V|E|E|I|V\R(N)</td><td>529.3953</td><td>1057.625</td><td>0.1581</td></tr>
<tr><td></td><td></td><td></td><td></td><td></td><td></td><td>3</td><td>14.37</td><td>3.19</td><td>96.3</td><td>(K)E D/F/G/G/G H/P D/P N/L/T Y A K(E)</td><td>573.6428</td><td>1717.782</td><td>1.1322</td></tr>
</table>

<table>
<tr><td></td><td></td><td></td><td></td><td></td><td></td><td>2</td><td>12.92</td><td>5.46</td><td>98.7</td><td>(R)G M/T/L\V|V|S|G D/G R(Y)</td><td>546.4218</td><td>1091.551</td><td>0.285</td></tr>
<tr><td></td><td></td><td></td><td></td><td></td><td></td><td>2</td><td>12.81</td><td>3.6</td><td>94.1</td><td>(K)S/I|F/D/F/Q|S I|Q\K(L)</td><td>606.8447</td><td>1212.626</td><td>0.0562</td></tr>
<tr><td></td><td></td><td></td><td></td><td></td><td></td><td>2</td><td>11.45</td><td>2.96</td><td>97.1</td><td>(R)S M|P/T/S/A|A L|D V\V|A K(H)</td><td>645.2</td><td>1289.677</td><td>-0.2842</td></tr>
<tr><td>1390</td><td>XP_002283510.1</td><td>T-complex protein 1 subunit zeta</td><td>9(9)</td><td>20%</td><td>137.21</td><td>2</td><td>18.95</td><td>9.84</td><td>97.8</td><td>(K)G I/D|P/P/S/L|D|L\L|A\R(A)</td><td>634.1375</td><td>1266.705</td><td>0.5625</td></tr>
<tr><td></td><td></td><td>(Vitis vinifera)</td><td></td><td></td><td></td><td>2</td><td>18.55</td><td>8.74</td><td>96.5</td><td>(R)L/V/E/G|L|V|L|D|H\G S R(H)</td><td>647.8379</td><td>1294.711</td><td>-0.0429</td></tr>
<tr><td></td><td></td><td></td><td></td><td></td><td></td><td>2</td><td>16.57</td><td>7.89</td><td>92.1</td><td>(K)E/M/Q|I|Q|N|P/T A|I|M\I\A R(T)</td><td>809.1174</td><td>1615.83</td><td>1.3981</td></tr>
<tr><td></td><td></td><td></td><td></td><td></td><td></td><td>2</td><td>16.53</td><td>7.78</td><td>90.7</td><td>(K)S/A/A|L/H|M/N/I|N A\A\K(G)</td><td>620.9509</td><td>1240.647</td><td>0.2479</td></tr>
<tr><td></td><td></td><td></td><td></td><td></td><td></td><td>3</td><td>16.52</td><td>6.4</td><td>91.2</td><td>(K)T L/A/E/N/S|G/L/D/T Q D/V/I|I/A L T/G E H D R(G)</td><td>823.7402</td><td>2468.226</td><td>0.9797</td></tr>
<tr><td></td><td></td><td></td><td></td><td></td><td></td><td>2</td><td>13.91</td><td>4.51</td><td>97.2</td><td>(R)A/T|L/Q/F|L|E\K(F)</td><td>475.5929</td><td>949.535</td><td>0.6432</td></tr>
<tr><td></td><td></td><td></td><td></td><td></td><td></td><td>2</td><td>12.68</td><td>4.89</td><td>96.6</td><td>(R)V/L|V|D|G/F E I\A\K(R)</td><td>546.1868</td><td>1090.614</td><td>0.752</td></tr>
<tr><td></td><td></td><td></td><td></td><td></td><td></td><td>2</td><td>12.44</td><td>2.13</td><td>97.7</td><td>(R)V/L|N|P|N/A E\V\L\N K(S)</td><td>606.0627</td><td>1210.679</td><td>0.4391</td></tr>
<tr><td></td><td></td><td></td><td></td><td></td><td></td><td>2</td><td>11.06</td><td>2.8</td><td>82.4</td><td>(K)Y/T|F|V|E/N/V K(N)</td><td>500.9297</td><td>999.515</td><td>1.3376</td></tr>
<tr><td>1391</td><td>AAX48772</td><td>9,10[9',10']carotenoid cleavage dioxygenase</td><td>4(4)</td><td>9%</td><td>60.26</td><td>2</td><td>19.65</td><td>5.23</td><td>97.1</td><td>(R)L E|N|P/D/L|D|L/V|G G D\V\K(E)</td><td>742.6375</td><td>1483.764</td><td>0.5039</td></tr>
<tr><td></td><td></td><td>(Vitis vinifera)</td><td></td><td></td><td></td><td>2</td><td>15.73</td><td>8.91</td><td>86.5</td><td>(K)L L/A|L/S/E/A/D|K|P Y V\L\K(V)</td><td>780.5034</td><td>1559.904</td><td>0.0952</td></tr>
<tr><td></td><td></td><td></td><td></td><td></td><td></td><td>2</td><td>13.63</td><td>5.06</td><td>96.5</td><td>(R)Y V|Y/G/T|I|L D\S I A K(V)</td><td>672.4697</td><td>1342.725</td><td>1.2068</td></tr>
<tr><td></td><td></td><td></td><td></td><td></td><td></td><td>2</td><td>11.25</td><td>2.58</td><td>77.5</td><td>(R)F/G S E|A/V|F|V|P R(E)</td><td>555.1102</td><td>1108.579</td><td>0.6346</td></tr>
<tr><td>1411</td><td>XP_010664138.1</td><td>V-type proton ATPase subunit B 2 isoform X1</td><td>2(3)</td><td>6%</td><td>30.92</td><td>2</td><td>17</td><td>8.83</td><td>100</td><td>(K)A V/V Q/V|F/E/G|T|S/G I|D\N K(Y)</td><td>782.5354</td><td>1563.801</td><td>0.2622</td></tr>
<tr><td></td><td></td><td>(Vitis vinifera)</td><td></td><td></td><td></td><td>2</td><td>16</td><td>8.68</td><td>100</td><td>(K)A V/V/Q/V|F|E/G\T/S|G I\D N K(Y)</td><td>782.4243</td><td>1563.801</td><td>0.04</td></tr>
<tr><td></td><td></td><td></td><td></td><td></td><td></td><td>2</td><td>13.92</td><td>5.59</td><td>97.6</td><td>(R)T V/S/G V|A|G/P L V|I\L\E K(V)</td><td>692.1421</td><td>1382.825</td><td>0.4516</td></tr>
<tr><td>1437</td><td>XP_002263180.1</td><td>Dihydrolipoyl dehydrogenase, mitochondrial</td><td>10(19)</td><td>29%</td><td>161.18</td><td>2</td><td>19.71</td><td>9.34</td><td>95</td><td>(K)F/I/S/P S E/V|S/V|D/T/I|E|G G\N A V\V\K(G)</td><td>1024.5845</td><td>2048.055</td><td>0.1071</td></tr>
<tr><td></td><td></td><td>(Vitis vinifera)</td><td></td><td></td><td></td><td>3</td><td>19.18</td><td>6.58</td><td>93.9</td><td>(K)F/I/S P S E|V|S V\D T|I|E|G/G\N A|V\V\K(G)</td><td>683.7993</td><td>2048.055</td><td>1.3287</td></tr>
<tr><td></td><td></td><td></td><td></td><td></td><td></td><td>2</td><td>18.08</td><td>6.21</td><td>94</td><td>(K)F/I/S/P S E V|S/V|D|T I|E/G G\N A V\V\K(G)</td><td>1025.1309</td><td>2048.055</td><td>1.1999</td></tr>
<tr><td></td><td></td><td></td><td></td><td></td><td></td><td>2</td><td>17.97</td><td>5.89</td><td>92</td><td>(R)I/V/S/S/T/G/A/L|A|L|S/E|I\P K(K)</td><td>743.6794</td><td>1485.852</td><td>0.4992</td></tr>
<tr><td></td><td></td><td></td><td></td><td></td><td></td><td>2</td><td>17.87</td><td>4.36</td><td>96.4</td><td>(K)V/A|G|V|D|T/S/G D L\V\K(L)</td><td>581.0757</td><td>1160.616</td><td>0.5284</td></tr>
<tr><td></td><td></td><td></td><td></td><td></td><td></td><td>2</td><td>17.64</td><td>7.09</td><td>97.3</td><td>(K)N/I|I|I|A|T|G/S|D\V\K(S)</td><td>565.8496</td><td>1130.642</td><td>0.0504</td></tr>
<tr><td></td><td></td><td></td><td></td><td></td><td></td><td>2</td><td>17.25</td><td>9.48</td><td>85</td><td>(K)L T L E/P A/A/G G/E/Q S/T/L/E|A\D|V\V|L V S A G R(S)</td><td>1242.7212</td><td>2483.299</td><td>1.1363</td></tr>
<tr><td></td><td></td><td></td><td></td><td></td><td></td><td>2</td><td>17.01</td><td>6.6</td><td>87.9</td><td>(R)I/V/S S/T/G/A/L/A/L|S/E|I|P K(K)</td><td>743.345</td><td>1485.852</td><td>-0.1696</td></tr>
<tr><td></td><td></td><td></td><td></td><td></td><td></td><td>2</td><td>16.71</td><td>5.35</td><td>97.5</td><td>(K)A/I|D|D|A|E\G\L|V\K(I)</td><td>515.9166</td><td>1030.542</td><td>0.2844</td></tr>
<tr><td></td><td></td><td></td><td></td><td></td><td></td><td>2</td><td>15.74</td><td>5.56</td><td>89.3</td><td>(R)G T/L/G G T/C/L|N|V|G/C/I|P S K(A)</td><td>817.656</td><td>1633.804</td><td>0.5011</td></tr>
<tr><td></td><td></td><td></td><td></td><td></td><td></td><td>2</td><td>14.25</td><td>3.73</td><td>86.2</td><td>(K)F/P/S/V/E|V|D|L|P A M M\G Q\K(D)</td><td>825.3655</td><td>1648.807</td><td>0.9164</td></tr>
<tr><td></td><td></td><td></td><td></td><td></td><td></td><td>2</td><td>14</td><td>6.2</td><td>100</td><td>(K)F I/S/P S E/V|S/V|D/T/I/E|G G\N A V V K(G)</td><td>1024.7412</td><td>2048.055</td><td>0.4205</td></tr>
<tr><td></td><td></td><td></td><td></td><td></td><td></td><td>2</td><td>12.89</td><td>4.58</td><td>84.1</td><td>(R)S/P/F|T/A|G L/G/L D\K(I)</td><td>553.1255</td><td>1105.589</td><td>-0.3451</td></tr>
<tr><td></td><td></td><td></td><td></td><td></td><td></td><td>2</td><td>12.72</td><td>4.01</td><td>86.6</td><td>(R)S/P/F|T|A|G L/G L D K(I)</td><td>553.3406</td><td>1105.589</td><td>0.0851</td></tr>
<tr><td></td><td></td><td></td><td></td><td></td><td></td><td>2</td><td>12.07</td><td>3.27</td><td>79.9</td><td>(R)I/V/S S T/G A/L/A|L|S/E\I\P\K(K)</td><td>743.5625</td><td>1485.852</td><td>0.2654</td></tr>
<tr><td></td><td></td><td></td><td></td><td></td><td></td><td>2</td><td>11.15</td><td>4.71</td><td>75.1</td><td>(K)E/A/A|M/A/T/Y D|K/P I\H\I(-)</td><td>730.8191</td><td>1459.725</td><td>0.9059</td></tr>
<tr><td></td><td></td><td></td><td></td><td></td><td></td><td>2</td><td>10.96</td><td>4.13</td><td>91.2</td><td>(K)F I/S/P S/E/V|S/V/D|T/I E/G G\N A V V K(G)</td><td>1024.7148</td><td>2048.055</td><td>0.3677</td></tr>
<tr><td></td><td></td><td></td><td></td><td></td><td></td><td>2</td><td>10.8</td><td>2.41</td><td>72</td><td>(K)E A A m/A/T|Y\D|K/P I\H\I(-)</td><td>738.5111</td><td>1459.725</td><td>16.2899</td></tr>
<tr><td></td><td></td><td></td><td></td><td></td><td></td><td>2</td><td>10.67</td><td>2.59</td><td>70.9</td><td>(R)I/V/S S T/G/A/L/A|L|S E|I\P K(K)</td><td>743.6958</td><td>1485.852</td><td>0.532</td></tr>
<tr><td>1447</td><td>XP_002284370.1</td><td>Probable mitochondrial-processing peptidase subunit beta</td><td>4(5)</td><td>13%</td><td>62.11</td><td>3</td><td>21.63</td><td>10.72</td><td>94.4</td><td>(K)L S T D/P T T\A|S Q\L\V|V\E|Q|P A/I|F/T|G S E V\R(M)</td><td>883.4031</td><td>2646.362</td><td>1.8326</td></tr>
<tr><td></td><td></td><td>(Vitis vinifera)</td><td></td><td></td><td></td><td>3</td><td>20.72</td><td>8.03</td><td>94.3</td><td>(K)L S T D/P/T T\A|S Q L\V|V\E\Q|P A/I|F|T G S E V\R(M)</td><td>883.1733</td><td>2646.362</td><td>1.1432</td></tr>
<tr><td></td><td></td><td></td><td></td><td></td><td></td><td>3</td><td>16.47</td><td>7.7</td><td>92.7</td><td>(K)S S L/L/L H I D G T|S|P|V A|E/D|I G R(Q)</td><td>661.0481</td><td>1980.04</td><td>1.0901</td></tr>
<tr><td></td><td></td><td></td><td></td><td></td><td></td><td>3</td><td>13.11</td><td>4.4</td><td>87.6</td><td>(K)A/H|L|Q/N Y I/S T/H/Y|T A P R(M)</td><td>591.729</td><td>1771.887</td><td>1.285</td></tr>
<tr><td></td><td></td><td></td><td></td><td></td><td></td><td>2</td><td>10.9</td><td>4.45</td><td>97.7</td><td>(R)T I|L|G|P/A Q/N I K(T)</td><td>528.0117</td><td>1054.626</td><td>0.3906</td></tr>
<tr><td>1450</td><td>XP_010664138.1</td><td>V-type proton ATPase subunit B 2 isoform X1</td><td>3(3)</td><td>8%</td><td>44.3</td><td>2</td><td>16.56</td><td>7.94</td><td>95.5</td><td>(K)Y T|T/V|Q|F/T/G E/V L\K(T)</td><td>694.0676</td><td>1385.731</td><td>1.3968</td></tr>
<tr><td></td><td></td><td>(Vitis vinifera)</td><td></td><td></td><td></td><td>2</td><td>13.89</td><td>6.09</td><td>96.6</td><td>(K)T/P|V|S/L|D|M L|G R(I)</td><td>545.1912</td><td>1088.577</td><td>0.7983</td></tr>
<tr><td></td><td></td><td></td><td></td><td></td><td></td><td>3</td><td>13.85</td><td>3.08</td><td>96.7</td><td>(R)D H A D V\S N Q|L|Y|A/N\Y A\I\G\K(D)</td><td>627.2726</td><td>1878.898</td><td>0.9052</td></tr>
<tr><td>1460</td><td>XP_002283951.1</td><td>ATP synthase subunit beta, mitochondrial</td><td>12(20)</td><td>32%</td><td>216.33</td><td>2</td><td>21.67</td><td>13.12</td><td>98.9</td><td>(R)L V/L/E|V/A|Q|H|L|G|E|N\M V\R(T)</td><td>854.52</td><td>1707.921</td><td>0.1117</td></tr>
<tr><td></td><td></td><td>(Vitis vinifera)</td><td></td><td></td><td></td><td>2</td><td>21.37</td><td>10.87</td><td>95.6</td><td>(R)I/P/S A/V|G/Y|Q|P T/L|A|T/D|L/G G L Q E R(-)</td><td>1093.8171</td><td>2186.145</td><td>0.4817</td></tr>
<tr><td></td><td></td><td></td><td></td><td></td><td></td><td>2</td><td>20.89</td><td>10.41</td><td>98.7</td><td>(K)T V/L|I|M|E|L|I|N|N\V\A\K(A)</td><td>729.5284</td><td>1457.84</td><td>0.2099</td></tr>
<tr><td></td><td></td><td></td><td></td><td></td><td></td><td>2</td><td>20.32</td><td>9.16</td><td>96.9</td><td>(R)E A/P S F/V/D/Q/A|T/E|Q/Q/I|L|V|T G I K(V)</td><td>1088.0361</td><td>2174.134</td><td>0.931</td></tr>
<tr><td></td><td></td><td></td><td></td><td></td><td></td><td>2</td><td>19.66</td><td>9.41</td><td>94.1</td><td>(R)V/G/L/T/G/L|T|V/A|E/H|F\R(F)</td><td>700.9087</td><td>1399.769</td><td>1.0409</td></tr>
<tr><td></td><td></td><td></td><td></td><td></td><td></td><td>2</td><td>19.31</td><td>7.23</td><td>99</td><td>(R)T I|A|M|D|G/T/E|G L|V\R(G)</td><td>632.0898</td><td>1262.641</td><td>0.5314</td></tr>
<tr><td></td><td></td><td></td><td></td><td></td><td></td><td>2</td><td>19.02</td><td>11.98</td><td>95.1</td><td>(R)F T/Q/A|N/S/E/V|S/A|L|L\G R(I)</td><td>747.4736</td><td>1492.775</td><td>1.1645</td></tr>
<tr><td></td><td></td><td></td><td></td><td></td><td></td><td>2</td><td>17.36</td><td>6.52</td><td>95.7</td><td>(R)Q I S E/L/G|I|Y|P/A/V|D|P L D/S T S R(M)</td><td>1031.6074</td><td>2061.05</td><td>1.1576</td></tr>
<tr><td></td><td></td><td></td><td></td><td></td><td></td><td>2</td><td>17.03</td><td>7.45</td><td>89.1</td><td>(R)T/I|A/m/D/G/T|E|G L/V R(G)</td><td>640.0553</td><td>1262.641</td><td>16.4624</td></tr>
<tr><td></td><td></td><td></td><td></td><td></td><td></td><td>2</td><td>16.39</td><td>9.86</td><td>90.6</td><td>(R)L V/L E V/A|Q|H|L|G|E\N m V R(T)</td><td>862.6313</td><td>1707.921</td><td>16.3343</td></tr>
<tr><td></td><td></td><td></td><td></td><td></td><td></td><td>2</td><td>16.26</td><td>5.51</td><td>92.4</td><td>(R)I/P/S A V/G Y/Q|P T\L|A/T/D/L/G/G L Q E R(-)</td><td>1094.2163</td><td>2186.145</td><td>1.2801</td></tr>
<tr><td></td><td></td><td></td><td></td><td></td><td></td><td>3</td><td>15.96</td><td>3.68</td><td>89.6</td><td>(K)A H G G/F S V|F|A\G\V|G|E\R(T)</td><td>464.4718</td><td>1390.686</td><td>0.7146</td></tr>
<tr><td></td><td></td><td></td><td></td><td></td><td></td><td>3</td><td>15.86</td><td>6.43</td><td>96.1</td><td>(R)V/G/L|T|G L|T|V|A/E H F\R(F)</td><td>467.3667</td><td>1399.769</td><td>0.3163</td></tr>
<tr><td></td><td></td><td></td><td></td><td></td><td></td><td>2</td><td>15.32</td><td>5.26</td><td>89.5</td><td>(R)E A/P S F/V/D Q/A/T/E/Q Q/I/L|V\T G I K(V)</td><td>1088.0439</td><td>2174.134</td><td>0.9466</td></tr>
<tr><td></td><td></td><td></td><td></td><td></td><td></td><td>2</td><td>15.04</td><td>6.24</td><td>91.7</td><td>(-)V L/N/T|G/S|P I|T|V|P V\G R(V)</td><td>705.6141</td><td>1409.811</td><td>0.4098</td></tr>
<tr><td></td><td></td><td></td><td></td><td></td><td></td><td>2</td><td>14.24</td><td>4.81</td><td>95.7</td><td>(R)L V/L E/V/A/Q/H|L|G E\N m V R(T)</td><td>862.4705</td><td>1707.921</td><td>16.0127</td></tr>
<tr><td></td><td></td><td></td><td></td><td></td><td></td><td>2</td><td>13.65</td><td>3.55</td><td>92</td><td>(R)I I/N|V|I|G|E|P I D\E R(G)</td><td>684.5037</td><td>1367.753</td><td>0.2472</td></tr>
<tr><td></td><td></td><td></td><td></td><td></td><td></td><td>2</td><td>13.58</td><td>3.84</td><td>79.4</td><td>(R)F T/Q/A|N|S E/V|S A/L|L\G R(I)</td><td>746.7014</td><td>1492.775</td><td>-0.3799</td></tr>
<tr><td></td><td></td><td></td><td></td><td></td><td></td><td>2</td><td>12.08</td><td>2.92</td><td>79.5</td><td>(K)E S I/T|S/F/Q|G V|L|D\G K(Y)</td><td>690.9919</td><td>1380.7</td><td>0.276</td></tr>
<tr><td></td><td></td><td></td><td></td><td></td><td></td><td>2</td><td>10.1</td><td>3.48</td><td>75.2</td><td>(K)T V/L I|m/E/L|I|N N V\A K(A)</td><td>737.8118</td><td>1457.84</td><td>16.7767</td></tr>
</table>

<table>
<tr><td>1463</td><td>XP_002283310.1</td><td>Mitochondrial-processing peptidase subunit alpha</td><td>5(9)</td><td>14%</td><td>88.63</td><td>3</td><td>21.42</td><td>12.59</td><td>98.8</td><td>(K)E L V/A|V|A|T|P G Q|V|D/Q V|Q/L\D R(A)</td><td>647.1826</td><td>1938.029</td><td>1.5042</td></tr>
<tr><td></td><td></td><td>(Vitis vinifera)</td><td></td><td></td><td></td><td>2</td><td>21.08</td><td>10.02</td><td>94.7</td><td>(R)N/P A/F|L|D|W|E|V|S/E/Q L\E\K(V)</td><td>903.6218</td><td>1804.875</td><td>1.3611</td></tr>
<tr><td></td><td></td><td></td><td></td><td></td><td></td><td>2</td><td>18.67</td><td>8.06</td><td>93.5</td><td>(K)T A|V|L/m|N/L|E|S R(M)</td><td>575.4556</td><td>1133.598</td><td>16.3056</td></tr>
<tr><td></td><td></td><td></td><td></td><td></td><td></td><td>2</td><td>17.79</td><td>8.4</td><td>94.3</td><td>(K)E L V A/V|A|T|P/G Q\V|D/Q\V Q L D R(A)</td><td>969.9287</td><td>1938.029</td><td>0.821</td></tr>
<tr><td></td><td></td><td></td><td></td><td></td><td></td><td>3</td><td>17.5</td><td>8.61</td><td>100</td><td>(K)E L/V/A/V|A|T|P G Q\V/D Q\V\Q L\D R(A)</td><td>646.8584</td><td>1938.029</td><td>0.5316</td></tr>
<tr><td></td><td></td><td></td><td></td><td></td><td></td><td>2</td><td>16.75</td><td>9.73</td><td>95.9</td><td>(K)T/A|V|L|M|N|L|E|S R(M)</td><td>567.5396</td><td>1133.598</td><td>0.4736</td></tr>
<tr><td></td><td></td><td></td><td></td><td></td><td></td><td>2</td><td>14.91</td><td>6.64</td><td>96.8</td><td>(R)E V E A|I|G|G N/V|T|A/S A\S R(E)</td><td>730.9382</td><td>1460.734</td><td>0.1352</td></tr>
<tr><td></td><td></td><td></td><td></td><td></td><td></td><td>2</td><td>12.55</td><td>4.21</td><td>87.4</td><td>(R)E Q|M/G|Y/T/F|D A L K(T)</td><td>651.9727</td><td>1302.603</td><td>0.3347</td></tr>
<tr><td></td><td></td><td></td><td></td><td></td><td></td><td>2</td><td>11.4</td><td>3.36</td><td>95.8</td><td>(K)E L V A V|A|T|P/G Q V/D/Q V\Q L D R(A)</td><td>969.6548</td><td>1938.029</td><td>0.2732</td></tr>
<tr><td>1471</td><td>XP_002283632.1</td><td>Enolase 1</td><td>5(11)</td><td>18%</td><td>84.96</td><td>2</td><td>21.44</td><td>11.57</td><td>98.9</td><td>(K)V Q|I|V|G/D/D L|L|V|T|N\P K(R)</td><td>755.7885</td><td>1510.848</td><td>-0.2778</td></tr>
<tr><td></td><td></td><td>(Vitis vinifera)</td><td></td><td></td><td></td><td>2</td><td>19.71</td><td>10.73</td><td>94.5</td><td>(K)V Q|I|V|G/D|D L|L|V|T|N P K(R)</td><td>756.4834</td><td>1510.848</td><td>1.112</td></tr>
<tr><td></td><td></td><td></td><td></td><td></td><td></td><td>2</td><td>17.6</td><td>10.84</td><td>90.8</td><td>(K)A V/A/N/V|N|T|I\I|G|P A L\I\G K(D)</td><td>776.1399</td><td>1550.927</td><td>0.3461</td></tr>
<tr><td></td><td></td><td></td><td></td><td></td><td></td><td>2</td><td>17.42</td><td>9.14</td><td>98.1</td><td>(R)I E/E E/L|G A|D/A|V|Y|A|G A\N F R(K)</td><td>913.0674</td><td>1824.876</td><td>0.2513</td></tr>
<tr><td></td><td></td><td></td><td></td><td></td><td></td><td>2</td><td>17.03</td><td>6.56</td><td>88.9</td><td>(K)V N/Q|I|G/T|V|T E|S I E\A V\K(M)</td><td>794.3451</td><td>1587.859</td><td>-0.1759</td></tr>
<tr><td></td><td></td><td></td><td></td><td></td><td></td><td>2</td><td>17.03</td><td>8.41</td><td>91.6</td><td>(K)V Q|I|V|G/D/D L|L|V|T|N\P K(R)</td><td>756.7291</td><td>1510.848</td><td>1.6034</td></tr>
<tr><td></td><td></td><td></td><td></td><td></td><td></td><td>2</td><td>14.72</td><td>7.02</td><td>85.6</td><td>(K)A V/A/N/V|N/T|I\I|G|P A L\I\G K(D)</td><td>776.1519</td><td>1550.927</td><td>0.3701</td></tr>
<tr><td></td><td></td><td></td><td></td><td></td><td></td><td>2</td><td>13.56</td><td>4.53</td><td>80.5</td><td>(K)V N/Q/I|G/T/V|T/E|S I E A\V\K(M)</td><td>794.9048</td><td>1587.859</td><td>0.9435</td></tr>
<tr><td></td><td></td><td></td><td></td><td></td><td></td><td>2</td><td>13.18</td><td>7.25</td><td>95.5</td><td>(K)V Q/I|V|G/D D L/L|V|T|N P K(R)</td><td>756.0483</td><td>1510.848</td><td>0.2418</td></tr>
<tr><td></td><td></td><td></td><td></td><td></td><td></td><td>2</td><td>12.74</td><td>6.27</td><td>89.2</td><td>(K)V Q/I|V/G/D D L/L|V|T|N P K(R)</td><td>756.2688</td><td>1510.848</td><td>0.6828</td></tr>
<tr><td></td><td></td><td></td><td></td><td></td><td></td><td>2</td><td>11.47</td><td>4.69</td><td>98.2</td><td>(K)V S A A/V|P/S G A/S/T/G I/Y E A L E\L R(D)</td><td>996.079</td><td>1991.044</td><td>0.1063</td></tr>
<tr><td>1490</td><td>XP_002276114.1</td><td>Leucine aminopeptidase 1</td><td>4(6)</td><td>11%</td><td>56.69</td><td>2</td><td>17.9</td><td>10.8</td><td>98.4</td><td>(K)S V D\I|I|G|L|G T|G|P D V\Q\K(K)</td><td>750.8586</td><td>1498.811</td><td>1.8988</td></tr>
<tr><td></td><td></td><td>(Vitis vinifera)</td><td></td><td></td><td></td><td>2</td><td>15.31</td><td>8.69</td><td>95.3</td><td>(K)E L V/N/A|P A/N/V/L|T|P G V|L A E E A S K(I)</td><td>1061.7634</td><td>2122.139</td><td>0.3805</td></tr>
<tr><td></td><td></td><td></td><td></td><td></td><td></td><td>3</td><td>12</td><td>4.94</td><td>100</td><td>(K)E L V/N A P/A N V\L/T|P/G V|L A E E A S\K(I)</td><td>707.9717</td><td>2122.139</td><td>-0.2385</td></tr>
<tr><td></td><td></td><td></td><td></td><td></td><td></td><td>2</td><td>11.78</td><td>7.97</td><td>88.1</td><td>(K)I Y T/A S A I/A/S/G A|V|L/G I/H|E D\N R(F)</td><td>1029.8616</td><td>2058.061</td><td>0.6545</td></tr>
<tr><td></td><td></td><td></td><td></td><td></td><td></td><td>2</td><td>11.7</td><td>4.1</td><td>94.8</td><td>(K)G D/I|L|A|V\G|V|T E K(D)</td><td>551.7258</td><td>1101.615</td><td>0.8293</td></tr>
<tr><td></td><td></td><td></td><td></td><td></td><td></td><td>3</td><td>11.41</td><td>3.73</td><td>97.8</td><td>(K)I/Y/T/A/S/A/I/A/S G A V L/G I/H E D/N R(F)</td><td>687.1113</td><td>2058.061</td><td>1.2579</td></tr>
<tr><td>1505</td><td>XP_002267091.2</td><td>Enolase</td><td>13(21)</td><td>36%</td><td>223.07</td><td>2</td><td>22.46</td><td>13.1</td><td>99.1</td><td>(K)V/Q|I|V|G/D|D/L|L|V|T|N\P K(R)</td><td>755.9448</td><td>1510.848</td><td>0.0348</td></tr>
<tr><td></td><td></td><td>(Vitis vinifera)</td><td></td><td></td><td></td><td>2</td><td>21.62</td><td>9.4</td><td>97.8</td><td>(K)V V/I/G m/D|V|A|A/S/E|F|Y|D|N\K(D)</td><td>887.8955</td><td>1757.842</td><td>16.9423</td></tr>
<tr><td></td><td></td><td></td><td></td><td></td><td></td><td>2</td><td>21.25</td><td>10.7</td><td>100</td><td>(R)I E/E/E|L|G|S A|A|V|Y\A\G A K(F)</td><td>754.3463</td><td>1507.764</td><td>-0.0785</td></tr>
<tr><td></td><td></td><td></td><td></td><td></td><td></td><td>2</td><td>18.89</td><td>8.34</td><td>94.6</td><td>(R)A A/V/P S/G/A|S/T/G/I\Y/E A L|E\L R(D)</td><td>903.2651</td><td>1804.944</td><td>0.579</td></tr>
<tr><td></td><td></td><td></td><td></td><td></td><td></td><td>2</td><td>18.77</td><td>10.63</td><td>95.1</td><td>(K)Y G Q D/A/T/N/V|G/D|E/G G/F|A|P N I\Q E N K(E)</td><td>1163.0986</td><td>2324.043</td><td>1.1474</td></tr>
<tr><td></td><td></td><td></td><td></td><td></td><td></td><td>2</td><td>17.48</td><td>9.17</td><td>94</td><td>(K)A V/E N V/N A/I|I/A|P A/L|I|G K D|P T E Q V K(I)</td><td>1196.5833</td><td>2390.329</td><td>1.8304</td></tr>
<tr><td></td><td></td><td></td><td></td><td></td><td></td><td>2</td><td>17.17</td><td>7.54</td><td>97.6</td><td>(K)L A/m/Q E|F/m|I|L|P L/G\A S S F\K(E)</td><td>958.088</td><td>1882.98</td><td>32.1882</td></tr>
<tr><td></td><td></td><td></td><td></td><td></td><td></td><td>2</td><td>16.65</td><td>5.89</td><td>90.2</td><td>(K)V N/Q|I|G/S/V|T/E/S I|E A\V\K(M)</td><td>788.3102</td><td>1573.843</td><td>1.7699</td></tr>
<tr><td></td><td></td><td></td><td></td><td></td><td></td><td>2</td><td>16.28</td><td>7.4</td><td>95.7</td><td>(K)L V/L/P V/P A/F|N\V/I\N|G G S H A G N K(L)</td><td>1002.8147</td><td>2004.103</td><td>0.5196</td></tr>
<tr><td></td><td></td><td></td><td></td><td></td><td></td><td>1</td><td>15.8</td><td>15.8</td><td>86.4</td><td>(R)I E/E/E/L/G/S/A|A|V|Y\A\G A\K(F)</td><td>1507.6484</td><td>1507.764</td><td>-0.1155</td></tr>
<tr><td></td><td></td><td></td><td></td><td></td><td></td><td>1</td><td>15.36</td><td>8.53</td><td>88.3</td><td>(K)V Q/I/V/G D/D/L|L\V\T\N\P K(R)</td><td>1510.677</td><td>1510.848</td><td>-0.1705</td></tr>
<tr><td></td><td></td><td></td><td></td><td></td><td></td><td>1</td><td>14.6</td><td>6.15</td><td>93.9</td><td>(K)A V/E N V/N/A/I/I|A|P A\L\I\G\K(D)</td><td>1592.6296</td><td>1592.937</td><td>-0.3074</td></tr>
<tr><td></td><td></td><td></td><td></td><td></td><td></td><td>2</td><td>14</td><td>5.97</td><td>100</td><td>(K)L A m Q/E/F/m/I|L|P L|G A S S F K(E)</td><td>958.6552</td><td>1882.98</td><td>33.3226</td></tr>
<tr><td></td><td></td><td></td><td></td><td></td><td></td><td>3</td><td>13.71</td><td>5.29</td><td>97.6</td><td>(K)T/Y|D|L/N/F|K E/E/N|N/D/G S Q K(I)</td><td>634.8137</td><td>1901.851</td><td>0.5754</td></tr>
<tr><td></td><td></td><td></td><td></td><td></td><td></td><td>3</td><td>13.48</td><td>3.52</td><td>92.5</td><td>(K)A V/E/N/V|N/A|I/I/A/P A L I G K D/P T E Q V K(I)</td><td>797.2693</td><td>2390.329</td><td>-0.5356</td></tr>
<tr><td></td><td></td><td></td><td></td><td></td><td></td><td>2</td><td>13.25</td><td>5.58</td><td>100</td><td>(R)I E/E E|L|G/S A|A/V|Y A G A K(F)</td><td>754.7644</td><td>1507.764</td><td>0.7577</td></tr>
<tr><td></td><td></td><td></td><td></td><td></td><td></td><td>2</td><td>12.89</td><td>2.87</td><td>96.1</td><td>(K)L A m Q E/F/M|I|L|P\L/G A/S S F K(E)</td><td>950.5835</td><td>1882.98</td><td>17.1792</td></tr>
<tr><td></td><td></td><td></td><td></td><td></td><td></td><td>2</td><td>12.86</td><td>4.12</td><td>84.7</td><td>(R)A/G W/G|V|M/A/S/H\R(S)</td><td>536.573</td><td>1071.515</td><td>0.6235</td></tr>
<tr><td></td><td></td><td></td><td></td><td></td><td></td><td>2</td><td>11.82</td><td>6.83</td><td>95.6</td><td>(K)A V E N V N A/I/I/A/P A/L/I/G/K D|P T\E Q V K(I)</td><td>1195.9197</td><td>2390.329</td><td>0.5032</td></tr>
<tr><td></td><td></td><td></td><td></td><td></td><td></td><td>2</td><td>11.33</td><td>6.44</td><td>88.3</td><td>(K)V V/I/G m D/V/A/A|S E F Y D/N K\D K(T)</td><td>1009.5009</td><td>2000.963</td><td>17.0312</td></tr>
<tr><td></td><td></td><td></td><td></td><td></td><td></td><td>3</td><td>10.94</td><td>3.1</td><td>97.1</td><td>(K)V/V/I/G/m/D V/A A/S\E F Y D N K\D K(T)</td><td>673.3765</td><td>2000.963</td><td>17.1516</td></tr>
<tr><td>1514</td><td>XP_002285721.1</td><td>Tubulin alpha-3 chain</td><td>5(6)</td><td>15%</td><td>72.65</td><td>2</td><td>16.5</td><td>7.6</td><td>100</td><td>(R)I/H/F|M|L|S|S/Y|A|P V I\S A E K(A)</td><td>897.3088</td><td>1792.93</td><td>0.6801</td></tr>
<tr><td></td><td></td><td>(Vitis vinifera)</td><td></td><td></td><td></td><td>2</td><td>15.25</td><td>5.89</td><td>100</td><td>(K)D V|N|A|A|V|A|T I K(T)</td><td>501.6277</td><td>1001.563</td><td>0.6855</td></tr>
<tr><td></td><td></td><td></td><td></td><td></td><td></td><td>2</td><td>15.25</td><td>10.66</td><td>96.9</td><td>(R)I/H/F|M|L|S/S|Y\A|P V I S A E K(A)</td><td>897.3257</td><td>1792.93</td><td>0.7139</td></tr>
<tr><td></td><td></td><td></td><td></td><td></td><td></td><td>2</td><td>14.5</td><td>6.62</td><td>100</td><td>(R)T V/Q/F|V|D/W|C|P T G F\K(C)</td><td>792.907</td><td>1584.752</td><td>0.0552</td></tr>
<tr><td></td><td></td><td></td><td></td><td></td><td></td><td>2</td><td>13.4</td><td>3.83</td><td>72.3</td><td>(R)A V/C m/I/S/N/N/T/A/V|A/E/V/F S R(I)</td><td>942.1139</td><td>1868.899</td><td>14.3212</td></tr>
<tr><td></td><td></td><td></td><td></td><td></td><td></td><td>3</td><td>13</td><td>3.94</td><td>100</td><td>(R)S L|D|I/E R/P T Y\T|N/L N R(L)</td><td>564.9504</td><td>1691.871</td><td>0.9655</td></tr>
<tr><td>1517</td><td>XP_002283632.1</td><td>Enolase 1</td><td>4(10)</td><td>15%</td><td>75.09</td><td>2</td><td>23</td><td>13.11</td><td>100</td><td>(K)V/Q|I|V|G/D/D/L|L|V|T|N\P\K(R)</td><td>755.9578</td><td>1510.848</td><td>0.0608</td></tr>
<tr><td></td><td></td><td>(Vitis vinifera)</td><td></td><td></td><td></td><td>3</td><td>18.94</td><td>6.79</td><td>88.2</td><td>(K)Y G Q D A\T\N/V|G D E/G G\F|A|P/N/I|Q E N K(E)</td><td>775.4735</td><td>2324.043</td><td>0.3634</td></tr>
<tr><td></td><td></td><td></td><td></td><td></td><td></td><td>2</td><td>17.23</td><td>6.32</td><td>93.5</td><td>(K)V N/Q|I|G/T/V|T E|S/I/E A V\K(M)</td><td>794.4683</td><td>1587.859</td><td>0.0705</td></tr>
<tr><td></td><td></td><td></td><td></td><td></td><td></td><td>2</td><td>16.5</td><td>10.45</td><td>100</td><td>(K)V Q/I|V|G/D/D L|L|V|T|N\P K(R)</td><td>756.2312</td><td>1510.848</td><td>0.6076</td></tr>
<tr><td></td><td></td><td></td><td></td><td></td><td></td><td>2</td><td>15.92</td><td>7.34</td><td>89.1</td><td>(K)A V A/N/V|N/T\I\I|G|P A L|I\G K(D)</td><td>775.9041</td><td>1550.927</td><td>-0.1255</td></tr>
<tr><td></td><td></td><td></td><td></td><td></td><td></td><td>2</td><td>15.64</td><td>7.47</td><td>84.2</td><td>(K)V/Q/I|V/G/D D/L\L\V|T|N\P K(R)</td><td>756.3594</td><td>1510.848</td><td>0.864</td></tr>
<tr><td></td><td></td><td></td><td></td><td></td><td></td><td>2</td><td>15.41</td><td>8.7</td><td>98.3</td><td>(K)V Q/I|V/G/D/D L/L|V|T|N P K(R)</td><td>756.2413</td><td>1510.848</td><td>0.6278</td></tr>
<tr><td></td><td></td><td></td><td></td><td></td><td></td><td>2</td><td>14.25</td><td>6.24</td><td>83.8</td><td>(K)V N/Q|I|G/T/V|T E/S/I E A\V\K(M)</td><td>794.7544</td><td>1587.859</td><td>0.6427</td></tr>
<tr><td></td><td></td><td></td><td></td><td></td><td></td><td>2</td><td>12.54</td><td>4.78</td><td>82.3</td><td>(K)A/V/A/N/V|N/T I I|G|P A L\I\G K(D)</td><td>776.3083</td><td>1550.927</td><td>0.6829</td></tr>
<tr><td></td><td></td><td></td><td></td><td></td><td></td><td>2</td><td>12.13</td><td>3.16</td><td>92.1</td><td>(K)V N Q/I|G T/V/T/E|S I E A V\K(M)</td><td>794.9153</td><td>1587.859</td><td>0.9645</td></tr>
<tr><td>1531</td><td>XP_002267091.2</td><td>Enolase</td><td>13(32)</td><td>37%</td><td>222.9</td><td>2</td><td>21.96</td><td>12.19</td><td>99.2</td><td>(K)V Q|I|V|G/D|D/L|L|V|T|N\P K(R)</td><td>756.1738</td><td>1510.848</td><td>0.4928</td></tr>
<tr><td></td><td></td><td>(Vitis vinifera)</td><td></td><td></td><td></td><td>2</td><td>20.9</td><td>11.79</td><td>97.9</td><td>(K)V N/Q|I|G/S/V|T/E|S I/E A\V\K(M)</td><td>787.2893</td><td>1573.843</td><td>-0.2719</td></tr>
<tr><td></td><td></td><td></td><td></td><td></td><td></td><td>2</td><td>20.17</td><td>8.64</td><td>97.8</td><td>(R)I E/E|E|L|G|S A|A|V|Y\A\G A K(F)</td><td>754.4357</td><td>1507.764</td><td>0.1003</td></tr>
<tr><td></td><td></td><td></td><td></td><td></td><td></td><td>2</td><td>19.52</td><td>10.52</td><td>91.6</td><td>(K)V Q|I|V|G/D/D/L|L|V|T|N\P K(R)</td><td>755.8408</td><td>1510.848</td><td>-0.1732</td></tr>
</table>

<table>
<tr><td></td><td></td><td></td><td></td><td></td><td></td><td></td><td></td><td></td><td></td><td></td><td></td><td></td><td></td></tr>
<tr><td></td><td></td><td></td><td></td><td></td><td></td><td>2</td><td>18.95</td><td>11.29</td><td>86.7</td><td>(K)V Q/I|V|G/D/D/L|L|V|T|N\P K(R)</td><td>756.0818</td><td>1510.848</td><td>0.3088</td></tr>
<tr><td></td><td></td><td></td><td></td><td></td><td></td><td>2</td><td>18.86</td><td>11.34</td><td>90.6</td><td>(K)V Q/I|V|G/D|D/L/L|V|T|N\P K(R)</td><td>756.2454</td><td>1510.848</td><td>0.636</td></tr>
<tr><td></td><td></td><td></td><td></td><td></td><td></td><td>2</td><td>18.63</td><td>7.42</td><td>93.7</td><td>(K)V V/I/G m/D|V|A|A/S/E|F|Y D\N\K(D)</td><td>887.7195</td><td>1757.842</td><td>16.5903</td></tr>
<tr><td></td><td></td><td></td><td></td><td></td><td></td><td>2</td><td>17.84</td><td>8.2</td><td>91.3</td><td>(R)A A/V/P/S/G A/S/T|G I\Y|E A|L\E\L\R(D)</td><td>902.9336</td><td>1804.944</td><td>-0.084</td></tr>
<tr><td></td><td></td><td></td><td></td><td></td><td></td><td>2</td><td>17.7</td><td>9.38</td><td>87.9</td><td>(K)V Q|I|V|G/D/D L|L|V|T|N\P K(R)</td><td>755.9337</td><td>1510.848</td><td>0.0126</td></tr>
<tr><td></td><td></td><td></td><td></td><td></td><td></td><td>2</td><td>17.39</td><td>7.05</td><td>92</td><td>(K)V N/Q|I|G/S/V|T/E/S/I\E\A\V\K(M)</td><td>787.5682</td><td>1573.843</td><td>0.2859</td></tr>
<tr><td></td><td></td><td></td><td></td><td></td><td></td><td>3</td><td>17.26</td><td>6.15</td><td>95.1</td><td>(K)K Y G Q D A T N/V|G\D E G/G/F|A|P N/I|Q E\N\K(E)</td><td>818.4077</td><td>2452.137</td><td>1.071</td></tr>
<tr><td></td><td></td><td></td><td></td><td></td><td></td><td>2</td><td>17.21</td><td>5.92</td><td>92.3</td><td>(K)L A m/Q\E|F|m I|L|P L|G A S/S F K(E)</td><td>958.1603</td><td>1882.98</td><td>32.3328</td></tr>
<tr><td></td><td></td><td></td><td></td><td></td><td></td><td>2</td><td>17.1</td><td>10.06</td><td>95.7</td><td>(R)G/N/P T V/E/V|D|V|T|L|S|D/G T F A R(A)</td><td>939.7681</td><td>1877.924</td><td>0.605</td></tr>
<tr><td></td><td></td><td></td><td></td><td></td><td></td><td>2</td><td>17.08</td><td>6.81</td><td>89.1</td><td>(K)V/N/Q/I|G/S/V|T/E/S I/E\A\V\K(M)</td><td>787.2346</td><td>1573.843</td><td>-0.3813</td></tr>
<tr><td></td><td></td><td></td><td></td><td></td><td></td><td>2</td><td>16.89</td><td>6.96</td><td>89.2</td><td>(K)Y G Q D A/T/N/V|G D|E/G/G F|A|P/N I\Q E N K(E)</td><td>1162.8569</td><td>2324.043</td><td>0.664</td></tr>
<tr><td></td><td></td><td></td><td></td><td></td><td></td><td>2</td><td>16.84</td><td>6.78</td><td>90.8</td><td>(R)A A/V/P/S/G/A|S/T/G/I|Y E\A\L E\L R(D)</td><td>903.5137</td><td>1804.944</td><td>1.0762</td></tr>
<tr><td></td><td></td><td></td><td></td><td></td><td></td><td>2</td><td>16.66</td><td>6.39</td><td>91.1</td><td>(K)V N/Q|I|G/S/V/T/E/S/I\E\A\V\K(M)</td><td>787.8379</td><td>1573.843</td><td>0.8253</td></tr>
<tr><td></td><td></td><td></td><td></td><td></td><td></td><td>3</td><td>16.51</td><td>4.77</td><td>88.8</td><td>(K)A/V/E/N/V|N/A|I|I/A/P A L I/G K D/P T E Q V K(I)</td><td>798.0891</td><td>2390.329</td><td>1.9238</td></tr>
<tr><td></td><td></td><td></td><td></td><td></td><td></td><td>2</td><td>16.49</td><td>7.97</td><td>85.5</td><td>(K)V Q/I|V|G/D/D/L/L|V|T|N\P K(R)</td><td>756.1537</td><td>1510.848</td><td>0.4526</td></tr>
<tr><td></td><td></td><td></td><td></td><td></td><td></td><td>2</td><td>16.22</td><td>5.72</td><td>91.2</td><td>(K)V N/Q|I|G/S/V|T/E/S/I/E A V\K(M)</td><td>787.5486</td><td>1573.843</td><td>0.2467</td></tr>
<tr><td></td><td></td><td></td><td></td><td></td><td></td><td>2</td><td>15.93</td><td>7.79</td><td>96.2</td><td>(R)A A/V/P S/G A|S|T|G I Y E|A\L\E\L R(D)</td><td>903.3379</td><td>1804.944</td><td>0.7246</td></tr>
<tr><td></td><td></td><td></td><td></td><td></td><td></td><td>2</td><td>15.85</td><td>5.39</td><td>82.6</td><td>(K)V/N/Q|I|G|S/V|T|E/S I/E A V K(M)</td><td>788.0264</td><td>1573.843</td><td>1.2023</td></tr>
<tr><td></td><td></td><td></td><td></td><td></td><td></td><td>3</td><td>15.63</td><td>4.49</td><td>93.5</td><td>(K)T/Y|D|L|N/F|K/E E/N|N/D|G S Q K(I)</td><td>634.8689</td><td>1901.851</td><td>0.741</td></tr>
<tr><td></td><td></td><td></td><td></td><td></td><td></td><td>3</td><td>15.59</td><td>3.07</td><td>81.3</td><td>(K)K Y G/Q\D A T N V G D\E\G/G|F|A/P N I\Q E N\K(E)</td><td>818.0941</td><td>2452.137</td><td>0.1302</td></tr>
<tr><td></td><td></td><td></td><td></td><td></td><td></td><td>2</td><td>15.29</td><td>6.58</td><td>90.4</td><td>(R)A A/V/P S G A/S/T/G I|Y\E A\L\E\L R(D)</td><td>903.1365</td><td>1804.944</td><td>0.3218</td></tr>
<tr><td></td><td></td><td></td><td></td><td></td><td></td><td>3</td><td>15</td><td>4.43</td><td>80.5</td><td>(K)Y G Q D A|T N V|G/D E/G G\F|A|P N I\Q E N K(E)</td><td>775.7588</td><td>2324.043</td><td>1.2193</td></tr>
<tr><td></td><td></td><td></td><td></td><td></td><td></td><td>2</td><td>13.74</td><td>3.67</td><td>85.9</td><td>(K)V N/Q|I|G/S/V|T/E/S I E A V\K(M)</td><td>787.6848</td><td>1573.843</td><td>0.5191</td></tr>
<tr><td></td><td></td><td></td><td></td><td></td><td></td><td>2</td><td>13.42</td><td>3.04</td><td>72.5</td><td>(K)V N/Q|I|G/S/V/T/E\S/I/E A V\K(M)</td><td>787.2576</td><td>1573.843</td><td>-0.3353</td></tr>
<tr><td></td><td></td><td></td><td></td><td></td><td></td><td>2</td><td>12.7</td><td>3.79</td><td>98.4</td><td>(K)L A M Q/E/F m|I|L|P L G A\S S F K(E)</td><td>950.5153</td><td>1882.98</td><td>17.0428</td></tr>
<tr><td></td><td></td><td></td><td></td><td></td><td></td><td>2</td><td>12.11</td><td>2</td><td>83.9</td><td>(K)Y/N|Q L|I|R(I)</td><td>404.0049</td><td>806.452</td><td>0.5506</td></tr>
<tr><td></td><td></td><td></td><td></td><td></td><td></td><td>2</td><td>10.69</td><td>2.39</td><td>96.2</td><td>(K)F R|A\P V|E|P\Y(-)</td><td>489.5565</td><td>978.504</td><td>-0.3986</td></tr>
<tr><td></td><td></td><td></td><td></td><td></td><td></td><td>3</td><td>10.25</td><td>3.44</td><td>96.4</td><td>(R)I E E E L\G S\A|A|V/Y A G A K(F)</td><td>503.6352</td><td>1507.764</td><td>1.1272</td></tr>
<tr><td>1576</td><td>XP_002280514.1</td><td>ATP-citrate synthase alpha chain protein 2</td><td>3(5)</td><td>9%</td><td>50.43</td><td>2</td><td>19.92</td><td>12.15</td><td>89.4</td><td>(R)V/L/S/P/T/E|G/Y I|H|S/L/D|E K(T)</td><td>844.6458</td><td>1687.854</td><td>0.4306</td></tr>
<tr><td></td><td></td><td>(Vitis vinifera)</td><td></td><td></td><td></td><td>2</td><td>16.31</td><td>5.06</td><td>91.5</td><td>(R)L/V|V|K/P D|M\L|F\G\K(R)</td><td>624.187</td><td>1246.723</td><td>0.6439</td></tr>
<tr><td></td><td></td><td></td><td></td><td></td><td></td><td>2</td><td>14.2</td><td>4.41</td><td>94.6</td><td>(R)G G/P/N/Y|Q|T/G L\A\R(M)</td><td>567.4973</td><td>1133.57</td><td>0.4175</td></tr>
<tr><td></td><td></td><td></td><td></td><td></td><td></td><td>3</td><td>13.62</td><td>2.16</td><td>93.8</td><td>(R)V/L|S|P T E G Y\I\H/S\L D|E K(T)</td><td>563.5493</td><td>1687.854</td><td>0.7796</td></tr>
<tr><td></td><td></td><td></td><td></td><td></td><td></td><td>3</td><td>11.9</td><td>3.13</td><td>97.3</td><td>(R)L/V|V/K|P D/M|L|F G K(R)</td><td>416.8131</td><td>1246.723</td><td>1.702</td></tr>
<tr><td>1597</td><td>XP_002283140.2</td><td>Aminoacylase-1 isoform X1</td><td>2(2)</td><td>5%</td><td>35.3</td><td>2</td><td>18.45</td><td>7.34</td><td>98.9</td><td>(K)L Y|D|N|T|A|M/E/N I\L\K(S)</td><td>713.2043</td><td>1424.709</td><td>0.6923</td></tr>
<tr><td></td><td></td><td>(Vitis vinifera)</td><td></td><td></td><td></td><td>3</td><td>16.85</td><td>5.02</td><td>88.5</td><td>(K)L G R P/E\I|F|P|A|S|T\D|A R(Y)</td><td>510.5555</td><td>1529.807</td><td>-0.1551</td></tr>
<tr><td>1616</td><td>BAB41020.1</td><td>UDP-glucose:flavonoid 3-O-glucosyltransferase</td><td>3(4)</td><td>6%</td><td>51</td><td>2</td><td>20.47</td><td>11.24</td><td>94.8</td><td>(R)m V|E|D|A|L|E|I|G|V\R(I)</td><td>624.134</td><td>1231.635</td><td>15.6256</td></tr>
<tr><td></td><td></td><td>(Vitis vinifera)</td><td></td><td></td><td></td><td>2</td><td>18.31</td><td>7.54</td><td>96.3</td><td>(K)T/L|V|D|L/V|S|K|P K(D)</td><td>550.2358</td><td>1099.672</td><td>-0.2078</td></tr>
<tr><td></td><td></td><td></td><td></td><td></td><td></td><td>2</td><td>12.77</td><td>5.43</td><td>85.2</td><td>(R)M V|E/D/A|L|E|I|G V\R(I)</td><td>616.2781</td><td>1231.635</td><td>-0.0862</td></tr>
<tr><td></td><td></td><td></td><td></td><td></td><td></td><td>2</td><td>12.22</td><td>4.92</td><td>93</td><td>(R)V/P|F|I|W|S L R(D)</td><td>509.1429</td><td>1017.588</td><td>-0.3095</td></tr>
<tr><td>1625</td><td>XP_003635049.1</td><td>Cysteine desulfurase 1, mitochondrial-like</td><td>6(9)</td><td>17%</td><td>96.58</td><td>2</td><td>19.81</td><td>9.56</td><td>96.7</td><td>(R)A V/E|L|T|V/Q|Q|V|E\K(L)</td><td>622.7273</td><td>1243.689</td><td>0.7581</td></tr>
<tr><td></td><td></td><td>(Vitis vinifera)</td><td></td><td></td><td></td><td>3</td><td>18.97</td><td>4.53</td><td>90.1</td><td>(R)T H L\Y\G\W|E|S|D/L|A|V\E\K(A)</td><td>550.1431</td><td>1647.801</td><td>0.6134</td></tr>
<tr><td></td><td></td><td></td><td></td><td></td><td></td><td>2</td><td>18.6</td><td>11.7</td><td>91.6</td><td>(R)T/H|L|Y|G|W|E|S\D|L/A\V\E K(A)</td><td>825.265</td><td>1647.801</td><td>1.7214</td></tr>
<tr><td></td><td></td><td></td><td></td><td></td><td></td><td>2</td><td>17.22</td><td>7.62</td><td>92.2</td><td>(K)E/L/G V|P|F/H|T/D/A/A Q A L\G K(I)</td><td>827.4264</td><td>1653.86</td><td>-0.014</td></tr>
<tr><td></td><td></td><td></td><td></td><td></td><td></td><td>2</td><td>16.83</td><td>11.52</td><td>97.3</td><td>(R)A Q|V|A|A|L|I|N A|S\P K(E)</td><td>592.3823</td><td>1182.684</td><td>1.0732</td></tr>
<tr><td></td><td></td><td></td><td></td><td></td><td></td><td>3</td><td>12.23</td><td>3.62</td><td>99</td><td>(R)A/L|G|V|D/E/D/M A H T S I R(F)</td><td>505.6935</td><td>1514.727</td><td>0.3392</td></tr>
<tr><td></td><td></td><td></td><td></td><td></td><td></td><td>2</td><td>11.6</td><td>4.68</td><td>74.3</td><td>(R)A/L/G/V/D/E/D/M/A/H|T S I R(F)</td><td>758.4304</td><td>1514.727</td><td>1.1268</td></tr>
<tr><td></td><td></td><td></td><td></td><td></td><td></td><td>2</td><td>11.52</td><td>3.06</td><td>74.1</td><td>(K)E S/Y/E/D/A D/G/I|S\M K(G)</td><td>673.385</td><td>1344.562</td><td>1.2003</td></tr>
<tr><td></td><td></td><td></td><td></td><td></td><td></td><td>2</td><td>11.41</td><td>5.52</td><td>86.7</td><td>(R)A Q/V/A/A/L|I|N A|S\P K(E)</td><td>592.0579</td><td>1182.684</td><td>0.4244</td></tr>
<tr><td>1653</td><td>ABM67590.1</td><td>Anthocyanidin synthase</td><td>6(15)</td><td>28%</td><td>100.14</td><td>3</td><td>19.92</td><td>8.92</td><td>97.7</td><td>(K)I I L K P\L\P E T V\S E|T|E|P/P L|F|P P R(T)</td><td>792.103</td><td>2373.343</td><td>0.9516</td></tr>
<tr><td></td><td></td><td>(Vitis vinifera)</td><td></td><td></td><td></td><td>2</td><td>18.9</td><td>10.63</td><td>97.1</td><td>(K)I I/L/K/P/L|P E/T/V|S E|T/E|P P L\F/P P R(T)</td><td>1187.3506</td><td>2373.343</td><td>0.3511</td></tr>
<tr><td></td><td></td><td></td><td></td><td></td><td></td><td>2</td><td>17.58</td><td>8.39</td><td>95.5</td><td>(K)E V/G G/M/E/E/L|L|L|Q\K(K)</td><td>673.5527</td><td>1345.703</td><td>0.3949</td></tr>
<tr><td></td><td></td><td></td><td></td><td></td><td></td><td>2</td><td>17.26</td><td>7.52</td><td>95.7</td><td>(K)V/A/G E/T/F|F/N|L|P M\E\E\K(E)</td><td>806.8926</td><td>1611.772</td><td>1.0056</td></tr>
<tr><td></td><td></td><td></td><td></td><td></td><td></td><td>2</td><td>16.96</td><td>8.8</td><td>96.9</td><td>(K)E V/G G m/E/E/L|L|L|Q\K(K)</td><td>681.4985</td><td>1345.703</td><td>16.2865</td></tr>
<tr><td></td><td></td><td></td><td></td><td></td><td></td><td>2</td><td>16.85</td><td>7.93</td><td>95.8</td><td>(K)I I/L/K|P/L/P E|T/V/S E|T\E|P P L F P P R(T)</td><td>1187.5298</td><td>2373.343</td><td>0.7095</td></tr>
<tr><td></td><td></td><td></td><td></td><td></td><td></td><td>2</td><td>15.99</td><td>7.53</td><td>84.5</td><td>(K)I L S V/L/S|L|G L|G|L|E\E\G R(L)</td><td>778.8745</td><td>1555.905</td><td>0.8363</td></tr>
<tr><td></td><td></td><td></td><td></td><td></td><td></td><td>3</td><td>15.61</td><td>5.07</td><td>89</td><td>(K)I I L K P L P E T V\S E/T|E|P/P L|F|P\P R(T)</td><td>792.0642</td><td>2373.343</td><td>0.8352</td></tr>
<tr><td></td><td></td><td></td><td></td><td></td><td></td><td>2</td><td>14.88</td><td>5.52</td><td>91.4</td><td>(K)V A/G E/T/F|F/N|L|P m E E K(E)</td><td>814.5876</td><td>1611.772</td><td>16.3956</td></tr>
<tr><td></td><td></td><td></td><td></td><td></td><td></td><td>3</td><td>14.83</td><td>3.3</td><td>85.4</td><td>(K)A A/M/E/W|G V/M/H L V N/H/G|I S\D|D/L\I N R(V)</td><td>827.2178</td><td>2478.202</td><td>1.4372</td></tr>
<tr><td></td><td></td><td></td><td></td><td></td><td></td><td>2</td><td>14.56</td><td>6.11</td><td>83.8</td><td>(R)V E/S L/S/S|S/G I|Q|S\I|P K(E)</td><td>716.6323</td><td>1431.769</td><td>0.4884</td></tr>
<tr><td></td><td></td><td></td><td></td><td></td><td></td><td>2</td><td>13.94</td><td>3.97</td><td>91</td><td>(K)E V/G G m/E/E/L/L|L|Q\K(K)</td><td>681.667</td><td>1345.703</td><td>16.6235</td></tr>
<tr><td></td><td></td><td></td><td></td><td></td><td></td><td>2</td><td>13.3</td><td>4.68</td><td>80.7</td><td>(R)V E/S/L/S/S/S/G I|Q|S I|P K(E)</td><td>716.6658</td><td>1431.769</td><td>0.5554</td></tr>
<tr><td></td><td></td><td></td><td></td><td></td><td></td><td>2</td><td>11.98</td><td>3.59</td><td>81.1</td><td>(R)V E/S L/S/S|S/G/I/Q|S I|P K(E)</td><td>716.5649</td><td>1431.769</td><td>0.3536</td></tr>
<tr><td></td><td></td><td></td><td></td><td></td><td></td><td>2</td><td>11.17</td><td>4.06</td><td>96.3</td><td>(K)E V/G G M/E/E/L|L|L Q\K(K)</td><td>673.4595</td><td>1345.703</td><td>0.2085</td></tr>
<tr><td>1661</td><td>XP_002282516.1</td><td>Actin-7</td><td>11(36)</td><td>37%</td><td>198.79</td><td>2</td><td>22.75</td><td>13.97</td><td>100</td><td>(K)L A/Y/V|A|L|D|Y|E|Q|E|L|E\T A\K(S)</td><td>929.3197</td><td>1855.932</td><td>1.6997</td></tr>
<tr><td></td><td></td><td>(Vitis vinifera)</td><td></td><td></td><td></td><td>2</td><td>21.97</td><td>9.18</td><td>99</td><td>(K)N Y|E|L|P D/G/Q/V|I|T|I|G A E R(F)</td><td>888.1166</td><td>1774.897</td><td>0.3289</td></tr>
<tr><td></td><td></td><td></td><td></td><td></td><td></td><td>2</td><td>21.5</td><td>8.6</td><td>100</td><td>(K)N Y|E|L|P D/G Q|V|I|T I|G A E R(F)</td><td>887.9701</td><td>1774.897</td><td>0.0359</td></tr>
<tr><td></td><td></td><td></td><td></td><td></td><td></td><td>2</td><td>20.63</td><td>12.12</td><td>97.1</td><td>(R)V/A/P E E/H|P/V|L L|T|E|A|P L N\P K(A)</td><td>977.8594</td><td>1954.064</td><td>0.6471</td></tr>
</table>

<table>
<tr><td></td><td></td><td></td><td></td><td></td><td></td><td>2</td><td>20.16</td><td>11.02</td><td>98.5</td><td>(K)L A/Y V|A|L|D|Y/E|Q|E\L|E\T\A\K(S)</td><td>928.8003</td><td>1855.932</td><td>0.6609</td></tr>
<tr><td></td><td></td><td></td><td></td><td></td><td></td><td>2</td><td>19.46</td><td>8.6</td><td>92.9</td><td>(K)Y/P I/E|H|G/I|V|S N W\D|D|M\E K(I)</td><td>967.2579</td><td>1932.88</td><td>0.6289</td></tr>
<tr><td></td><td></td><td></td><td></td><td></td><td></td><td>3</td><td>18.8</td><td>2.04</td><td>95.2</td><td>(K)I/W|H/H|T|F|Y/N|E|L R(V)</td><td>506.0833</td><td>1515.749</td><td>0.4862</td></tr>
<tr><td></td><td></td><td></td><td></td><td></td><td></td><td>2</td><td>18.22</td><td>10.06</td><td>95.1</td><td>(K)D L Y G N I|V|L|S|G/G S/T/M|F|P G I\A\D R(M)</td><td>1092.0078</td><td>2183.08</td><td>-0.0718</td></tr>
<tr><td></td><td></td><td></td><td></td><td></td><td></td><td>2</td><td>17.96</td><td>10.22</td><td>90.6</td><td>(K)L A Y V/A|L|D|Y/E|Q|E/L|E\T A K(S)</td><td>928.3971</td><td>1855.932</td><td>-0.1455</td></tr>
<tr><td></td><td></td><td></td><td></td><td></td><td></td><td>2</td><td>17.95</td><td>9.76</td><td>94.8</td><td>(K)L A/Y V/A|L|D|Y|E\Q\E\L\E\T A\K(S)</td><td>928.7969</td><td>1855.932</td><td>0.6541</td></tr>
<tr><td></td><td></td><td></td><td></td><td></td><td></td><td>2</td><td>17.93</td><td>7.22</td><td>97.7</td><td>(K)N Y|E|L|P D G Q|V|I|T I|G A E R(F)</td><td>888.043</td><td>1774.897</td><td>0.1817</td></tr>
<tr><td></td><td></td><td></td><td></td><td></td><td></td><td>2</td><td>17.27</td><td>7.31</td><td>94.6</td><td>(R)A/V/F/P/S/I V|G/R|P\R(H)</td><td>600.261</td><td>1198.706</td><td>0.8092</td></tr>
<tr><td></td><td></td><td></td><td></td><td></td><td></td><td>2</td><td>16.52</td><td>9.54</td><td>89.5</td><td>(R)V/A/P E E/H|P V|L L/T|E|A|P L N\P K(A)</td><td>978.4181</td><td>1954.064</td><td>1.7645</td></tr>
<tr><td></td><td></td><td></td><td></td><td></td><td></td><td>2</td><td>16.51</td><td>5.08</td><td>86.3</td><td>(R)G Y|M|F|T|T|T|A|E\R(E)</td><td>588.6464</td><td>1176.535</td><td>-0.2499</td></tr>
<tr><td></td><td></td><td></td><td></td><td></td><td></td><td>2</td><td>16.42</td><td>4.96</td><td>88</td><td>(K)N Y|E|L|P D G Q V/I|T I|G A E R(F)</td><td>888.0171</td><td>1774.897</td><td>0.1299</td></tr>
<tr><td></td><td></td><td></td><td></td><td></td><td></td><td>2</td><td>16.29</td><td>6.1</td><td>91.4</td><td>(K)I/W/H|H|T|F|Y/N E\L\R(V)</td><td>758.6287</td><td>1515.749</td><td>0.501</td></tr>
<tr><td></td><td></td><td></td><td></td><td></td><td></td><td>3</td><td>15.9</td><td>4.66</td><td>94.2</td><td>(K)G E|Y|D/E|S/G/P S I/V H R(K)</td><td>482.7145</td><td>1445.666</td><td>0.4634</td></tr>
<tr><td></td><td></td><td></td><td></td><td></td><td></td><td>2</td><td>14.47</td><td>7.48</td><td>91.2</td><td>(R)G Y/m|F|T|T/T/A\E\R(E)</td><td>596.9045</td><td>1176.535</td><td>16.2663</td></tr>
<tr><td></td><td></td><td></td><td></td><td></td><td></td><td>2</td><td>14.44</td><td>4.3</td><td>97.5</td><td>(K)N Y\E|L|P/D G Q V|I|T\I|G A E R(F)</td><td>887.959</td><td>1774.897</td><td>0.0137</td></tr>
<tr><td></td><td></td><td></td><td></td><td></td><td></td><td>2</td><td>14.26</td><td>8.03</td><td>90.4</td><td>(K)G E/Y/D/E|S|G/P S I|V/H R(K)</td><td>723.3845</td><td>1445.666</td><td>0.0962</td></tr>
<tr><td></td><td></td><td></td><td></td><td></td><td></td><td>3</td><td>14.14</td><td>5.48</td><td>74.1</td><td>(K)Y/P/I E/H G\I|V|S|N/W/D D M E K(I)</td><td>645.262</td><td>1932.88</td><td>0.8918</td></tr>
<tr><td></td><td></td><td></td><td></td><td></td><td></td><td>3</td><td>13.93</td><td>3.26</td><td>84.8</td><td>(R)V A/P E/E H P V L/L|T|E A|P L N\P K(A)</td><td>652.2522</td><td>1954.064</td><td>0.6776</td></tr>
<tr><td></td><td></td><td></td><td></td><td></td><td></td><td>2</td><td>13.66</td><td>4.14</td><td>87.3</td><td>(K)E/I|T/A/L|A|P S S M K(I)</td><td>574.4629</td><td>1147.603</td><td>0.3158</td></tr>
<tr><td></td><td></td><td></td><td></td><td></td><td></td><td>2</td><td>13.62</td><td>4.94</td><td>89.2</td><td>(K)A/G/F|A|G|D|D|A\P R(A)</td><td>488.8138</td><td>976.448</td><td>0.172</td></tr>
<tr><td></td><td></td><td></td><td></td><td></td><td></td><td>2</td><td>13.5</td><td>4.15</td><td>100</td><td>(K)N Y\E|L|P D G Q V|I|T I|G A E R(F)</td><td>888.4803</td><td>1774.897</td><td>1.0563</td></tr>
<tr><td></td><td></td><td></td><td></td><td></td><td></td><td>2</td><td>13.05</td><td>4.33</td><td>93.4</td><td>(K)A G/F|A|G|D|D|A|P R(A)</td><td>488.969</td><td>976.448</td><td>0.4824</td></tr>
<tr><td></td><td></td><td></td><td></td><td></td><td></td><td>3</td><td>12.76</td><td>5.69</td><td>77</td><td>(K)Y/P/I E\H G|I|V|S/N/W/D/D M E K(I)</td><td>645.3176</td><td>1932.88</td><td>1.0586</td></tr>
<tr><td></td><td></td><td></td><td></td><td></td><td></td><td>2</td><td>12</td><td>5.51</td><td>100</td><td>(K)A G/F|A|G/D|D|A\P R(A)</td><td>488.8546</td><td>976.448</td><td>0.2536</td></tr>
<tr><td></td><td></td><td></td><td></td><td></td><td></td><td>2</td><td>11.98</td><td>4.28</td><td>93.4</td><td>(K)E/I/T/A|L|A|P S S M K(I)</td><td>574.1965</td><td>1147.603</td><td>-0.217</td></tr>
<tr><td></td><td></td><td></td><td></td><td></td><td></td><td>2</td><td>11.67</td><td>2.84</td><td>85.8</td><td>(K)E I|T/A/L|A\P/S S m\K(I)</td><td>582.6804</td><td>1147.603</td><td>16.7508</td></tr>
<tr><td></td><td></td><td></td><td></td><td></td><td></td><td>2</td><td>11</td><td>4.29</td><td>100</td><td>(K)L A Y V|A|L|D/Y/E/Q E L\E\T A K(S)</td><td>928.0634</td><td>1855.932</td><td>-0.8129</td></tr>
<tr><td></td><td></td><td></td><td></td><td></td><td></td><td>3</td><td>10.83</td><td>4.22</td><td>85.9</td><td>(R)V A/P E E H P V L/L|T|E A|P L N\P K(A)</td><td>652.2158</td><td>1954.064</td><td>0.5684</td></tr>
<tr><td></td><td></td><td></td><td></td><td></td><td></td><td>2</td><td>10.51</td><td>5.65</td><td>95.8</td><td>(K)L A Y V|A/L|D/Y/E\Q E L E\T A K(S)</td><td>929.4071</td><td>1855.932</td><td>1.8745</td></tr>
<tr><td></td><td></td><td></td><td></td><td></td><td></td><td>3</td><td>10.46</td><td>6.15</td><td>80.2</td><td>(K)Y/P/I E H G\I|V|S N/W/D\D\M E K(I)</td><td>645.1394</td><td>1932.88</td><td>0.524</td></tr>
<tr><td></td><td></td><td></td><td></td><td></td><td></td><td>2</td><td>10.41</td><td>2.39</td><td>85.7</td><td>(K)A G/F|A|G\D/D/A|P R(A)</td><td>488.8149</td><td>976.448</td><td>0.1742</td></tr>
<tr><td></td><td></td><td></td><td></td><td></td><td></td><td>2</td><td>10.07</td><td>4.58</td><td>92.8</td><td>(K)L A Y V A|L|D|Y/E\Q E L\E\T A K(S)</td><td>928.527</td><td>1855.932</td><td>0.1143</td></tr>
<tr><td>1677</td><td>XP_010649306.1</td><td>Protein YLS2-like
(Vitis vinifera)</td><td>3(3)</td><td>9%</td><td>39.77</td><td>2</td><td>15.41</td><td>5.75</td><td>91.8</td><td>(K)G L/L/E|V|T A|D|G M\V\K(T)</td><td>616.9595</td><td>1232.656</td><td>0.2562</td></tr>
<tr><td></td><td></td><td></td><td></td><td></td><td></td><td>2</td><td>12.96</td><td>2.9</td><td>76.6</td><td>(R)M/L/Q/G/S/E M/I|G\V|G K(L)</td><td>625.2822</td><td>1249.628</td><td>-0.0708</td></tr>
<tr><td></td><td></td><td></td><td></td><td></td><td></td><td>2</td><td>11.4</td><td>2.43</td><td>81.3</td><td>(R)L/M|S|F|D|P S T E E T K(V)</td><td>693.7285</td><td>1384.63</td><td>1.8196</td></tr>
<tr><td>1679</td><td>XP_002276099.1</td><td>DNA damage-inducible protein 1
(Vitis vinifera)</td><td>3(4)</td><td>11%</td><td>55.96</td><td>2</td><td>22</td><td>10.57</td><td>100</td><td>(K)A F|V/D|S|G/A|Q|S|T|I|I\S\K(S)</td><td>712.1676</td><td>1423.743</td><td>-0.4148</td></tr>
<tr><td></td><td></td><td></td><td></td><td></td><td></td><td>2</td><td>18.3</td><td>9.69</td><td>92.1</td><td>(R)V/P L Q Q/Q|Q|L|L|F|N|G Q E M R(N)</td><td>965.7574</td><td>1929.001</td><td>1.5064</td></tr>
<tr><td></td><td></td><td></td><td></td><td></td><td></td><td>2</td><td>15.83</td><td>7.99</td><td>94.4</td><td>(R)V/P L Q/Q/Q/Q/L|L|F|N|G Q\E M R(N)</td><td>965.2316</td><td>1929.001</td><td>0.4548</td></tr>
<tr><td></td><td></td><td></td><td></td><td></td><td></td><td>3</td><td>15.66</td><td>5.2</td><td>84.1</td><td>(R)G/I A H G V\G Q|S|E|I|L|G\R(I)</td><td>465.4913</td><td>1393.755</td><td>0.7047</td></tr>
<tr><td>1715</td><td>XP_002278444.1</td><td>formate dehydrogenase, mitochondrial
(Vitis vinifera)</td><td>2(3)</td><td>6%</td><td>36.08</td><td>2</td><td>19.37</td><td>9.82</td><td>97.6</td><td>(K)F/E|E|D|V|D|V|m|L|P\K(C)</td><td>670.1204</td><td>1321.634</td><td>17.5991</td></tr>
<tr><td></td><td></td><td></td><td></td><td></td><td></td><td>2</td><td>17.77</td><td>6.02</td><td>93.5</td><td>(K)F/E|E|D|V|D|V|M|L|P K(C)</td><td>660.9661</td><td>1321.634</td><td>-0.7095</td></tr>
<tr><td></td><td></td><td></td><td></td><td></td><td></td><td>3</td><td>16.71</td><td>6.5</td><td>95.4</td><td>(R)I/K m/D|P E|L|E|N/Q|I\G A K(F)</td><td>535.1155</td><td>1585.825</td><td>17.5065</td></tr>
<tr><td>1720</td><td>CAC39216.1</td><td>glutamine synthetase
(Vitis vinifera)</td><td>3(3)</td><td>13%</td><td>51.23</td><td>2</td><td>17.73</td><td>5.74</td><td>94.5</td><td>(K)I I/A E/Y|I|W I|G|G|S|G I D/L\R(S)</td><td>889.1963</td><td>1775.969</td><td>1.4163</td></tr>
<tr><td></td><td></td><td></td><td></td><td></td><td></td><td>2</td><td>17.31</td><td>7.41</td><td>91.5</td><td>(R)T L/S/G/P V|T|G|P\A\Q|L\P K(W)</td><td>683.7253</td><td>1365.774</td><td>0.6697</td></tr>
<tr><td></td><td></td><td></td><td></td><td></td><td></td><td>2</td><td>16.19</td><td>8.8</td><td>91.1</td><td>(R)I T E I/A/G/V|V|V|S/F/D|P K/P I K(G)</td><td>907.4219</td><td>1813.047</td><td>0.7896</td></tr>
<tr><td>1726</td><td>XP_002278444.1</td><td>Formate dehydrogenase, mitochondrial
(Vitis vinifera)</td><td>4(4)</td><td>10%</td><td>60.27</td><td>3</td><td>20.12</td><td>6.51</td><td>95.4</td><td>(R)I/K/m/D|P E|L|E|N/Q|I\G A\K(F)</td><td>534.864</td><td>1585.825</td><td>16.752</td></tr>
<tr><td></td><td></td><td></td><td></td><td></td><td></td><td>2</td><td>13.92</td><td>3.14</td><td>89.1</td><td>(K)G V|L|I|V N|N|A R(G)</td><td>478.3829</td><td>955.568</td><td>0.1902</td></tr>
<tr><td></td><td></td><td></td><td></td><td></td><td></td><td>3</td><td>13.75</td><td>3.52</td><td>100</td><td>(R)D W|L|E|S Q|G\H Q Y|I/V T D D K(E)</td><td>645.7191</td><td>1933.893</td><td>1.2501</td></tr>
<tr><td></td><td></td><td></td><td></td><td></td><td></td><td>2</td><td>12.48</td><td>4.78</td><td>99</td><td>(K)M/D/P/E/L|E|N/Q I|G A K(F)</td><td>672.2556</td><td>1344.646</td><td>-1.1425</td></tr>
<tr><td>1731</td><td>CAC39216.1</td><td>Glutamine synthetase
(Vitis vinifera)</td><td>6(14)</td><td>34%</td><td>99.4</td><td>2</td><td>18.78</td><td>8.04</td><td>94.5</td><td>(K)W N Y D/G S S T G Q|A|P G E/D|S E/V/I|L/Y/P Q A I F K(D)</td><td>1486.9746</td><td>2972.395</td><td>0.5471</td></tr>
<tr><td></td><td></td><td></td><td></td><td></td><td></td><td>2</td><td>18.36</td><td>8.44</td><td>93.2</td><td>(K)I I/A E/Y|I|W I|G|G|S|G N/D|L\R(S)</td><td>889.3564</td><td>1776.928</td><td>0.7776</td></tr>
<tr><td></td><td></td><td></td><td></td><td></td><td></td><td>2</td><td>18.24</td><td>8.16</td><td>94.6</td><td>(R)T L/S/G/P/V/T|G|P\A\Q/L\P K(W)</td><td>684.1069</td><td>1365.774</td><td>1.4329</td></tr>
<tr><td></td><td></td><td></td><td></td><td></td><td></td><td>2</td><td>18.19</td><td>7.91</td><td>94</td><td>(R)I T E I/A/G/V|V|V|S|F/D|P K/P I K(G)</td><td>907.3999</td><td>1813.047</td><td>0.7456</td></tr>
<tr><td></td><td></td><td></td><td></td><td></td><td></td><td>2</td><td>16.63</td><td>5.59</td><td>90.8</td><td>(K)W N/Y D G S S T G Q|A|P/G E/D|S E/V/I|L/Y/P Q A I F K(D)</td><td>1487.1033</td><td>2972.395</td><td>0.8045</td></tr>
<tr><td></td><td></td><td></td><td></td><td></td><td></td><td>2</td><td>14.54</td><td>3.86</td><td>86.2</td><td>(K)W N Y D/G S S T G Q/A|P/G E\D/S E V|I|L/Y/P Q A I F K(D)</td><td>1486.9209</td><td>2972.395</td><td>0.4397</td></tr>
<tr><td></td><td></td><td></td><td></td><td></td><td></td><td>2</td><td>14.49</td><td>4.85</td><td>81.4</td><td>(R)I T E/I/A/G V|V|V|S F D|P K/P I K(G)</td><td>907.7666</td><td>1813.047</td><td>1.479</td></tr>
<tr><td></td><td></td><td></td><td></td><td></td><td></td><td>3</td><td>14.46</td><td>5.34</td><td>70.8</td><td>(K)I F S H P E V T A E E|P W Y|G\I|E/Q|E/Y\T\L|L\Q\K(D)</td><td>1003.2104</td><td>3007.472</td><td>0.1443</td></tr>
<tr><td></td><td></td><td></td><td></td><td></td><td></td><td>2</td><td>13.94</td><td>3.54</td><td>88.9</td><td>(K)I I/A E Y|I|W I|G|G|S|G N D L\R(S)</td><td>889.5542</td><td>1776.928</td><td>1.1732</td></tr>
<tr><td></td><td></td><td></td><td></td><td></td><td></td><td>3</td><td>13.39</td><td>5.94</td><td>87.1</td><td>(K)I F S H P E V T A E E|P W Y|G\I|E Q E|Y T\L|L\Q\K(D)</td><td>1003.6715</td><td>3007.472</td><td>1.5276</td></tr>
<tr><td></td><td></td><td></td><td></td><td></td><td></td><td>2</td><td>12.4</td><td>3.54</td><td>82.8</td><td>(R)I/T E I/A/G V|V|V|S/F D|P K P I K(G)</td><td>907.345</td><td>1813.047</td><td>0.6358</td></tr>
<tr><td></td><td></td><td></td><td></td><td></td><td></td><td>2</td><td>12.29</td><td>4.84</td><td>95.3</td><td>(R)T L/S/G/P V/T|G/P\A\Q\L\P K(W)</td><td>684.012</td><td>1365.774</td><td>1.2431</td></tr>
<tr><td></td><td></td><td></td><td></td><td></td><td></td><td>2</td><td>11.6</td><td>5.69</td><td>81.8</td><td>(R)I T E I/A/G/V/V|V|S/F D|P K P I\K(G)</td><td>907.1097</td><td>1813.047</td><td>0.1652</td></tr>
<tr><td></td><td></td><td></td><td></td><td></td><td></td><td>2</td><td>11.37</td><td>4.36</td><td>74.6</td><td>(R)G G D/N I/L V\m C/D/A/Y/T/P G G E/P I/P T N K R(F)</td><td>1295.3413</td><td>2575.228</td><td>14.4474</td></tr>
<tr><td>1765</td><td>XP_002263490.1</td><td>UDP-arabinopyranose mutase 1</td><td>4(4)</td><td>12%</td><td>55.21</td><td>2</td><td>14.98</td><td>5.73</td><td>99.1</td><td>(R)Y V|D|A|V|M|T|I|P K(G)</td><td>569.2224</td><td>1136.602</td><td>0.8355</td></tr>
<tr><td></td><td></td><td></td><td></td><td></td><td></td><td>2</td><td>14.67</td><td>3.9</td><td>94.5</td><td>(K)D/I/N|A|L/E/Q/H\I\K(N)</td><td>591.2188</td><td>1180.632</td><td>0.7983</td></tr>
<tr><td></td><td></td><td></td><td></td><td></td><td></td><td>2</td><td>14.67</td><td>5.22</td><td>86.8</td><td>(R)V/P/D/G/F/D|Y/E|L|Y N R(N)</td><td>744.7465</td><td>1487.68</td><td>0.8056</td></tr>
</table>

<table>
<tr><td></td><td></td><td></td><td></td><td></td><td></td><td>3</td><td>10.89</td><td>2.78</td><td>94.4</td><td>(K)T/G|L|P Y|I/W H S K(A)</td><td>401.6476</td><td>1201.636</td><td>1.2918</td></tr>
<tr><td>1767</td><td></td><td>Phosphoglycerate kinase, cytosolic</td><td>16(52)</td><td>52%</td><td>271.39</td><td>2</td><td>24</td><td>17.42</td><td>100</td><td>(K)A Q/G/Y/G/V/G/S/S/L/V|E/E/D|K|L|D|L|A/T/S L L E K(A)</td><td>1312.2202</td><td>2622.351</td><td>1.0823</td></tr>
<tr><td></td><td></td><td>(Vitis vinifera)</td><td></td><td></td><td></td><td>2</td><td>22.5</td><td>13.27</td><td>100</td><td>(K)M/S/H|I|S|T|G/G|G|A S L/E|L\L\E\G K(T)</td><td>894.2871</td><td>1786.9</td><td>0.6665</td></tr>
<tr><td></td><td></td><td></td><td></td><td></td><td></td><td>2</td><td>20.36</td><td>10.38</td><td>98</td><td>(K)T I/I/W|N|G|P/M/G|V|F/E|F D\K(F)</td><td>878.1138</td><td>1753.862</td><td>1.3585</td></tr>
<tr><td></td><td></td><td></td><td></td><td></td><td></td><td>2</td><td>20.31</td><td>10.46</td><td>95.6</td><td>(K)G V T T I|I|G|G/G/D\S V|A|A|V|E\K(V)</td><td>787.2366</td><td>1573.843</td><td>-0.3773</td></tr>
<tr><td></td><td></td><td></td><td></td><td></td><td></td><td>2</td><td>19.58</td><td>9.71</td><td>95.6</td><td>(K)m S/H|I|S|T/G/G/G A|S L\E|L\L E\G K(T)</td><td>902.1323</td><td>1786.9</td><td>16.3569</td></tr>
<tr><td></td><td></td><td></td><td></td><td></td><td></td><td>3</td><td>18.84</td><td>8.21</td><td>95.8</td><td>(K)A/Q/G/Y/G/V|G/S/S/L/V/E E D K|L/D L/A/T S L/L E K(A)</td><td>875.3584</td><td>2622.351</td><td>1.7098</td></tr>
<tr><td></td><td></td><td></td><td></td><td></td><td></td><td>2</td><td>18.71</td><td>7.2</td><td>93.9</td><td>(K)I/P/E|G/G|V|L|L|L|E|N V R(F)</td><td>705.162</td><td>1408.816</td><td>0.5009</td></tr>
<tr><td></td><td></td><td></td><td></td><td></td><td></td><td>2</td><td>18.67</td><td>6.9</td><td>93.6</td><td>(K)I/P/E|G/G/V|L|L|L|E|N V R(F)</td><td>705.2007</td><td>1408.816</td><td>0.5783</td></tr>
<tr><td></td><td></td><td></td><td></td><td></td><td></td><td>2</td><td>18.16</td><td>7.03</td><td>93.5</td><td>(K)I/P/E|G/G/V|L|L|L|E|N V\R(F)</td><td>705.1632</td><td>1408.816</td><td>0.5033</td></tr>
<tr><td></td><td></td><td></td><td></td><td></td><td></td><td>2</td><td>18.09</td><td>7.53</td><td>93.8</td><td>(K)L A/S/I/A/D|L|Y|V/N/D|A/F/G T A H\R(A)</td><td>967.7035</td><td>1933.977</td><td>0.4231</td></tr>
<tr><td></td><td></td><td></td><td></td><td></td><td></td><td>2</td><td>18.05</td><td>8.72</td><td>96.7</td><td>(K)E L/D|Y|L|V|G|A|V|S|N\P K(R)</td><td>703.0046</td><td>1404.737</td><td>0.265</td></tr>
<tr><td></td><td></td><td></td><td></td><td></td><td></td><td>2</td><td>18.05</td><td>8.49</td><td>96.7</td><td>(K)E L D|Y/L|V|G|A|V|S|N\P K(R)</td><td>703.054</td><td>1404.737</td><td>0.3638</td></tr>
<tr><td></td><td></td><td></td><td></td><td></td><td></td><td>2</td><td>18.03</td><td>6.38</td><td>91.2</td><td>(K)I/P/E|G/G/V|L|L|L|E|N V R(F)</td><td>704.9332</td><td>1408.816</td><td>0.0433</td></tr>
<tr><td></td><td></td><td></td><td></td><td></td><td></td><td>2</td><td>17.5</td><td>7.83</td><td>90.2</td><td>(K)G V T\T I|I|G|G|G/D S V/A\A\V\E K(V)</td><td>787.2986</td><td>1573.843</td><td>-0.2533</td></tr>
<tr><td></td><td></td><td></td><td></td><td></td><td></td><td>2</td><td>17.4</td><td>7.25</td><td>89.5</td><td>(K)G V T T I|I|G|G|G D\S/V/A|A V\E\K(V)</td><td>787.4119</td><td>1573.843</td><td>-0.0267</td></tr>
<tr><td></td><td></td><td></td><td></td><td></td><td></td><td>2</td><td>17.32</td><td>8.01</td><td>92.7</td><td>(K)G V T T I/I|G|G/G/D|S V\A\A\V E\K(V)</td><td>787.5146</td><td>1573.843</td><td>0.1787</td></tr>
<tr><td></td><td></td><td></td><td></td><td></td><td></td><td>2</td><td>17.2</td><td>4.29</td><td>96.7</td><td>(K)Y L|K|P/S/V|A\G/F|L|M\Q\K(E)</td><td>742.0215</td><td>1481.819</td><td>1.2172</td></tr>
<tr><td></td><td></td><td></td><td></td><td></td><td></td><td>2</td><td>17.19</td><td>4.11</td><td>96.7</td><td>(K)Y/S|L|K|P/L/V|P R(L)</td><td>537.1284</td><td>1072.651</td><td>0.5982</td></tr>
<tr><td></td><td></td><td></td><td></td><td></td><td></td><td>3</td><td>17.1</td><td>7.59</td><td>93.1</td><td>(K)Y L/K|P S V|A|G|F|L|M Q\K(E)</td><td>494.5583</td><td>1481.819</td><td>-0.1581</td></tr>
<tr><td></td><td></td><td></td><td></td><td></td><td></td><td>2</td><td>16.69</td><td>5.84</td><td>86.3</td><td>(K)I/P/E/G/G|V|L|L|L|E|N V R(F)</td><td>704.7485</td><td>1408.816</td><td>-0.3261</td></tr>
<tr><td></td><td></td><td></td><td></td><td></td><td></td><td>3</td><td>16.45</td><td>7.22</td><td>83.1</td><td>(K)L/A/S/I/A/D/L/Y/V/N/D/A F/G T\A H R(A)</td><td>645.6172</td><td>1933.977</td><td>0.8604</td></tr>
<tr><td></td><td></td><td></td><td></td><td></td><td></td><td>3</td><td>16.15</td><td>4.03</td><td>87</td><td>(K)M S/H I S T G/G G A|S/L|E|L|L|E\G K(T)</td><td>596.5164</td><td>1786.9</td><td>0.6343</td></tr>
<tr><td></td><td></td><td></td><td></td><td></td><td></td><td>3</td><td>16.04</td><td>6.08</td><td>87.5</td><td>(K)A Q/G/Y/G/V/G/S/S L|V E\E D/K/L/D L/A/T S L L\E K(A)</td><td>875.0103</td><td>2622.351</td><td>0.6655</td></tr>
<tr><td></td><td></td><td></td><td></td><td></td><td></td><td>2</td><td>16.04</td><td>7.73</td><td>91.9</td><td>(K)E L D|Y/L|V|G|A|V|S|N\P K(R)</td><td>703.5234</td><td>1404.737</td><td>1.3026</td></tr>
<tr><td></td><td></td><td></td><td></td><td></td><td></td><td>3</td><td>15.94</td><td>5.57</td><td>95.1</td><td>(K)Y L|K|P S V|A|G|F/L|M\Q\K(E)</td><td>494.9911</td><td>1481.819</td><td>1.1403</td></tr>
<tr><td></td><td></td><td></td><td></td><td></td><td></td><td>2</td><td>15.56</td><td>6</td><td>90.8</td><td>(K)E L/D|Y|L|V|G/A/V|S|N\P K(R)</td><td>702.9458</td><td>1404.737</td><td>0.1474</td></tr>
<tr><td></td><td></td><td></td><td></td><td></td><td></td><td>2</td><td>15.19</td><td>5.17</td><td>96.9</td><td>(K)T/F|G|E|S|L/D|T/T K(T)</td><td>549.9683</td><td>1098.531</td><td>0.398</td></tr>
<tr><td></td><td></td><td></td><td></td><td></td><td></td><td>3</td><td>14.86</td><td>5.16</td><td>95.3</td><td>(K)A/Q/G/Y|G/V/G/S/S/L/V/E E D K/L/D L A/T/S L L E K(A)</td><td>874.6824</td><td>2622.351</td><td>-0.3182</td></tr>
<tr><td></td><td></td><td></td><td></td><td></td><td></td><td>2</td><td>14.61</td><td>4.89</td><td>87.5</td><td>(K)I/P/E/G G/V|L|L|L|E/N V\R(F)</td><td>705.3197</td><td>1408.816</td><td>0.8163</td></tr>
<tr><td></td><td></td><td></td><td></td><td></td><td></td><td>2</td><td>14.5</td><td>2.89</td><td>88.5</td><td>(R)L/S|E/L|L|G|V|E V\K(M)</td><td>544.1655</td><td>1086.641</td><td>0.6832</td></tr>
<tr><td></td><td></td><td></td><td></td><td></td><td></td><td>2</td><td>14.5</td><td>6.71</td><td>100</td><td>(K)E L D|Y/L|V|G|A\V|S|N\P K(R)</td><td>703.355</td><td>1404.737</td><td>0.9658</td></tr>
<tr><td></td><td></td><td></td><td></td><td></td><td></td><td>2</td><td>14.24</td><td>5.71</td><td>93</td><td>(K)I/G|V|I|E S/L|L|G K(V)</td><td>515.0801</td><td>1028.635</td><td>0.5179</td></tr>
<tr><td></td><td></td><td></td><td></td><td></td><td></td><td>2</td><td>14.18</td><td>6.91</td><td>95</td><td>(K)E L D|Y/L|V|G/A|V|S|N\P K(R)</td><td>702.7715</td><td>1404.737</td><td>-0.2012</td></tr>
<tr><td></td><td></td><td></td><td></td><td></td><td></td><td>3</td><td>13.91</td><td>10.6</td><td>76.6</td><td>(K)K/L/A S I|A D L/Y|V|N/D/A F/G/T A H R(A)</td><td>688.6218</td><td>2062.072</td><td>1.7792</td></tr>
<tr><td></td><td></td><td></td><td></td><td></td><td></td><td>3</td><td>13.81</td><td>5.47</td><td>80.3</td><td>(R)F/Y|K/E E|E\K|N/D P\E|F A\K(K)</td><td>591.647</td><td>1773.833</td><td>-0.9066</td></tr>
<tr><td></td><td></td><td></td><td></td><td></td><td></td><td>2</td><td>13.73</td><td>4.75</td><td>94.6</td><td>(K)E L D|Y/L|V|G/A|V|S|N\P K(R)</td><td>702.8729</td><td>1404.737</td><td>0.0016</td></tr>
<tr><td></td><td></td><td></td><td></td><td></td><td></td><td>2</td><td>13.45</td><td>6.07</td><td>76</td><td>(R)L/S|E|L|L|G\V|E\V\K(M)</td><td>544.0564</td><td>1086.641</td><td>0.465</td></tr>
<tr><td></td><td></td><td></td><td></td><td></td><td></td><td>2</td><td>13.37</td><td>3.91</td><td>84.3</td><td>(K)I/P/E|G G V|L|L|L|E N V\R(F)</td><td>705.0353</td><td>1408.816</td><td>0.2475</td></tr>
<tr><td></td><td></td><td></td><td></td><td></td><td></td><td>3</td><td>13.18</td><td>2.13</td><td>78.7</td><td>(R)F/Y/K/E\E|E\K|N/D P E|F A\K(K)</td><td>592.2628</td><td>1773.833</td><td>0.9408</td></tr>
<tr><td></td><td></td><td></td><td></td><td></td><td></td><td>2</td><td>12.99</td><td>4.7</td><td>88.1</td><td>(K)E L D|Y/L|V|G|A/V|S|N P K(R)</td><td>703.0857</td><td>1404.737</td><td>0.4272</td></tr>
<tr><td></td><td></td><td></td><td></td><td></td><td></td><td>3</td><td>12.8</td><td>6.12</td><td>90.9</td><td>(K)Y L/K\P S V|A|G|F|L/m\Q K(E)</td><td>500.3162</td><td>1481.819</td><td>17.1156</td></tr>
<tr><td></td><td></td><td></td><td></td><td></td><td></td><td>2</td><td>12.3</td><td>2.47</td><td>84.6</td><td>(K)I/P/E/G G\V|L|L|L|E N V R(F)</td><td>705.6692</td><td>1408.816</td><td>1.5153</td></tr>
<tr><td></td><td></td><td></td><td></td><td></td><td></td><td>2</td><td>12.24</td><td>5.74</td><td>95.9</td><td>(K)E L D|Y/L|V|G/A/V|S|N\P K(R)</td><td>703.4231</td><td>1404.737</td><td>1.102</td></tr>
<tr><td></td><td></td><td></td><td></td><td></td><td></td><td>2</td><td>12.12</td><td>5.29</td><td>85</td><td>(K)A Q/G Y/G V G S S/L/V/E\E/D|K|L D|L\A T S L L E K(A)</td><td>1311.0879</td><td>2622.351</td><td>-1.1823</td></tr>
<tr><td></td><td></td><td></td><td></td><td></td><td></td><td>2</td><td>11.71</td><td>3.19</td><td>89.5</td><td>(K)I/P/E/G G/V|L|L/L|E\N V R(F)</td><td>705.374</td><td>1408.816</td><td>0.9249</td></tr>
<tr><td></td><td></td><td></td><td></td><td></td><td></td><td>2</td><td>11.7</td><td>5.85</td><td>81.8</td><td>(K)R/P/F A A I|V|G|G\S\K(V)</td><td>552.5391</td><td>1102.637</td><td>1.4342</td></tr>
<tr><td></td><td></td><td></td><td></td><td></td><td></td><td>2</td><td>11.63</td><td>2.93</td><td>83.3</td><td>(K)T L/P G V|L|A|L|D D\A(-)</td><td>543.0903</td><td>1084.589</td><td>0.5849</td></tr>
<tr><td></td><td></td><td></td><td></td><td></td><td></td><td>2</td><td>11.37</td><td>3.9</td><td>76.6</td><td>(K)G V T T I|I|G/G/G\D S/V\A A\V\E K(V)</td><td>787.5593</td><td>1573.843</td><td>0.2681</td></tr>
<tr><td></td><td></td><td></td><td></td><td></td><td></td><td>2</td><td>11.35</td><td>2.41</td><td>70.2</td><td>(K)I/P/E/G G/V|L|L/L|E N V R(F)</td><td>705.0051</td><td>1408.816</td><td>0.1871</td></tr>
<tr><td></td><td></td><td></td><td></td><td></td><td></td><td>3</td><td>11.17</td><td>2.48</td><td>85.6</td><td>(K)M S H I S T G G G A|S|L/E/L|L|E\G K(T)</td><td>596.9399</td><td>1786.9</td><td>1.9048</td></tr>
<tr><td></td><td></td><td></td><td></td><td></td><td></td><td>3</td><td>10.77</td><td>3.06</td><td>75.4</td><td>(K)Y L/K\P S V|A|G|F|L/m\Q K(E)</td><td>500.3334</td><td>1481.819</td><td>17.1672</td></tr>
<tr><td></td><td></td><td></td><td></td><td></td><td></td><td>2</td><td>10.61</td><td>3.46</td><td>79.5</td><td>(R)L/S/E|L|L|G|V\E V K(M)</td><td>544.1997</td><td>1086.641</td><td>0.7516</td></tr>
<tr><td>1779</td><td>XP_002283381.1</td><td>Fructose-bisphosphate aldolase cytoplasmic isozyme</td><td>2(2)</td><td>7%</td><td>32.34</td><td>2</td><td>18.44</td><td>13.06</td><td>89.4</td><td>(K)G I/L|A|A|D|E S|T/G\T/I|G K(R)</td><td>667.1661</td><td>1332.7</td><td>0.6244</td></tr>
<tr><td></td><td></td><td>(Vitis vinifera)</td><td></td><td></td><td></td><td>3</td><td>13.9</td><td>5.17</td><td>90.8</td><td>(K)K V S\P E|V\V|A|E/Y|T\V\R(T)</td><td>493.2299</td><td>1476.806</td><td>0.8695</td></tr>
<tr><td>1784</td><td>XP_002282603.1</td><td>Probable protein disulfide-isomerase A6</td><td>7(14)</td><td>23%</td><td>125.29</td><td>3</td><td>20.23</td><td>5.44</td><td>99.2</td><td>(R)I/E|E|E|V|E|K|L|E|G S A A R(Y)</td><td>520.7537</td><td>1559.791</td><td>0.4554</td></tr>
<tr><td></td><td></td><td>(Vitis vinifera)</td><td></td><td></td><td></td><td>3</td><td>20.18</td><td>6.95</td><td>96</td><td>(R)S A E A/L|A|E|F\V|N/N/E|G/G T\N V K(I)</td><td>617.3269</td><td>1849.893</td><td>0.0735</td></tr>
<tr><td></td><td></td><td></td><td></td><td></td><td></td><td>3</td><td>19.71</td><td>9.15</td><td>97.6</td><td>(K)S/E|G/D|V/V/I/A/N L/D/A D\K Y K(D)</td><td>580.2524</td><td>1736.87</td><td>1.8725</td></tr>
<tr><td></td><td></td><td></td><td></td><td></td><td></td><td>2</td><td>19.28</td><td>11.02</td><td>96</td><td>(K)S E/G D|V/V|I|A|N L|D|A/D/K Y K(D)</td><td>869.7438</td><td>1736.87</td><td>1.6102</td></tr>
<tr><td></td><td></td><td></td><td></td><td></td><td></td><td>2</td><td>18.08</td><td>6.78</td><td>92.6</td><td>(K)S E/G D/V|V|I/A/N L|D|A D|K Y\K(D)</td><td>869.2614</td><td>1736.87</td><td>0.6454</td></tr>
<tr><td></td><td></td><td></td><td></td><td></td><td></td><td>2</td><td>17.83</td><td>7.22</td><td>92.1</td><td>(R)I/E/E/E|V|E|K|L|E|G S A A R(Y)</td><td>780.3535</td><td>1559.791</td><td>-0.0914</td></tr>
<tr><td></td><td></td><td></td><td></td><td></td><td></td><td>2</td><td>17.61</td><td>6.93</td><td>93.7</td><td>(K)Y G/V/S/G|Y|P|T I|Q/W|F\P K(G)</td><td>822.3843</td><td>1642.826</td><td>0.9349</td></tr>
<tr><td></td><td></td><td></td><td></td><td></td><td></td><td>2</td><td>17.01</td><td>6.47</td><td>95.5</td><td>(K)S E/G/D|V|V|I/A|N/L|D A D\K(Y)</td><td>723.4799</td><td>1445.712</td><td>0.2407</td></tr>
<tr><td></td><td></td><td></td><td></td><td></td><td></td><td>2</td><td>16.95</td><td>5.6</td><td>92.8</td><td>(K)A G/T/V|A|S/L|D/S L V\K(E)</td><td>581.1464</td><td>1160.652</td><td>0.6334</td></tr>
<tr><td></td><td></td><td></td><td></td><td></td><td></td><td>2</td><td>14.82</td><td>5.09</td><td>91.5</td><td>(K)Y G V/S|G|Y|P T I/Q/W|F\P K(G)</td><td>822.1816</td><td>1642.826</td><td>0.5295</td></tr>
<tr><td></td><td></td><td></td><td></td><td></td><td></td><td>2</td><td>14.77</td><td>5.48</td><td>82.1</td><td>(R)S A E A L\A|E|F|V/N|N|E|G G T N V K(I)</td><td>925.7402</td><td>1849.893</td><td>0.5805</td></tr>
</table>

						2	13.6	3.51	86.4	(K)Y/G|V|S|G|Y|P T L\K(F)	543.243	1084.567	0.9114
						2	11.63	3.1	74.4	(R)S A E A L A/E/F|V N|N/E/G G\T N\V\K(I)	925.7336	1849.893	0.5673
						2	11.5	4.08	100	(R)S A E A/L/A E|F/V/N|N\E G G T\N V\K(I)	926.1949	1849.893	1.4899
1801	XP_002284459.1	**11S globulin subunit beta**	4(8)	16%	64.49	2	17.99	8.96	96.3	(K)V A/Y V L/Q/G N/G V|A|G I|V|L|P E\S E E K(V)	1087.0769	2172.155	0.9918
		(Vitis vinifera)				2	17.09	10.15	92.7	(K)V L/E/A/S/F|S/V|G/S D\M E\K(L)	749.678	1498.709	-0.3607
						2	16.34	3.15	94.5	(R)A/W|D|L|E|E|D V\V\K(S)	602.2012	1203.589	-0.1941
						2	13.5	4.86	100	(K)V A/Y V L Q/G N/G V|A|G I|V|L|P E S E E K(V)	1086.8906	2172.155	0.6192
						2	13.07	3.19	94.5	(K)L E|G/T/F/E|M|P E\P K(K)	639.2832	1277.608	-0.0491
						2	12.5	5.31	100	(K)V A/Y V L Q/G/N G V|A/G I|V|L|P E S E E K(V)	1087.1958	2172.155	1.2296
						2	12.07	3.58	88.4	(K)V L/E/A/S/F/S/V/G S D m\E\K(L)	758.0627	1498.709	16.4087
						2	11.92	4	76.1	(K)V A/Y V/L Q/G N G V\A/G I|V|L|P E S E\E K(V)	1086.7656	2172.155	0.3692
1803		**26S proteasome non-ATPase regulatory subunit 7 homolog A**	7(8)	25%	92.6	2	17.11	4.39	93.5	(K)E/H|V|V|G|W|Y/S T G P K(L)	680.4177	1359.669	0.1589
		(Vitis vinifera)				2	16.5	11.54	100	(K)A Y/Y/A|V|E/E|V|K/E|N\A T Q K(S)	872.061	1742.86	0.2552
						2	15.18	7.18	89.9	(K)A E D A\K/P A A|V|P A\V\A\G\S(-)	677.1885	1353.701	-0.3311
						2	12.36	4.66	97.3	(R)S V/I/A/L/H/N L\I|N\N K(M)	668.4873	1335.774	0.193
						3	11.73	3.43	99	(K)A/Y|Y|A|V E/E\V K E N\A T Q K(S)	582.1947	1742.86	1.71
						2	10.75	4.56	100	(R)V I|G/V|L|L|G\S S F K(G)	560.4478	1119.677	0.2111
						2	10.45	2.61	80.9	(K)D T T I/S/T/L|A|T/E V T G K(L)	718.6716	1436.748	-0.412
						2	10.25	2.71	100	(K)A Y|Y|A|V|E\E V\K(E)	536.9424	1071.536	1.3418
1818	2P3X	**Polyphenol Oxidase**	2(2)	5%	23.29	2	12.86	5.28	97	(-)A/P|I/Q|A|P D|I\S K(C)	520.4093	1039.578	0.2331
		(Vitis vinifera)				2	10.43	3.17	98.1	(K)L F|L|G|Y|P Y R(A)	515.0011	1028.556	0.4385
1821	AAB41022.1	**Polyphenol oxidase**	3(4)	7%	41.41	3	16.3	7.4	84	(K)W T|G/L/A/D K P S\E D|M/G/N|F|Y/T\A G R(D)	739.7224	2216.008	1.145
		(Vitis vinifera)				2	14.54	4.15	87.4	(R)Y Q/Y/Q/D/I|P\I|P W|L\P\K(N)	831.3149	1660.873	0.7492
						3	11.07	3.16	86.1	(K)W/T|G/L/A/D K P S E D/m G N F/Y\T A\G R(D)	745.1687	2216.008	17.4839
						2	10.57	2.43	94.8	(K)L F|L|G|Y|P Y R(A)	515.0437	1028.556	0.5237
1853	CAN68994.1	**Hypothetical protein VITISV_040294**	2(2)	7%	34.97	2	18.35	8.45	97.5	(K)Y/I|G|L|S|E/A|S/A|S T I R(R)	685.0657	1367.717	1.4076
		(Vitis vinifera)				2	16.62	4.76	92.8	(K)L V|E/N|L|S N|N/D|F R(K)	660.9023	1320.654	0.1431
	protein-protein BLAST:	**Aldo-keto reductase 4**											
1854	CAN68994.1	**Hypothetical protein VITISV_040294**	2(2)	7%	31.62	2	18.42	6.92	98.6	(K)Y/I|G|L|S/E/A|S/A|S T I R(R)	684.2859	1367.717	-0.152
	protein-protein BLAST:	*(Vitis vinifera)*				2	13.2	4.52	93.5	(R)V/P/I/E|V|T|I/G E|L K(K)	599.4895	1197.709	0.2628
		**Aldo-keto reductase 4**											
1855	CAN68994.1	**Hypothetical protein VITISV_040294**	2(2)	6%	24.57	2	14.32	3.4	87.9	(K)L/V/E/N|L|S N/N\D|F R(K)	660.9688	1320.654	0.2761
		(Vitis vinifera)				2	10.25	3.28	100	(R)V/P/I/E|V|T I/G E|L K(K)			
	protein-protein BLAST:	**Aldo-keto reductase 4**									599.5891	1197.709	0.462
1866	XP_002270155.1	**Glutelin type-A 3**	2(3)	9%	38.33	2	19.65	5.02	95.2	(R)G F|A|L|P/H|Y|A D|S N\K(I)	660.4261	1319.638	0.207
		(Vitis vinifera)				2	18.68	10.96	89.4	(K)I/F/E/G E/G G T/Y|Y|S/W|S/S/A E Y E L\L K(E)	1215.1157	2429.118	0.1058
						2	18.33	9.2	97.1	(K)I F/E/G E/G/G/T/Y/Y|S/W|S/S A/E/Y E L\L K(E)	1215.9082	2429.118	1.6908
1867	XP_002270155.1	**Glutelin type-A 3**	5(14)	21%	97.17	2	21.76	11.28	96.8	(K)K/F/P/F/L|G/E|V|G/L|S A T L\V\K(M)	853.5789	1705.989	0.1618
		(Vitis vinifera)				2	21.3	10.81	95.9	(K)K/F/P/F/L|G|E|V|G|L|S\A T L\V\K(M)	853.887	1705.989	0.778
						2	20.17	6.5	96.4	(R)G F|A|L|P/H|Y|A|D|S N\K(I)	660.822	1319.638	0.9988
						2	19.38	10.4	97.7	(K)I F/E G E/G/G/T/Y/Y|S/W|S/S/A/E|Y E L\L K(E)	1215.8804	2429.118	1.6352
						3	18.07	3.93	93.2	(K)L V/Y/N/I|D|A/A|L|P D|I|H|V Q\N A G/L|L\T A L T A K(K)	912.6973	2734.514	1.5636
						2	17.79	7.92	90.8	(K)F/P/F|L|G|E|V G/L|S\A/T L\V\K(M)	789.7192	1577.894	0.5374
						3	17.04	4.73	87.9	(K)K F/P F L\G E|V|G|L|S|A|T L\V\K(M)	569.259	1705.989	-0.2263
						2	16.93	8.62	98.4	(K)I F/E/G E/G/G/T/Y/Y|S/W|S/S/A/E Y E L\L K(E)	1215.2649	2429.118	0.4042
						2	14.06	7.19	92.6	(K)I F/E G/E/G/G T/Y/Y|S/W/S/S A/E Y E L\L K(E)	1215.5518	2429.118	0.978
						3	13.26	2.84	79.2	(K)K F P/F\L G/E\V|G|L|S|A|T L\V\K(M)	569.6602	1705.989	0.9773
						2	13.2	3.24	98.9	(R)I Q|V|V|G|I|N/S E R(A)	557.8367	1114.622	0.0446
						2	12.75	5.44	77.4	(K)I F E G E/G G/T/Y|Y/S/W|S/S/A E/Y E L L\K(E)	1215.0791	2429.118	0.0326
						2	10.69	4.52	93.5	(R)I/Q|V|V|G/I|N S E R(A)	558.1606	1114.622	0.6924
						2	10.5	3.32	100	(K)L V/Y N I D/A/A/L|P D/I|H\V Q\N/A G L\L T A L T A K(K)	1368.0205	2734.514	0.5199
1887	ABC86739.1	**Cyclase**	4(4)	16%	61.06	2	18.09	8.9	94.3	(K)E F/E/S/D/Y|A G|F|T/E/D/G\A R(W)	847.2908	1693.698	-0.1233
		(Vitis pseudoreticulata)				2	16.63	3.61	95.5	(R)L/P|G|A|E/G|A|P I\R(C)	490.8502	980.552	0.1408
						2	13.92	2	87.9	(R)W/L|V|E|N/T D|I\K(L)	559.4907	1117.589	0.3853
						2	12.42	3.75	98	(R)E V|I|L|V|E/S L K(L)	515.4656	1029.619	0.3049
1916	CAI56335.1	**Isoflavone reductase-like protein 6**	11(36)	43%	176.1	2	19.5	11.43	96.2	(K)Y/T|T V|D|E|Y|L|D\Q|F\V(-)	746.8967	1492.684	0.1019
		(Vitis vinifera)				2	19.24	10.77	93.9	(K)S/S/G V/S/L/V/Y/G/D/L/Y/D/H\E\S L V\K(A)	1034.7603	2068.023	0.49
						2	19.15	7.99	98.7	(R)D/K|V|I|I|L|G|D|G\N|P K(A)	635.0327	1268.721	0.3372
						2	18.77	9.3	94.3	(K)S/S G V/S L/V|Y/G/D/L/Y/D|H\E\S L\V\K(A)	1034.8079	2068.023	0.5852
						2	18.72	9.14	92.6	(K)S/S/G V/S/L|V|Y|G/D/L/Y/D/H\E\S L V K(A)	1034.6038	2068.023	0.177
						3	18.66	6.2	97.1	(K)S/S/G/V|S/L|V|Y|G D/L/Y D/H E S L V K(A)	690.3005	2068.023	0.8636
						2	18.25	10.76	94.4	(K)S/S/G V/S/L/V/Y/G/D/L/Y/D/H\E S L V K(A)	1035.0715	2068.023	1.1124

<table>
<tr><td></td><td></td><td></td><td></td><td></td><td></td><td>2</td><td>18.19</td><td>10.18</td><td>92.8</td><td>(K)S/S/G V/S/L|V|Y/G/D/L/Y/D/H E\S L V\K(A)</td><td>1035.238</td><td>2068.023</td><td>1.4454</td></tr>
<tr><td></td><td></td><td></td><td></td><td></td><td></td><td>2</td><td>17.82</td><td>8.45</td><td>89.8</td><td>(K)S/S/G/V/S L/V|Y/G/D/L/Y/D H\E/S\L V K(A)</td><td>1034.3726</td><td>2068.023</td><td>-0.2854</td></tr>
<tr><td></td><td></td><td></td><td></td><td></td><td></td><td>2</td><td>17.48</td><td>7.72</td><td>92.2</td><td>(K)G D/H T N F E|I|E|P\S/F|G V/E/A S E\L Y/P D\V K(Y)</td><td>1340.4746</td><td>2680.241</td><td>-0.2994</td></tr>
<tr><td></td><td></td><td></td><td></td><td></td><td></td><td>2</td><td>17.12</td><td>5.93</td><td>93.4</td><td>(R)F/F|P/S/E|F|G|N D V D\R(V)</td><td>715.2839</td><td>1429.638</td><td>-0.0777</td></tr>
<tr><td></td><td></td><td></td><td></td><td></td><td></td><td>2</td><td>17.09</td><td>7.98</td><td>91.3</td><td>(K)A V/F/N/K/E|D|D|I|G T/Y/T I\K(A)</td><td>857.6561</td><td>1713.869</td><td>0.4355</td></tr>
<tr><td></td><td></td><td></td><td></td><td></td><td></td><td>3</td><td>17.04</td><td>4.9</td><td>94.9</td><td>(K)S/S/G/V|S/L|V|Y|G D/L/Y D H E S L V K(A)</td><td>690.2484</td><td>2068.023</td><td>0.7073</td></tr>
<tr><td></td><td></td><td></td><td></td><td></td><td></td><td>2</td><td>16.33</td><td>7.51</td><td>86.8</td><td>(K)S/S/G V/S/L/V|Y/G/D/L/Y/D/H E S L V K(A)</td><td>1034.7837</td><td>2068.023</td><td>0.5368</td></tr>
<tr><td></td><td></td><td></td><td></td><td></td><td></td><td>2</td><td>15.44</td><td>6.19</td><td>97</td><td>(R)F/F|P/S/E|F|G|N D|V D R(V)</td><td>715.3948</td><td>1429.638</td><td>0.1441</td></tr>
<tr><td></td><td></td><td></td><td></td><td></td><td></td><td>2</td><td>15.38</td><td>6.71</td><td>96.9</td><td>(K)I L|I|I|G|G/T|G Y\I\G K(F)</td><td>602.7512</td><td>1204.73</td><td>-0.2349</td></tr>
<tr><td></td><td></td><td></td><td></td><td></td><td></td><td>2</td><td>15.18</td><td>3.9</td><td>96.3</td><td>(R)F/F|P/S/E/F|G/N D/V D R(V)</td><td>715.3442</td><td>1429.638</td><td>0.0429</td></tr>
<tr><td></td><td></td><td></td><td></td><td></td><td></td><td>2</td><td>14.85</td><td>4.47</td><td>92.7</td><td>(R)F/F|P/S/E|F|G/N D/V D R(V)</td><td>716.0349</td><td>1429.638</td><td>1.4243</td></tr>
<tr><td></td><td></td><td></td><td></td><td></td><td></td><td>2</td><td>14.68</td><td>4.69</td><td>96.4</td><td>(R)F/F|P/S/E|F|G|N D V D R(V)</td><td>715.4734</td><td>1429.638</td><td>0.3013</td></tr>
<tr><td></td><td></td><td></td><td></td><td></td><td></td><td>2</td><td>14.29</td><td>5.86</td><td>95.1</td><td>(K)I L|I|I|G|G/T G/Y/I\G K(F)</td><td>603.4878</td><td>1204.73</td><td>1.2383</td></tr>
<tr><td></td><td></td><td></td><td></td><td></td><td></td><td>2</td><td>14.17</td><td>4.7</td><td>96</td><td>(R)L/G/H|P|T|F\A\L/I\R(E)</td><td>563.0081</td><td>1124.658</td><td>0.3514</td></tr>
<tr><td></td><td></td><td></td><td></td><td></td><td></td><td>3</td><td>13.61</td><td>2.04</td><td>89.8</td><td>(K)A V/F/N K E\D\D|I|G/T\Y T I K(A)</td><td>572.2939</td><td>1713.869</td><td>0.9978</td></tr>
<tr><td></td><td></td><td></td><td></td><td></td><td></td><td>3</td><td>13.56</td><td>3.69</td><td>87.9</td><td>(K)A V|F/N/K E\D D|I|G/T/Y T I\K(A)</td><td>572.3875</td><td>1713.869</td><td>1.2786</td></tr>
<tr><td></td><td></td><td></td><td></td><td></td><td></td><td>2</td><td>13.41</td><td>5.34</td><td>97.7</td><td>(K)V I|I|L|G/D|G N\P K(A)</td><td>513.4473</td><td>1025.599</td><td>0.2884</td></tr>
<tr><td></td><td></td><td></td><td></td><td></td><td></td><td>2</td><td>13.38</td><td>2.7</td><td>96.7</td><td>(K)I L|I|I|G|G T/G/Y I\G K(F)</td><td>603.3521</td><td>1204.73</td><td>0.9669</td></tr>
<tr><td></td><td></td><td></td><td></td><td></td><td></td><td>2</td><td>13.35</td><td>3.73</td><td>96.2</td><td>(K)I L|I|I|G|G T G/Y\I\G K(F)</td><td>603.0691</td><td>1204.73</td><td>0.4009</td></tr>
<tr><td></td><td></td><td></td><td></td><td></td><td></td><td>2</td><td>12.91</td><td>3.71</td><td>91.7</td><td>(K)V I|I|L|G/D/G\N|P K(A)</td><td>513.2983</td><td>1025.599</td><td>-0.0096</td></tr>
<tr><td></td><td></td><td></td><td></td><td></td><td></td><td>2</td><td>12.65</td><td>3.69</td><td>94.6</td><td>(R)F/F|P/S/E|F|G N D V\D R(V)</td><td>715.5154</td><td>1429.638</td><td>0.3853</td></tr>
<tr><td></td><td></td><td></td><td></td><td></td><td></td><td>2</td><td>12.34</td><td>2.52</td><td>95.8</td><td>(K)I L|I|I|G|G T G Y\I\G K(F)</td><td>603.1533</td><td>1204.73</td><td>0.5693</td></tr>
<tr><td></td><td></td><td></td><td></td><td></td><td></td><td>3</td><td>12.18</td><td>4.08</td><td>91.5</td><td>(K)A V|F/N\K E D D|I|G|T/Y T I\K(A)</td><td>572.0425</td><td>1713.869</td><td>0.2436</td></tr>
<tr><td></td><td></td><td></td><td></td><td></td><td></td><td>2</td><td>11.87</td><td>4.52</td><td>96.8</td><td>(K)V I|I|L|G/D|G N\P K(A)</td><td>513.3103</td><td>1025.599</td><td>0.0144</td></tr>
<tr><td></td><td></td><td></td><td></td><td></td><td></td><td>2</td><td>11.81</td><td>3.89</td><td>91.8</td><td>(K)S/G|I|I|E|S\F K(S)</td><td>440.5084</td><td>880.477</td><td>-0.4679</td></tr>
<tr><td></td><td></td><td></td><td></td><td></td><td></td><td>2</td><td>11.75</td><td>3.46</td><td>100</td><td>(K)F I|V|A|A|S|A R(L)</td><td>417.9625</td><td>834.483</td><td>0.4345</td></tr>
<tr><td></td><td></td><td></td><td></td><td></td><td></td><td>2</td><td>11.73</td><td>5.39</td><td>82.7</td><td>(K)S/S G V S/L/V/Y/G/D/L/Y/D/H/E S\L V K(A)</td><td>1034.7461</td><td>2068.023</td><td>0.4616</td></tr>
<tr><td></td><td></td><td></td><td></td><td></td><td></td><td>2</td><td>11.31</td><td>3.31</td><td>95.3</td><td>(K)I L|I|I|G|G/T G Y I\G K(F)</td><td>603.3103</td><td>1204.73</td><td>0.8833</td></tr>
<tr><td></td><td></td><td></td><td></td><td></td><td></td><td>2</td><td>10.87</td><td>2.4</td><td>95.7</td><td>(K)F I|V|A|A|S/A R(L)</td><td>417.7874</td><td>834.483</td><td>0.0843</td></tr>
<tr><td>1917</td><td>XP_002284566.1</td><td>26S proteasome non-ATPase regulatory subunit 14 homolog</td><td>3(9)</td><td>11%</td><td>46.22</td><td>2</td><td>20.32</td><td>9.39</td><td>95.9</td><td>(R)L I/N|P Q/T|M/m|L|G\Q|E|P R(Q)</td><td>822.7397</td><td>1627.83</td><td>16.6427</td></tr>
<tr><td></td><td></td><td>(Vitis vinifera)</td><td></td><td></td><td></td><td>2</td><td>19.14</td><td>8.1</td><td>95.2</td><td>(R)L I/N|P Q/T/M/M|L|G Q|E|P R(Q)</td><td>814.8174</td><td>1627.83</td><td>0.7981</td></tr>
<tr><td></td><td></td><td></td><td></td><td></td><td></td><td>3</td><td>18.28</td><td>7.06</td><td>89.7</td><td>(R)L I/N|P Q\T|m|m|L/G|Q E|P R(Q)</td><td>554.4036</td><td>1627.83</td><td>33.3668</td></tr>
<tr><td></td><td></td><td></td><td></td><td></td><td></td><td>3</td><td>18.27</td><td>9.22</td><td>97.2</td><td>(R)L I/N/P Q|T\M|m|L|G|Q E\P\R(Q)</td><td>548.6757</td><td>1627.83</td><td>16.1831</td></tr>
<tr><td></td><td></td><td></td><td></td><td></td><td></td><td>2</td><td>17.58</td><td>5.65</td><td>87.2</td><td>(R)L I/N/P/Q/T/m/m/L|G Q|E|P R(Q)</td><td>830.4653</td><td>1627.83</td><td>32.0939</td></tr>
<tr><td></td><td></td><td></td><td></td><td></td><td></td><td>3</td><td>15.97</td><td>4.42</td><td>86.4</td><td>(R)L I|N/P Q\T m|M|L|G/Q/E\P R(Q)</td><td>548.73</td><td>1627.83</td><td>16.346</td></tr>
<tr><td></td><td></td><td></td><td></td><td></td><td></td><td>2</td><td>15.65</td><td>4.63</td><td>96.8</td><td>(K)A V/Q/E/E|D E|L|P|P E\K(L)</td><td>692.6177</td><td>1383.664</td><td>0.5643</td></tr>
<tr><td></td><td></td><td></td><td></td><td></td><td></td><td>3</td><td>14.41</td><td>3.44</td><td>97.8</td><td>(R)L I/N/P Q T\M\M|L|G\Q/E P R(Q)</td><td>543.5845</td><td>1627.83</td><td>0.9095</td></tr>
<tr><td></td><td></td><td></td><td></td><td></td><td></td><td>2</td><td>10.25</td><td>2.52</td><td>93.7</td><td>(K)L/A|I|A|N/V/G R(Q)</td><td>407.6617</td><td>813.494</td><td>0.822</td></tr>
<tr><td>1924</td><td>CAI56334.1</td><td>Isoflavone reductase-like protein 5</td><td>7(16)</td><td>35%</td><td>119.72</td><td>2</td><td>20.32</td><td>15.17</td><td>97.7</td><td>(K)Q V D/V/V/I/S/T/V/G/H|A|Q|L|P D\Q V K(I)</td><td>1017.9014</td><td>2033.103</td><td>1.6929</td></tr>
<tr><td></td><td></td><td>(Vitis vinifera)</td><td></td><td></td><td></td><td>2</td><td>19.28</td><td>10.52</td><td>94.4</td><td>(K)S S G V/T L|V|Y|G/D|L|Y|D/H E S L\V K(A)</td><td>1042.2012</td><td>2082.039</td><td>1.3561</td></tr>
<tr><td></td><td></td><td></td><td></td><td></td><td></td><td>2</td><td>18.4</td><td>8.03</td><td>97.6</td><td>(K)I L|I|I|G|G|T/G/Y\I|G K(F)</td><td>603.0681</td><td>1204.73</td><td>0.3989</td></tr>
<tr><td></td><td></td><td></td><td></td><td></td><td></td><td>2</td><td>18.26</td><td>12.63</td><td>91</td><td>(K)Q V D/V/V/I/S/T/V/G/H/A|Q|L|P D Q V K(I)</td><td>1017.6027</td><td>2033.103</td><td>1.0955</td></tr>
<tr><td></td><td></td><td></td><td></td><td></td><td></td><td>2</td><td>17.89</td><td>7.07</td><td>96.8</td><td>(K)I L|I|I|G|G|T/G/Y\I\G K(F)</td><td>603.0717</td><td>1204.73</td><td>0.4061</td></tr>
<tr><td></td><td></td><td></td><td></td><td></td><td></td><td>2</td><td>17.76</td><td>6.04</td><td>94.5</td><td>(K)S S G V/T/L/V|Y|G/D|L/Y/D/H E\S L\V K(A)</td><td>1041.7095</td><td>2082.039</td><td>0.3727</td></tr>
<tr><td></td><td></td><td></td><td></td><td></td><td></td><td>3</td><td>17.72</td><td>9.38</td><td>94.7</td><td>(K)Q/V/D/V/V/I/S/T/V/G/H/A/Q/L/P D Q V K(I)</td><td>678.103</td><td>2033.103</td><td>-0.8081</td></tr>
<tr><td></td><td></td><td></td><td></td><td></td><td></td><td>3</td><td>17.55</td><td>7.69</td><td>90.4</td><td>(K)G D H T N F/E I\E|P/S/F G V|E A T/E|L|Y|P D V\K(Y)</td><td>899.1707</td><td>2694.257</td><td>1.2406</td></tr>
<tr><td></td><td></td><td></td><td></td><td></td><td></td><td>2</td><td>16.94</td><td>8.58</td><td>93.4</td><td>(K)S S G V/T L/V|Y|G/D/L/Y|D/H E\S L V K(A)</td><td>1041.9395</td><td>2082.039</td><td>0.8327</td></tr>
<tr><td></td><td></td><td></td><td></td><td></td><td></td><td>3</td><td>16.84</td><td>5.5</td><td>94.7</td><td>(K)S S/G/V|T/L|V|Y/G/D|L/Y D/H E S L V K(A)</td><td>694.8777</td><td>2082.039</td><td>0.5796</td></tr>
<tr><td></td><td></td><td></td><td></td><td></td><td></td><td>2</td><td>16.67</td><td>6.07</td><td>96.1</td><td>(R)F/F|P/S/E|F|G/N/D/V\D R(V)</td><td>716.0524</td><td>1429.638</td><td>1.4593</td></tr>
<tr><td></td><td></td><td></td><td></td><td></td><td></td><td>2</td><td>16.59</td><td>5.76</td><td>93.7</td><td>(R)F/F|P/S/E|F|G/N/D/V D\R(V)</td><td>715.2529</td><td>1429.638</td><td>-0.1397</td></tr>
<tr><td></td><td></td><td></td><td></td><td></td><td></td><td>2</td><td>15.54</td><td>9.28</td><td>88.9</td><td>(K)Y/T/T V/D|E|Y|L|N\Q\F\V(-)</td><td>746.9836</td><td>1491.7</td><td>1.2597</td></tr>
<tr><td></td><td></td><td></td><td></td><td></td><td></td><td>3</td><td>14.15</td><td>3.03</td><td>94.5</td><td>(K)Q/V/D/V/V/I/S/T V/G H A Q/L/P D Q V K(I)</td><td>678.4781</td><td>2033.103</td><td>0.3172</td></tr>
<tr><td></td><td></td><td></td><td></td><td></td><td></td><td>3</td><td>13.21</td><td>2.26</td><td>88.7</td><td>(K)Q V/D/V/V/I/S/T V/G H/A Q/L P D Q V K(I)</td><td>678.6348</td><td>2033.103</td><td>0.7873</td></tr>
<tr><td></td><td></td><td></td><td></td><td></td><td></td><td>2</td><td>11.96</td><td>4.71</td><td>98.9</td><td>(K)V I|I|L|G/D\G/N\P K(A)</td><td>513.6199</td><td>1025.599</td><td>0.6336</td></tr>
<tr><td>1935</td><td>CAI56334.1</td><td>Isoflavone reductase-like protein 5</td><td>5(5)</td><td>24%</td><td>68.36</td><td>2</td><td>19.28</td><td>11.38</td><td>96.6</td><td>(K)Q V D/V V/I|S/T/V/G/H|A/Q|L|P D Q V K(I)</td><td>1017.5693</td><td>2033.103</td><td>1.0287</td></tr>
<tr><td></td><td></td><td>(Vitis vinifera)</td><td></td><td></td><td></td><td>2</td><td>13.5</td><td>4.81</td><td>100</td><td>(R)D/K/V|I|I|L|G D|G\N\P K(A)</td><td>635.0706</td><td>1268.721</td><td>0.413</td></tr>
<tr><td></td><td></td><td></td><td></td><td></td><td></td><td>2</td><td>12.83</td><td>6.6</td><td>95.8</td><td>(K)S S G V/T L/V/Y/G/D L/Y/D/H\E\S L V K(A)</td><td>1041.8113</td><td>2082.039</td><td>0.5763</td></tr>
<tr><td></td><td></td><td></td><td></td><td></td><td></td><td>2</td><td>11.5</td><td>3.48</td><td>100</td><td>(K)I L|I|I|G|G T G/Y I\G K(F)</td><td>603.0875</td><td>1204.73</td><td>0.4377</td></tr>
<tr><td></td><td></td><td></td><td></td><td></td><td></td><td>2</td><td>11.25</td><td>3.66</td><td>100</td><td>(R)F F|P/S/E|F|G N D V D R(V)</td><td>716.1396</td><td>1429.638</td><td>1.6337</td></tr>
<tr><td>1951</td><td>XP_002277446.1</td><td>Glucan endo-1,3-beta-glucosidase</td><td>5(10)</td><td>25%</td><td>75.22</td><td>3</td><td>17.8</td><td>5.4</td><td>92.3</td><td>(K)V S T A/I|D/T G V\L\G\V|S|Y|P P S S G S F K(S)</td><td>724.0669</td><td>2169.107</td><td>1.0788</td></tr>
<tr><td></td><td></td><td>(Vitis vinifera)</td><td></td><td></td><td></td><td>2</td><td>17.72</td><td>8.77</td><td>93</td><td>(K)V S T A I D/T G V|L|G|V|S|Y|P/P S S G S\F K(S)</td><td>1085.292</td><td>2169.107</td><td>0.4693</td></tr>
<tr><td></td><td></td><td></td><td></td><td></td><td></td><td>2</td><td>16.82</td><td>11.15</td><td>95.2</td><td>(K)T/Y|N/S/N/L|I/Q/H\V\K(G)</td><td>659.1943</td><td>1316.696</td><td>0.6856</td></tr>
<tr><td></td><td></td><td></td><td></td><td></td><td></td><td>2</td><td>15.04</td><td>5.3</td><td>82.5</td><td>(K)I V V/S E\S/G/W|P/S A G|G T\Q|T T\V\D N A R(T)</td><td>1116.7178</td><td>2232.089</td><td>0.3392</td></tr>
<tr><td></td><td></td><td></td><td></td><td></td><td></td><td>3</td><td>14.29</td><td>4.77</td><td>89.2</td><td>(K)I V/V S E\S G W|P S A G G\T Q/T/T/V/D N A\R(T)</td><td>744.9126</td><td>2232.089</td><td>0.6341</td></tr>
<tr><td></td><td></td><td></td><td></td><td></td><td></td><td>3</td><td>13.95</td><td>3.09</td><td>87.4</td><td>(K)V S T A I\D T G V|L\G\V|S|Y|P P S S G S F K(S)</td><td>724.2338</td><td>2169.107</td><td>1.5795</td></tr>
<tr><td></td><td></td><td></td><td></td><td></td><td></td><td>3</td><td>13.18</td><td>6.38</td><td>91.1</td><td>(R)N G N/N/L|P A\P G E V V|A\L|Y/N/Q Y\N\I\R(R)</td><td>772.1506</td><td>2316.173</td><td>-1.7359</td></tr>
</table>

<table>
<tr><td></td><td></td><td></td><td></td><td></td><td></td><td>2</td><td>12.38</td><td>5.73</td><td>96.1</td><td>(K)H/W|G/L|F|L|P N K(Q)</td><td>555.6532</td><td>1111.605</td><td>-1.3056</td></tr>
<tr><td></td><td></td><td></td><td></td><td></td><td></td><td>3</td><td>12.14</td><td>4.25</td><td>94.7</td><td>(K)T/Y|N/S N|L|I/Q/H V K(G)</td><td>440.0171</td><td>1316.696</td><td>1.341</td></tr>
<tr><td></td><td></td><td></td><td></td><td></td><td></td><td>2</td><td>11.32</td><td>4.85</td><td>95.2</td><td>(K)V S T A I D T/G V/L|G V|S|Y|P P S S G S F K(S)</td><td>1085.5562</td><td>2169.107</td><td>0.9977</td></tr>
<tr><td>1961</td><td>XP_002277446.1</td><td>Glucan endo-1,3-beta-glucosidase</td><td>8(46)</td><td>45%</td><td>148.68</td><td>3</td><td>21.5</td><td>11.95</td><td>95.7</td><td>(K)R/P T G P I E T Y\V\F|A|M\F|D|E/D/N K/T|P E\L\E\K(H)</td><td>976.7633</td><td>2927.413</td><td>0.8622</td></tr>
<tr><td></td><td></td><td>(Vitis vinifera)</td><td></td><td></td><td></td><td>3</td><td>20.82</td><td>6.45</td><td>91.4</td><td>(R)N G N/N/L|P A/P/G E V\V\A|L|Y|N/Q|Y|N\I\R(R)</td><td>773.3164</td><td>2316.173</td><td>1.7615</td></tr>
<tr><td></td><td></td><td></td><td></td><td></td><td></td><td>2</td><td>20.42</td><td>13.39</td><td>98.3</td><td>(K)V S T A I/D|T G V|L|G|V|S|Y|P P S\S G S\F K(S)</td><td>1084.9221</td><td>2169.107</td><td>-0.2705</td></tr>
<tr><td></td><td></td><td></td><td></td><td></td><td></td><td>3</td><td>20.18</td><td>9.39</td><td>95.2</td><td>(R)Y I A V G N E/V/S/P/S\G A Q A|Q/F/V|L|P A/M Q/N/I N N A I|S/S A G L|G N Q I\K(V)</td><td>1325.1904</td><td>3972.028</td><td>1.5283</td></tr>
<tr><td></td><td></td><td></td><td></td><td></td><td></td><td>2</td><td>19.91</td><td>9.02</td><td>98</td><td>(K)V S T A I D T/G V|L|G\V|S|Y|P P S S G S F K(S)</td><td>1085.7266</td><td>2169.107</td><td>1.3385</td></tr>
<tr><td></td><td></td><td></td><td></td><td></td><td></td><td>2</td><td>19.43</td><td>9.53</td><td>98.3</td><td>(K)V S T A I D/T/G V|L|G|V|S|Y|P/P S S G S\F K(S)</td><td>1085.9111</td><td>2169.107</td><td>1.7075</td></tr>
<tr><td></td><td></td><td></td><td></td><td></td><td></td><td>3</td><td>19.37</td><td>9.48</td><td>88.8</td><td>(K)R P T G P I E/T Y\V\F\A|m|F/D E D N K|T|P E L E\K(H)</td><td>982.4742</td><td>2927.413</td><td>17.9949</td></tr>
<tr><td></td><td></td><td></td><td></td><td></td><td></td><td>2</td><td>19.06</td><td>12.28</td><td>89.4</td><td>(K)V S T A I/D T/G|V|L|G|V|S|Y|P/P S S G S\F K(S)</td><td>1085.1404</td><td>2169.107</td><td>0.1661</td></tr>
<tr><td></td><td></td><td></td><td></td><td></td><td></td><td>2</td><td>18.9</td><td>11.82</td><td>97.6</td><td>(K)V S T A I/D T/G V|L|G|V|S|Y|P/P S S G S\F K(S)</td><td>1085.0596</td><td>2169.107</td><td>0.0045</td></tr>
<tr><td></td><td></td><td></td><td></td><td></td><td></td><td>3</td><td>18.77</td><td>8.36</td><td>91.2</td><td>(R)N G N N/L|P A\P G E V\V\A\L|Y/N/Q|Y|N I\R(R)</td><td>773.0959</td><td>2316.173</td><td>1.1</td></tr>
<tr><td></td><td></td><td></td><td></td><td></td><td></td><td>2</td><td>18.57</td><td>6.45</td><td>96.3</td><td>(K)V S T A I/D T G V|L|G|V|S|Y|P/P S S G\S\F K(S)</td><td>1085.8494</td><td>2169.107</td><td>1.5841</td></tr>
<tr><td></td><td></td><td></td><td></td><td></td><td></td><td>2</td><td>18.34</td><td>10.27</td><td>95.7</td><td>(K)V S T A I/D/T/G V|L|G|V|S|Y|P P S S G S\F K(S)</td><td>1085.0298</td><td>2169.107</td><td>-0.0551</td></tr>
<tr><td></td><td></td><td></td><td></td><td></td><td></td><td>3</td><td>18.12</td><td>9.25</td><td>89.6</td><td>(R)N G N N/L|P A\P/G\E V\V\A\L|Y/N/Q|Y\N I\R(R)</td><td>773.2659</td><td>2316.173</td><td>1.61</td></tr>
<tr><td></td><td></td><td></td><td></td><td></td><td></td><td>2</td><td>17.81</td><td>11.38</td><td>95.9</td><td>(K)V S T A I/D T/G V|L|G/V|S|Y|P P S S G S\F K(S)</td><td>1084.8091</td><td>2169.107</td><td>-0.4965</td></tr>
<tr><td></td><td></td><td></td><td></td><td></td><td></td><td>2</td><td>17.78</td><td>10.71</td><td>95.1</td><td>(K)V S/T A I/D T G V|L|G|V|S|Y|P/P S S G S F K(S)</td><td>1085.2212</td><td>2169.107</td><td>0.3277</td></tr>
<tr><td></td><td></td><td></td><td></td><td></td><td></td><td>2</td><td>17.67</td><td>10.35</td><td>93.1</td><td>(K)V S T A I/D/T G V/L|G\V|S|Y|P P S S G S\F K(S)</td><td>1085.0967</td><td>2169.107</td><td>0.0787</td></tr>
<tr><td></td><td></td><td></td><td></td><td></td><td></td><td>2</td><td>17.54</td><td>7.25</td><td>90.9</td><td>(R)N G N N L|P A|P G E|V|V A/L|Y|N\Q\Y N I R(R)</td><td>1158.5562</td><td>2316.173</td><td>-0.068</td></tr>
<tr><td></td><td></td><td></td><td></td><td></td><td></td><td>3</td><td>17.37</td><td>4.71</td><td>85.8</td><td>(R)N G N N/L|P A\P/G E V\V\A|L|Y|N/Q|Y\N I\R(R)</td><td>773.3058</td><td>2316.173</td><td>1.7297</td></tr>
<tr><td></td><td></td><td></td><td></td><td></td><td></td><td>2</td><td>17.18</td><td>7.3</td><td>85.7</td><td>(K)I V/V/S E|S/G/W|P/S/A G/G T Q|T T\V|D N A R(T)</td><td>1116.2725</td><td>2232.089</td><td>-0.5514</td></tr>
<tr><td></td><td></td><td></td><td></td><td></td><td></td><td>3</td><td>16.97</td><td>7.23</td><td>89.3</td><td>(K)T/Y|N/S N|L|I/Q H/V K(G)</td><td>440.0438</td><td>1316.696</td><td>1.4211</td></tr>
<tr><td></td><td></td><td></td><td></td><td></td><td></td><td>2</td><td>16.84</td><td>8.39</td><td>93</td><td>(R)N G N N/L/P A|P G E/V/V A|L|Y|N/Q Y N I R(R)</td><td>1158.6924</td><td>2316.173</td><td>0.2044</td></tr>
<tr><td></td><td></td><td></td><td></td><td></td><td></td><td>2</td><td>16.21</td><td>7.21</td><td>92.1</td><td>(K)H/W|G/L|F|L|P|N\K(Q)</td><td>556.3125</td><td>1111.605</td><td>0.013</td></tr>
<tr><td></td><td></td><td></td><td></td><td></td><td></td><td>2</td><td>16.14</td><td>6.49</td><td>84.2</td><td>(K)I V/V/S E/S/G/W|P/S/A G/G T Q|T T\V|D N A R(T)</td><td>1116.9258</td><td>2232.089</td><td>0.7552</td></tr>
<tr><td></td><td></td><td></td><td></td><td></td><td></td><td>2</td><td>16.14</td><td>6.5</td><td>91.3</td><td>(K)H/W|G|L|F|L|P\N\K(Q)</td><td>556.4816</td><td>1111.605</td><td>0.3512</td></tr>
<tr><td></td><td></td><td></td><td></td><td></td><td></td><td>2</td><td>15.77</td><td>5.93</td><td>87.2</td><td>(R)N G N N/L|P A/P G E/V/V A|L|Y|N/Q\Y N I R(R)</td><td>1159.4014</td><td>2316.173</td><td>1.6224</td></tr>
<tr><td></td><td></td><td></td><td></td><td></td><td></td><td>3</td><td>15.4</td><td>3.23</td><td>93.1</td><td>(R)H/W|G L F|L|P/N/K/Q\P\K(-)</td><td>489.1721</td><td>1464.811</td><td>0.6907</td></tr>
<tr><td></td><td></td><td></td><td></td><td></td><td></td><td>2</td><td>15.06</td><td>6.68</td><td>73.3</td><td>(K)I V/V/S E\S/G/W|P/S/A G G T Q/T/T V|D N A R(T)</td><td>1116.6025</td><td>2232.089</td><td>0.1086</td></tr>
<tr><td></td><td></td><td></td><td></td><td></td><td></td><td>2</td><td>14.91</td><td>6.42</td><td>88.2</td><td>(K)T/Y|N/S/N/L|I/Q/H\V\K(G)</td><td>658.8475</td><td>1316.696</td><td>-0.008</td></tr>
<tr><td></td><td></td><td></td><td></td><td></td><td></td><td>3</td><td>14.78</td><td>5.59</td><td>92.6</td><td>(K)V S/T A I D T|G V/L G\V|S/Y|P P S/S G S F\K(S)</td><td>724.3126</td><td>2169.107</td><td>1.8159</td></tr>
<tr><td></td><td></td><td></td><td></td><td></td><td></td><td>2</td><td>14.63</td><td>3.07</td><td>81.3</td><td>(K)I V/V S E/S/G W|P S/A G/G T Q/T\T\V\D N A R(T)</td><td>1117.2358</td><td>2232.089</td><td>1.3752</td></tr>
<tr><td></td><td></td><td></td><td></td><td></td><td></td><td>2</td><td>14.61</td><td>7.26</td><td>83.2</td><td>(K)T/Y|N/S/N/L|I/Q/H\V\K(G)</td><td>658.894</td><td>1316.696</td><td>0.085</td></tr>
<tr><td></td><td></td><td></td><td></td><td></td><td></td><td>3</td><td>14.31</td><td>5.39</td><td>93.8</td><td>(K)R P T G P I E T Y|V F A\M|F/D E\D\N/K/T|P E\L E\K(H)</td><td>976.2858</td><td>2927.413</td><td>-0.5703</td></tr>
<tr><td></td><td></td><td></td><td></td><td></td><td></td><td>3</td><td>14.27</td><td>4.79</td><td>88.2</td><td>(K)I V/V/S E/S G W|P S A/G G T Q/T T|V/D N A R(T)</td><td>744.6721</td><td>2232.089</td><td>-0.0874</td></tr>
<tr><td></td><td></td><td></td><td></td><td></td><td></td><td>2</td><td>14.11</td><td>6.09</td><td>89.8</td><td>(K)H/W|G/L|F|L|P|N\K(Q)</td><td>556.6484</td><td>1111.605</td><td>0.6848</td></tr>
<tr><td></td><td></td><td></td><td></td><td></td><td></td><td>2</td><td>14</td><td>8.96</td><td>100</td><td>(K)V S T A I D T/G V/L|G/V|S|Y|P P S S G S F K(S)</td><td>1085.3396</td><td>2169.107</td><td>0.5645</td></tr>
<tr><td></td><td></td><td></td><td></td><td></td><td></td><td>3</td><td>13.96</td><td>4.53</td><td>89.7</td><td>(K)I V/V S E/S G W|P S A G G T Q/T|T|V/D N A R(T)</td><td>745.0687</td><td>2232.089</td><td>1.1024</td></tr>
<tr><td></td><td></td><td></td><td></td><td></td><td></td><td>3</td><td>13.74</td><td>3.23</td><td>72.5</td><td>(R)N/G N N/L/P A P G E V\V\A|L|Y/N Q|Y|N I\R(R)</td><td>772.6099</td><td>2316.173</td><td>-0.358</td></tr>
<tr><td></td><td></td><td></td><td></td><td></td><td></td><td>3</td><td>13.36</td><td>5.05</td><td>73.8</td><td>(R)N G N N L|P\A\P G E V\V\A\L/Y|N Q/Y N I\R(R)</td><td>773.2506</td><td>2316.173</td><td>1.5641</td></tr>
<tr><td></td><td></td><td></td><td></td><td></td><td></td><td>3</td><td>13.34</td><td>4.69</td><td>86.5</td><td>(K)I V/V S E S G W|P S A\G G T Q/T/T|V|D N A R(T)</td><td>744.8895</td><td>2232.089</td><td>0.5648</td></tr>
<tr><td></td><td></td><td></td><td></td><td></td><td></td><td>2</td><td>13.07</td><td>5.61</td><td>86</td><td>(K)H/W|G/L|F|L|P N\K(Q)</td><td>556.1565</td><td>1111.605</td><td>-0.299</td></tr>
<tr><td></td><td></td><td></td><td></td><td></td><td></td><td>2</td><td>13.03</td><td>3.75</td><td>80.1</td><td>(K)I V V/S E/S/G/W|P/S/A G G T/Q/T T V/D N A R(T)</td><td>1116.7859</td><td>2232.089</td><td>0.4754</td></tr>
<tr><td></td><td></td><td></td><td></td><td></td><td></td><td>3</td><td>12.74</td><td>4.53</td><td>89</td><td>(K)I V/V/S E S/G/W|P S A G G T Q/T|T V/D N A R(T)</td><td>744.592</td><td>2232.089</td><td>-0.3277</td></tr>
<tr><td></td><td></td><td></td><td></td><td></td><td></td><td>3</td><td>12.72</td><td>5.49</td><td>99</td><td>(K)T/Y|N/S N|L|I/Q H V K(G)</td><td>439.954</td><td>1316.696</td><td>1.1517</td></tr>
<tr><td></td><td></td><td></td><td></td><td></td><td></td><td>3</td><td>12.49</td><td>2.54</td><td>75.9</td><td>(K)I V/V/S E\S G W|P S\A G G T Q/T|T/V D/N A R(T)</td><td>745.0082</td><td>2232.089</td><td>0.9209</td></tr>
<tr><td></td><td></td><td></td><td></td><td></td><td></td><td>2</td><td>11.62</td><td>3.12</td><td>81.9</td><td>(K)T/Y|N/S N/L/I/Q H\V\K(G)</td><td>659.4933</td><td>1316.696</td><td>1.2836</td></tr>
<tr><td></td><td></td><td></td><td></td><td></td><td></td><td>2</td><td>11.17</td><td>3.72</td><td>85</td><td>(K)H/W|G/L|F|L|P N\K(Q)</td><td>556.8049</td><td>1111.605</td><td>0.9978</td></tr>
<tr><td>1963</td><td>XP_002277446.1</td><td>Glucan endo-1,3-beta-glucosidase</td><td>5(35)</td><td>25%</td><td>85.42</td><td>2</td><td>19.72</td><td>10.28</td><td>95</td><td>(R)N G N N/L|P A|P G/E|V/V A/L|Y|N/Q Y N I R(R)</td><td>1158.8503</td><td>2316.173</td><td>0.5202</td></tr>
<tr><td></td><td></td><td>(Vitis vinifera)</td><td></td><td></td><td></td><td>2</td><td>18.32</td><td>7.29</td><td>95.4</td><td>(K)V S T A I/D/T G V|L|G/V|S|Y|P/P S S G S\F K(S)</td><td>1084.8198</td><td>2169.107</td><td>-0.4751</td></tr>
<tr><td></td><td></td><td></td><td></td><td></td><td></td><td>3</td><td>18.2</td><td>6.84</td><td>97.2</td><td>(K)T/Y|N/S/N|L|I|Q\H|V K(G)</td><td>439.9157</td><td>1316.696</td><td>1.0368</td></tr>
<tr><td></td><td></td><td></td><td></td><td></td><td></td><td>2</td><td>17.43</td><td>9.67</td><td>98.7</td><td>(K)V S T A I/D/T/G V|L|G|V|S/Y|P P S S G S\F K(S)</td><td>1085.356</td><td>2169.107</td><td>0.5973</td></tr>
<tr><td></td><td></td><td></td><td></td><td></td><td></td><td>2</td><td>17.2</td><td>6.36</td><td>87.7</td><td>(K)I V V/S E|S\G|W|P/S/A G/G\T Q\T\T\V\D N A R(T)</td><td>1117.4092</td><td>2232.089</td><td>1.722</td></tr>
<tr><td></td><td></td><td></td><td></td><td></td><td></td><td>3</td><td>17.02</td><td>7.52</td><td>92.3</td><td>(K)I V/V S E/S/G W|P S\A G G T Q/T|T|V/D N A\R(T)</td><td>745.1533</td><td>2232.089</td><td>1.3562</td></tr>
<tr><td></td><td></td><td></td><td></td><td></td><td></td><td>3</td><td>16.27</td><td>5.97</td><td>91.4</td><td>(K)V S/T A I|D T G V L\G\V|S/Y|P P S S G S F K(S)</td><td>723.7458</td><td>2169.107</td><td>0.1155</td></tr>
<tr><td></td><td></td><td></td><td></td><td></td><td></td><td>2</td><td>16.12</td><td>6.34</td><td>95.4</td><td>(K)T Y|N/S/N/L|I/Q/H\V\K(G)</td><td>659.1335</td><td>1316.696</td><td>0.564</td></tr>
<tr><td></td><td></td><td></td><td></td><td></td><td></td><td>3</td><td>15.62</td><td>5.59</td><td>92</td><td>(K)I V/V S E/S/G W|P S A\G G T\Q T|T V/D N A\R(T)</td><td>744.7854</td><td>2232.089</td><td>0.2525</td></tr>
<tr><td></td><td></td><td></td><td></td><td></td><td></td><td>2</td><td>15.51</td><td>7.96</td><td>90.6</td><td>(K)V S T A I D|T G V|L|G/V|S|Y|P P S S G S F K(S)</td><td>1085.4685</td><td>2169.107</td><td>0.8223</td></tr>
<tr><td></td><td></td><td></td><td></td><td></td><td></td><td>3</td><td>15.44</td><td>5.09</td><td>76.7</td><td>(R)N G N N/L|P\A P G E V\V\A\L|Y|N Q/Y\N I\R(R)</td><td>772.9719</td><td>2316.173</td><td>0.728</td></tr>
<tr><td></td><td></td><td></td><td></td><td></td><td></td><td>2</td><td>15.34</td><td>4.19</td><td>84.8</td><td>(K)I V/V S E|S G/W|P/S/A G/G T/Q T/T\V|D N A R(T)</td><td>1116.3076</td><td>2232.089</td><td>-0.4812</td></tr>
<tr><td></td><td></td><td></td><td></td><td></td><td></td><td>3</td><td>15.31</td><td>5.4</td><td>87.4</td><td>(K)I V/V/S E/S G W|P S\A G G T Q/T|T/V/D\N A R(T)</td><td>744.6816</td><td>2232.089</td><td>-0.0589</td></tr>
<tr><td></td><td></td><td></td><td></td><td></td><td></td><td>2</td><td>15.25</td><td>7.21</td><td>94.6</td><td>(K)V S T A I D/T/G V/L|G|V|S|Y|P P S S G S\F K(S)</td><td>1085.7607</td><td>2169.107</td><td>1.4067</td></tr>
<tr><td></td><td></td><td></td><td></td><td></td><td></td><td>2</td><td>15.05</td><td>6.32</td><td>90.4</td><td>(K)V S T A/I D T G V|L|G/V|S/Y|P P S\S G S\F K(S)</td><td>1085.0815</td><td>2169.107</td><td>0.0483</td></tr>
<tr><td></td><td></td><td></td><td></td><td></td><td></td><td>2</td><td>15.04</td><td>4.76</td><td>81</td><td>(K)I V/V/S E\S G|W|P S/A G/G T Q|T/T\V/D N A R(T)</td><td>1116.8423</td><td>2232.089</td><td>0.5882</td></tr>
<tr><td></td><td></td><td></td><td></td><td></td><td></td><td>2</td><td>14.52</td><td>10.05</td><td>92</td><td>(K)T Y/N/S/N/L|I|Q/H\V\K(G)</td><td>658.8229</td><td>1316.696</td><td>-0.0572</td></tr>
<tr><td></td><td></td><td></td><td></td><td></td><td></td><td>3</td><td>14.12</td><td>4.31</td><td>85.2</td><td>(K)I V/V/S E/S/G W|P S A G G T Q/T|T V/D N A\R(T)</td><td>744.9934</td><td>2232.089</td><td>0.8765</td></tr>
</table>

<table>
<tr><th></th><th></th><th></th><th></th><th></th><th></th><th></th><th></th><th></th><th></th><th></th><th></th><th></th><th></th></tr>
<tr><td></td><td></td><td></td><td></td><td></td><td></td><td>2</td><td>13.93</td><td>7.28</td><td>84.9</td><td>(K)T/Y|N/S/N/L|I|Q H\V\K(G)</td><td>659.2296</td><td>1316.696</td><td>0.7562</td></tr>
<tr><td></td><td></td><td></td><td></td><td></td><td></td><td>2</td><td>13.88</td><td>4.69</td><td>85.3</td><td>(K)V S T A I/D T G V|L|G/V|S/Y|P/P S S G S F K(S)</td><td>1085.1829</td><td>2169.107</td><td>0.2511</td></tr>
<tr><td></td><td></td><td></td><td></td><td></td><td></td><td>2</td><td>13.84</td><td>3.71</td><td>88.4</td><td>(K)V S T A I D T G V|L|G/V|S|Y|P P S S G S F K(S)</td><td>1085.1782</td><td>2169.107</td><td>0.2417</td></tr>
<tr><td></td><td></td><td></td><td></td><td></td><td></td><td>2</td><td>13.6</td><td>6.06</td><td>79.1</td><td>(K)T/Y|N|S/N/L|I/Q/H\V\K(G)</td><td>658.9011</td><td>1316.696</td><td>0.0992</td></tr>
<tr><td></td><td></td><td></td><td></td><td></td><td></td><td>2</td><td>13.52</td><td>2.68</td><td>80.8</td><td>(K)I V V/S E|S/G W|P|S A G G/T Q T/T V D N A R(T)</td><td>1116.7896</td><td>2232.089</td><td>0.4828</td></tr>
<tr><td></td><td></td><td></td><td></td><td></td><td></td><td>3</td><td>13.51</td><td>4.26</td><td>85.3</td><td>(K)V/S T A I\D T G V L\G\V|S/Y/P P\S S G S F K(S)</td><td>723.8053</td><td>2169.107</td><td>0.294</td></tr>
<tr><td></td><td></td><td></td><td></td><td></td><td></td><td>3</td><td>13.22</td><td>2.47</td><td>84.7</td><td>(K)I V/V S E\S/G/W|P S A G G T Q/T|T V/D N A R(T)</td><td>745.3455</td><td>2232.089</td><td>1.9328</td></tr>
<tr><td></td><td></td><td></td><td></td><td></td><td></td><td>3</td><td>12.25</td><td>5.12</td><td>100</td><td>(K)T/Y|N/S N|L|I/Q H V\K(G)</td><td>440.0555</td><td>1316.696</td><td>1.4562</td></tr>
<tr><td></td><td></td><td></td><td></td><td></td><td></td><td>2</td><td>12.14</td><td>3.86</td><td>84.6</td><td>(K)I V V/S E/S/G W|P/S/A G\G T Q T T\V\D N A R(T)</td><td>1116.7046</td><td>2232.089</td><td>0.3128</td></tr>
<tr><td></td><td></td><td></td><td></td><td></td><td></td><td>2</td><td>11.98</td><td>4.89</td><td>93.6</td><td>(K)H/W|G L|F|L|P N\K(Q)</td><td>556.2397</td><td>1111.605</td><td>-0.1326</td></tr>
<tr><td></td><td></td><td></td><td></td><td></td><td></td><td>3</td><td>11.8</td><td>2.03</td><td>73.5</td><td>(K)V S/T/A I\D T G V/L/G\V/S/Y/P\P S S G S F K(S)</td><td>724.2483</td><td>2169.107</td><td>1.623</td></tr>
<tr><td></td><td></td><td></td><td></td><td></td><td></td><td>2</td><td>11.79</td><td>4.52</td><td>84.3</td><td>(K)H/W|G/L|F|L|P\N\K(Q)</td><td>556.7302</td><td>1111.605</td><td>0.8484</td></tr>
<tr><td></td><td></td><td></td><td></td><td></td><td></td><td>3</td><td>11.53</td><td>2.98</td><td>89.8</td><td>(K)T/Y|N/S/N\L|I/Q H V K(G)</td><td>439.5507</td><td>1316.696</td><td>-0.0582</td></tr>
<tr><td></td><td></td><td></td><td></td><td></td><td></td><td>2</td><td>11.14</td><td>5.94</td><td>91.2</td><td>(K)H/W|G/L|F|L|P N\K(Q)</td><td>556.9205</td><td>1111.605</td><td>1.229</td></tr>
<tr><td></td><td></td><td></td><td></td><td></td><td></td><td>2</td><td>10.76</td><td>5.4</td><td>90.9</td><td>(K)T/Y/N/S/N/L|I/Q H V\K(G)</td><td>659.2837</td><td>1316.696</td><td>0.8644</td></tr>
<tr><td></td><td></td><td></td><td></td><td></td><td></td><td>2</td><td>10.74</td><td>3.44</td><td>92.4</td><td>(K)H/W|G L|F|L|P N\K(Q)</td><td>556.6782</td><td>1111.605</td><td>0.7444</td></tr>
<tr><td></td><td></td><td></td><td></td><td></td><td></td><td>3</td><td>10.7</td><td>4.05</td><td>97.7</td><td>(K)T/Y|N/S/N\L|I/Q H V K(G)</td><td>439.9528</td><td>1316.696</td><td>1.1481</td></tr>
<tr><td>1988</td><td>XP_002274796.1</td><td>Oxygen-evolving enhancer protein 1, chloroplastic</td><td>4(6)</td><td>19%</td><td>63.82</td><td>2</td><td>19.8</td><td>10.32</td><td>97.2</td><td>(K)S K/P E T G E|V|I|G V|F|E|S/I\Q|P S D/T D L G A K(T)</td><td>1302.9927</td><td>2604.304</td><td>0.6742</td></tr>
<tr><td></td><td></td><td>(Vitis vinifera)</td><td></td><td></td><td></td><td>2</td><td>15.75</td><td>5.37</td><td>88.7</td><td>(R)G G S T G/Y/D/N/A|V|A|L|P\A G G R(G)</td><td>782.7075</td><td>1562.756</td><td>1.652</td></tr>
<tr><td></td><td></td><td></td><td></td><td></td><td></td><td>2</td><td>14.89</td><td>6.12</td><td>98</td><td>(K)D G I D Y A/A|V|T|V|Q|L|P G G E R(V)</td><td>881.8506</td><td>1760.881</td><td>1.8126</td></tr>
<tr><td></td><td></td><td></td><td></td><td></td><td></td><td>3</td><td>14</td><td>4.87</td><td>100</td><td>(R)G/G S T/G\Y D N A\V|A\L|P/A G G R(G)</td><td>521.8608</td><td>1562.756</td><td>0.8121</td></tr>
<tr><td></td><td></td><td></td><td></td><td></td><td></td><td>3</td><td>13.38</td><td>3.05</td><td>87.1</td><td>(K)F E E K/D G I|D Y A A\V|T/V/Q|L|P G G E R(V)</td><td>765.4565</td><td>2294.13</td><td>0.225</td></tr>
<tr><td></td><td></td><td></td><td></td><td></td><td></td><td>3</td><td>13</td><td>6.9</td><td>100</td><td>(K)D G I/D Y A\A\V\T\V/Q|L|P/G G E R(V)</td><td>588.0125</td><td>1760.881</td><td>1.1416</td></tr>
<tr><td>2002</td><td>BAB78506.1</td><td>Xyloglucan endo-transglycosylase</td><td>2(5)</td><td>7%</td><td>32.6</td><td>2</td><td>18.47</td><td>8.13</td><td>98.8</td><td>(K)F/S/P/I|S|F|N|Q|G/F R(N)</td><td>650.3629</td><td>1299.648</td><td>0.0705</td></tr>
<tr><td></td><td></td><td>(Vitis labrusca x Vitis vinifera)</td><td></td><td></td><td></td><td>2</td><td>14.77</td><td>4.24</td><td>90.7</td><td>(K)F/S/P/I|S|F|N/Q|G F R(N)</td><td>650.4585</td><td>1299.648</td><td>0.2617</td></tr>
<tr><td></td><td></td><td></td><td></td><td></td><td></td><td>2</td><td>14.64</td><td>4.25</td><td>94.6</td><td>(K)F/S/P/I|S|F/N Q G F\R(N)</td><td>650.6779</td><td>1299.648</td><td>0.7005</td></tr>
<tr><td></td><td></td><td></td><td></td><td></td><td></td><td>2</td><td>14.48</td><td>3.79</td><td>88.2</td><td>(K)F/S/P/I|S|F|N Q\G\F\R(N)</td><td>650.3643</td><td>1299.648</td><td>0.0733</td></tr>
<tr><td></td><td></td><td></td><td></td><td></td><td></td><td>2</td><td>14.13</td><td>4.62</td><td>95.1</td><td>(R)S/G/Y|F/S\A A|I\K(L)</td><td>472.3149</td><td>943.488</td><td>0.1342</td></tr>
<tr><td>2028</td><td>XP_002267609.1</td><td>Remorin</td><td>3(3)</td><td>14%</td><td>39.75</td><td>2</td><td>16.07</td><td>6.85</td><td>92.4</td><td>(K)L S/A/T/E|A|W|E|N\S Q\K(A)</td><td>682.3989</td><td>1363.649</td><td>0.1417</td></tr>
<tr><td></td><td></td><td>(Vitis vinifera)</td><td></td><td></td><td></td><td>2</td><td>12.07</td><td>5.81</td><td>92.5</td><td>(K)K/L/S A/T/E/A/W\E|N S\Q\K(A)</td><td>746.7546</td><td>1491.744</td><td>0.7581</td></tr>
<tr><td></td><td></td><td></td><td></td><td></td><td></td><td>3</td><td>11.61</td><td>3.39</td><td>97.1</td><td>(R)R G E D|L\L|K|A/E E|m A A K(Y)</td><td>526.5319</td><td>1560.805</td><td>16.7761</td></tr>
<tr><td>2029</td><td>XP_002283483.1</td><td>Stem-specific protein TSJT1</td><td>5(5)</td><td>22%</td><td>78.02</td><td>2</td><td>18.89</td><td>10.35</td><td>98.1</td><td>(R)T/P|D|S|A|S L|P A\L\K(D)</td><td>550.2813</td><td>1099.599</td><td>-0.044</td></tr>
<tr><td></td><td></td><td>(Vitis vinifera)</td><td></td><td></td><td></td><td>3</td><td>17.54</td><td>4.32</td><td>92.2</td><td>(R)D R/G\P Y|P|A|D|Q|V|V\R(D)</td><td>458.2986</td><td>1372.697</td><td>0.1845</td></tr>
<tr><td></td><td></td><td></td><td></td><td></td><td></td><td>2</td><td>15.61</td><td>5.06</td><td>90.6</td><td>(K)F A|F|V|L|Y|D/S|S|N R(T)</td><td>660.3046</td><td>1318.643</td><td>0.9593</td></tr>
<tr><td></td><td></td><td></td><td></td><td></td><td></td><td>2</td><td>14.47</td><td>5.05</td><td>90.1</td><td>(R)S F|E/H|P/L|N E|L\R(A)</td><td>621.4529</td><td>1241.627</td><td>0.2712</td></tr>
<tr><td></td><td></td><td></td><td></td><td></td><td></td><td>2</td><td>11.51</td><td>3</td><td>94.8</td><td>(K)S I|A|E|A|P E/G L R(T)</td><td>521.6846</td><td>1042.553</td><td>-0.1908</td></tr>
<tr><td>2104</td><td>XP_010654260.1</td><td>Uncharacterized protein At5g02240</td><td>5(10)</td><td>15%</td><td>82.65</td><td>2</td><td>19.58</td><td>4.93</td><td>94.9</td><td>(R)E/L|L|V/G/K/D/D|E\L\L\K(T)</td><td>686.645</td><td>1371.773</td><td>0.5098</td></tr>
<tr><td></td><td></td><td>(Vitis vinifera)</td><td></td><td></td><td></td><td>2</td><td>17.74</td><td>7.41</td><td>95.8</td><td>(K)A F|D|L|A|S|N|P E\R(V)</td><td>560.4009</td><td>1119.543</td><td>0.2516</td></tr>
<tr><td></td><td></td><td></td><td></td><td></td><td></td><td>2</td><td>16.05</td><td>9.37</td><td>91.2</td><td>(K)I/G/G A/D|D|V/L|V|G D|I R(N)</td><td>650.6938</td><td>1299.69</td><td>0.69</td></tr>
<tr><td></td><td></td><td></td><td></td><td></td><td></td><td>2</td><td>15.69</td><td>10.65</td><td>90.8</td><td>(K)I/G G A/D|D|V/L|V|G D|I R(N)</td><td>651.0527</td><td>1299.69</td><td>1.4078</td></tr>
<tr><td></td><td></td><td></td><td></td><td></td><td></td><td>2</td><td>15.5</td><td>8.91</td><td>100</td><td>(K)I/G/G A D|D|V/L|V|G\D|I R(N)</td><td>650.2672</td><td>1299.69</td><td>-0.1632</td></tr>
<tr><td></td><td></td><td></td><td></td><td></td><td></td><td>2</td><td>15.23</td><td>7.71</td><td>92</td><td>(K)I/G G A/D/D|V/L|V|G D|I\R(N)</td><td>650.978</td><td>1299.69</td><td>1.2584</td></tr>
<tr><td></td><td></td><td></td><td></td><td></td><td></td><td>2</td><td>15.17</td><td>5.9</td><td>94.7</td><td>(K)A/L|F|S|Q|I|S|T R(F)</td><td>511.9322</td><td>1022.563</td><td>0.2942</td></tr>
<tr><td></td><td></td><td></td><td></td><td></td><td></td><td>3</td><td>14.11</td><td>6.34</td><td>81.7</td><td>(R)E L|L/V/G/K D/D|E L\L/K/T E|T R(T)</td><td>620.6931</td><td>1859.012</td><td>1.0527</td></tr>
<tr><td></td><td></td><td></td><td></td><td></td><td></td><td>2</td><td>13.94</td><td>4.28</td><td>91.5</td><td>(K)A F|D|L|A|S|N|P E R(V)</td><td>560.2839</td><td>1119.543</td><td>0.0176</td></tr>
<tr><td></td><td></td><td></td><td></td><td></td><td></td><td>2</td><td>11.18</td><td>6.29</td><td>81.4</td><td>(K)I/G G A/D D|V/L|V|G D|I R(N)</td><td>650.6195</td><td>1299.69</td><td>0.5414</td></tr>
<tr><td>2107</td><td>XP_002284767.1</td><td>L-ascorbate peroxidase 2, cytosolic</td><td>12(19)</td><td>54%</td><td>202.78</td><td>2</td><td>24</td><td>15.13</td><td>100</td><td>(K)Y/A|A|D/E|D|A|F|F|E|D|Y\K(E)</td><td>792.3931</td><td>1583.654</td><td>0.1253</td></tr>
<tr><td></td><td></td><td>(Vitis vinifera)</td><td></td><td></td><td></td><td>2</td><td>20.81</td><td>9.47</td><td>93.7</td><td>(K)A/L/L/S/D|P/A/F|R|P L\V\E\K(Y)</td><td>779.4404</td><td>1555.884</td><td>1.9893</td></tr>
<tr><td></td><td></td><td></td><td></td><td></td><td></td><td>3</td><td>19.83</td><td>9.57</td><td>97.9</td><td>(R)Q V/F/V/T Q/M/G L/S D/K D I/V|A/L/S/G A/H\T L G R(C)</td><td>881.66</td><td>2643.392</td><td>-0.4269</td></tr>
<tr><td></td><td></td><td></td><td></td><td></td><td></td><td>2</td><td>19.26</td><td>8.33</td><td>92.7</td><td>(K)A L/L|S/D|P/A/F|R|P L\V\E\K(Y)</td><td>779.0356</td><td>1555.884</td><td>1.1797</td></tr>
<tr><td></td><td></td><td></td><td></td><td></td><td></td><td>2</td><td>19.1</td><td>14.49</td><td>93.8</td><td>(K)K/P/E/E/L/A|H|G|A N N|G L D/I A\V R(L)</td><td>953.4825</td><td>1903.998</td><td>1.9593</td></tr>
<tr><td></td><td></td><td></td><td></td><td></td><td></td><td>2</td><td>18.8</td><td>10.08</td><td>96.3</td><td>(R)S G F E/G/P W/T|S/N|P L/I|F|D|N S Y F K(E)</td><td>1153.7563</td><td>2306.076</td><td>0.4289</td></tr>
<tr><td></td><td></td><td></td><td></td><td></td><td></td><td>2</td><td>18.72</td><td>8.24</td><td>95.6</td><td>(R)S G F E/G/P W/T/S/N|P L|I|F|D|N S Y F K(E)</td><td>1154.1162</td><td>2306.076</td><td>1.1487</td></tr>
<tr><td></td><td></td><td></td><td></td><td></td><td></td><td>2</td><td>16.32</td><td>6.5</td><td>88.2</td><td>(K)D I|V|A|L|S|G/A/H/T\L G R(C)</td><td>655.781</td><td>1309.722</td><td>0.8324</td></tr>
<tr><td></td><td></td><td></td><td></td><td></td><td></td><td>3</td><td>16.22</td><td>3.35</td><td>98.1</td><td>(K)A/L|L|S|D|P/A F R P\L V E K(Y)</td><td>519.5244</td><td>1555.884</td><td>0.6744</td></tr>
<tr><td></td><td></td><td></td><td></td><td></td><td></td><td>2</td><td>15.72</td><td>4.96</td><td>93.6</td><td>(R)Q V/F/V|T/Q/M/G/L|S D K(D)</td><td>676.7832</td><td>1352.688</td><td>-0.1287</td></tr>
<tr><td></td><td></td><td></td><td></td><td></td><td></td><td>2</td><td>15.39</td><td>6.21</td><td>88.9</td><td>(R)I/A/W/H|S|A G/T|F\D|V\K(T)</td><td>666.3815</td><td>1331.674</td><td>0.0815</td></tr>
<tr><td></td><td></td><td></td><td></td><td></td><td></td><td>3</td><td>15.22</td><td>5.74</td><td>98.4</td><td>(K)Y/A|A|D|E/D|A/F/F/E D Y K E/A H L K(L)</td><td>721.6606</td><td>2161.971</td><td>0.996</td></tr>
<tr><td></td><td></td><td></td><td></td><td></td><td></td><td>3</td><td>15.16</td><td>4.66</td><td>90.3</td><td>(R)I/A|W/H|S A|G/T|F\D|V\K(T)</td><td>444.5828</td><td>1331.674</td><td>0.0596</td></tr>
<tr><td></td><td></td><td></td><td></td><td></td><td></td><td>2</td><td>14.58</td><td>3.51</td><td>96.3</td><td>(K)E G|L|I|Q/L|P/S D K(A)</td><td>550.2996</td><td>1099.599</td><td>-0.0074</td></tr>
<tr><td></td><td></td><td></td><td></td><td></td><td></td><td>3</td><td>12.68</td><td>4.97</td><td>94.5</td><td>(K)K/P/E E L A|H|G/A N|N|G L/D I A V R(L)</td><td>635.9489</td><td>1903.998</td><td>1.8337</td></tr>
<tr><td></td><td></td><td></td><td></td><td></td><td></td><td>2</td><td>12.11</td><td>2.85</td><td>92.8</td><td>(K)S Y|P/T/V|S/E E/Y K\K(A)</td><td>665.7744</td><td>1330.652</td><td>-0.111</td></tr>
<tr><td></td><td></td><td></td><td></td><td></td><td></td><td>3</td><td>12.02</td><td>7.4</td><td>84.9</td><td>(R)Q V/F/V T Q/M/G\L/S D K D I/V/A/L/S/G/A H T L G R(C)</td><td>882.1683</td><td>2643.392</td><td>1.098</td></tr>
<tr><td></td><td></td><td></td><td></td><td></td><td></td><td>2</td><td>11.35</td><td>2.45</td><td>72.8</td><td>(K)A/L/L/S D/P/A F|R|P L V\E\K(Y)</td><td>777.8685</td><td>1555.884</td><td>-1.1545</td></tr>
<tr><td></td><td></td><td></td><td></td><td></td><td></td><td>2</td><td>10.9</td><td>2.61</td><td>81.9</td><td>(K)E L/L S G E/K/E|G L/I/Q/L|P S D\K(A)</td><td>928.7573</td><td>1856.001</td><td>0.5062</td></tr>
<tr><td>2117</td><td>P51117.1</td><td>Chalcone-flavonone isomerase 1</td><td>4(6)</td><td>22%</td><td>67.5</td><td>2</td><td>19.05</td><td>13.08</td><td>95.8</td><td>(K)L L/T E/A|V|L|E|S|I|I\G K(H)</td><td>693.4446</td><td>1385.825</td><td>0.0569</td></tr>
<tr><td></td><td></td><td>(Vitis vinifera)</td><td></td><td></td><td></td><td>2</td><td>18.86</td><td>6.65</td><td>95.9</td><td>(K)D G S|L|P E/V|G/N A/V|I|E\N\K(L)</td><td>771.6783</td><td>1541.781</td><td>0.5687</td></tr>
</table>

<table>
<tr><td></td><td></td><td></td><td></td><td></td><td></td><td>2</td><td>15.65</td><td>7.67</td><td>87</td><td>(K)T V/E/E L|A|D/S|V|D/F\F\R(D)</td><td>764.3772</td><td>1527.733</td><td>0.0146</td></tr>
<tr><td></td><td></td><td></td><td></td><td></td><td></td><td>2</td><td>14.5</td><td>7.85</td><td>100</td><td>(K)T V/E/E/L/A/D/S|V/D|F|F R(D)</td><td>764.1923</td><td>1527.733</td><td>-0.3552</td></tr>
<tr><td></td><td></td><td></td><td></td><td></td><td></td><td>2</td><td>13.94</td><td>4.24</td><td>97.6</td><td>(K)S/V|G|I|Y|T/D A E\A K(A)</td><td>577.429</td><td>1153.574</td><td>0.2772</td></tr>
<tr><td></td><td></td><td></td><td></td><td></td><td></td><td>2</td><td>13.87</td><td>4.04</td><td>95.9</td><td>(K)D G S|L/P E/V|G/N A V|I|E\N\K(L)</td><td>771.3522</td><td>1541.781</td><td>-0.0835</td></tr>
<tr><td>2124</td><td>XP_010657212.1</td><td>Carboxymethylenebutenolidase homolog</td><td>5(5)</td><td>20%</td><td>74.21</td><td>2</td><td>18.72</td><td>10.97</td><td>95</td><td>(R)D D/T/T/F|D|A|Y|V|V|G K(E)</td><td>666.1614</td><td>1330.616</td><td>0.6994</td></tr>
<tr><td></td><td></td><td>(Vitis vinifera)</td><td></td><td></td><td></td><td>2</td><td>15.75</td><td>4.58</td><td>100</td><td>(K)I/S|Q|L|D|P G\F K(A)</td><td>503.0596</td><td>1004.541</td><td>0.5708</td></tr>
<tr><td></td><td></td><td></td><td></td><td></td><td></td><td>2</td><td>14.23</td><td>8.05</td><td>87.9</td><td>(K)G m G m/P D E/D/E D|A/V|G L/A/W S R(F)</td><td>984.536</td><td>1935.821</td><td>32.2436</td></tr>
<tr><td></td><td></td><td></td><td></td><td></td><td></td><td>2</td><td>12.91</td><td>2.77</td><td>92.9</td><td>(K)D I|C|A|S/V|N/W\L K(A)</td><td>603.7734</td><td>1205.598</td><td>0.9412</td></tr>
<tr><td></td><td></td><td></td><td></td><td></td><td></td><td>3</td><td>12.6</td><td>4.59</td><td>90.3</td><td>(R)K G m G m|P/D E D E D\A|V G|L|A|W\S R(F)</td><td>699.6023</td><td>2063.916</td><td>32.8762</td></tr>
<tr><td>2161</td><td>4L5H_A</td><td>Thaumatin-like Proteins</td><td>3(15)</td><td>14%</td><td>50.68</td><td>2</td><td>17.5</td><td>8.85</td><td>93.7</td><td>(R)R L/D|S/G Q/S/W/T|I|T|V|N|P G T T N\A R(I)</td><td>1088.2783</td><td>2174.095</td><td>1.4545</td></tr>
<tr><td></td><td></td><td>(Vitis vinifera)</td><td></td><td></td><td></td><td>2</td><td>16.59</td><td>4.37</td><td>92.6</td><td>(-)A/T|F/D|I|L|N\K(C)</td><td>461.68</td><td>921.504</td><td>0.8487</td></tr>
<tr><td></td><td></td><td></td><td></td><td></td><td></td><td>3</td><td>16.59</td><td>7.52</td><td>95.2</td><td>(R)L D S G Q\S/W|T\I|T\V|N/P/G/T T\N A R(I)</td><td>673.6459</td><td>2017.994</td><td>0.9294</td></tr>
<tr><td></td><td></td><td></td><td></td><td></td><td></td><td>2</td><td>16.51</td><td>7.55</td><td>88.5</td><td>(R)R L/D|S G Q|S/W/T/I/T V|N|P G T T N\A R(I)</td><td>1087.968</td><td>2174.095</td><td>0.8339</td></tr>
<tr><td></td><td></td><td></td><td></td><td></td><td></td><td>2</td><td>16.3</td><td>5.31</td><td>91.8</td><td>(R)R/L D|S/G Q S/W/T|I|T\V|N|P G T T N\A R(I)</td><td>1087.7964</td><td>2174.095</td><td>0.4907</td></tr>
<tr><td></td><td></td><td></td><td></td><td></td><td></td><td>2</td><td>15.36</td><td>7.56</td><td>88.1</td><td>(R)R L D|S/G Q S/W/T\I|T\V|N|P G T T N\A R(I)</td><td>1087.5283</td><td>2174.095</td><td>-0.0455</td></tr>
<tr><td></td><td></td><td></td><td></td><td></td><td></td><td>2</td><td>15.12</td><td>6.53</td><td>83.2</td><td>(R)R L/D|S G Q S/W|T/I|T|V|N|P\G T T N A R(I)</td><td>1088.5171</td><td>2174.095</td><td>1.9321</td></tr>
<tr><td></td><td></td><td></td><td></td><td></td><td></td><td>2</td><td>14.65</td><td>5.79</td><td>95.8</td><td>(-)A/T|F/D|I|L|N\K(C)</td><td>461.4043</td><td>921.504</td><td>0.2973</td></tr>
<tr><td></td><td></td><td></td><td></td><td></td><td></td><td>2</td><td>14.16</td><td>7.77</td><td>89</td><td>(R)R L D|S G Q\S/W|T\I/T|V\N|P G T T N\A R(I)</td><td>1088.2241</td><td>2174.095</td><td>1.3461</td></tr>
<tr><td></td><td></td><td></td><td></td><td></td><td></td><td>2</td><td>14.11</td><td>4.49</td><td>92</td><td>(R)L/D/S G Q S/W/T/I/T/V|N|P G T T\N A R(I)</td><td>1009.6713</td><td>2017.994</td><td>0.3416</td></tr>
<tr><td></td><td></td><td></td><td></td><td></td><td></td><td>2</td><td>12.95</td><td>4.4</td><td>97.9</td><td>(-)A/T|F/D|I|L|N\K(C)</td><td>461.7375</td><td>921.504</td><td>0.9637</td></tr>
<tr><td></td><td></td><td></td><td></td><td></td><td></td><td>2</td><td>12.5</td><td>3.83</td><td>100</td><td>(-)A/T|F/D|I|L|N\K(C)</td><td>461.4882</td><td>921.504</td><td>0.4651</td></tr>
<tr><td></td><td></td><td></td><td></td><td></td><td></td><td>2</td><td>12.08</td><td>4.46</td><td>93.2</td><td>(-)A/T|F/D/I|L|N\K(C)</td><td>461.4947</td><td>921.504</td><td>0.4781</td></tr>
<tr><td></td><td></td><td></td><td></td><td></td><td></td><td>2</td><td>11</td><td>3.24</td><td>100</td><td>(-)A T|F/D|I|L|N\K(C)</td><td>461.5159</td><td>921.504</td><td>0.5205</td></tr>
<tr><td></td><td></td><td></td><td></td><td></td><td></td><td>2</td><td>10.45</td><td>3.75</td><td>97.6</td><td>(-)A/T|F/D/I|L|N K(C)</td><td>461.3823</td><td>921.504</td><td>0.2533</td></tr>
<tr><td>2172</td><td>4L5H_A</td><td>Thaumatin-like Proteins</td><td>5(7)</td><td>28%</td><td>82.16</td><td>3</td><td>20.38</td><td>7.61</td><td>97.8</td><td>(R)L D S G Q\S/W|T|I|T\V|N|P/G/T T\N A R(I)</td><td>673.2769</td><td>2017.994</td><td>-0.1776</td></tr>
<tr><td></td><td></td><td>(Vitis vinifera)</td><td></td><td></td><td></td><td>2</td><td>17.65</td><td>4.93</td><td>97.1</td><td>(K)T S/L/F|T|C|P S/G T N\Y\K(V)</td><td>738.4849</td><td>1475.684</td><td>0.279</td></tr>
<tr><td></td><td></td><td></td><td></td><td></td><td></td><td>2</td><td>16.35</td><td>4.71</td><td>94.7</td><td>(-)A/T|F/D|I|L|N\K(C)</td><td>461.6103</td><td>921.504</td><td>0.7093</td></tr>
<tr><td></td><td></td><td></td><td></td><td></td><td></td><td>2</td><td>15.34</td><td>5.99</td><td>94.3</td><td>(R)R L D|S G Q\S/W|T|I|T\V|N|P G T T N A R(I)</td><td>1087.9287</td><td>2174.095</td><td>0.7553</td></tr>
<tr><td></td><td></td><td></td><td></td><td></td><td></td><td>3</td><td>13.99</td><td>3.69</td><td>83.7</td><td>(R)R L D S G Q\S/W|T|I|T|V|N|P/G/T T N A R(I)</td><td>725.8992</td><td>2174.095</td><td>1.5882</td></tr>
<tr><td></td><td></td><td></td><td></td><td></td><td></td><td>2</td><td>13.87</td><td>6.07</td><td>88.5</td><td>(R)L/D S G Q S/W/T/I/T/V|N|P G T T\N A R(I)</td><td>1009.3541</td><td>2017.994</td><td>-0.2928</td></tr>
<tr><td></td><td></td><td></td><td></td><td></td><td></td><td>2</td><td>12.44</td><td>8.61</td><td>93.1</td><td>(K)C T Y T/V|W A|A|A|S|P G G G R(R)</td><td>777.6558</td><td>1553.717</td><td>0.5878</td></tr>
<tr><td>2204</td><td>AAZ93634.1</td><td>MSA</td><td>1(4)</td><td>14%</td><td>22.75</td><td>3</td><td>22.75</td><td>10.81</td><td>96.3</td><td>(K)H L/E/H L\G E\L/G|V|A|A|A|G|A/Y|A\L\H\E\K(H)</td><td>729.3257</td><td>2186.135</td><td>-0.1727</td></tr>
<tr><td></td><td></td><td>(Vitis vinifera)</td><td></td><td></td><td></td><td>3</td><td>15.75</td><td>7.51</td><td>96.1</td><td>(K)H L/E/H/L G E L G\V|A|A|A|G|A/Y/A\L\H E K(H)</td><td>729.494</td><td>2186.135</td><td>0.3322</td></tr>
<tr><td></td><td></td><td></td><td></td><td></td><td></td><td>3</td><td>13.68</td><td>4.55</td><td>79.9</td><td>(K)H L/E H L G\E L G\V\A|A|A|G|A\Y A\L\H E\K(H)</td><td>729.8792</td><td>2186.135</td><td>1.4878</td></tr>
<tr><td></td><td></td><td></td><td></td><td></td><td></td><td>3</td><td>13.22</td><td>5.14</td><td>94.5</td><td>(K)H L/E H L G E L G\V|A|A|A|G|A Y A\L\H\E K(H)</td><td>729.5854</td><td>2186.135</td><td>0.6064</td></tr>
<tr><td></td><td></td><td></td><td></td><td></td><td></td><td></td><td></td><td></td><td></td><td>MS/MS spectrum is reported at the end of the table</td><td></td><td></td><td></td></tr>
<tr><td>2227</td><td>XP_002280658.1</td><td>Stem-specific protein TSJT1</td><td>4(6)</td><td>22%</td><td>67.09</td><td>3</td><td>18.31</td><td>10.56</td><td>91.9</td><td>(K)I T/A\V|P A|P D\E E|I|W|G\A|T F K(V)</td><td>615.991</td><td>1844.943</td><td>1.0156</td></tr>
<tr><td></td><td></td><td>(Vitis vinifera)</td><td></td><td></td><td></td><td>2</td><td>17.13</td><td>8.43</td><td>97.4</td><td>(K)I/T|A/E|A|L|m|N R(F)</td><td>518.3032</td><td>1018.535</td><td>17.0641</td></tr>
<tr><td></td><td></td><td></td><td></td><td></td><td></td><td>2</td><td>16.46</td><td>7.79</td><td>90.5</td><td>(K)S A N/E|V|V|L V|I|E|A\Y\K(A)</td><td>718.1888</td><td>1434.784</td><td>0.5865</td></tr>
<tr><td></td><td></td><td></td><td></td><td></td><td></td><td>2</td><td>15.72</td><td>6.82</td><td>94.3</td><td>(K)I/T|A/E|A|L|M/N R(F)</td><td>509.8807</td><td>1018.535</td><td>0.2191</td></tr>
<tr><td></td><td></td><td></td><td></td><td></td><td></td><td>2</td><td>15.19</td><td>6.25</td><td>86.6</td><td>(K)S/T S T/L|F|V/A|S|D Q\F\G K(V)</td><td>744.5232</td><td>1487.738</td><td>0.3015</td></tr>
<tr><td></td><td></td><td></td><td></td><td></td><td></td><td>3</td><td>12.33</td><td>3.03</td><td>89.8</td><td>(K)S/T/S T\L F|V|A|S/D/Q F G K(V)</td><td>496.7887</td><td>1487.738</td><td>0.6139</td></tr>
<tr><td>2231</td><td>CAB85637.1</td><td>Putative thaumatin-like protein</td><td>1(1)</td><td>9%</td><td>16.29</td><td>3</td><td>16.29</td><td>5.23</td><td>86.5</td><td>(R)R L D S G Q/S|W/T|I|T|V|N/P/G T\T N A R(I)</td><td>725.6035</td><td>2174.095</td><td>0.7011</td></tr>
<tr><td></td><td></td><td>(Vitis vinifera)</td><td></td><td></td><td></td><td></td><td></td><td></td><td></td><td>MS/MS spectrum is reported at the end of the table</td><td></td><td></td><td></td></tr>
<tr><td>2264</td><td>XP_002283286.1</td><td>NAD(P)H dehydrogenase (quinone) FQR1</td><td>8(34)</td><td>46%</td><td>129.4</td><td>2</td><td>19.74</td><td>3.61</td><td>96.7</td><td>(K)A F|L|D|A|T|G|G/L|W\R(T)</td><td>603.9612</td><td>1206.627</td><td>0.2885</td></tr>
<tr><td></td><td></td><td>(Vitis vinifera)</td><td></td><td></td><td></td><td>2</td><td>19.55</td><td>4.86</td><td>96.6</td><td>(K)A F|L|D|A|T|G|G/L|W\R(T)</td><td>604.3835</td><td>1206.627</td><td>1.1331</td></tr>
<tr><td></td><td></td><td></td><td></td><td></td><td></td><td>3</td><td>17.9</td><td>5.86</td><td>88.1</td><td>(R)Q/P/S/E/L/E/L\E|Q/A|F|H Q G\K(Y)</td><td>581.3486</td><td>1740.855</td><td>1.1761</td></tr>
<tr><td></td><td></td><td></td><td></td><td></td><td></td><td>2</td><td>17.8</td><td>8.47</td><td>95.8</td><td>(K)G G/S/P Y|G/A|G/T F/A|G/D|G S R(Q)</td><td>728.7053</td><td>1456.645</td><td>-0.2418</td></tr>
<tr><td></td><td></td><td></td><td></td><td></td><td></td><td>3</td><td>17.7</td><td>6.61</td><td>93.4</td><td>(R)Q P/S/E/L/E/L\E/Q/A|F|H Q G\K(Y)</td><td>581.0712</td><td>1740.855</td><td>0.3439</td></tr>
<tr><td></td><td></td><td></td><td></td><td></td><td></td><td>2</td><td>17.52</td><td>6.06</td><td>91.7</td><td>(K)A F|L|D|A|T\G|G\L|W\R(T)</td><td>604.1296</td><td>1206.627</td><td>0.6253</td></tr>
<tr><td></td><td></td><td></td><td></td><td></td><td></td><td>2</td><td>17.12</td><td>5.07</td><td>98.2</td><td>(K)A F|L/D|A|T|G|G/L|W R(T)</td><td>604.0833</td><td>1206.627</td><td>0.5327</td></tr>
<tr><td></td><td></td><td></td><td></td><td></td><td></td><td>2</td><td>17</td><td>5.1</td><td>100</td><td>(K)A F|L|D/A|T|G|G/L|W\R(T)</td><td>603.7964</td><td>1206.627</td><td>-0.0411</td></tr>
<tr><td></td><td></td><td></td><td></td><td></td><td></td><td>2</td><td>16.69</td><td>6.36</td><td>94.8</td><td>(K)G G/S/P Y/G/A|G/T|F/A|G D G S R(Q)</td><td>729.0149</td><td>1456.645</td><td>0.3774</td></tr>
<tr><td></td><td></td><td></td><td></td><td></td><td></td><td>2</td><td>16.64</td><td>6.68</td><td>93.6</td><td>(K)G G/S/P Y/G/A|G/T/F/A/G D|G S R(Q)</td><td>728.9307</td><td>1456.645</td><td>0.209</td></tr>
<tr><td></td><td></td><td></td><td></td><td></td><td></td><td>3</td><td>16.63</td><td>7.01</td><td>88.3</td><td>(R)Q P/S/E/L/E/L\E|Q/A/F|H Q G\K(Y)</td><td>581.2256</td><td>1740.855</td><td>0.8071</td></tr>
<tr><td></td><td></td><td></td><td></td><td></td><td></td><td>2</td><td>16.51</td><td>6.71</td><td>91.3</td><td>(K)G A/A|S|V|E\G|V|E|A\K(L)</td><td>508.9807</td><td>1017.521</td><td>-0.567</td></tr>
<tr><td></td><td></td><td></td><td></td><td></td><td></td><td>2</td><td>15.79</td><td>4.99</td><td>94.8</td><td>(K)G A/A|S|V|E\G/V|E\A\K(L)</td><td>509.3863</td><td>1017.521</td><td>0.2442</td></tr>
<tr><td></td><td></td><td></td><td></td><td></td><td></td><td>3</td><td>15.12</td><td>6.38</td><td>87</td><td>(K)V K/G G S P Y\G A/G T|F A|G|D|G\S R(Q)</td><td>562.344</td><td>1683.809</td><td>1.2089</td></tr>
<tr><td></td><td></td><td></td><td></td><td></td><td></td><td>2</td><td>14.71</td><td>6.59</td><td>83.1</td><td>(R)F/G|M|M|A\A|Q|F K(A)</td><td>515.7483</td><td>1030.485</td><td>0.0045</td></tr>
<tr><td></td><td></td><td></td><td></td><td></td><td></td><td>2</td><td>14.54</td><td>2.45</td><td>90.1</td><td>(K)L W/Q|V|P E/T|L|P E E V L\G K(M)</td><td>869.3668</td><td>1737.942</td><td>-0.2158</td></tr>
<tr><td></td><td></td><td></td><td></td><td></td><td></td><td>3</td><td>14.08</td><td>5.31</td><td>90.2</td><td>(R)Q P/S E/L/E/L\E Q/A\F/H Q G\K(Y)</td><td>581.4153</td><td>1740.855</td><td>1.3762</td></tr>
<tr><td></td><td></td><td></td><td></td><td></td><td></td><td>2</td><td>14.05</td><td>3.04</td><td>89.6</td><td>(K)L W\Q|V|P E|T|L|P E E V\L\G K(M)</td><td>869.7268</td><td>1737.942</td><td>0.5042</td></tr>
<tr><td></td><td></td><td></td><td></td><td></td><td></td><td>2</td><td>13.79</td><td>4.69</td><td>94.8</td><td>(K)G G/S/P Y/G/A/G/T/F A/G D|G S R(Q)</td><td>729.1699</td><td>1456.645</td><td>0.6874</td></tr>
<tr><td></td><td></td><td></td><td></td><td></td><td></td><td>3</td><td>13.38</td><td>3.85</td><td>97</td><td>(R)Q P/S/E/L/E/L E Q/A|F H Q G\K(Y)</td><td>581.1772</td><td>1740.855</td><td>0.6619</td></tr>
<tr><td></td><td></td><td></td><td></td><td></td><td></td><td>2</td><td>13.23</td><td>5.65</td><td>81.5</td><td>(R)F/G|M|M|A\A|Q|F K(A)</td><td>515.991</td><td>1030.485</td><td>0.4899</td></tr>
<tr><td></td><td></td><td></td><td></td><td></td><td></td><td>2</td><td>13.13</td><td>4.47</td><td>87</td><td>(R)F/G|M|M|A\A|Q|F K(A)</td><td>516.2283</td><td>1030.485</td><td>0.9645</td></tr>
<tr><td></td><td></td><td></td><td></td><td></td><td></td><td>2</td><td>13.08</td><td>4.01</td><td>72.8</td><td>(K)V Y\I|V|Y/Y/S/M Y/G|H|V E\K(L)</td><td>876.0173</td><td>1750.851</td><td>0.1764</td></tr>
</table>

<table>
<tr><td></td><td></td><td></td><td></td><td></td><td></td><td>2</td><td>12.82</td><td>5.23</td><td>92.1</td><td>(R)F/G/m|M|A|A|Q|F K(A)</td><td>523.5667</td><td>1030.485</td><td>15.6413</td></tr>
<tr><td></td><td></td><td></td><td></td><td></td><td></td><td>2</td><td>12.78</td><td>3.09</td><td>82.6</td><td>(K)G A/A/S/V|E\G/V|E\A\K(L)</td><td>509.8121</td><td>1017.521</td><td>1.0958</td></tr>
<tr><td></td><td></td><td></td><td></td><td></td><td></td><td>2</td><td>12.5</td><td>5.97</td><td>100</td><td>(K)A F|L/D\A|T\G\G\L|W R(T)</td><td>603.9288</td><td>1206.627</td><td>0.2237</td></tr>
<tr><td></td><td></td><td></td><td></td><td></td><td></td><td>3</td><td>12.44</td><td>4.42</td><td>86.7</td><td>(R)Q P/S/E/L/E/L\E Q/A|F H Q G\K(Y)</td><td>580.4996</td><td>1740.855</td><td>-1.3709</td></tr>
<tr><td></td><td></td><td></td><td></td><td></td><td></td><td>2</td><td>12.43</td><td>5.71</td><td>81.8</td><td>(R)F/G|M|M|A\A|Q|F K(A)</td><td>515.6079</td><td>1030.485</td><td>-0.2763</td></tr>
<tr><td></td><td></td><td></td><td></td><td></td><td></td><td>2</td><td>12.43</td><td>7.09</td><td>84.7</td><td>(R)F/G|M|M|A\A|Q|F K(A)</td><td>515.9104</td><td>1030.485</td><td>0.3287</td></tr>
<tr><td></td><td></td><td></td><td></td><td></td><td></td><td>2</td><td>12.26</td><td>2.82</td><td>91.4</td><td>(K)A F/L/D/A|T\G|G L|W\R(T)</td><td>604.3069</td><td>1206.627</td><td>0.9799</td></tr>
<tr><td></td><td></td><td></td><td></td><td></td><td></td><td>2</td><td>11.79</td><td>3.74</td><td>84.8</td><td>(K)G A/A/S/V|E\G V|E\A K(L)</td><td>509.3821</td><td>1017.521</td><td>0.2358</td></tr>
<tr><td></td><td></td><td></td><td></td><td></td><td></td><td>2</td><td>11.53</td><td>2.14</td><td>77.5</td><td>(K)L/W Q|V|P E/T|L|P E E V\L G K(M)</td><td>869.3647</td><td>1737.942</td><td>-0.22</td></tr>
<tr><td></td><td></td><td></td><td></td><td></td><td></td><td>2</td><td>10.82</td><td>4.11</td><td>89.3</td><td>(K)G G/S/P Y/G/A/G T/F/A/G D\G S R(Q)</td><td>729.4482</td><td>1456.645</td><td>1.244</td></tr>
<tr><td></td><td></td><td></td><td></td><td></td><td></td><td>2</td><td>10.1</td><td>5</td><td>85.1</td><td>(R)Q P/S E L E L|E/Q/A/F\H Q G\K(Y)</td><td>871.1501</td><td>1740.855</td><td>0.4378</td></tr>
<tr><td>2269</td><td>AKG51696.1</td><td>Superoxide dismutase</td><td>3(4)</td><td>17%</td><td>51.51</td><td>2</td><td>19.13</td><td>5.56</td><td>96.7</td><td>(K)N L|T|P/V/H/E|G G G E|P\P K(G)</td><td>716.5894</td><td>1431.723</td><td>0.4489</td></tr>
<tr><td></td><td></td><td>(Vitis vinifera)</td><td></td><td></td><td></td><td>2</td><td>17.58</td><td>11.74</td><td>94.5</td><td>(K)L V/V/E|T/T/A/N|Q|D|P L V\T K(G)</td><td>814.2173</td><td>1627.89</td><td>-0.4628</td></tr>
<tr><td></td><td></td><td></td><td></td><td></td><td></td><td>2</td><td>14.8</td><td>6.6</td><td>95.6</td><td>(K)A/L/E/Q/L/H|E|A|M\E\K(G)</td><td>649.8979</td><td>1298.641</td><td>0.1476</td></tr>
<tr><td></td><td></td><td></td><td></td><td></td><td></td><td>2</td><td>10.79</td><td>5.07</td><td>96.5</td><td>(K)L V/V E/T/T/A/N Q|D|P L V\T K(G)</td><td>814.3735</td><td>1627.89</td><td>-0.1504</td></tr>
<tr><td>2278</td><td>ABB02395.1</td><td>Temperature-induced lipocalin</td><td>2(7)</td><td>13%</td><td>31.21</td><td>2</td><td>17.52</td><td>7.88</td><td>91.1</td><td>(R)A T/Y/T/L|E/A|D\G|T/T V\R(V)</td><td>699.709</td><td>1397.691</td><td>0.72</td></tr>
<tr><td></td><td></td><td>(Vitis vinifera)</td><td></td><td></td><td></td><td>3</td><td>13.69</td><td>4.33</td><td>96.4</td><td>(R)V/L|N/E/T/W|S\D G K R(S)</td><td>435.682</td><td>1304.659</td><td>0.3721</td></tr>
<tr><td></td><td></td><td></td><td></td><td></td><td></td><td>2</td><td>13.36</td><td>3.76</td><td>94.6</td><td>(R)V/L/N|E/T/W/S/D/G K R(S)</td><td>653.488</td><td>1304.659</td><td>1.3094</td></tr>
<tr><td></td><td></td><td></td><td></td><td></td><td></td><td>2</td><td>13.25</td><td>2.26</td><td>84.2</td><td>(R)A T/Y/T/L|E/A|D\G T/T V R(V)</td><td>700.1365</td><td>1397.691</td><td>1.575</td></tr>
<tr><td></td><td></td><td></td><td></td><td></td><td></td><td>2</td><td>13.2</td><td>2.06</td><td>82.6</td><td>(R)A T/Y/T|L/E/A|D\G T T V R(V)</td><td>699.5597</td><td>1397.691</td><td>0.4214</td></tr>
<tr><td></td><td></td><td></td><td></td><td></td><td></td><td>2</td><td>10.94</td><td>2.05</td><td>87.6</td><td>(R)A T/Y/T/L|E/A/D\G T/T V R(V)</td><td>699.5742</td><td>1397.691</td><td>0.4504</td></tr>
<tr><td></td><td></td><td></td><td></td><td></td><td></td><td>3</td><td>10.04</td><td>2.54</td><td>96.8</td><td>(R)V/L|N/E|T W/S\D G K/R(S)</td><td>435.9149</td><td>1304.659</td><td>1.0708</td></tr>
<tr><td>2289</td><td>NP_001268052.1</td><td>Ripening-related protein grip22 precursor</td><td>1(4)</td><td>6%</td><td>14.26</td><td>2</td><td>14.26</td><td>8.57</td><td>80.7</td><td>(R)I V/A|L/S/T/G|W|Y|N G\G S\R(C)</td><td>741.4071</td><td>1480.754</td><td>1.0526</td></tr>
<tr><td></td><td></td><td>(Vitis vinifera)</td><td></td><td></td><td></td><td>2</td><td>13.7</td><td>3.59</td><td>78</td><td>(R)I V/A\L/S|T/G|W|Y|N G/G S\R(C)</td><td>740.7445</td><td>1480.754</td><td>-0.2726</td></tr>
<tr><td></td><td></td><td></td><td></td><td></td><td></td><td>2</td><td>13.29</td><td>4.41</td><td>73.9</td><td>(R)I V/A|L|S T/G/W|Y\N/G G S\R(C)</td><td>741.343</td><td>1480.754</td><td>0.9244</td></tr>
<tr><td></td><td></td><td></td><td></td><td></td><td></td><td>2</td><td>12.32</td><td>7.12</td><td>89.3</td><td>(R)I V/A\L|S/T/G/W|Y|N G G S\R(C)</td><td>741.281</td><td>1480.754</td><td>0.8004</td></tr>
<tr><td></td><td></td><td></td><td></td><td></td><td></td><td></td><td></td><td></td><td></td><td>MS/MS spectrum is reported at the end of the table</td><td></td><td></td><td></td></tr>
<tr><td>2342</td><td>XP_002281506.1</td><td>Class I heat shock protein</td><td>2(10)</td><td>18%</td><td>33.21</td><td>2</td><td>19.26</td><td>4.36</td><td>89.6</td><td>(K)A A/M/E/N|G/V|L|T|V|T/V|P K(A)</td><td>715.6631</td><td>1429.772</td><td>0.547</td></tr>
<tr><td></td><td></td><td>(Vitis vinifera)</td><td></td><td></td><td></td><td>2</td><td>17.06</td><td>6.93</td><td>91</td><td>(K)A A/M E/N|G/V|L|T|V/T\V|P K(A)</td><td>716.1011</td><td>1429.772</td><td>1.423</td></tr>
<tr><td></td><td></td><td></td><td></td><td></td><td></td><td>2</td><td>16.07</td><td>6.14</td><td>93.7</td><td>(K)A A/M/E/N/G/V|L|T|V/T\V|P K(A)</td><td>715.6948</td><td>1429.772</td><td>0.6104</td></tr>
<tr><td></td><td></td><td></td><td></td><td></td><td></td><td>2</td><td>15.44</td><td>5.92</td><td>83.9</td><td>(K)A A/M/E/N/G/V|L|T|V/T\V|P K(A)</td><td>715.9612</td><td>1429.772</td><td>1.1432</td></tr>
<tr><td></td><td></td><td></td><td></td><td></td><td></td><td>2</td><td>13.95</td><td>7.73</td><td>93.8</td><td>(R)F F|G N|P/S/V|S/D|P F P R(E)</td><td>733.9364</td><td>1466.706</td><td>0.1592</td></tr>
<tr><td></td><td></td><td></td><td></td><td></td><td></td><td>2</td><td>13.6</td><td>3.91</td><td>89.9</td><td>(R)F F|G N|P/S/V\S|D|P F P R(E)</td><td>734.1375</td><td>1466.706</td><td>0.5614</td></tr>
<tr><td></td><td></td><td></td><td></td><td></td><td></td><td>2</td><td>13.52</td><td>3.35</td><td>86.5</td><td>(K)A A/M E/N/G/V|L/T/V\T|V|P K(A)</td><td>715.1425</td><td>1429.772</td><td>-0.4942</td></tr>
<tr><td></td><td></td><td></td><td></td><td></td><td></td><td>2</td><td>12.91</td><td>2.8</td><td>83.7</td><td>(R)F F|G|N|P/S V|S/D|P F P R(E)</td><td>734.1631</td><td>1466.706</td><td>0.6126</td></tr>
<tr><td></td><td></td><td></td><td></td><td></td><td></td><td>2</td><td>12.29</td><td>6</td><td>80.8</td><td>(K)A/A/m/E/N/G/V|L|T/V T V\P K(A)</td><td>723.7595</td><td>1429.772</td><td>16.7398</td></tr>
<tr><td></td><td></td><td></td><td></td><td></td><td></td><td>2</td><td>10.73</td><td>2.37</td><td>95.5</td><td>(K)A A/M E/N|G/V/L/T|V T V\P K(A)</td><td>715.9231</td><td>1429.772</td><td>1.067</td></tr>
<tr><td>2344</td><td>XP_010657906.1</td><td>22.0 kDa class IV heat shock protein-like</td><td>5(10)</td><td>30%</td><td>76.21</td><td>3</td><td>21.18</td><td>9.56</td><td>97.4</td><td>(K)V/V/N\I/A|E|E|T N|S|G E/D|V m\A\T\K(S)</td><td>641.775</td><td>1906.906</td><td>16.4042</td></tr>
<tr><td></td><td></td><td>(Vitis vinifera)</td><td></td><td></td><td></td><td>2</td><td>20.5</td><td>14.22</td><td>100</td><td>(K)V V N I/A/E/E|T|N S|G E|D\V|m\A\T\K(S)</td><td>962.0211</td><td>1906.906</td><td>16.1287</td></tr>
<tr><td></td><td></td><td></td><td></td><td></td><td></td><td>2</td><td>19.54</td><td>19.54</td><td>95.6</td><td>(K)V V/N/I/A/E/E|T/N\S|G/E\D V|M\A\T K(S)</td><td>953.7321</td><td>1906.906</td><td>-0.4493</td></tr>
<tr><td></td><td></td><td></td><td></td><td></td><td></td><td>2</td><td>17.81</td><td>11.72</td><td>97.7</td><td>(K)V V N I/A/E/E|T|N S|G/E/D\V/m\A\T\K(S)</td><td>962.2721</td><td>1906.906</td><td>16.6307</td></tr>
<tr><td></td><td></td><td></td><td></td><td></td><td></td><td>2</td><td>17.1</td><td>5.64</td><td>97.2</td><td>(K)G L|E|T|I|A|L|A\R(S)</td><td>472.4836</td><td>943.557</td><td>0.4028</td></tr>
<tr><td></td><td></td><td></td><td></td><td></td><td></td><td>2</td><td>16.09</td><td>3.81</td><td>93.4</td><td>(R)I/L/E/H|S|P I\T V|P K(G)</td><td>618.0494</td><td>1233.72</td><td>1.3714</td></tr>
<tr><td></td><td></td><td></td><td></td><td></td><td></td><td>3</td><td>14.42</td><td>5.02</td><td>92.4</td><td>(R)I/L|E H/S|P\I/T V\P K(G)</td><td>412.159</td><td>1233.72</td><td>0.7423</td></tr>
<tr><td></td><td></td><td></td><td></td><td></td><td></td><td>2</td><td>14.15</td><td>10.17</td><td>95</td><td>(K)V V N I/A/E/E/T|N S|G E/D\V|m/A T K(S)</td><td>962.8232</td><td>1906.906</td><td>17.7329</td></tr>
<tr><td></td><td></td><td></td><td></td><td></td><td></td><td>2</td><td>11.5</td><td>3.52</td><td>100</td><td>(R)L/P|A|N/A/D|L D\R(I)</td><td>492.8099</td><td>984.511</td><td>0.1016</td></tr>
<tr><td></td><td></td><td></td><td></td><td></td><td></td><td>2</td><td>10.34</td><td>2.13</td><td>77.1</td><td>(K)A/H|L|E\N|G V\L\R(I)</td><td>504.4832</td><td>1008.559</td><td>-0.5994</td></tr>
<tr><td>2356</td><td>CAC16165.1</td><td>Pathogenesis-related protein 10</td><td>3(15)</td><td>23%</td><td>51.63</td><td>2</td><td>20.16</td><td>10.59</td><td>98.2</td><td>(K)A I/E/A/Y|V|L|A|H|P\D\A\Y(-)</td><td>717.0642</td><td>1432.711</td><td>0.4104</td></tr>
<tr><td></td><td></td><td>(Vitis vinifera)</td><td></td><td></td><td></td><td>2</td><td>19.2</td><td>6.16</td><td>97.9</td><td>(K)A/A/V\L|D|A|D|N|L|I|P\K(V)</td><td>620.6614</td><td>1239.694</td><td>0.6212</td></tr>
<tr><td></td><td></td><td></td><td></td><td></td><td></td><td>2</td><td>18.9</td><td>8.69</td><td>98.1</td><td>(K)A I/E/A/Y|V|L|A|H|P\D\A\Y(-)</td><td>717.0088</td><td>1432.711</td><td>0.2996</td></tr>
<tr><td></td><td></td><td></td><td></td><td></td><td></td><td>2</td><td>18.22</td><td>8.26</td><td>94.4</td><td>(K)A I/E/A|Y|V|L|A|H|P D A\Y(-)</td><td>717.7908</td><td>1432.711</td><td>1.8636</td></tr>
<tr><td></td><td></td><td></td><td></td><td></td><td></td><td>2</td><td>17.96</td><td>9.91</td><td>99</td><td>(K)A I E/A|Y|V|L|A|H|P\D\A\Y(-)</td><td>717.0801</td><td>1432.711</td><td>0.4422</td></tr>
<tr><td></td><td></td><td></td><td></td><td></td><td></td><td>2</td><td>17.93</td><td>5.51</td><td>97.1</td><td>(K)A/A/V\L|D|A|D|N|L|I|P\K(V)</td><td>620.3845</td><td>1239.694</td><td>0.0674</td></tr>
<tr><td></td><td></td><td></td><td></td><td></td><td></td><td>2</td><td>16.64</td><td>3.57</td><td>95.1</td><td>(K)A/A/V\L|D|A|D|N|L|I|P\K(V)</td><td>621.0363</td><td>1239.694</td><td>1.371</td></tr>
<tr><td></td><td></td><td></td><td></td><td></td><td></td><td>2</td><td>15.93</td><td>9.15</td><td>93.3</td><td>(K)A I E/A/Y|V|L|A|H|P D A\Y(-)</td><td>717.0872</td><td>1432.711</td><td>0.4564</td></tr>
<tr><td></td><td></td><td></td><td></td><td></td><td></td><td>2</td><td>15.86</td><td>7.48</td><td>96.7</td><td>(K)A I E/A/Y|V|L|A|H|P D\A\Y(-)</td><td>717.0181</td><td>1432.711</td><td>0.3182</td></tr>
<tr><td></td><td></td><td></td><td></td><td></td><td></td><td>2</td><td>15.63</td><td>3.59</td><td>94.6</td><td>(K)A/A/V\L|D|A|D|N|L|I|P K(V)</td><td>620.5541</td><td>1239.694</td><td>0.4066</td></tr>
<tr><td></td><td></td><td></td><td></td><td></td><td></td><td>2</td><td>15.1</td><td>6.79</td><td>93.3</td><td>(K)A I/E/A/Y|V|L|A|H|P D A\Y(-)</td><td>717.3667</td><td>1432.711</td><td>1.0154</td></tr>
<tr><td></td><td></td><td></td><td></td><td></td><td></td><td>2</td><td>14.57</td><td>5.65</td><td>92.3</td><td>(K)A I E A Y/V|L|A|H|P D A\Y(-)</td><td>717.1072</td><td>1432.711</td><td>0.4964</td></tr>
<tr><td></td><td></td><td></td><td></td><td></td><td></td><td>2</td><td>12.27</td><td>2.4</td><td>82.5</td><td>(K)G G/K/E|D/A L|A|T\F\K(A)</td><td>569.3398</td><td>1136.595</td><td>1.0777</td></tr>
<tr><td></td><td></td><td></td><td></td><td></td><td></td><td>2</td><td>11.02</td><td>3.85</td><td>90.4</td><td>(K)G/G/K/E|D|A L|A\T\F K(A)</td><td>568.9817</td><td>1136.595</td><td>0.3615</td></tr>
<tr><td></td><td></td><td></td><td></td><td></td><td></td><td>2</td><td>10.9</td><td>2.64</td><td>95.8</td><td>(K)A/A/V L|D/A/D/N\L|I|P K(V)</td><td>620.7905</td><td>1239.694</td><td>0.8794</td></tr>
<tr><td>2381</td><td>CAC16165.1</td><td>Pathogenesis-related protein 10</td><td>3(5)</td><td>23%</td><td>45.73</td><td>2</td><td>19.5</td><td>7.8</td><td>100</td><td>(K)G/G/K/E|D|A/L|A|T\F\K(A)</td><td>568.8792</td><td>1136.595</td><td>0.1565</td></tr>
<tr><td></td><td></td><td>(Vitis vinifera)</td><td></td><td></td><td></td><td>2</td><td>19.39</td><td>6.97</td><td>97.7</td><td>(K)G/G/K/E|D|A|L|A|T|F\K(A)</td><td>569.0071</td><td>1136.595</td><td>0.4123</td></tr>
<tr><td></td><td></td><td></td><td></td><td></td><td></td><td>2</td><td>15.44</td><td>9.9</td><td>98.8</td><td>(K)A I/E/A/Y|V|L|A|H|P D A\Y(-)</td><td>717.0344</td><td>1432.711</td><td>0.3508</td></tr>
<tr><td></td><td></td><td></td><td></td><td></td><td></td><td>1</td><td>11.98</td><td>5.74</td><td>88.8</td><td>(K)A I E/A Y V|L|A|H\P D\A\Y(-)</td><td>1432.3728</td><td>1432.711</td><td>-0.3379</td></tr>
<tr><td></td><td></td><td></td><td></td><td></td><td></td><td>2</td><td>10.79</td><td>2.68</td><td>91.2</td><td>(K)A A/V L|D|A|D N|L\I|P K(V)</td><td>620.6104</td><td>1239.694</td><td>0.5192</td></tr>
</table>

<table>
<tr><td>2382</td><td>S52629</td><td>**Catechol oxidase (EC 1.10.3.1) precursor - grape**</td><td>4(17)</td><td>9%</td><td>69.12</td><td>2</td><td>21</td><td>10.86</td><td>96</td><td>(K)F D/V|Y/I|N|D|E D|Y|S/V/S R\P K(N)</td><td>974.1334</td><td>1946.913</td><td>0.3465</td></tr>
<tr><td></td><td></td><td>*(Vitis vinifera)*</td><td></td><td></td><td></td><td>2</td><td>18.38</td><td>10.58</td><td>93.3</td><td>(K)F D/V/Y/I|N/D/E D|Y|S/V/S R\P K(N)</td><td>974.2618</td><td>1946.913</td><td>0.6033</td></tr>
<tr><td></td><td></td><td></td><td></td><td></td><td></td><td>2</td><td>16.95</td><td>9.43</td><td>92.7</td><td>(R)N/S/E/F/A/G/S|F|V|N/V|P H\K(H)</td><td>767.0842</td><td>1532.749</td><td>0.4119</td></tr>
<tr><td></td><td></td><td></td><td></td><td></td><td></td><td>2</td><td>16.35</td><td>6.2</td><td>85.7</td><td>(K)T T|I/S/S/I|G/D|F|P K(A)</td><td>583.2852</td><td>1165.61</td><td>-0.0468</td></tr>
<tr><td></td><td></td><td></td><td></td><td></td><td></td><td>3</td><td>16.26</td><td>6.59</td><td>88.1</td><td>(R)N/S|E|F/A/G S|F|V|N|V|P H\K(H)</td><td>511.5488</td><td>1532.749</td><td>-0.1174</td></tr>
<tr><td></td><td></td><td></td><td></td><td></td><td></td><td>3</td><td>16.25</td><td>3.33</td><td>87.2</td><td>(R)N/S|E|F|A/G S\F/V|N V/P H\K(H)</td><td>511.7787</td><td>1532.749</td><td>0.5723</td></tr>
<tr><td></td><td></td><td></td><td></td><td></td><td></td><td>2</td><td>15.39</td><td>9.36</td><td>87.7</td><td>(K)F D/V|Y/I/N/D/E D|Y/S V/S K P R(N)</td><td>974.2008</td><td>1946.913</td><td>0.4813</td></tr>
<tr><td></td><td></td><td></td><td></td><td></td><td></td><td>3</td><td>15.08</td><td>3.88</td><td>94.7</td><td>(K)F/D|V|Y|I/N/D/E D/Y/S V S K P R(N)</td><td>649.9526</td><td>1946.913</td><td>0.9302</td></tr>
<tr><td></td><td></td><td></td><td></td><td></td><td></td><td>2</td><td>14.82</td><td>5.11</td><td>85.8</td><td>(K)G I/E/L/D|R E|N\F\V\K(F)</td><td>660.519</td><td>1319.695</td><td>0.3353</td></tr>
<tr><td></td><td></td><td></td><td></td><td></td><td></td><td>2</td><td>14.77</td><td>8.23</td><td>82.4</td><td>(R)N S/E|F|A/G/S|F|V/N/V|P/H\K(H)</td><td>766.8345</td><td>1532.749</td><td>-0.0875</td></tr>
<tr><td></td><td></td><td></td><td></td><td></td><td></td><td>3</td><td>13.92</td><td>3.82</td><td>86.7</td><td>(R)N/S|E|F/A/G S\F/V/N V/P H\K(H)</td><td>511.9884</td><td>1532.749</td><td>1.2014</td></tr>
<tr><td></td><td></td><td></td><td></td><td></td><td></td><td>2</td><td>13.72</td><td>3.59</td><td>79.9</td><td>(K)T T|I/S|S/I|G/D/F\P K(A)</td><td>583.4517</td><td>1165.61</td><td>0.2862</td></tr>
<tr><td></td><td></td><td></td><td></td><td></td><td></td><td>2</td><td>13.58</td><td>5.01</td><td>80.6</td><td>(K)T T|I/S|S/I|G D|F\P K(A)</td><td>583.376</td><td>1165.61</td><td>0.1348</td></tr>
<tr><td></td><td></td><td></td><td></td><td></td><td></td><td>2</td><td>13.33</td><td>3.13</td><td>78.4</td><td>(K)T T|I/S|S/I|G D|F\P K(A)</td><td>583.5</td><td>1165.61</td><td>0.3828</td></tr>
<tr><td></td><td></td><td></td><td></td><td></td><td></td><td>3</td><td>13.3</td><td>3.71</td><td>86.5</td><td>(R)N/S|E|F/A/G S/F\V/N\V|P H K(H)</td><td>511.8395</td><td>1532.749</td><td>0.7547</td></tr>
<tr><td></td><td></td><td></td><td></td><td></td><td></td><td>2</td><td>12.36</td><td>6.04</td><td>89.3</td><td>(K)F D/V/Y/I/N/D/E D/Y/S/V/S R\P K(N)</td><td>974.411</td><td>1946.913</td><td>0.9017</td></tr>
<tr><td></td><td></td><td></td><td></td><td></td><td></td><td>2</td><td>12</td><td>6.92</td><td>100</td><td>(K)F D/V/Y/I/N D|E/D|Y/S V S R\P K(N)</td><td>974.8275</td><td>1946.913</td><td>1.7347</td></tr>
<tr><td>2386</td><td>AAB41022.1</td><td>**Polyphenol oxidase**</td><td>3(3)</td><td>7%</td><td>38.75</td><td>2</td><td>14.29</td><td>5.65</td><td>82.4</td><td>(K)T/T|I/S|S/I|G D|F\P K(A)</td><td>583.4603</td><td>1165.61</td><td>0.3034</td></tr>
<tr><td></td><td></td><td>*(Vitis vinifera)*</td><td></td><td></td><td></td><td>3</td><td>14.19</td><td>4.57</td><td>82.9</td><td>(R)N/S|E|F|A/G S|F|V/N V/P H\K(H)</td><td>511.9826</td><td>1532.749</td><td>1.184</td></tr>
<tr><td></td><td></td><td></td><td></td><td></td><td></td><td>2</td><td>10.27</td><td>3.6</td><td>87.6</td><td>(K)F D/V/Y/I/N/D/E D/Y S V S R P K(N)</td><td>974.4591</td><td>1946.913</td><td>0.9979</td></tr>
<tr><td>2415</td><td>XP_002281285.1</td><td>**18.2 kDa class I heat shock protein**</td><td>1(3)</td><td>9%</td><td>19.36</td><td>2</td><td>19.36</td><td>8.85</td><td>91.4</td><td>(K)A A/m/E/N/G/V|L|T|V|T V|P K(A)</td><td>723.9524</td><td>1429.772</td><td>17.1256</td></tr>
<tr><td></td><td></td><td>*(Vitis vinifera)*</td><td></td><td></td><td></td><td>2</td><td>18.51</td><td>7.09</td><td>92.1</td><td>(K)A A/M/E/N/G/V|L|T|V/T\V|P K(A)</td><td>715.6863</td><td>1429.772</td><td>0.5934</td></tr>
<tr><td></td><td></td><td></td><td></td><td></td><td></td><td>3</td><td>12.37</td><td>2.57</td><td>97.7</td><td>(K)A A/M/E N G\V|L/T|V/T V P K(A)</td><td>477.5819</td><td>1429.772</td><td>0.9592</td></tr>
<tr><td></td><td></td><td></td><td></td><td></td><td></td><td></td><td></td><td></td><td></td><td>**MS/MS spectrum is reported at the end of the table**</td><td></td><td></td><td></td></tr>
<tr><td>2446</td><td>XP_003631809.1</td><td>**17.3 kDa class II heat shock protein-like**</td><td>3(3)</td><td>27%</td><td>51.82</td><td>2</td><td>20.9</td><td>13.76</td><td>98.8</td><td>(K)V Q/V|E/D/D|N/V|L|V|I|S\G E R(K)</td><td>836.6604</td><td>1671.855</td><td>0.4587</td></tr>
<tr><td></td><td></td><td>*(Vitis vinifera)*</td><td></td><td></td><td></td><td>2</td><td>16.66</td><td>6.71</td><td>91.5</td><td>(K)I S/A/V/C/Q/D|G V|L|T/V/T\V E K(L)</td><td>860.8041</td><td>1718.899</td><td>1.7016</td></tr>
<tr><td></td><td></td><td></td><td></td><td></td><td></td><td>3</td><td>14.26</td><td>3.07</td><td>85.2</td><td>(R)K F V|L\P\E|N|A|N/T D K(I)</td><td>459.5319</td><td>1375.722</td><td>0.8595</td></tr>
<tr><td>2517</td><td></td><td>**Nucleoside diphosphate kinase B**</td><td>4(19)</td><td>32%</td><td>60.3</td><td>2</td><td>19.09</td><td>6.23</td><td>96.3</td><td>(K)I/I/G A/T/N|P/S/D|S|A|P G T I\R(G)</td><td>785.9395</td><td>1569.823</td><td>1.0486</td></tr>
<tr><td></td><td></td><td>*(Vitis vinifera)*</td><td></td><td></td><td></td><td>2</td><td>17.7</td><td>7.81</td><td>92.5</td><td>(K)I/I/G A|T/N|P S D|S|A|P G T I R(G)</td><td>785.3965</td><td>1569.823</td><td>-0.0374</td></tr>
<tr><td></td><td></td><td></td><td></td><td></td><td></td><td>2</td><td>15.94</td><td>7.68</td><td>92.7</td><td>(R)N V|I|H|G|S\D|S V/G S A R(K)</td><td>650.0623</td><td>1298.645</td><td>0.4726</td></tr>
<tr><td></td><td></td><td></td><td></td><td></td><td></td><td>3</td><td>15.62</td><td>4.62</td><td>89.4</td><td>(K)I I/G A T\N\P S|D S|A|P/G T\I R(G)</td><td>524.2396</td><td>1569.823</td><td>0.8811</td></tr>
<tr><td></td><td></td><td></td><td></td><td></td><td></td><td>2</td><td>15.16</td><td>4.1</td><td>90.8</td><td>(K)I I/G/A/T/N|P S D|S|A|P G T I R(G)</td><td>785.8285</td><td>1569.823</td><td>0.8266</td></tr>
<tr><td></td><td></td><td></td><td></td><td></td><td></td><td>2</td><td>14.87</td><td>4.53</td><td>89.2</td><td>(K)I I/G A/T/N|P/S D|S/A|P G T I R(G)</td><td>785.3572</td><td>1569.823</td><td>-0.116</td></tr>
<tr><td></td><td></td><td></td><td></td><td></td><td></td><td>2</td><td>12.72</td><td>3.11</td><td>97.5</td><td>(R)G D/F|A|V|D|I|G\R(N)</td><td>475.3354</td><td>949.474</td><td>0.1898</td></tr>
<tr><td></td><td></td><td></td><td></td><td></td><td></td><td>2</td><td>12.7</td><td>3.87</td><td>92.7</td><td>(K)I I/G A/T/N|P S D/S/A|P G T I R(G)</td><td>785.9451</td><td>1569.823</td><td>1.0598</td></tr>
<tr><td></td><td></td><td></td><td></td><td></td><td></td><td>2</td><td>12.55</td><td>5.18</td><td>95.2</td><td>(R)N L|V|G/E|I|I|G R(F)</td><td>485.8397</td><td>970.568</td><td>0.1041</td></tr>
<tr><td></td><td></td><td></td><td></td><td></td><td></td><td>2</td><td>12.25</td><td>3.89</td><td>100</td><td>(R)G D/F|A|V|D|I|G R(N)</td><td>475.2458</td><td>949.474</td><td>0.0106</td></tr>
<tr><td></td><td></td><td></td><td></td><td></td><td></td><td>2</td><td>12.06</td><td>5.13</td><td>95.4</td><td>(R)N L|V|G\E/I|I|G\R(F)</td><td>485.6113</td><td>970.568</td><td>-0.3527</td></tr>
<tr><td></td><td></td><td></td><td></td><td></td><td></td><td>2</td><td>11.82</td><td>3.68</td><td>90.1</td><td>(R)N L|V|G/E|I|I|G R(F)</td><td>486.1157</td><td>970.568</td><td>0.6561</td></tr>
<tr><td></td><td></td><td></td><td></td><td></td><td></td><td>2</td><td>11.54</td><td>5.09</td><td>85.6</td><td>(R)N L|V|G|E/I|I|G R(F)</td><td>485.6241</td><td>970.568</td><td>-0.3271</td></tr>
<tr><td></td><td></td><td></td><td></td><td></td><td></td><td>2</td><td>11.21</td><td>4.33</td><td>99</td><td>(R)N L|V|G E|I|I|G R(F)</td><td>485.7473</td><td>970.568</td><td>-0.0807</td></tr>
<tr><td></td><td></td><td></td><td></td><td></td><td></td><td>2</td><td>11.16</td><td>2.59</td><td>92.2</td><td>(K)I I/G A/T/N|P S/D/S A|P G T I R(G)</td><td>785.5286</td><td>1569.823</td><td>0.2268</td></tr>
<tr><td></td><td></td><td></td><td></td><td></td><td></td><td>2</td><td>11.14</td><td>3.25</td><td>89.6</td><td>(K)I I/G A/T/N/P/S D/S/A|P G T I R(G)</td><td>785.5071</td><td>1569.823</td><td>0.1838</td></tr>
<tr><td></td><td></td><td></td><td></td><td></td><td></td><td>3</td><td>11.11</td><td>2.02</td><td>83.1</td><td>(R)N V|I/H G/S D S\V|G|S A R(K)</td><td>433.673</td><td>1298.645</td><td>0.3597</td></tr>
<tr><td></td><td></td><td></td><td></td><td></td><td></td><td>2</td><td>10.92</td><td>4.3</td><td>98</td><td>(R)N V/I|H|G|S D|S V G S A R(K)</td><td>649.7002</td><td>1298.645</td><td>-0.2516</td></tr>
<tr><td></td><td></td><td></td><td></td><td></td><td></td><td>2</td><td>10.65</td><td>4.43</td><td>97.7</td><td>(R)N L|V|G E|I|I|G R(F)</td><td>485.5988</td><td>970.568</td><td>-0.3777</td></tr>
<tr><td>2540</td><td>ADG35965.1</td><td>**Pathogenesis-related protein 4**</td><td>1(1)</td><td>14%</td><td>16.25</td><td>2</td><td>16.25</td><td>9.94</td><td>95.7</td><td>(K)Y G W/T/A/F|C/G/P S/G/P T|G Q\A|A\C G K(C)</td><td>1037.2209</td><td>2072.895</td><td>0.5392</td></tr>
<tr><td></td><td></td><td>*(Vitis hybrid cultivar)*</td><td></td><td></td><td></td><td></td><td></td><td></td><td></td><td>**MS/MS spectrum is reported at the end of the table**</td><td></td><td></td><td></td></tr>
<tr><td>2584</td><td>ADG35965.1</td><td>**Pathogenesis-related protein 4**</td><td>2(7)</td><td>24%</td><td>33.43</td><td>2</td><td>21.23</td><td>16.06</td><td>93</td><td>(R)I V/D/Q C|S/N/G|G/L|D|L|D|S G|V|F N K(L)</td><td>1019.9791</td><td>2037.955</td><td>0.9963</td></tr>
<tr><td></td><td></td><td>*(Vitis hybrid cultivar)*</td><td></td><td></td><td></td><td>2</td><td>19.71</td><td>14.78</td><td>93</td><td>(R)I V/D Q/C|S/N/G/G/L|D|L/D|S\G\V|F\N K(L)</td><td>1019.6119</td><td>2037.955</td><td>0.2619</td></tr>
<tr><td></td><td></td><td></td><td></td><td></td><td></td><td>2</td><td>18.27</td><td>7.95</td><td>91.9</td><td>(R)I V/D Q/C/S/N/G/G/L/D/L|D|S/G\V|F\N K(L)</td><td>1020.1797</td><td>2037.955</td><td>1.3975</td></tr>
<tr><td></td><td></td><td></td><td></td><td></td><td></td><td>2</td><td>15.27</td><td>10.58</td><td>96.8</td><td>(R)I V/D/Q C/S/N/G/G L/D/L/D|S\G\V/F\N\K(L)</td><td>1019.9254</td><td>2037.955</td><td>0.8889</td></tr>
<tr><td></td><td></td><td></td><td></td><td></td><td></td><td>2</td><td>12.85</td><td>6.34</td><td>86</td><td>(R)I/V D Q/C/S/N/G G L/D\L|D/S/G\V F N\K(L)</td><td>1019.7406</td><td>2037.955</td><td>0.5193</td></tr>
<tr><td></td><td></td><td></td><td></td><td></td><td></td><td>2</td><td>12.2</td><td>4.87</td><td>96.1</td><td>(K)C L/S V/T/N|T/A T/G|T/Q\A T\V R(I)</td><td>841.0029</td><td>1679.838</td><td>1.1604</td></tr>
<tr><td></td><td></td><td></td><td></td><td></td><td></td><td>2</td><td>10.48</td><td>5.5</td><td>76.7</td><td>(R)I V D Q C/S/N/G/G/L/D|L/D|S G\V F N K(L)</td><td>1020.4448</td><td>2037.955</td><td>1.9277</td></tr>
<tr><td>2618</td><td>CAI56334.1</td><td>**TPA: isoflavone reductase-like protein 5**</td><td>7(18)</td><td>29%</td><td>118.94</td><td>2</td><td>20.28</td><td>8.79</td><td>93.8</td><td>(K)S S G V/T/L|V/Y|G/D|L|Y/D|H E S L\V K(A)</td><td>1042.0425</td><td>2082.039</td><td>1.0387</td></tr>
<tr><td></td><td></td><td>*(Vitis vinifera)*</td><td></td><td></td><td></td><td>2</td><td>20.07</td><td>11.78</td><td>93.3</td><td>(K)Q V/D V V/I/S/T/V/G/H|A/Q L|P D|Q\V K(I)</td><td>1016.8196</td><td>2033.103</td><td>-0.4707</td></tr>
<tr><td></td><td></td><td></td><td></td><td></td><td></td><td>2</td><td>19.38</td><td>7.93</td><td>97.1</td><td>(K)I/L|I|I|G|G/T|G/Y\I|G K(F)</td><td>602.9048</td><td>1204.73</td><td>0.0723</td></tr>
<tr><td></td><td></td><td></td><td></td><td></td><td></td><td>2</td><td>18.63</td><td>8.13</td><td>90.2</td><td>(K)S S G V/T/L/V|Y/G/D/L|Y/D/H E\S L\V\K(A)</td><td>1041.9529</td><td>2082.039</td><td>0.8595</td></tr>
<tr><td></td><td></td><td></td><td></td><td></td><td></td><td>2</td><td>18.54</td><td>9.94</td><td>90.2</td><td>(K)Q V/D/V/V/I/S/T/V/G/H|A Q|L|P D Q V\K(I)</td><td>1017.3356</td><td>2033.103</td><td>0.5613</td></tr>
<tr><td></td><td></td><td></td><td></td><td></td><td></td><td>2</td><td>17.64</td><td>9.64</td><td>95.5</td><td>(R)D/K|V|I|I|L|G|D|G\N|P K(A)</td><td>634.9216</td><td>1268.721</td><td>0.115</td></tr>
<tr><td></td><td></td><td></td><td></td><td></td><td></td><td>2</td><td>17.23</td><td>9.65</td><td>96.1</td><td>(K)Q V D/V V|I/S/T/V/G/H/A Q/L|P D\Q V K(I)</td><td>1017.7416</td><td>2033.103</td><td>1.3733</td></tr>
<tr><td></td><td></td><td></td><td></td><td></td><td></td><td>2</td><td>17.13</td><td>7.84</td><td>90.6</td><td>(K)S S G V/T/L/V/Y|G/D|L|Y/D/H E\S L\V K(A)</td><td>1041.5645</td><td>2082.039</td><td>0.0827</td></tr>
<tr><td></td><td></td><td></td><td></td><td></td><td></td><td>3</td><td>16.68</td><td>5.06</td><td>91</td><td>(K)A V|F/N K E D D|I|G|T\Y T I\K(A)</td><td>572.0234</td><td>1713.869</td><td>0.1863</td></tr>
<tr><td></td><td></td><td></td><td></td><td></td><td></td><td>3</td><td>16.48</td><td>4.93</td><td>93.5</td><td>(K)S/S/G/V|T|L/V/Y/G\D|L/Y/D/H E S L V K(A)</td><td>695.0421</td><td>2082.039</td><td>1.0728</td></tr>
<tr><td></td><td></td><td></td><td></td><td></td><td></td><td>2</td><td>14.46</td><td>5.27</td><td>93.4</td><td>(R)F/F|P/S/E|F|G\N/D V\D\R(V)</td><td>715.5032</td><td>1429.638</td><td>0.3609</td></tr>
<tr><td></td><td></td><td></td><td></td><td></td><td></td><td>2</td><td>14.36</td><td>5.82</td><td>85.5</td><td>(K)S S/G V/T/L/V/Y/G/D/L/Y/D/H E S L V\K(A)</td><td>1041.7544</td><td>2082.039</td><td>0.4625</td></tr>
</table>

<table>
<tr><th></th><th></th><th></th><th></th><th></th><th></th><th></th><th></th><th></th><th></th><th></th><th></th><th></th><th></th></tr>
<tr><td></td><td></td><td></td><td></td><td></td><td></td><td>2</td><td>13.41</td><td>4.23</td><td>95.4</td><td>(R)F/F|P/S/E|F|G/N D\V\D R(V)</td><td>715.8508</td><td>1429.638</td><td>1.0561</td></tr>
<tr><td></td><td></td><td></td><td></td><td></td><td></td><td>2</td><td>13.26</td><td>3.27</td><td>93.2</td><td>(K)I L|I|I|G|G T G/Y\I\G K(F)</td><td>603.1582</td><td>1204.73</td><td>0.5791</td></tr>
<tr><td></td><td></td><td></td><td></td><td></td><td></td><td>2</td><td>13.07</td><td>5.46</td><td>86.4</td><td>(K)A V/F N|K/E|D|D|I|G T Y T I K(A)</td><td>857.7158</td><td>1713.869</td><td>0.5549</td></tr>
<tr><td></td><td></td><td></td><td></td><td></td><td></td><td>2</td><td>11.69</td><td>5.72</td><td>93.2</td><td>(K)S S/G V/T L/V/Y/G/D/L/Y/D H E S L V\K(A)</td><td>1041.8125</td><td>2082.039</td><td>0.5787</td></tr>
<tr><td></td><td></td><td></td><td></td><td></td><td></td><td>2</td><td>10.84</td><td>3.62</td><td>92.1</td><td>(R)F/F/P/S/E|F|G N D\V D R(V)</td><td>715.9869</td><td>1429.638</td><td>1.3283</td></tr>
<tr><td></td><td></td><td></td><td></td><td></td><td></td><td>2</td><td>10.43</td><td>2.54</td><td>98.4</td><td>(K)V I|I|L|G/D G N|P K(A)</td><td>513.5034</td><td>1025.599</td><td>0.4006</td></tr>
<tr><td>2619</td><td>XP_002267091.2</td><td>Enolase</td><td>3(3)</td><td>28%</td><td>50.16</td><td>2</td><td>21</td><td>11.56</td><td>100</td><td>(K)V Q|I|V|G/D/D/L|L|V|T|N\P K(R)</td><td>756.2333</td><td>1510.848</td><td>0.6118</td></tr>
<tr><td></td><td></td><td>(Vitis vinifera)</td><td></td><td></td><td></td><td>2</td><td>17.45</td><td>8.98</td><td>97.4</td><td>(R)A A/V/P/S/G A/S/T|G I|Y|E\A\L|E L R(D)</td><td>903.4209</td><td>1804.944</td><td>0.8906</td></tr>
<tr><td></td><td></td><td></td><td></td><td></td><td></td><td>3</td><td>11.71</td><td>4.39</td><td>98.1</td><td>(K)T/Y|D|L/N/F/K/E/E/N/N D G S Q K(I)</td><td>634.928</td><td>1901.851</td><td>0.9183</td></tr>
<tr><td>2626</td><td>AAB65776.1</td><td>Class IV endochitinase</td><td>4(6)</td><td>32%</td><td>77.44</td><td>3</td><td>22.87</td><td>13.59</td><td>97.7</td><td>(R)G P L Q L T/W|N/Y/N|Y G A|A|G N|S I|G F N G L/S/N/P G I/V/A/T|D V V\T S F K(T)</td><td>1315.1384</td><td>3942.966</td><td>0.4346</td></tr>
<tr><td></td><td></td><td>(Vitis vinifera)</td><td></td><td></td><td></td><td>2</td><td>20.61</td><td>14.18</td><td>98.5</td><td>(R)A A F L/S/A|L/N/S/Y/S|G|F|G N\D\G S\T D A N K(R)</td><td>1154.2378</td><td>2307.052</td><td>0.4159</td></tr>
<tr><td></td><td></td><td></td><td></td><td></td><td></td><td>2</td><td>20.27</td><td>15.87</td><td>96.9</td><td>(R)A A F L S/A|L|N/S|Y/S|G|F|G/N|D|G S T D A N K(R)</td><td>1154.3062</td><td>2307.052</td><td>0.5527</td></tr>
<tr><td></td><td></td><td></td><td></td><td></td><td></td><td>2</td><td>18.66</td><td>7.82</td><td>98.3</td><td>(R)A A/F L S A L/N/S/Y/S|G/F/G N D|G/S T D|A N K R(E)</td><td>1232.1299</td><td>2463.153</td><td>0.099</td></tr>
<tr><td></td><td></td><td></td><td></td><td></td><td></td><td>3</td><td>15.3</td><td>3.65</td><td>96.4</td><td>(K)T A L/W/F/W/m|N\N/V H S V\I/G\Q/G F G\A T I R(A)</td><td>874.8787</td><td>2605.313</td><td>17.3083</td></tr>
<tr><td></td><td></td><td></td><td></td><td></td><td></td><td>3</td><td>13.1</td><td>6.46</td><td>75.4</td><td>(R)G P L Q L T W N/Y N|Y G A A|G N S\I/G F N G/L/S N/P G I/V|A/T D V\V\T S F K(T)</td><td>1315.3215</td><td>3942.966</td><td>0.9839</td></tr>
<tr><td>2627</td><td>XP_002270168.1</td><td>V-type proton ATPase subunit E1</td><td>1(1)</td><td>6%</td><td>15.88</td><td>2</td><td>15.88</td><td>11.75</td><td>97.7</td><td>(K)V/L/Q/A/Q|D|D\L/V|N\S M K(E)</td><td>730.9707</td><td>1460.741</td><td>0.1928</td></tr>
<tr><td></td><td></td><td>(Vitis vinifera)</td><td></td><td></td><td></td><td></td><td></td><td></td><td></td><td>MS/MS spectrum is reported at the end of the table</td><td></td><td></td><td></td></tr>
<tr><td>2637</td><td>NP_001268052.1</td><td>Ripening-related protein grip22 precursor</td><td>2(4)</td><td>17%</td><td>34.58</td><td>2</td><td>18.83</td><td>9.68</td><td>89.8</td><td>(R)I V/A\L|S/T/G|W|Y|N/G\G S\R(C)</td><td>741.6205</td><td>1480.754</td><td>1.4794</td></tr>
<tr><td></td><td></td><td>(Vitis vinifera)</td><td></td><td></td><td></td><td>2</td><td>16.71</td><td>6.72</td><td>88.3</td><td>(R)I V/A|L|S/T|G/W|Y|N/G G S\R(C)</td><td>741.7219</td><td>1480.754</td><td>1.6822</td></tr>
<tr><td></td><td></td><td></td><td></td><td></td><td></td><td>2</td><td>15.75</td><td>7.22</td><td>74.8</td><td>(K)N T Y T C S P P I|T/S/S/T|P A/V|L|T N N N\F E K(G)</td><td>1328.9834</td><td>2656.256</td><td>0.7036</td></tr>
<tr><td></td><td></td><td></td><td></td><td></td><td></td><td>2</td><td>14.64</td><td>7.64</td><td>70.3</td><td>(K)N T/Y/T/C S P P I/T|S|S/T|P A/V/L|T N N N F E K(G)</td><td>1328.7178</td><td>2656.256</td><td>0.1724</td></tr>
<tr><td>2658</td><td>AAZ93634.1</td><td>MSA</td><td>3(15)</td><td>31%</td><td>69.78</td><td>2</td><td>25</td><td>20.18</td><td>100</td><td>(K)H/L/E/H/L|G E|L|G\V/A|A|A|G/A\Y\A\L\H\E\K(H)</td><td>1094.0049</td><td>2186.135</td><td>0.8672</td></tr>
<tr><td></td><td></td><td>(Vitis vinifera)</td><td></td><td></td><td></td><td>2</td><td>24.5</td><td>18.44</td><td>100</td><td>(K)H L/E/H/L|G E|L|G V/A|A|A|G A|Y\A\L\H\E\K(H)</td><td>1093.3748</td><td>2186.135</td><td>-0.393</td></tr>
<tr><td></td><td></td><td></td><td></td><td></td><td></td><td>3</td><td>23.85</td><td>13.02</td><td>97.8</td><td>(K)H L/E/H L|G E\L/G\V|A|A|A|G|A/Y|A|L\H\E\K(H)</td><td>729.7427</td><td>2186.135</td><td>1.0783</td></tr>
<tr><td></td><td></td><td></td><td></td><td></td><td></td><td>3</td><td>23.41</td><td>14.09</td><td>97.6</td><td>(K)H/K/I/E/E/E|I|A\A/A A A\V/G A/G G/F/A F/H|E\H\H\E\K(K)</td><td>919.7294</td><td>2756.354</td><td>0.8197</td></tr>
<tr><td></td><td></td><td></td><td></td><td></td><td></td><td>3</td><td>22.39</td><td>10.64</td><td>96.5</td><td>(K)H/K/I/E/E/E I/A A/A A\A V|G A/G/G/F|A/F/H E\H\H\E\K(K)</td><td>919.694</td><td>2756.354</td><td>0.7135</td></tr>
<tr><td></td><td></td><td></td><td></td><td></td><td></td><td>2</td><td>21.57</td><td>15.27</td><td>94.5</td><td>(K)H K/I E E E I|A|A|A|A|A|V|G A|G\G F A F H E H H\E\K(K)</td><td>1379.2881</td><td>2756.354</td><td>1.215</td></tr>
<tr><td></td><td></td><td></td><td></td><td></td><td></td><td>3</td><td>21.37</td><td>10.37</td><td>97.7</td><td>(K)I/E/E/E|I|A/A|A A/A|V|G|A/G G F A F H E H H E K(K)</td><td>830.9534</td><td>2491.2</td><td>-0.3544</td></tr>
<tr><td></td><td></td><td></td><td></td><td></td><td></td><td>3</td><td>21.2</td><td>10.93</td><td>96.1</td><td>(K)H L/E H L/G E L G\V|A|A|A|G|A/Y\A|L|H\E\K(H)</td><td>729.6317</td><td>2186.135</td><td>0.7453</td></tr>
<tr><td></td><td></td><td></td><td></td><td></td><td></td><td>3</td><td>21.19</td><td>10.45</td><td>95</td><td>(K)H L/E/H L G E L\G|V|A|A|A|G|A/Y|A\L\H\E K(H)</td><td>729.8677</td><td>2186.135</td><td>1.4533</td></tr>
<tr><td></td><td></td><td></td><td></td><td></td><td></td><td>2</td><td>20.25</td><td>9.98</td><td>94.9</td><td>(K)I E E E I/A/A/A|A|A|V/G/A/G G/F/A/F/H E\H H\E\K(K)</td><td>1246.8684</td><td>2491.2</td><td>1.5295</td></tr>
<tr><td></td><td></td><td></td><td></td><td></td><td></td><td>2</td><td>16.74</td><td>8.56</td><td>86.2</td><td>(K)I E E E/I A A|A/A|A|V/G/A/G G/F A/F H\E/H H\E\K(K)</td><td>1246.2031</td><td>2491.2</td><td>0.1989</td></tr>
<tr><td></td><td></td><td></td><td></td><td></td><td></td><td>3</td><td>15.14</td><td>5.89</td><td>92.5</td><td>(K)H L/E H L G E L G\V|A|A|A|G|A/Y/A\L\H\E K(H)</td><td>729.8275</td><td>2186.135</td><td>1.3327</td></tr>
<tr><td></td><td></td><td></td><td></td><td></td><td></td><td>3</td><td>14.88</td><td>6.15</td><td>91.1</td><td>(K)H L/E/H L G E L G\V|A|A|A|G|A Y|A\L\H E K(H)</td><td>729.322</td><td>2186.135</td><td>-0.1838</td></tr>
<tr><td></td><td></td><td></td><td></td><td></td><td></td><td>2</td><td>14.36</td><td>3.58</td><td>84.5</td><td>(K)I E E E I/A A/A|A|A/V/G/A/G G/F A F/H E\H H E\K(K)</td><td>1246.9492</td><td>2491.2</td><td>1.6911</td></tr>
<tr><td></td><td></td><td></td><td></td><td></td><td></td><td>2</td><td>14.13</td><td>10.35</td><td>95.6</td><td>(K)H L E H L/G E\L|G V/A\A/A|G A Y|A L\H\E K(H)</td><td>1094.1018</td><td>2186.135</td><td>1.061</td></tr>
</table>

MS/MS spectra of spot 2204

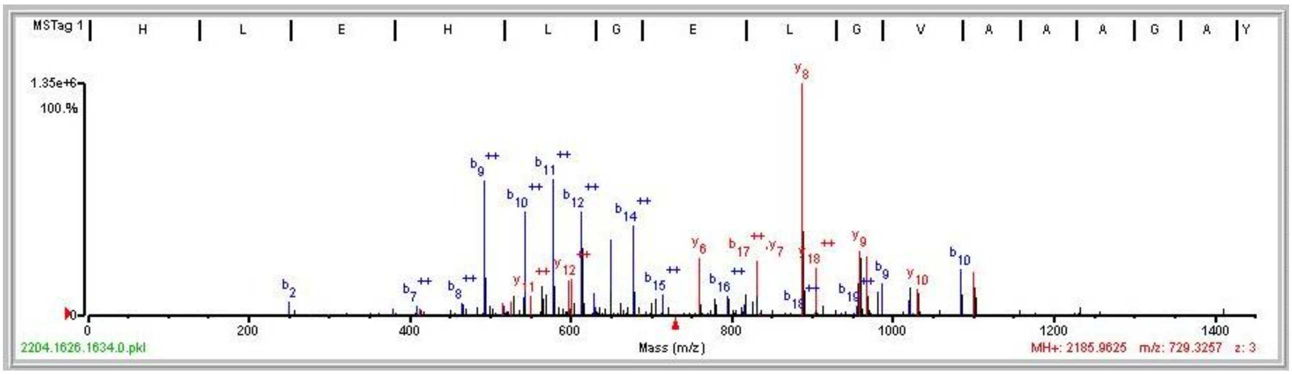

MS/MS spectra of spot 2231

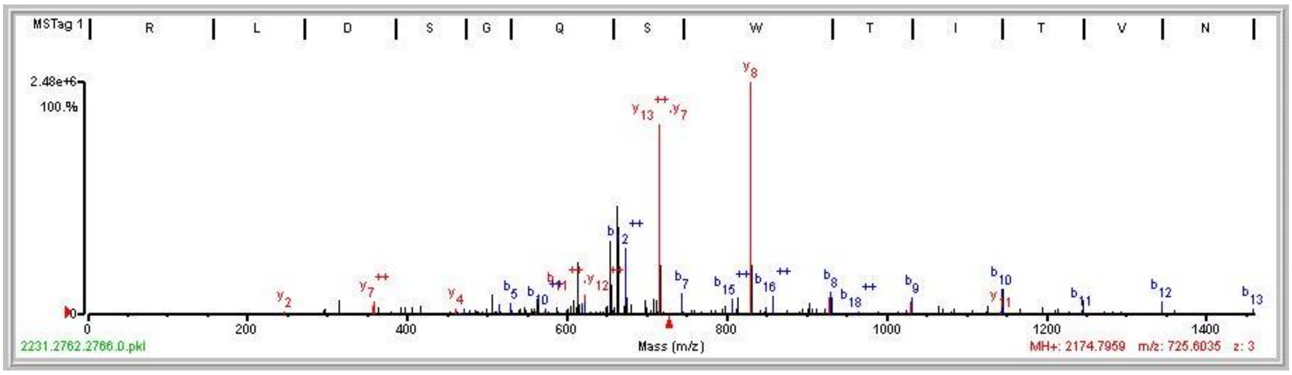

MS/MS spectra of spot 2289

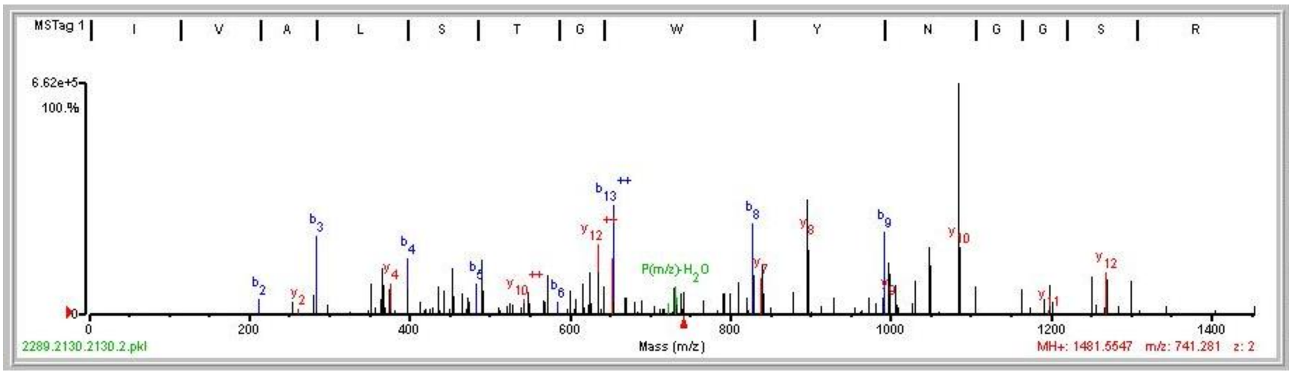

MS/MS spectra of spot 2415

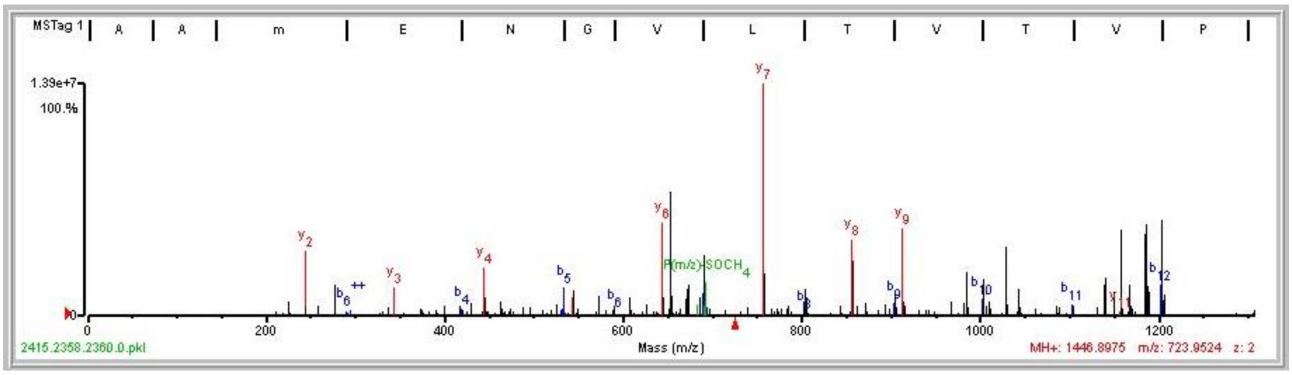

MS/MS spectra of spot 2540

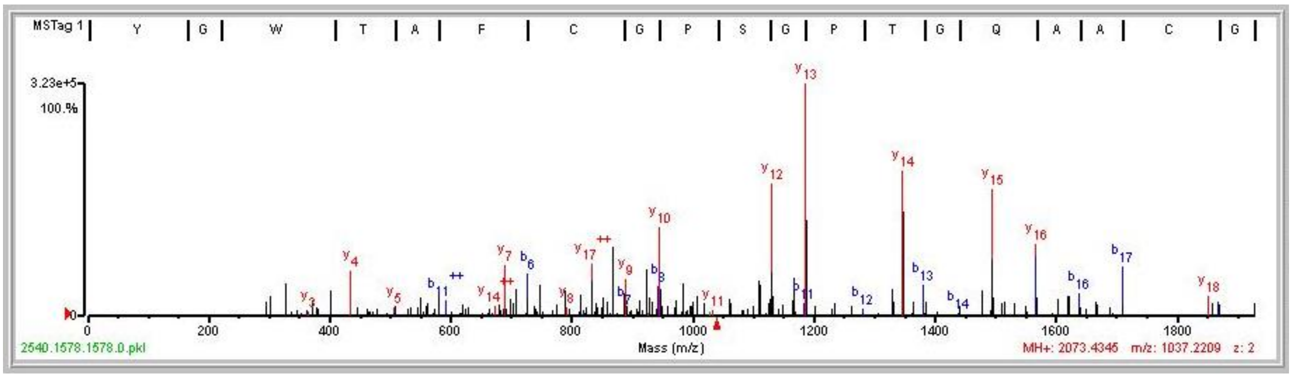

Supplement: Supplementary file 3 [file Table3.PDF]
