# Supplementary material for: Proteomic and metabolic traits of grape exocarp to explain different anthocyanin concentrations of the cultivars
Source: Front Plant Sci. 2015 Aug 4;6:603. doi: 10.3389/fpls.2015.00603 (PMC4523781; doi:10.3389/fpls.2015.00603)
Supplement: Supplementary file 4 [file Image1.PDF]

Supplementary Material - Figures

Proteomic and metabolic traits of grape exocarp to explain different anthocyanin concentrations of the cultivars

Alfredo Simone Negri[1], Bhakti Prinsi[1], Osvaldo Failla[1], Attilio Scienza[1], Luca Espen[1]*

[1]Dipartimento di Scienze Agrarie e Ambientali, Produzione, Territorio, Agroenergia (DISAA), Università degli Studi di Milano, Milano, Italia

*** Correspondence:** Luca Espen, Dipartimento di Scienze Agrarie e Ambientali, Produzione, Territorio, Agroenergia (DISAA), Università degli Studi di Milano, via Celoria n.2, Milano, 20133, Italia.
luca.espen@unimi.it

Supplementary Figure S1

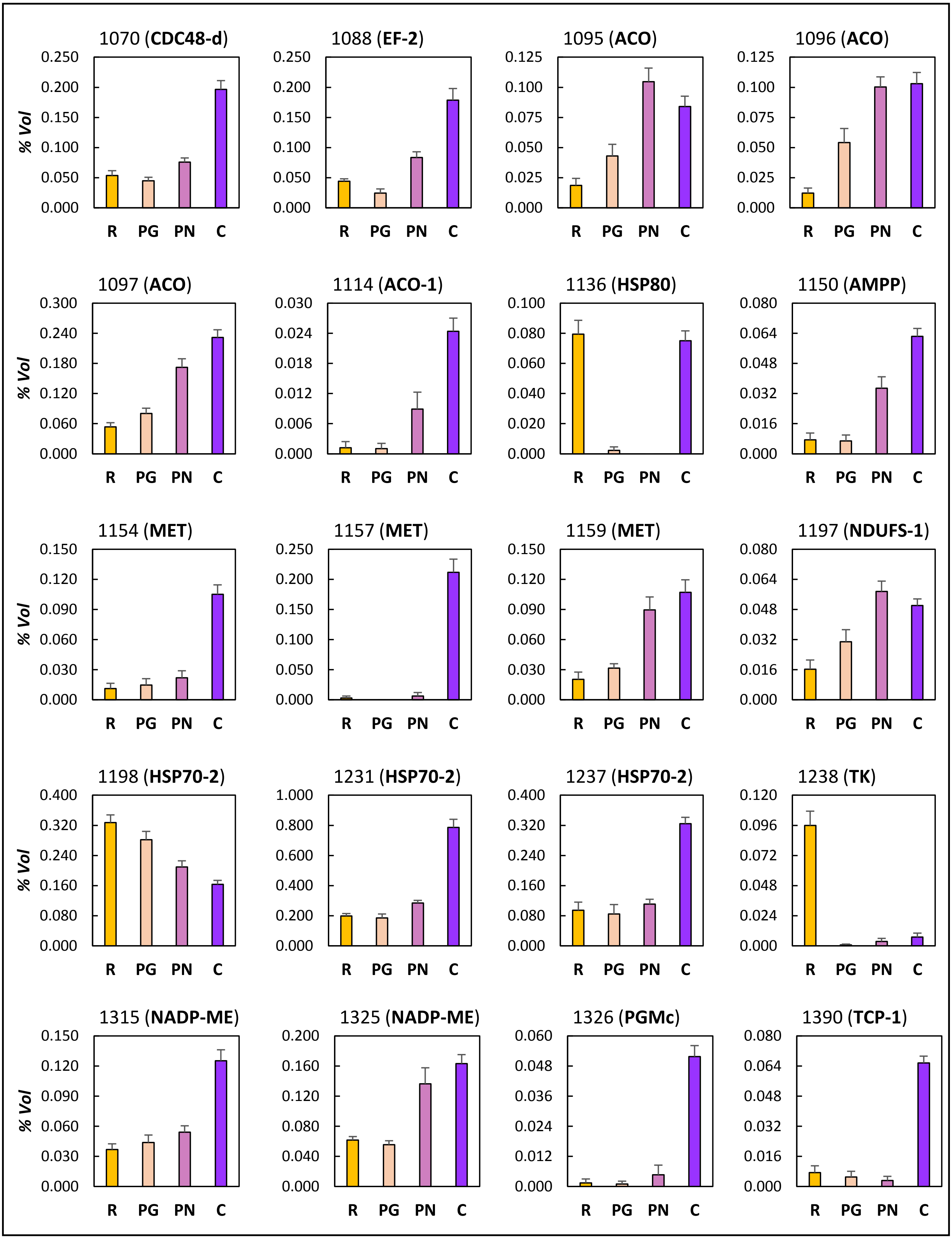

Supplementary Figure S1 (*continued*)

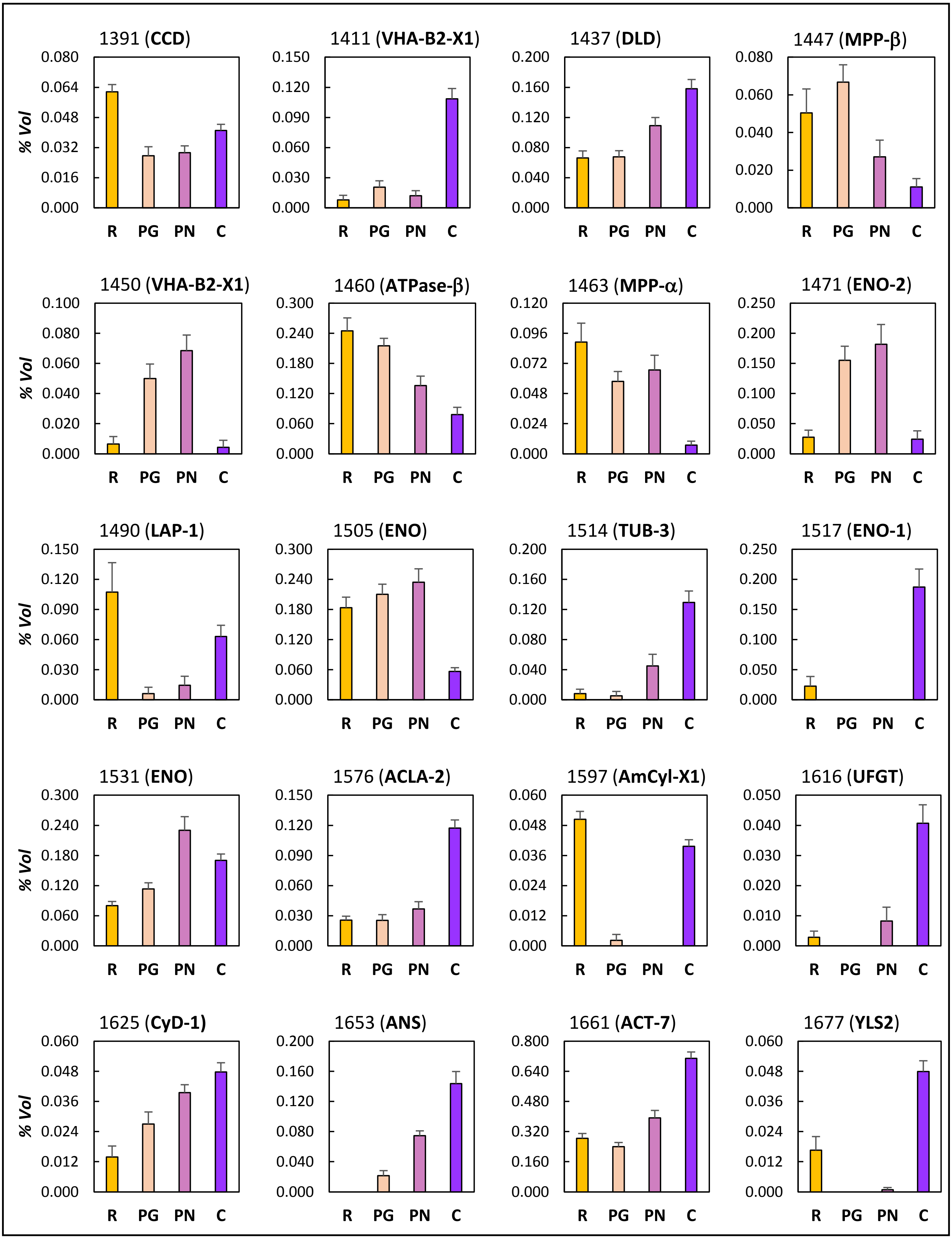

Supplementary Figure S1 (*continued*)

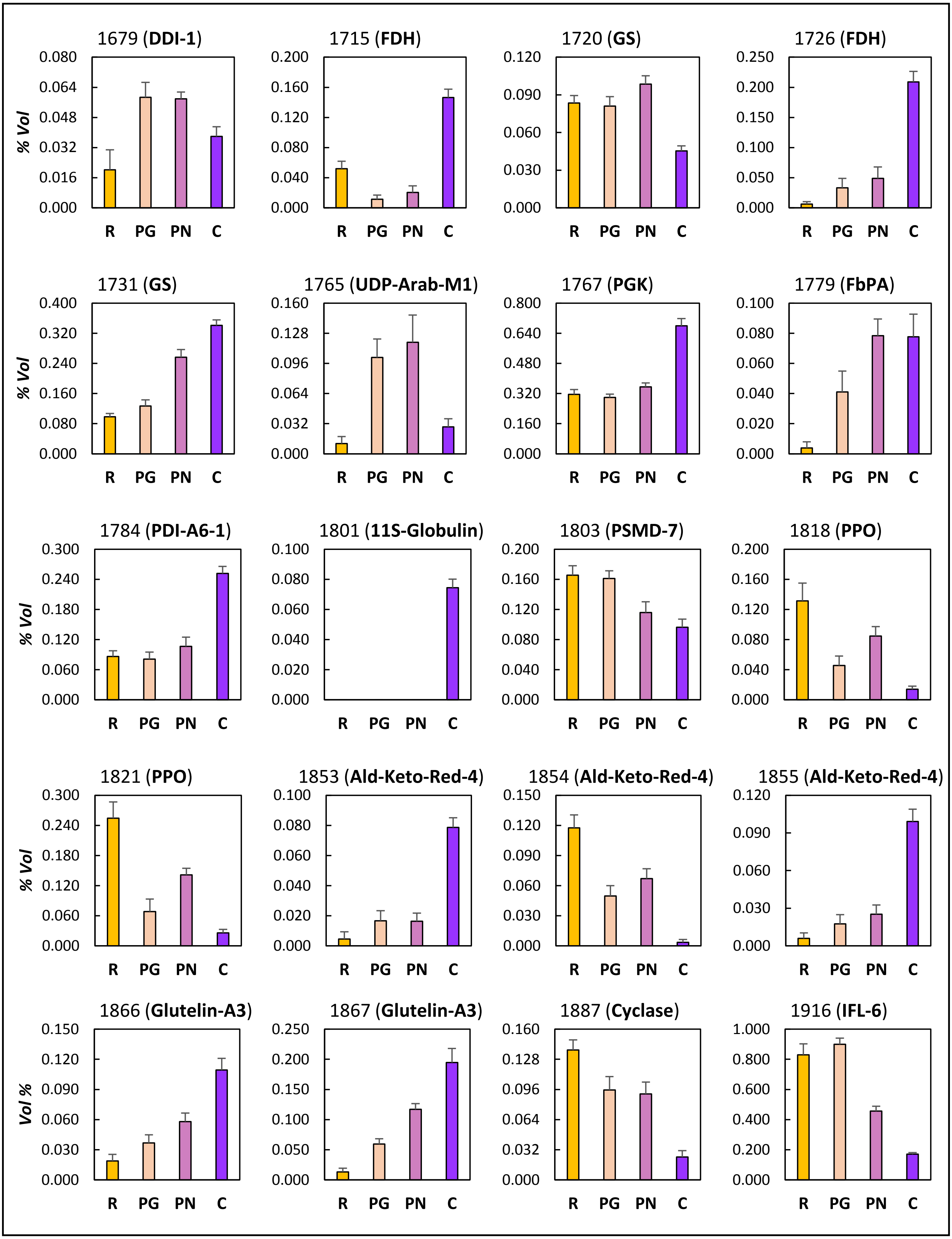

Supplementary Figure S1 (*continued*)

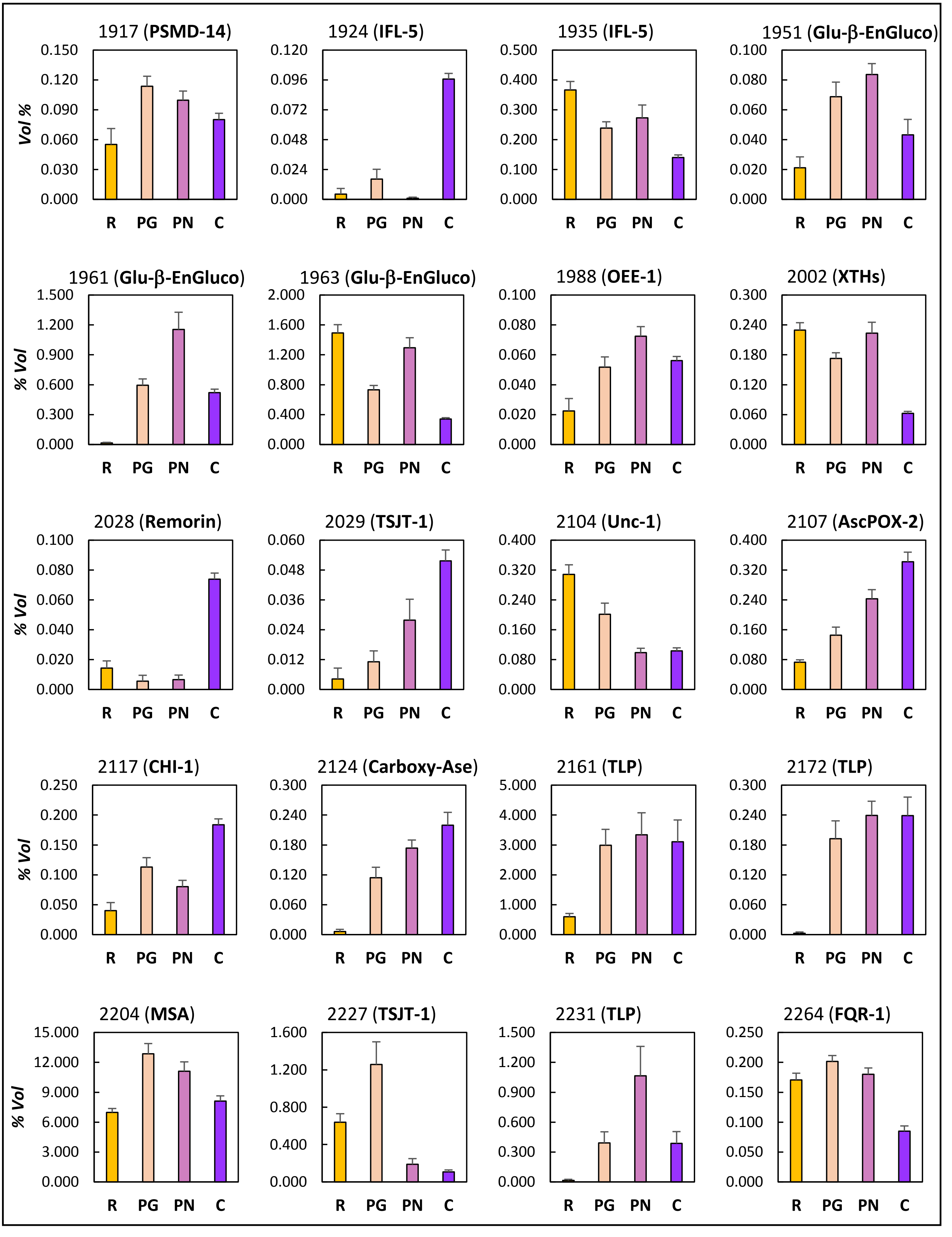

Supplementary Figure S1 (*continued*)

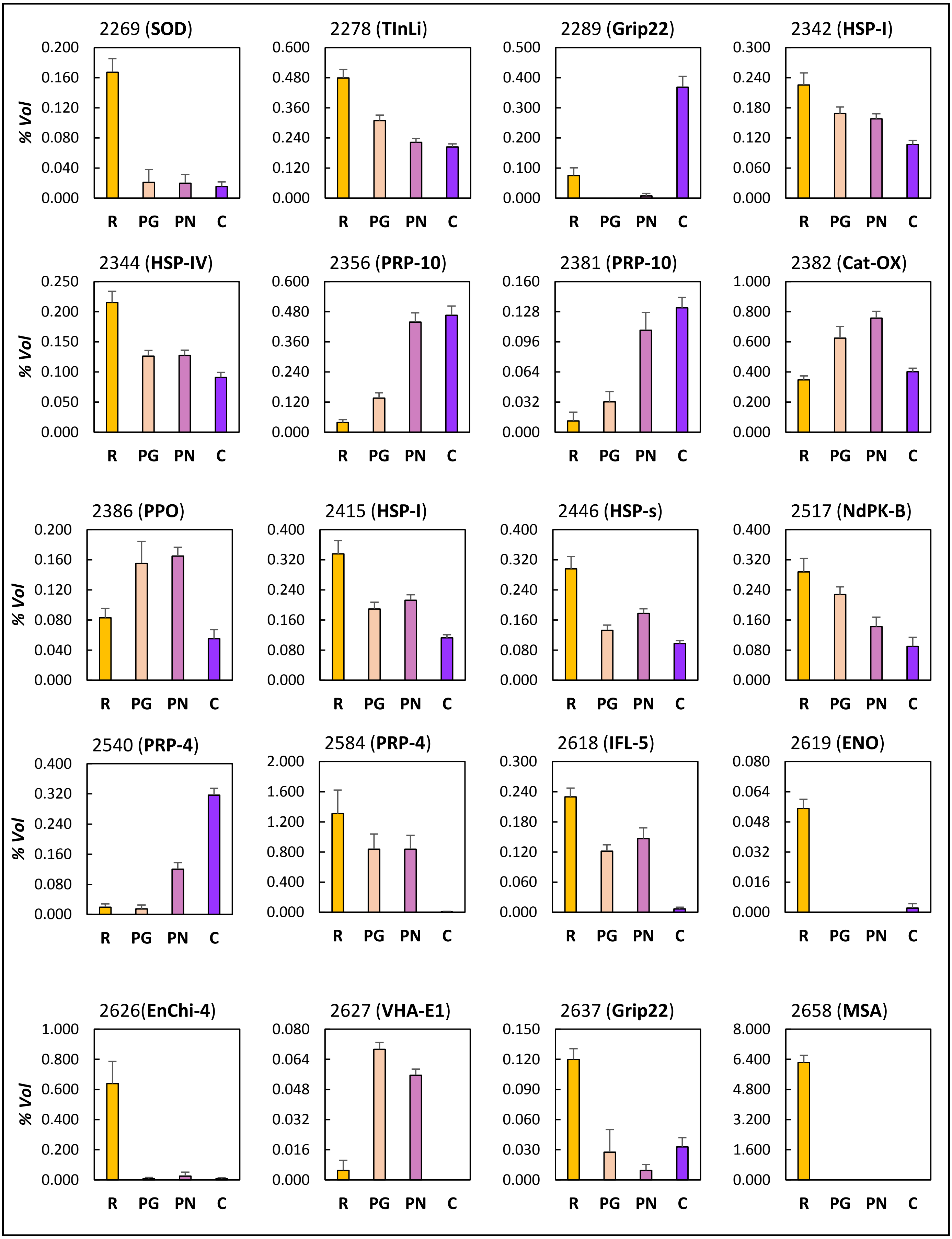

Supplementary Figure S1 (*continued*)

Supplementary figure S1. Changes in the abundance of the identified proteins. The figure shows the spot volume changes of the proteins identified by LC-ESI-MS/MS. Values are the mean ± SE of twelve 2-DE gels derived from six independent biological replicates (three for years) analyzed in duplicate. All spots showed at least a two-fold change in their relative volume at least in two of the four grape cultivars investigated. **R**, Riesling; **PG**, Pinot Gris; **PN**, Pinot Noir; **C**, Croatina.

Supplementary Figure S2

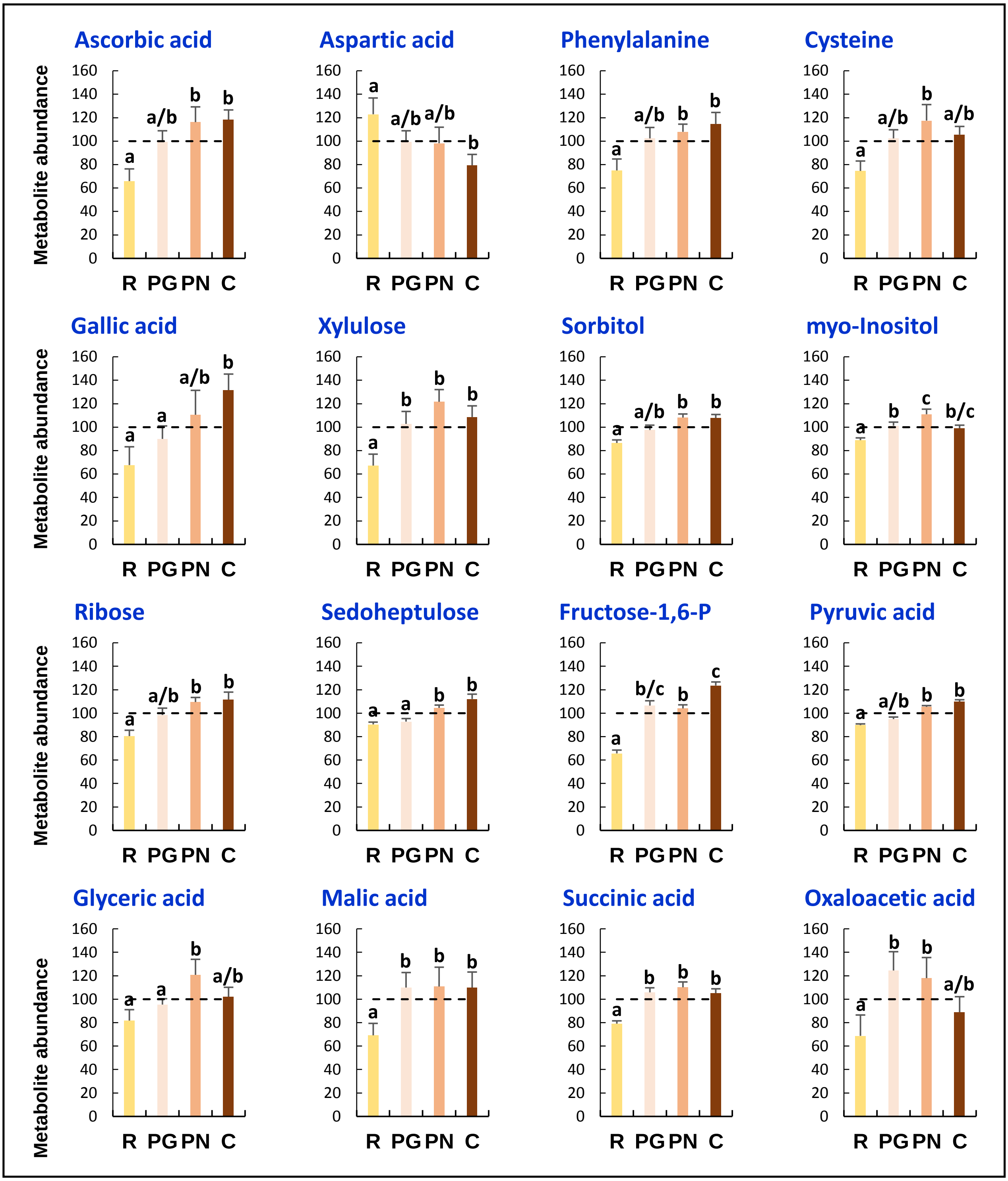

Supplementary figure S2. Contents of metabolites in the grape exocarp tissue. The figure shows the metabolites identified by GC-MS analysis that resulted significantly different to ANOVA test ($p \leq 0.05$) at least in two of the four grape cultivars investigated. **R**, Riesling; **PG**, Pinot Gris; **PN**, Pinot Noir; **C**, Croatina. Values are the mean ± SE of six biological replicates analyzed in duplicate (n=12). Samples indicated with the different letters differ significantly according to Tukey's test ($p < 0.05$).
